# Machine learning analysis identifies genes differentiating triple negative breast cancers

**DOI:** 10.1038/s41598-020-67525-1

**Published:** 2020-06-26

**Authors:** Charu Kothari, Mazid Abiodoun Osseni, Lynda Agbo, Geneviève Ouellette, Maxime Déraspe, François Laviolette, Jacques Corbeil, Jean-Philippe Lambert, Caroline Diorio, Francine Durocher

**Affiliations:** 1 Département de Médecine Moléculaire, Faculté de médecine, Université Laval, Québec City, QC Canada; 2 Centre de Recherche Sur Le Cancer, Centre de Recherche du CHU de Québec-Université Laval, 2705 Laurier Blvd, Bloc R4778, Québec, G1V4G2 Canada; 3 Big Data Research Centre, CHU de Québec-Université Laval, Quebec City, QC Canada; 4 Département D’informatique Et de génie Logiciel, Faculté des sciences et de génie, Université Laval, Québec City, QC Canada; 5 Département de Médecine Sociale Et Préventive, Faculté de Médecine, Université Laval, Québec City, QC Canada

**Keywords:** Cancer, Transcriptomics, Breast cancer, Molecular medicine

## Abstract

Triple negative breast cancer (TNBC) is one of the most aggressive form of breast cancer (BC) with the highest mortality due to high rate of relapse, resistance, and lack of an effective treatment. Various molecular approaches have been used to target TNBC but with little success. Here, using machine learning algorithms, we analyzed the available BC data from the Cancer Genome Atlas Network (TCGA) and have identified two potential genes, TBC1D9 (TBC1 domain family member 9) and MFGE8 (Milk Fat Globule-EGF Factor 8 Protein), that could successfully differentiate TNBC from non-TNBC, irrespective of their heterogeneity. TBC1D9 is under-expressed in TNBC as compared to non-TNBC patients, while MFGE8 is over-expressed. Overexpression of TBC1D9 has a better prognosis whereas overexpression of MFGE8 correlates with a poor prognosis. Protein–protein interaction analysis by affinity purification mass spectrometry (AP-MS) and proximity biotinylation (BioID) experiments identified a role for TBC1D9 in maintaining cellular integrity, whereas MFGE8 would be involved in various tumor survival processes. These promising genes could serve as biomarkers for TNBC and deserve further investigation as they have the potential to be developed as therapeutic targets for TNBC.

## Introduction

Triple negative breast cancer (TNBC) accounts for 10–20% of all breast cancers (BC). They are characterized by lack of the hormonal receptors estrogen (ER) and progesterone (PR), and the overexpression of human epidermal growth factor receptor 2 (HER2)^1^. It is the most aggressive form of BC and is very heterogeneous^2^.

The complexity of TNBC increases due to its high risk of relapse, and poor progression-free survival (PFS) and overall survival (OS)^3^. The PFS for metastatic TNBC patients is 3–4 months after treatment failure^4^. The 5-year mortality rate for early stage TNBC after surgery is 37%, whereas half of them relapse^5^.

According to gene expression pattern, TNBC has been classified in 6 different molecular subtypes namely Basal like (BL)1, BL2, Luminal androgen receptor (LAR), Immunomodulatory (IM), Mesenchymal (M) and Mesenchymal stem like (MSL), with some that cannot be classified^6^. Lehmann et al., 2011, have shown that each of these subgroups can be further divided into intrinsic subtypes of BC (Luminal A, Luminal B, HER2, normal breast like, Basal like and unclassified) based on their gene expression^6^. This stipulates why TNBC has different clinicopathological outcomes for different patients, rendering treatment arduous. On March 8, 2019, FDA approved immunotherapy Atezolizumab (targeting PD-L1) in combination with chemotherapy (nab-paclitaxel) for initial treatment of women with advanced TNBC positive for PD-L1 protein expression^7,8^. Nevertheless, there is no FDA approved target therapy for TNBC patients as a whole so far^9^. TNBC heterogeneity and aggressiveness call for an unmet need to identify genes that could serve as biomarkers to differentiate TNBC from other BCs, as well as serve as potential targets therapy irrespective of their heterogeneity.

Research groups have tried various approaches to identify biomarkers for TNBC using different techniques to study gene expression^10^. This has left us with a vast amount of data that needs to be thoroughly analyzed. One way of extracting useful information is by machine learning. Machine learning (ML) is a computer-based algorithm and statistical model which uses data as a training model, learns from the data pattern and inferences and improves with experience (number of times it reads the data), without detailed programming to do the desired task^11^. Different algorithms can be used such as Decision tree (DT), Random forest (RF) and Set covering machine (SCM). A DT uses a tree-like graph that comprises decision models consisting of all the possible outcomes^12^. RF is a classification or regression method consisting of multiple DT, where the final output is the modes (for classification) or means (for regression) of all the outputs clubbed together from every DT^12^. SCM is an algorithm whose goal is to learn a conjunction or a disjunction of rules. This is achieved by finding the decision function depending on the smallest number of attributes^13^.

In the present study, we have analyzed a dataset consisting of 877 BC patients from The Cancer Genome Atlas Program (TCGA) by three different machine learning algorithms: DT, RF and SCM. The analysis identified 20 genes, out of which two genes were characterized further, namely TBC1 Domain Family Member 9 (*TBC1D9*), a GTPase activating protein, and Milk fat globule-EGF factor 8 (*MFGE8)*, also known as lactadherin, which is a membrane glycoprotein. These identified genes were able to differentiate TNBC from non-TNBC, irrespective of their heterogeneity. The protein–protein interaction analysis highlights their potential as therapeutic targets for this highly aggressive subgroup of BC.

## Results

### Machine learning algorithms identify potential genes differentiating TNBC from non-TNBC

**Figure 1:**
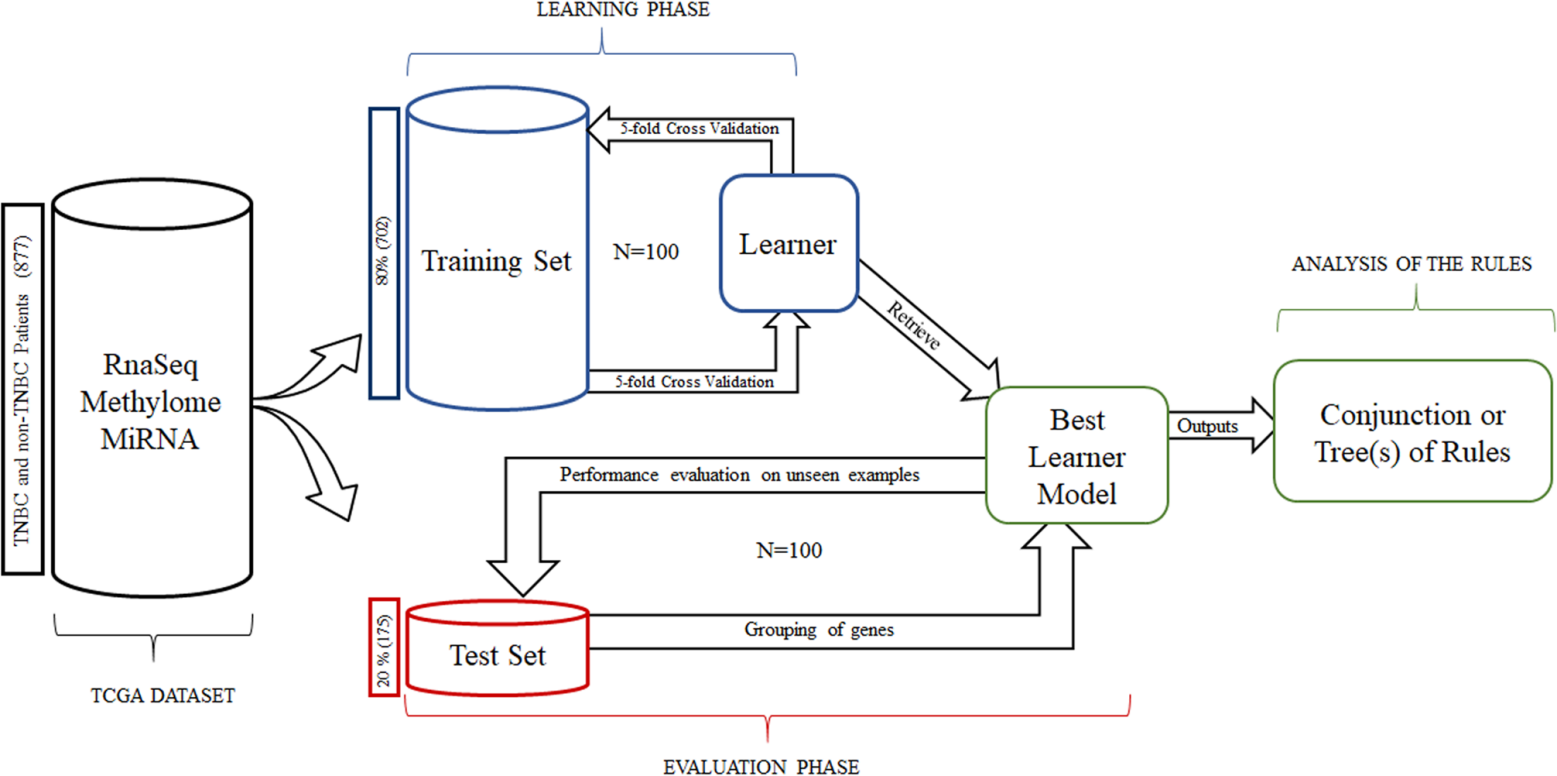
Machine learning (ML) analysis pipeline. A dataset consisting of 877 patients was selected, which comprises data from RNAseq, methylome and miRNA analysis. This dataset was divided into a training set (80% patients) and a test set (20% patients). The training set (blue) was used to train three ML algorithms: decision tree (DT), Random forest (RF) and Set-covering machine (SCM) to identify genes differently expressed in Triple negative breast cancer (TNBC) or non-TNBC. The process was repeated 100 times (N = number of repeats), to come up with the best learner model. This model was then applied to the test set (Red) to get the grouping of genes according to TNBC/non-TNBC subtypes. This process was also repeated 100 times to validate our findings. The output is a conjunction of rules for SCM and tree/(s) for DT/RF, which led to 20 potential genes.

Treatment of TNBC requires a gene or a gene set that can simply differentiate TNBC from all other BC subgroups taking into consideration the complexity of its classification. With this goal, we analyzed the TCGA-BRCA dataset from TCGA portal by three different ML algorithms, namely SCM, DT and RF (Fig. 1).

We analyzed the multiple data from 877 breast cancer patients available on TCGA. The dataset comprised 16% (140 samples) TNBC and 84% (737 samples) non-TNBC patients. In the non-TNBC subgroup, 63% were Luminal A, 16% were Luminal B and 5% were HER2 over-expressing samples. The analysis led to 20 potential genes that could differentiate TNBC from non-TNBC. From these 20 genes, 15 were down-regulated (*TBC1D9, GATA3, SLC16A6, ESR1, INPP4B, SLC44A4, ANXA9, AGR2, MCCC2, TSPAN1, STBD1, MLPH, CACNA2D2, RARA, STARD3*) and 5 were upregulated (*PPP1R14C, SFRS13B, LDHB, MFGE8, PSAT1*) in TNBC as compared to non-TNBC patients. We did not find any significant changes in methylation and miRNA data which could differentiate TNBC from non-TNBC.

### Selection of three potential genes based on BC patient’s survival outcome

**Figure 2:**
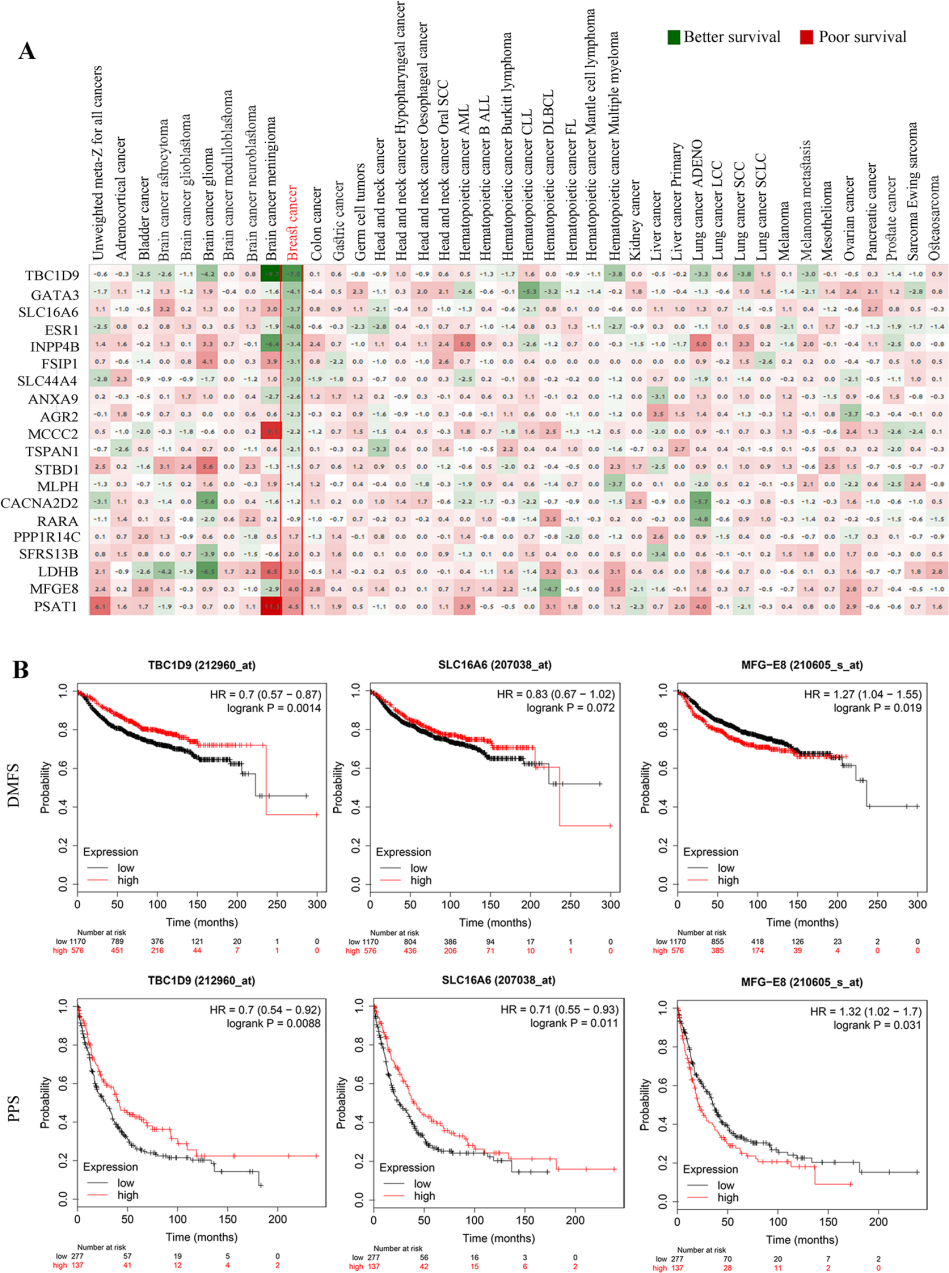
Survival analysis. (**A**) Survival analysis was performed on the 20 genes from ML analysis, in 40 different cancers using Precog meta-Z analysis. Survival z-scores are collapsed by cancer/cancer subtype as described in Gentles/Newman et al.^54^. False discovery rates corresponding to the meta-Z scores were calculated by the method of Storey and Tibshirani^55^. The data in breast cancer is highlighted in red, which is from 16 different datasets consisting of 2,164 patients. Green = Better survival; Red = Poor survival. (**B**) Kaplan–Meier plots depicting the effect of the three selected genes (*TBC1D9*, *SLC16A6* and *MFGE8*) on distance metastasis free survival (DMFS) and post progression survival (PPS). The graph was prepared by splitting patients by upper tertile. The expression of genes was determined by gene chip in kmplotter. DMFS analysis was performed in 1746 patients and PPs was performed in 414 patients.

The 20 genes identified by ML were further analyzed to come up with the most promising genes. We first analyzed the effect of these genes on survival outcome in 2,164 patients from 16 different datasets (Fig. 2A). The heatmap is based on the meta z-score obtained when survival Z scores are collapsed by cancer/cancer subtype. We then selected the top three genes with the best or the worst effect on survival outcome, and with the maximum repeats in ML analyses. This led to the following three genes: *TBC1D9* (TBC1 domain family number 9), *SLC16A6* (Solute Carrier Family 16 Member 6) and *MFGE8* (Milk Fat Globule-EGF Factor 8 Protein). Expression of *TBC1D9* and *SLC16A6* had better survival outcome among BC patients whereas expression of *MFGE8* had poor survival outcome (Fig. 2A).

We further investigated the outcome of these three genes on survival of BC patients for which we used the online tool developed by Gyorffy et al., known as KM (Kaplan–meier) plotter (Fig. 2B)^14^. The analysis showed that BC patients with high expression of *TBC1D9* had better survival outcome for distance metastasis free survival (DMFS) and post-progression survival (PPS), with a p-value of 0.0014 and 0.0088 respectively. *SLC16A6* also showed similar results for both DMFS (p-value = 0.072) and PPS (p-value = 0.011), but the p-value for DMFS was not significant. On the other hand, BC patients with high expression of *MFGE8* had poor survival outcome for both DMFS (p-value = 0.019) and PPS (p-value = 0.031).

### The three selected genes effectively differentiate TNBC from non-TNBC in different patient cohorts

**Figure 3:**
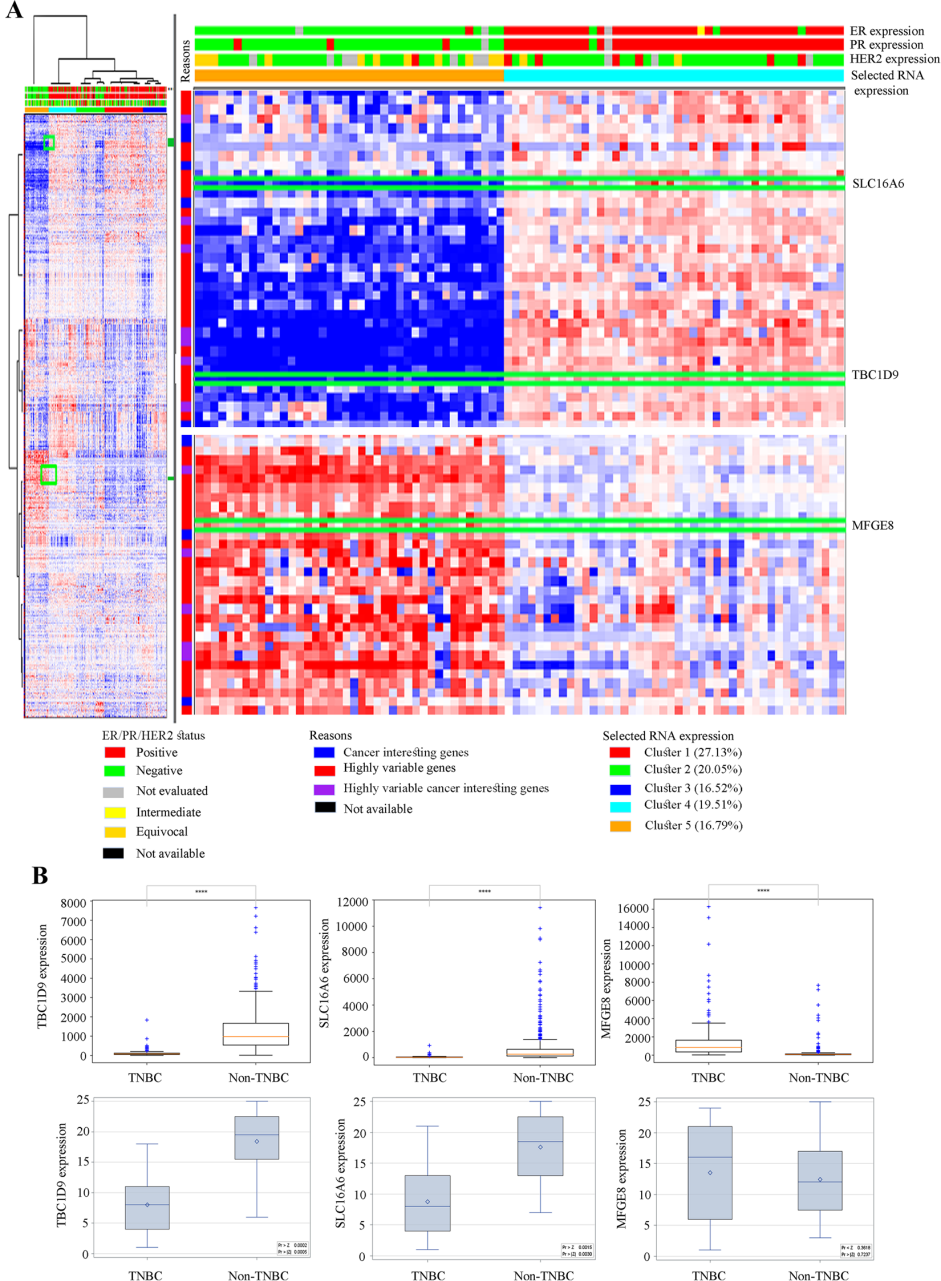
Analysis of the potential of the three selected genes to differentiate TNBC from non-TNBC based on their expression pattern. (**A**) The TCGA provisional dataset (tcga_rnaseqV2_brca_v2.0_gene_sample) consisting of 1,101 patients and 1,108 samples was analyzed in cbioportal to see the difference in the expression level of the three selected genes (*TBC1D9*, *SLC16A6* and *MFGE8*) in TNBC and non-TNBC patients. (**B**) mRNA expression of the selected genes in TNBC and non-TNBC patients. The first row shows the data from 877 patients from the dataset TCGA-BRCA (nationwidechildrens.org_clinical_patient_brca.txt), which consists of 140 TNBC and 737 non-TNBC patients. The second row is the expression in patients from our tissue repository (TNBC = 13; non-TNBC = 12). The statistical analysis score is the distribution of Wilcoxon rank sum test. **** = p value < 0.0001.

Using the TCGA provisional dataset from the cBioPortal for cancer genomics, the expression pattern of the three selected genes in 1,101 patients was verified (Fig. 3A). Based on their expression pattern, *TBC1D9* and *SLC16A6* expression were higher in non-TNBC, whereas *MFGE8* was more expressed in TNBC patients.

The expression level of these genes was also verified in the original dataset consisting of 877 patients and the same result was obtained (Fig. 3B). To validate our findings in an independent cohort, 13 TNBC and 12 non-TNBC patients were selected from the tissue bank of Centre des Maladies du Sein (Hôpital du St-Sacrement, Quebec, Canada) with the aid of senior pathologists, and the expression of these three genes was verified by qPCR in these samples. The expression pattern in these samples further confirmed our findings (Fig. 3B). For MFGE8 expression, we obtained a p-value of 0.72 when comparing non-TNBC (Luminal A, Luminal B and HER2) to TNBC. However, a p-value of 0.16 was obtained when the expression of MGFE8 was compared in the non-TNBC (excluding HER2 subgroup) vs TNBC group.

### Protein–protein interaction analysis highlights a role for TBC1D9 in maintaining cellular integrity

The evidence of association of TBC1D9 expression with better survival of BC patients led us to explore the role of this protein with regard to its interacting partners. Hence, AP-MS and BioID experiments were performed to identify their interactors and in turn understand their role in biological processes. The data obtained from AP-MS or BioID experiments were analyzed using SAINTexpress. Enforcing a SAINTexpress BFDR cutoff of ≤ 0.01, 68 and 77 significant interactors were identified by AP-MS and BioID, respectively (Supplementary Tables 1 and 2). These genes were further filtered for possible non-specific interactors utilizing the Crapome portal yielding final datasets of 52 and 67 significant protein interactors by AP-MS and BioID, respectively (Supplementary Table 3). Compared with the already known interactors from Biogrid (https://thebiogrid.org/) (Supplementary Fig. 1A), four proteins were identified by AP-MS (MAP1LC3B, ARL8A, CPT1A and SRSF2), one with BioID (ABHD16A) and five with both AP-MS and BioID (PRPF38B, DDX41, YME1L1, SSB and SNRPE). Out of them, only two proteins were significant according to our cut-off: ARL8A (BFDR = 0) and ABHD16A (BFDR = 0.01) (Supplementary Tables 1 and 2).

**Figure 4:**
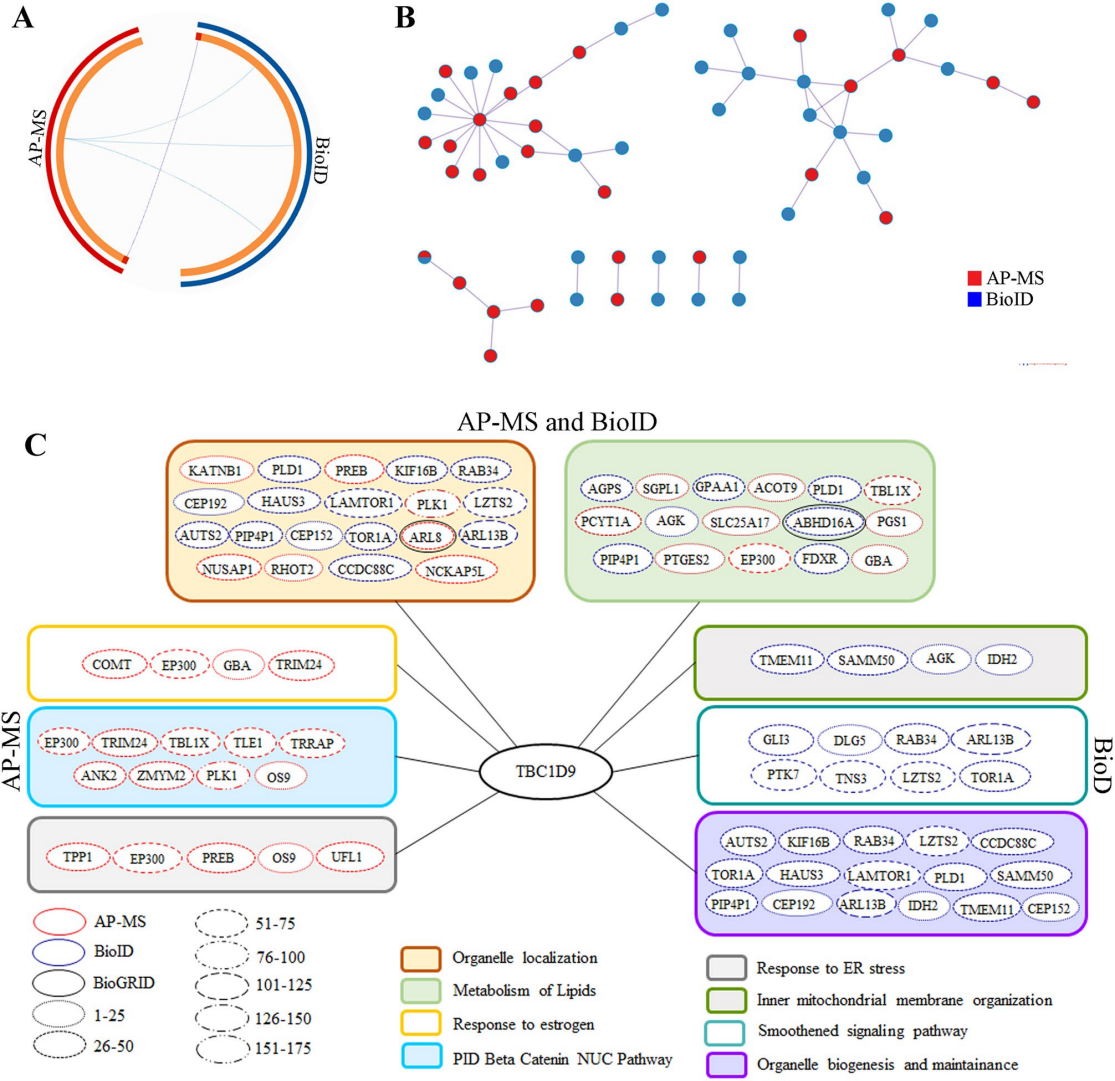
Protein–Protein interaction (PPI) network based on AP-MS and BioID data in TBC1D9. (**A**) The Circos plot shows how genes identified in AP-MS and BioID overlap. On the outside, each arc represents the identity of each gene list, Red = AP-MS; Blue = BioID. On the inside, each arc represents a gene list, where each gene has a spot on the arc. Dark orange color represents the genes that appear in multiple lists and the light orange color represents genes that are unique to that gene list. Purple lines link the same genes that are shared by multiple gene lists. The greater the number of purple links and the longer the dark orange arcs are, imply greater overlap among the input gene lists. (**B**) All input gene lists were merged into one list and resulted in a PPI network. Network nodes are displayed as pies. Color code for pie sector represents a gene list (AP-MS/BioID). (**C**) Pathways affected by TBC1D9; pathways identified by AP-MS, BioID or both. AP-MS = Affinity purification coupled to mass spectrometry; BioID = Proximity-dependent biotinylation assay.

The proteins identified by both methods were further analyzed using the metascape online tool to better understand their roles. The circos plot in Fig. 4A depicts the comparison analysis of the data obtained with AP-MS and BioID. The red part of the circle represents the AP-MS data whereas the blue is for BioID data. The inner circle (orange) represents each protein identified. The dark orange colour represents proteins that appear in multiple lists. The proteins overlapping in different gene ontology (GO) terms are connected with the purple line. To understand which pathways these proteins affect, a comprehensive protein–protein interaction (PPI) network was generated by metascape involving both AP-MS and BioID data, based on this interaction network (Fig. 4B). The unique PPI from metascape applies Molecular Complex Detection (MCODE) algorithm to the resultant networks to identify tightly connected network cores. Then it analyzes each network component for pathway enrichment and based on them, finally assigns biological functions. The analysis highlighted the enrichment of pathways related to metabolism of lipids and organelle localization by both AP-MS and BioID (Fig. 4C). The processes affected by metabolism of lipid pathway are metabolism of lipids (logP = − 5.6), glycerophospholipid metabolic process (logP = − 3.0), and glycerolipid metabolic process (logP = − 2.3) (Supplementary Table 4). For organelle localization the major processes affected are organelle localization (logP = − 3.8), microtubule-based processes (logP = − 3.5), loss of Nlp from mitotic centrosomes (logP = − 3.4), AURKA activation by TPX2 (logP = − 3.4), centrosome maturation (logP = − 3.2), regulation of PLK1 activity at G2/M transition (logP = − 3.1), and recruitment of NuMa to mitotic centrosome (logP = − 2.9) ( Supplementary Table 4).

The pathways highlighted by AP-MS are response to estrogen (logP = − 4.7), PID beta catenin NUC pathway (logP = − 4.6), response to ER stress (logP = − 3.5), and the ones identified by BioID are inner mitochondrial membrane organization (logP = − 3.6), smoothened signaling pathway (logP = − 3.3) and organelle biogenesis and maintenance (logP = − 4.9) ( Supplementary Table 4).

### Protein–protein interaction analysis highlights a role for MFGE8 in many oncogenic processes

AP-MS and BioID experiments were performed for MFGE8 to explore its protein–protein interaction network to better understand the high expression of MFGE8 in TNBC and its correlation with poor prognosis in breast cancer. The analysis of AP-MS and BioID were done in a similar way as TBC1D9. One hundred and thirty-eight (138) significant interactors (BFDR ≤ 0.01) were obtained by AP-MS, and 12 by BioID (Supplementary Tables 5 and 6). These interactors were further filtered by Crapome data, which led to 123 interactors by AP-MS, and 9 interactors by BioID (Supplementary Table 7). When compared with the known interactors from Biogrid (Supplementary Fig. 1B), one protein by AP-MS (ABCE1) was found, two proteins by BioID (YTHDF2 and MTDH) and one was found by both (FUS), but none fell in the cutoff of BFDR ≤ 0.01 (Supplementary Tables 5 and 6).

**Figure 5:**
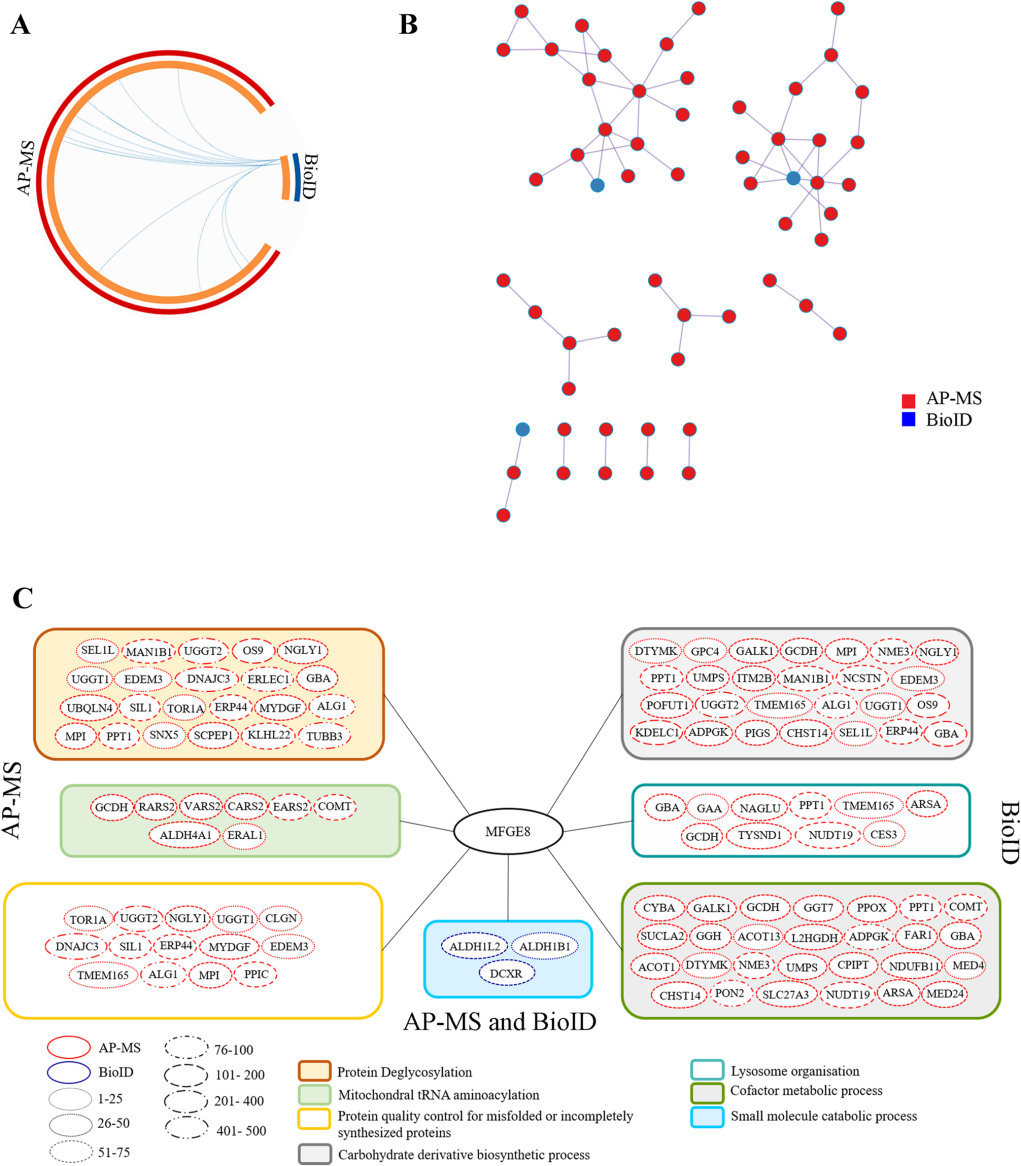
Protein–Protein interaction (PPI) network based on AP-MS and BioID data in MFGE8. (**A**) The Circos plot shows how genes identified in AP-MS and BioID overlap. On the outside, each arc represents the identity of each gene list, Red = AP-MS; Blue = BioID. On the inside, each arc represents a gene list, where each gene has a spot on the arc. Dark orange color represents the genes that appear in multiple lists and the light orange color represents genes that are unique to that gene list. Purple lines link the same genes that are shared by multiple gene lists. The greater the number of purple links and the longer the dark orange arcs are, imply greater overlap among the input gene lists. (**B**) All input gene lists were merged into one list and resulted in a PPI network. Network nodes are displayed as pies. Color code for pie sector represents a gene list (AP-MS/BioID). (**C**) Pathways affected by MFGE8; pathways identified by AP-MS, BioID or both. AP-MS = Affinity purified mass spectrometry; BioID = Biotin identification.

Protein interactors for MFGE8 were also analyzed by metascape online tool to identify the biological processes MFGE8 is involved into. Not many significant interactors were found by BioID which is depicted in circos plot Fig. 5A, where red part is AP-MS and blue is BioID. Few protein interactors sharing the GO terms were found, which is represented by purple colour lines in the circos plot. A PPI network was prepared to identify biological functions (Fig. 5B). According to the interactors identified by AP-MS, an enrichment of protein deglycosylation (logP = − 9.8), carbohydrate derivative biosynthetic process (logP = − 9.4), mitochondrial tRNA aminoacylation (logP = − 5.4), protein quality control for misfolded or incompletely synthesized proteins (logP = − 4.9), cofactor metabolic process (logP = − 4.9) and lysosome organization (logP = − 4.8) (Fig. 5C) were identified. For BioID, an enrichment of small molecule catabolic process was obtained (logP = − 3.3) (Fig. 5C, Supplementary Table 8). Besides, few proteins by BioID involved in cofactor catabolic process (ALDH1L2, DXDR), protein deglycosylation (TRIM13, DAD1) and carbohydrate derivative catabolic process (TRIM13, DAD1 and DXDR) were also identified (Fig. 5C).

## Discussion

TNBC, the most heterogeneous and aggressive BC, lacks any effective therapy to date. TNBC response to neoadjuvant therapy looks promising on first look, and accounts for a pathological complete response (pCR) of around 30–40% at the time of surgery^5,15^. Yet, any traces of the residual disease after neoadjuvant therapy results into a 6 times higher risk of relapse and a more than 12 times risk of metastasis^5,16^. Moreover, the mean survival time for patients who relapsed is less than 13 months^17^. Additionally, response to chemotherapy further varies with different subtypes of TNBC. According to Masuda et al., BL1 has the highest pCR rate (52%), whereas BL2 and LAR subgroups show the lowest pCR rate (0% and 10% respectively)^16^. If the intrasubtype variation is added, it further complicates the outcomes of treatments. Many studies have been reported to understand the molecular traits of TNBC to come up with potential therapeutic targets^18,19^. Many of these targets are under clinical trials and target growth factor receptors (EGFR, cMET, VEGFR), downstream signalling (PI3K/mTOR pathway, SRC, WNT signaling), cell cycle checkpoints (CHK1/2), PARP inhibitors, the androgen receptor and so on (ClinicalTrials.gov). Most are effective only for a subgroup of breast cancer and yet have not been very promising due to many other underlying factors. In this study, we have identified genes that could differentiate TNBC from other BC, irrespective of their heterogeneity.

In this study, we have taken advantage of the vast amount of available data stored in TCGA. We selected a dataset consisting of 63% Luminal A, 16% Luminal B, 5% HER2+ and 16% TNBC, representative of the BC prevalence. The analysis of the dataset by ML led to 20 potential genes differentiating TNBC from non-TNBC. We identified 15 downregulated and 5 upregulated genes in TNBC as compared to non-TNBC. Of most significant importance is the identification of ESR1 (estrogen receptor) which was downregulated in TNBC, further confirming the efficacy of ML analysis. These genes were further evaluated for their survival outcome across 40 different cancers. Most strikingly, the identified genes gave a trend of better or poor survival outcome only in BC patient samples based on their expression pattern (Fig. 2A). Further analysis of the three selected genes (based on survival outcome and number of repeats by ML analysis), *TBC1D9, SLC16A6* and *MFGE8*, showed that each has an effect on DMSF and PPS (Fig. 2B), where the expression of the first two genes (*TBC1D9* and *SLC16A6*) have better survival outcome. On the opposite, *MFGE8* displays poor survival outcome. Since TNBC is the most aggressive form of BC and the chances of metastasis and relapse are very high, this finding suggests that these genes might be playing an utmost important role in the TNBC recurrence and spread.

The analysis of TCGA-BRCA RNAseq dataset confirmed that these genes are indeed able to differentiate TNBC from non-TNBC patients (Fig. 3A). The expression of these three genes was further validated in tissue samples. The same expression pattern as in the ML analysis, i.e. *TBC1D9* and *SLC16A6* were downregulated in TNBC, whereas *MFGE8* was upregulated, was obtained (Fig. 3B), particularly after exclusion of HER2 subtype samples, although statistical significance was not reached because the sample size was too small. These results are not surprising since the expression of several genes was found to be highly correlated with HER2 status measured at the RNA levels (TCGA analysis), but less correlated at the protein levels (samples analysis)^20^.

TBC1D9 is a GTPase activation protein whose expression has been shown to be linked to low mortality and recurrence in breast cancer^21^. SLC16A6 is a transporter for monocarboxylates across the plasma membrane. Polymorphisms in *SLC16A6* gene have been reported in breast cancer^22^. MFGE8, also known as lactadherin, is known to promote phagocytosis of apoptotic cells and has been shown to induce the tumorigenic potential of mammary epithelial cells^23^.

**Figure 6:**
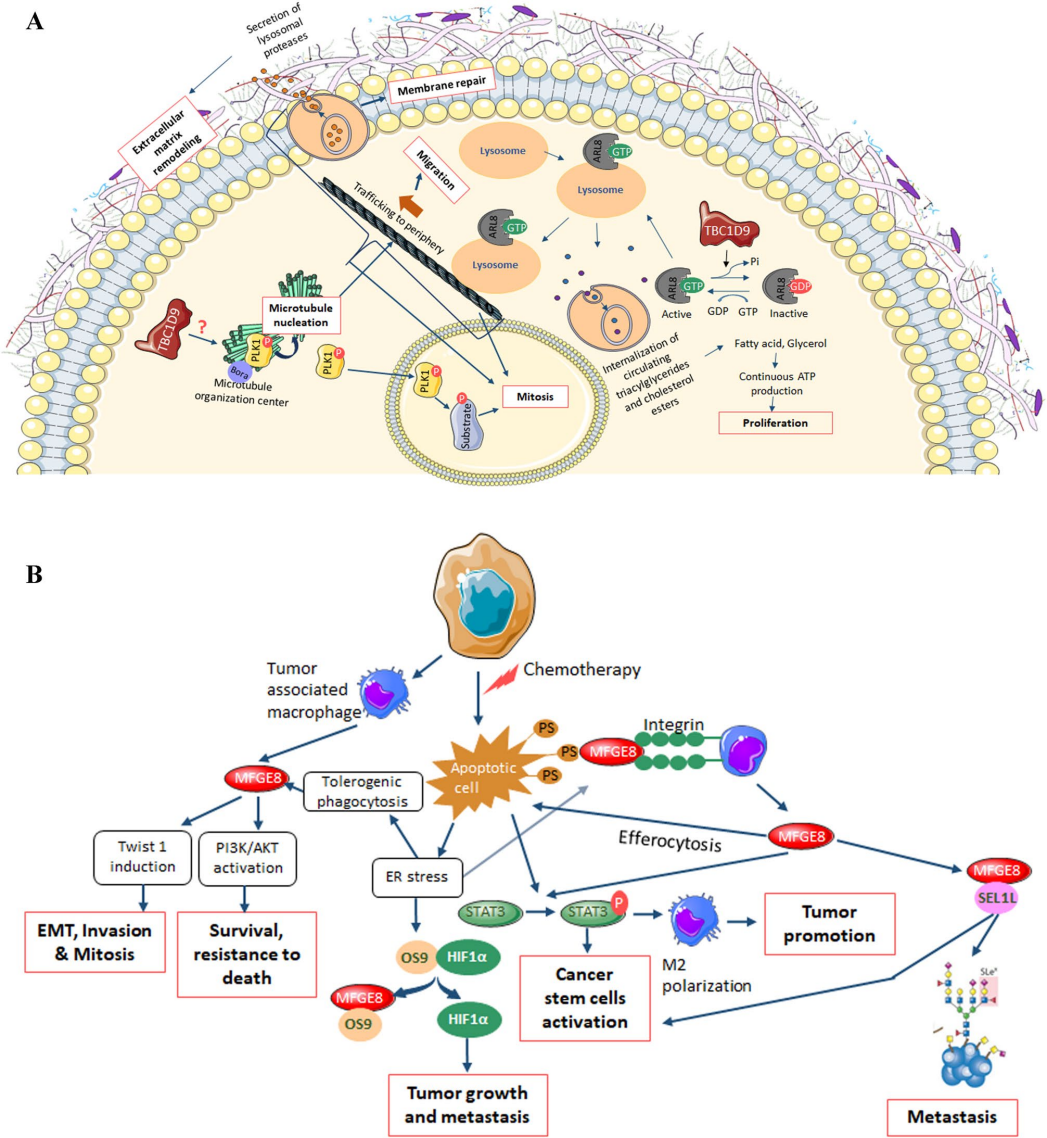
Schematic representation of the possible roles of TBC1D9 (**A**) and MFGE8 (**B**). (**A**) TBC1D9, a GTPase activating protein, inactivates ARL8. When active, ARL8 localizes on the lysosomal membrane and regulates the lysosomal positioning on microtubules that leads to trafficking of lysosome to periphery, therefore resulting in cell migration. ARL8 is essential for membrane repair and extracellular matrix remodelling by controlling the exocytosis of lysosome^25^. ARL8 bound lysosomes are also recruited to the circulating triacylglycerides and cholesterol esters, which are internalized by lysosomes and broken down into fatty acids and glycerol, respectively, leading to continuous ATP production essential for cell proliferation. A study have also shown that lysosomal exocytosis is essential for correct mitosis^26^. PLK1 has a role in microtubule nucleation and mitosis. In the present study, a PLK1 interaction with TBC1D9 has been identified, which could possibly lead to inactive PLK1 by controlling its disassociation from its partners. This hypothesis however needs to be verified. (**B**) MFGE8 is known to interact with phosphatidylserine which is expressed in the apoptotic cells. After interaction, the complex binds to the integrin on the macrophages. This binding results in more secretion of MFGE8. MFGE8 activates effrocytosis of apoptotic cells. The process of effrocytosis and MFGE8 itself activates STAT3, leading to M2 polarization, tumor promotion and cancer stem cell activation. The apoptotic signaling also induces ER stress, resulting in tolerogenic phagocytosis, which also secretes MFGE8. This leads to EMT, invasion, mitosis by twist induction or survival and resistance to death by activation of the PI3K/AKT pathway. Upon ER stress induction, HIF1α is induced. This might be due to the interaction of secreted MFGE8 with HIF1α inhibitor OS9, therefore releasing HIF1α, resulting in tumor growth and metastasis. MFGE8 also binds to SEL1L, whose increased expression has been seen in metastasis and cancer stem cell activation. The mechanism by which MFGE8 might regulate SEL1L is an open question.

We further investigated the two promising genes *TBC1D9* and *MFGE8* to uncover their role in TNBC. The interactors of TBC1D9 showed that it has a role in organelle localization, metabolism of lipids and organelle biogenesis and maintenance. The most important interactor of TBC1D9, ARL8A (ADP Ribosylation Factor Like GTPase 8A, fold change = 30), which has been also identified as an interactor of TBC1D9 by AP-MS (Biogrid data), is a GTPase known to bind to lysosome and therefore recruiting lysosome to the microtubule for its trafficking to periphery, resulting in cell migration^24^. Lysosome trafficking leading to exocytosis helps in extracellular matrix remodelling and membrane repair during cancer^25^. Nugues et al., have suggested that exocytosis by lysosome has an important role in mitosis^26^. It has been shown that upon binding of ARL8 to the lysosome, the lysosome is recruited to the cytoplasm where it internalizes circulating triacylglycerides and cholesterol esters to release fatty acids and glycerol, leading to continuous production of ATP required for rapid proliferation in cancer cells^27^. TBC1D9, which is a GTPase activating protein, acts on ARL8A (a GTPase) by inactivating this protein, therefore regulating proliferation, migration, membrane repair, extracellular matrix remodelling and mitosis (Fig. 6A). We have also identified PLK1 (polo-like kinase 1, fold change = 155) as an interactor of TBC1D9, which has a role in microtubule nucleation resulting in microtubule formation^28,29^, therefore affecting trafficking of lysosome. Active PLK1 enters nucleus where it plays an important role in mitosis^30,31^. It might be possible that TBC1D9 inhibits the activation of PLK1, and therefore regulating these processes, but this will need to be further evaluated.

As for MFGE8, it is known to interact with the phosphatidylserine (PS)-enriched surfaces, mostly labeling the apoptotic cells. After interacting with PS, it binds to integrin on the macrophage leading to M2 polarization by activation of STAT3 signalling resulting in tumor promotion and pro-oncogenic inflammatory response^32^ (Fig. 6B). Vallabhapurapu et al., have shown that many viable cancer cells express PS on its surface, which is recognized by macrophages resulting in immunity to antitumor drugs^33^. Furthermore, these tumor associated macrophages secrete MFGE8^34^, which has been shown to increase epithelial to mesenchymal transition (EMT), invasion and mitosis by activating Twist 1 (Twist-related protein 1)^35^, and survival and resistance to stress by activating PI3k/AKT (Phosphoinositide 3-kinase/Protein kinase B) pathway^35^. Our data have highlighted a role for MFGE8 in mitochondrial tRNA aminoacylation, through its interactions with many aminoacyl t-RNA synthetases which could result in regulating tRNA maturation and proofreading, RNA splicing, amino-acid editing, and tmRNA aminoacylation protein synthesis^36^, resulting in regulating the shift from oxidative to glycolytic metabolism, prominent in cancer cells via activation of the PI3K–PTEN–AKT pathway^36^. We have also found that MFGE8 interacts with OS9 (Osteosarcoma Amplified 9, Endoplasmic Reticulum Lectin). OS9 interacts with HIF1α (Hypoxia Inducible Factor 1 Subunit Alpha) and leads to the degradation of HIF1α^37^. In a cancer hypoxic condition, MFGE8 might interact with OS9 and therefore releasing HIF1α, resulting in tumor growth and metastasis. However, this concept will need further evaluation. On the other hand, MFGE8 also interacts with SEL1L (Suppressor of Lin-12-Like Protein 1), whose role is in protein glycosylation. Protein glycosylation is an important event in cancer progression, as incorrect glycosylation can lead to cancer progression and metastasis^38^.

The search to identify genes differentiating TNBC from non-TNBC has led to the identification of two potential genes, i.e. *TBC1D9* and *MFGE8.* The bioinformatics results show that TBC1D9 might have a role in maintaining cellular integrity and therefore its expression is related to better survival outcome, whereas MFGE8 has a role in several oncogenic processes resulting in poor survival outcome in BC patients.

The major problem in treating TNBC is its heterogeneity. Even the 6 molecularly classified (or after refinement four classes) TNBC subgroups display gene expression resembling other BC. As each patient shows different characteristics, the genes identified in this study serve the long desiring aim of finding common patterns across all TNBC patients, as these could be further developed as potential therapeutic targets. Targeting *MFGE8* in TNBC would be possible as it is overexpressed in TNBC. Hence it could be downregulated by using MFGE8 specific inhibitors. Moreover, effector molecules of MFGE8 could also be targeted. However, *TBC1D9* is downregulated in TNBC. Therefore the question arises as to how to appropriately target a gene which is downregulated in a disease. This could be performed with various approaches such as targeting its regulators, by gene therapy, a vaccine approach, or by inhibiting the activated pathways (in this case ARL8A) due to inhibition of TBC1D9. These approaches are currently being investigated for various tumor suppressor genes and are of great utility^39,40^.

The approach described in this study combines multiple disciplines linking clinical information, -omics data, machine learning algorithms and bioinformatics tools, and has proved to be useful and adequate to provide candidate genes that deserve to be pursued further.

## Material and methods

### Materials

Constructs for the genes of interest were generated via Gateway cloning into pDEST 3′ 3xFLAG-pcDNA5-FRT-TO or pDEST 3′ BirA*-FLAG-pcDNA5-FRT-TO according to Lambert et al. for TBC1D9 and MFGE8^41^. The pDEST-TBC1D9-BirA*-FLAG or 3xFLAG vectors were cloned from pENTR223-TBC1D9-open vector (HsCD00379273) obtained from PlasmID DF/HCC DNA Resource Core at Harvard Medical School. pSTV2-MFGE8-BirA*FLAG or 3*FLAG vectors were cloned from pLX304-MFGE8-V5. This vector was kindly provided by Prof. Mathieu Laplante, IUCPQ, Québec, Canada. The anti-FLAG M2 Magnetic beads and streptavidin sepharose beads were purchased from SIGMA (M8823) and GE Healthcare (17-5113-01), respectively. Anti-FLAG antibody was purchased from SIGMA (1:5,000; F1804). PMSF and DTT were purchased from Bio Basic INC (PB0425) and protease inhibitor cocktail from SIGMA (P8340, 1:500).

### Dataset used

We used the dataset TCGA BRCA (nationwidechildrens.org_clinical_patient_brca.txt) consisting of 877 patients; 140 TNBC and 737 non-TNBC. TNBC status was determined by the gene expression of ER, PR and HER2. The non-TNBC group consisted of Luminal A (ER+ and/or PR+, HER2−), Luminal B (ER+ and/or PR+, HER2+), and HER2 (ER−, PR−, HER2+). The features space size was 98,026 attributes. For the RNA expression there are two types of information available: Gene expression (about 20,500 genes) and their isoforms (different versions of the genes). We selected the isoforms version (73,599 attributes). The miRNA expression contains 1,046 attributes. For the methylation dataset we built a view called methyl_fusion (23,381 attributes) based on the HumanMethylation450 and HumanMethylation27 BeadChip Kits. The fusion view was built by merging the information available in the previous two techniques. The N/A data in the methylation dataset were replaced by a special mean value: a mean based on the positive examples values and another one based on the negative examples were built. The N/A values were filled based on their labels.

### Machine learning analysis

Three different algorithms were utilized to analyze the TCGA dataset: DT^42^, RF^43^ and SCM^13^. In the supervised ML settings, we assume that the data are available as a set $S\stackrel{\scriptscriptstyle\mathrm{def}}{=}{\{({x}_{i},{y}_{i})\}}_{i=0}^{m}\sim {D}^{m}$ where ${x}_{i}\in X$ is a training example, ${y}_{i}\in Y$ the associated label or phenotype, $D$ is a data generating distribution and *m* the size of the dataset. We focus here on the binary classification problem i.e. $y\in \left\{-1,\left.1\right\}\right.$ or $y\in \left\{0,\left.1\right\}\right.$. The goal of every learning algorithm is to obtain a predictor $h:X\to Y$ such that $h\left(x\right)=y\forall (x,y)\sim D$. Originally introduced by Marchand et al., the SCM is a greedy algorithm whose goal is to learn a conjunction or a disjunction of rules^13^. This is achieved by finding the decision function depending on the smallest number of attributes. Lets $P$ be the subset of positive examples and $N$ be the subset of negative examples i.e $S=P\cup N$. A function $h$ is said to be consistent with an example if it correctly classifies that example. Lets then define ${N}_{h}$ the subset of examples in $N$ for which $h$ are consistent. Lets also define ${\stackrel{-}{P}}_{h}$ the subset of examples in $P$ for which $h$ make mistakes. The SCM utility function provides the predictor $h$ that maximises ${U}_{h}=\left|{N}_{h}\right|-p\times \left|{\stackrel{-}{P}}_{h}\right|$. The SCM has 2 hyper-parameters: the penalty $p$ and the early stopping point $s$. They are the two model-selection parameters that give the user the ability to control the proper trade off between the training accuracy and the size of the function. The SCM performs efficiently in the classification framework problem with focus on interpretable and sparse models. A decision tree is a tree where each node represents a feature (attribute), each link (branch) represents a decision (rule) and each leaf represents an outcome (categorical or continues value). For instability purpose, we also use the RF algorithm which is essentially a bunch of DTs with majority vote.

For the analysis the main matrices used were accuracy, precision and F1-score. These matrices present how well the algorithm performs on the negative and positive example simultaneously. Since the dataset is unbalanced, we focused our attention on the F1-Score, because if the F1-score is high, that means our algorithm is performing well and vice versa

$$ \begin{gathered} {\text{Accuracy}} = \frac{{{\text{TP}} + {\text{TN}}}}{{{\text{TP}} + {\text{TN}} + {\text{FN}} + {\text{FP}}}}\quad {\text{Precision}} = \frac{{{\text{TP}}}}{{{\text{TP}} + {\text{FP}}}} \hfill \\ {\text{Recall}} = \frac{{{\text{TP}}}}{{{\text{TP}} + {\text{FN}}}}\quad {\text{F1}}\;{\text{score}} = {2}*\frac{{{\text{Precision}}*{\text{Recall}}}}{{{\text{Precision}} + {\text{Recall}}}} \hfill \\ \end{gathered} $$

where, TP = true positive, TN = true negative, FN = false negative, FP = false positive.

In order to avoid any chances of randomness, we repeated the learning experiences 100 times and presented the result of all the repeats. From these results, we retrieved the ten best models after the 100 repetitions. In the case of the SCM, it is straight forward since all the models were conjunctions type. For the DTs and the RF, we make the assumptions for the model to consider only the first three features and then do the count with these features. The detailed flow chart of ML analysis is presented in Fig. 1.

### Survival analysis

The ML analysis provided 20 potential genes for which their effect on survival was analyzed in 16 different datasets consisting of 2,164 patients using PREdiction of Clinical Outcomes from Genomic Profiles (Precog). Based on the most pronounced effect on survival outcome, and the maximum time a gene was coming up in ML analysis repeats, three genes were selected: *TBC1D9, SLC16A6* and *MFGE8*. Further, we looked at their effect on survival outcome of BC patients in another dataset using Kaplan Meier plotter (https://kmplot.com/analysis/). The DMFS was analyzed on 1746 patients and PPS was analyzed on 414 patients.

#### Analysis of selected genes differentiating TNBC from non-TNBC

The expression of selected genes were analyzed in TCGA provincial dataset in cBioPortal (https://www.cbioportal.org/). The dataset consisted of 1,101 patients. The difference was analyzed according to their RNA expression (tcga_rnaseqv2-brca-v2.0_gene sample). Furthermore, expression was checked in the original dataset TCGA BRCA to see if they could differentiate TNBC from non-TNBC. A similar analysis was done by validating the mRNA expression level of these genes in patients from the tissue bank of Centre des Maladies du Sein (Hôpital du St-Sacrement, Quebec, Canada). The patient samples consisted of 13 TNBC and 12 non-TNBC based on clinical biomarkers (ER, PR, HER2) measured by immunohistochemistry. The non-TNBC subgroup consisted of four samples of each Luminal A, Luminal B and HER2 subgroups.

### RNA isolation

Total RNA from breast tissue samples were isolated by Qiagen RNeasy Mini Kit (Qiagen, Hilden, Germany). Preparation for whole-genome expression analysis was performed using the SensationPlus™ FFPE Amplification Kit.

### Quantitative real-time PCR (q-PCR) analysis

Quantitative PCR was performed using SyBr Green technology as described previously^44^. Briefly, oligo-primer pairs that allow the amplification of ~ 200 base pairs (bp) of the indicated specific mRNA were designed by GeneTools software and their specificity was verified by blasting the GenBank database. The sequence of primers is indicated in Supplementary Table 9. Second-derivative and double-correction method were used for data calculation and normalization^45^, with three housekeeping genes (*ATP50*, *HPRT1* and *GAPDH*). The mRNA levels were expressed as number of copies/µg of total RNA calculated using corresponding standard curves. The qPCR result was analysed using the Wilcoxon rank sum test.

### Affinity-purification (AP)

HEK293 Flp-In T-REx cells stably expressing tetracycline-inducible pDEST-TBC1D9/MFGE8 3xFLAG were generated as per Lambert et al.^41^. Untagged parental cells were used as controls. These cells were grown in 15 cm plates at 75% confluency, two plates per replicate, induced with 1 µg/µL of tetracycline and incubated at 37 °C for 24 h. After 24 h, cells were washed with fresh 1X PBS, pelleted in 2 ml tubes and lysed with 1.5 ml of AP lysis buffer. AP lysis buffer: 0.1% (v/v) NP-40, 50 mM HEPES–NaOH pH 8.0, 100 mM KCl, 2 mM EDTA, 10% (v/v) Glycerol; supplemented with 1 mM DTT, 1 mM PMSF and 1X protease inhibitor cocktail (SIGMA, P8340) prior utilization. Chromatin shearing was done by sonication (for 30 s at power ~ 4 using Sonic Dismembrator 60 equipped with 1/8″ probe) while storing on ice. Then, 250 units of turbonuclease (SIGMA, T4330) was added and incubated on rotator for 1 h at 4 °C. The samples were centrifuged at 14,000 rpm for 20 min at 4 °C, and the supernatant was collected in a new tube.

#### Anti-FLAG immunoprecipitation

The anti-FLAG M2 Magnetic beads were prepared by washing them three times in cold 1XPBS and used 25 µL/sample. Supernatant containing the proteins from the cells was added to the tubes containing 25 µL of anti-FLAG magnetic beads. The mixture was incubated at 4 °C for 2 h on rotator. The beads were pelleted by centrifugation for 1 min at 1,000 rpm and were placed on an ice-cold magnetic rack to remove the supernatant. The beads were resuspended in 1 mL of cold lysis buffer and transferred to a new 1.5 mL tube. They were then pelleted by centrifugation and washed again with 1 mL of 20 mM Tris–HCl pH 8.0, 2 mM CaCl_2_. After the last wash, a quick centrifugation was done (10 s at 1,000 rpm) and the samples were placed on magnetic rack on ice to remove extra liquid.

### Proximity biotinylation assay (BioID)

Tetracycline-inducible HEK293 Flp-In T-REx cells stably expressing BirA*-FLAG-TBC1D9/MFGE8 fusion protein were grown in 15 cm plates to 75% confluence, two plates per replicate. Negative controls samples for BioID experiments were parental Flp-In T-REx HEK293 stable cells expressing BirA*-FLAG fused either to a green fluorescent protein (GFP), to a nuclear localization sequence (NLS) or by itself as previously done^41^. The cells were treated with 1 µg/µL of tetracycline and 50 µM biotin simultaneously and were incubated at 37 °C for 24 h. After 24 h, cells were washed with fresh 1X PBS and were lysed in 1.5 mL of RIPA lysis Buffer on ice. RIPA lysis buffer: 1% (v/v) NP-40, 0.1% SDS, 50 mM Tris–HCl pH7.4, 150 mM NaCl, 0.5% (w/v) Sodium Deoxycholate, 1 mM EDTA; supplemented with 1 mM DTT, 1 mM PMSF (Bio Basic INC, PB0425) and 1X protease inhibitor cocktail (SIGMA, P8340) prior utilization. Samples were sonicated to shear the chromatin (30 s at power ~ 4 using Sonic Dismembrator 60 equipped with 1/8″ probe) on ice. Further, 250 units of turbonuclease were added and incubated on rotator for 1 h at 4 °C. The sample was centrifuged at 14,000 rpm for 20 min at 4 °C.

#### Streptavidin-based affinity capture of biotinylated proteins

The Streptavidin Sepharose beads were washed twice in 1 mL of lysis buffer (60 µL of slurry per sample). The beads were pelleted by centrifugation after each washing and were then incubated with samples at 4 °C on rotator for 3 h. The bound beads were pelleted by centrifugation and were washed twice with 1 mL of RIPA lysis buffer (without protease inhibitors) and transferred to a new 1.5 mL Eppendorf tube to minimize background contaminants. Tubes were centrifuged and the supernatants were discarded. The beads were washed three times with 1 mL of 50 mM Ammonium bicarbonate (ABC).

### Sample preparation for LC–MS/MS analysis

#### On-beads trypsin digestion

For BioID, the beads were resuspended in 100 µL of 50 mM ABC with 1 µg of trypsin (resuspend in Tris–HCl pH 8.0). The samples were incubated overnight (~ 15 h) at 37 °C with shaking with an extra 1 µg of trypsin added subsequently for 2–4 h to insure complete digestion. Samples were gently centrifuged, and the supernatant transferred in new tubes. The beads were rinsed twice and the supernatant pooled. For AP, the washed bound beads were resuspended in 750 ng of trypsin and were incubated overnight at 37 °C with gentle lateral agitation. After overnight incubation, samples were magnetized, and supernatants were collected in new tubes. Another 250 ng of trypsin was added to the beads and incubated 3–4 h without agitation. The supernatants were then transferred to new tubes.

#### Peptides recovery and desalting

The peptide digestion was stopped by adding formic acid (from a 50% stock solution) to a final concentration of 2%. Samples were dried in a Speed-Vac vacuum concentrator without heat. The samples were desalted with C_18_ Stage Tips and sent for MS processing. The C_18_ StageTips were prepared according to the published protocol by Rappsilber et al.^46^. Conditioning: The disks were made wet by passing 20 µL of 100% methanol through the StageTip. Then, 20 µL of buffer B (0.5% formic acid, 80% acetonitrile in water) was added to the StageTip and centrifuged (3,000 rpm, 30 s). Further, 20 µL of buffer A (0.5% formic acid in water) was added to the StageTip and centrifuged (3,000 rpm, 30 s). The samples prepared for AP were directly loaded to the C_18_-StageTips. For BioID, the dried samples were resuspended in 20 µL of buffer A before loading to the C_18_-StageTips. The tips were centrifuged at 3,000 rpm for 3 min. The C_18_-StageTips were washed twice with 20 µL of buffer A at 3,000 rpm for 3 min each time. The samples were eluted by placing the C_18_-StageTips in a fresh tube and adding 20 µL of buffer B. The tubes were centrifuged at 3,000 rpm for 30 s. The process was repeated three times. The elute was dried using SpeedVac and sent for LC–MS/MS analysis.

### Proteins identification by mass spectrometry

The analyses were performed at the proteomic platform of the Quebec Genomics Center. Peptide samples were separated by online reversed-phase (RP) nanoscale capillary liquid chromatography (nanoLC) and analyzed by electrospray mass spectrometry (ESI MS/MS). The experiments were performed with a Dionex UltiMate 3,000 nanoRSLC chromatography system (Thermo Fisher Scientific) connected to an Orbitrap Fusion mass spectrometer (Thermo Fisher Scientific) equipped with a nanoelectrospray ion source. Peptides were trapped at 20 µL/min in loading solvent (2% acetonitrile, 0.05% TFA) on a 5 mm × 300 µm C_18_ pepmap cartridge pre-column (Thermo Fisher Scientific) for 5 min. Then, the pre-column was switched online with a self-made 50 cm × 75 µm internal diameter separation column packed with ReproSil-Pur C_18_-AQ 3-μm resin (Dr. Maisch HPLC) and the peptides were eluted with a linear gradient from 5 to 40% solvent B (A: 0,1% formic acid, B: 80% acetonitrile, 0.1% formic acid) in 90 min, at 300 nL/min^47^. Mass spectra were acquired using a data dependent acquisition mode using Thermo XCalibur software version 3.0.63. Full scan mass spectra (350–1,800 m/z) were acquired in the orbitrap using an AGC target of 4e5, a maximum injection time of 50 ms and a resolution of 120,000. Internal calibration using lock mass on the m/z 445.12003 siloxane ion was used. Each MS scan was followed by acquisition of fragmentation spectra of the most intense ions for a total cycle time of 3 s (top speed mode). The selected ions were isolated using the quadrupole analyzer in a window of 1.6 m/z and fragmented by Higher energy Collision-induced Dissociation (HCD) with 35% of collision energy. The detection of resulting fragments was done by the linear ion trap with an AGC target of 1E4 in rapid scan rate and a maximum injection time of 50 ms. Dynamic exclusion of previously fragmented peptides was set for a period of 20 s and a tolerance of 10 ppm^48^.

### Data dependent acquisition MS analysis

Mass spectrometry data was stored, searched and analyzed using the ProHits laboratory information management system (LIMS) platform^49^. Thermo Fisher scientific RAW mass spectrometry files were converted to mzML and mzXML using ProteoWizard (3.0.4468)^50^. The mzML and mzXML files were then searched using Mascot (v2.3.02) and Comet (v2012.02 rev.0). The spectra were searched with the RefSeq database (version 57, January 30th, 2013) acquired from NCBI against a total of 72,482 human and adenovirus sequences supplemented with “common contaminants” from the Max Planck Institute (https://141.61.102.106:8080/share.cgi?ssid=0f2gfuB) and the Global Proteome Machine (GPM; https://www.thegpm.org/crap/index.html). Charges + 2, + 3 and + 4 were allowed and the parent mass tolerance was set at 12 ppm while the fragment bin tolerance was set at 0.6 amu. Deamidated asparagine and glutamine and oxidized methionine were allowed as variable modifications. The results from each search engine were analyzed through TPP (the Trans-Proteomic Pipeline (v4.6 OCCUPY rev 3)^51^ via the iProphet pipeline^52^.

### MS data archiving

All MS files used in this study were deposited at MassIVE (https://massive.ucsd.edu) and at ProteomeXchange (https://www.proteomexchange.org/). They were assigned the identifiers MassIVE MSV000084743 and PXD016934 and can be accessed at 10.25345/C5C39C and ftp://MSV000084743@massive.ucsd.edu.

### Statistical analysis of AP-MS and BioID data

The MS data generated by AP or BioID were analyzed using SAINTexpress^53^. For AP-MS data, four uncompressed untagged controls were used while for BioID samples, 24 control samples were compressed to 12. Significant protein interactors were those found to have a Bayesian False Discovery Rate (BFDR) ≤ 0.01. Significant interaction partners were further refined using the Crapome online tool (https://crapome.org/) to remove proteins commonly co-purified in FLAG tag pulldown. For this, a cut-off of 0–20 was enforced (i.e. proteins which have been identified 0–20 times with FLAG-tag out of 411 experiments). The resulting dataset was used for subsequent analysis with Metascape software (https://metascape.org/).

### Ethical approval

All patients provided written informed consent. Ethical approval of the study was obtained from the Research Ethics Committee of the Centre de Recherche du CHU de Québec, Canada.

### Consent for publication

The study was carried out in accordance with the relevant guidelines and regulations.

## Supplementary information

Supplementary information

## Data Availability

All MS files used in this study were deposited at MassIVE (https://massive.ucsd.edu) and at ProteomeXchange (https://www.proteomexchange.org/). They were assigned the identifiers MassIVE MSV000084743 and PXD016934 and can be accessed at 10.25345/C5C39C and ftp://MSV000084743@massive.ucsd.edu. The password to access these files until publication is “TNBC”.
