# Supplementary material for: Machine learning analysis identifies genes differentiating triple negative breast cancers
Source: Sci Rep. 2020 Jun 26;10:10464. doi: 10.1038/s41598-020-67525-1 (PMC7320018; doi:10.1038/s41598-020-67525-1)
Supplement: Supplementary file 1 — Supplementary information [file 41598_2020_67525_MOESM1_ESM.pdf]

Machine learning analysis identifies genes differentiating triple negative breast cancers.

Charu Kothari[1,2], Mazid Abiodoun Osseni[1,3], Lynda Agbo[1,2], Geneviève Ouellette[1,2], Maxime Déraspe[1,3], François Laviolette[3,4], Jacques Corbeil[1,3], Jean-Philippe Lambert[1,2], Caroline Diorio[2,5], Francine Durocher[1,2].

[1]Département de médecine moléculaire, Faculté de médecine, Université Laval. Québec City, QC, Canada.

[2]Centre de recherche sur le cancer, Centre de recherche du CHU de Québec-Université Laval, Québec City, QC, Canada.

[3]Big Data Research Centre, CHU de Québec-Université Laval, Quebec City, QC, Canada.

[4] Département d'informatique et de génie logiciel, Faculté des sciences et de génie, Université Laval, Québec City, QC, Canada

[5]Département de médecine sociale et préventive, Faculté de médecine, Université Laval, Québec City, QC, Canada.

*Correspondence: Francine Durocher

Centre de recherche du CHU de Québec-Université Laval

2705 Laurier Blvd, Bloc R4778

Québec (Québec) G1V4G2

Francine.Durocher@crchudequebec.ulaval.ca

Phone 418 525-4444, ext. 48508

Fax 418 654-2278

A.

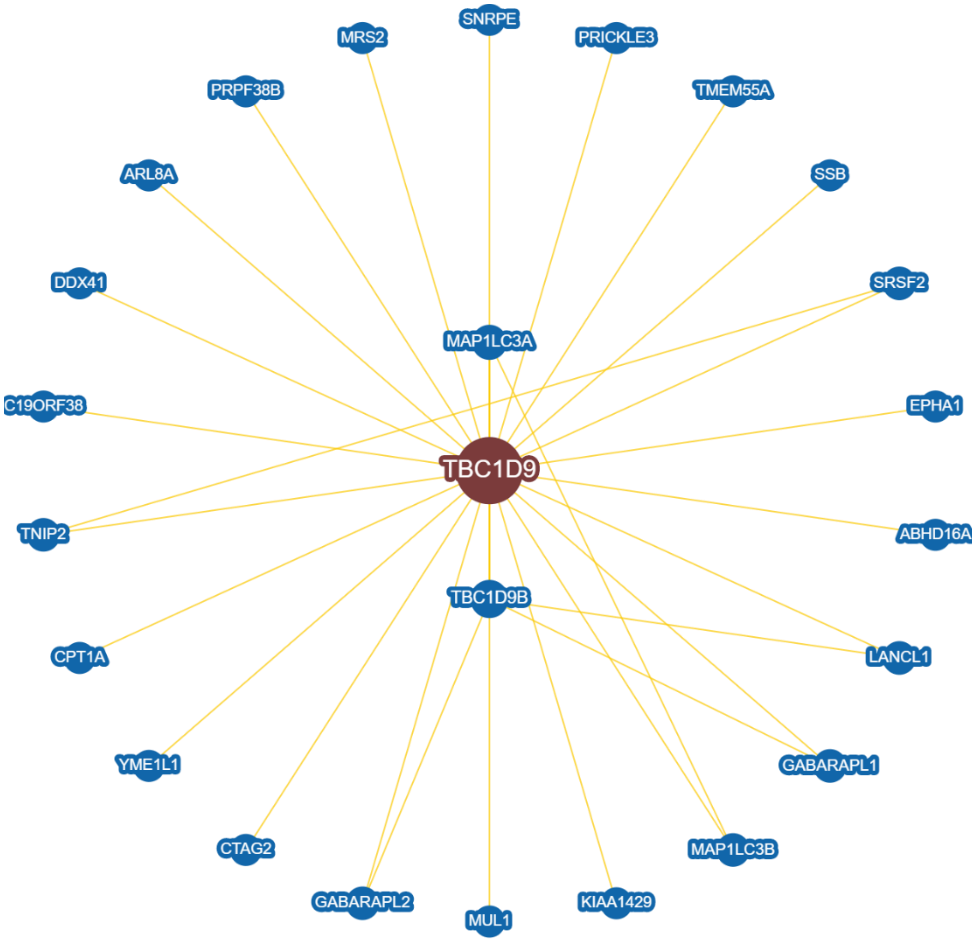

B.

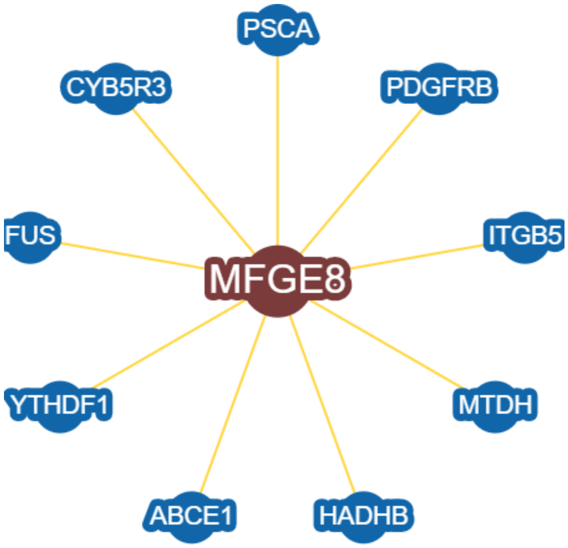

Supplementary Figure 1: Biogrid network. (A) TBC1D9 (B) MFGE8

Supplementary Table 1: AP-MS data for TBC1D9 after SAINT analysis

AP-MS data for TBC1D9-3xFLAG in Flp-In T-REx HEK293 cells. Preys with BFDR ≤ 1% are considered high-confidence and shown in Green here. Bait is 3xFLAG tagged TBC1D9 protein. Prey Gene is the Official Gene Symbol (from NCBI). Spectral counts for the prey (Spec, separated by "I" delimiter), Averaged probability across replicates (AvgSpec), Fold Change (counts in the purification divided by counts in the controls plus small factor to prevent division by 0) and Bayesian FDR (BFDR) are listed for each bait-prey relationship and are directly from the SAINTexpress output. 4 uncompressed untagged controls were employed in the SAINT analysis. The experiment was repeated twice.

PreyGene	Spec	AvgP	FoldChange	BFDR
HMGXB4	5|6	1	55	0
KNOP1	3|3	0.98	30	0
ACOT9	12|13	1	8.33	0
PPOX	4|3	0.99	35	0
ANK2	4|3	0.99	35	0
SMARCA4	5|4	1	45	0
COMT	3|3	0.98	30	0
TBL1X	6|6	1	60	0
ZMYM2	3|4	0.99	35	0
PLRG1	4|3	0.99	35	0
TRRAP	7|6	1	65	0
EP300	6|5	1	55	0
PHB	3|3	0.98	30	0
EXOSC10	4|4	1	40	0
RAB13	4|3	0.99	35	0
CBX4	4|4	1	40	0
TRIM24	4|6	1	50	0
RAB5A	4|4	1	40	0
PCYT1A	5|3	0.99	40	0
PLK1	16|15	1	155	0
TLE1	7|6	1	65	0
PGRMC2	3|3	0.98	30	0
PVR	4|4	1	40	0
ILVBL	16|18	1	22.67	0
FAM162A	3|4	0.99	35	0
VWA8	19|20	1	3.71	0
SMCHD1	12|12	1	120	0
UFL1	4|4	1	40	0
NOC2L	3|3	0.98	30	0
NUSAP1	4|5	1	45	0
RBBP6	3|3	0.98	30	0
APMAP	3|3	0.98	30	0
RDH14	4|3	0.99	35	0

SQOR	6|4	1	50	0
TMEM43	3|4	0.99	35	0
RIOX1	5|7	1	60	0
CLPTM1L	6|5	1	55	0
GPRIN1	3|4	0.99	35	0
ARL8A	16|14	1	30	0
TAF6	4|5	1	45	0
DNAJC19	3|3	0.98	30	0
RETREG3	4|3	0.99	35	0
RBM12B	3|3	0.98	30	0
TPP1	4|2	0.95	30	0.01
GSTK1	2|3	0.95	25	0.01
GBA	3|2	0.95	25	0.01
CCDC51	3|2	0.95	25	0.01
OS9	3|2	0.95	25	0.01
SGPL1	3|2	0.95	25	0.01
KATNB1	3|2	0.95	25	0.01
SLC25A17	3|2	0.95	25	0.01
CELF2	4|2	0.95	30	0.01
PREB	4|2	0.95	30	0.01
CCDC59	2|3	0.95	25	0.01
SUGP2	2|4	0.95	30	0.01
LSG1	2|3	0.95	25	0.01
YLPM1	11|10	0.97	14	0.01
COQ9	2|3	0.95	25	0.01
PYCR3	3|2	0.95	25	0.01
PGS1	3|2	0.95	25	0.01
PTGES2	11|9	0.97	8	0.01
ADPGK	2|4	0.95	30	0.01
LONP2	3|2	0.95	25	0.01
RHOT2	3|2	0.95	25	0.01
ANGEL2	2|3	0.95	25	0.01
ELMOD2	4|2	0.95	30	0.01
GGT7	3|2	0.95	25	0.01
MBLAC2	2|4	0.95	30	0.01
GGCX	2|2	0.91	20	0.02
CPT1A	2|2	0.91	20	0.02
NECTIN2	2|2	0.91	20	0.02
UNK	2|2	0.91	20	0.02
ZNF207	2|2	0.91	20	0.02
RTCA	2|2	0.91	20	0.02
TBC1D5	2|2	0.91	20	0.02
NKRF	7|6	0.93	6.5	0.02
AMMECR1L	2|2	0.91	20	0.02
UBE4A	2|2	0.91	20	0.02

MAD1L1	12|11	0.94	3.54	0.02
SMARCA5	2|2	0.91	20	0.02
DCAF5	2|2	0.91	20	0.02
TARBP2	2|2	0.91	20	0.02
DVL2	2|2	0.91	20	0.02
OXA1L	2|2	0.91	20	0.02
PPIL1	2|2	0.91	20	0.02
ARL8B	17|16	0.92	6.6	0.02
BRK1	2|2	0.91	20	0.02
TMX4	2|2	0.91	20	0.02
SLC25A19	2|2	0.91	20	0.02
FAR1	2|2	0.91	20	0.02
AGPAT1	2|2	0.91	20	0.02
PPFIBP1	2|2	0.91	20	0.02
CHMP2A	2|2	0.91	20	0.02
CPT2	8|9	0.9	4.25	0.04
GPD2	10|8	0.87	6	0.04
SIN3A	21|15	0.86	4.5	0.04
IDH2	5|5	0.87	10	0.04
PTP4A1	5|5	0.87	10	0.04
SLC25A11	7|6	0.84	8.67	0.04
PRPF3	8|8	0.9	6.4	0.04
PLXNB2	12|12	0.87	6	0.04
DCP1A	5|5	0.87	10	0.04
DNAJC1	4|6	0.85	10	0.04
UPF3B	11|11	0.84	3.38	0.04
DCAKD	7|6	0.84	8.67	0.04
TARS2	6|4	0.85	10	0.04
MIA2	4|4	0.76	8	0.05
BCAS2	4|4	0.76	8	0.05
PHF3	4|4	0.76	8	0.05
THEM6	4|4	0.76	8	0.05
CLGN	11|10	0.73	4.67	0.06
SLC25A4	15|13	0.74	5.09	0.06
SLC27A2	5|5	0.71	6.67	0.06
HAX1	5|5	0.73	5	0.06
NUP58	3|5	0.73	8	0.06
DHX35	5|3	0.73	8	0.06
HDHD5	6|7	0.75	5.2	0.06
TECR	5|3	0.73	8	0.06
SPTLC2	6|4	0.7	5	0.07
SPTLC1	7|7	0.7	4.67	0.07
PDCD4	6|4	0.7	5	0.07
USP34	6|4	0.69	6.67	0.07
ZDBF2	6|6	0.68	6	0.07

MOSPD2	10|10	0.68	3.64	0.07
CDH2	3|4	0.67	7	0.08
CHP1	4|3	0.67	7	0.08
ZNF318	3|4	0.67	7	0.08
SYNE2	11|13	0.68	4.36	0.08
NDC1	9|7	0.68	4.57	0.08
TMA16	4|3	0.67	7	0.08
VARS2	3|4	0.67	7	0.08
PES1	5|4	0.62	4.5	0.09
TOR1AIP1	2|7	0.66	9	0.09
VAPB	4|5	0.62	4.5	0.09
DIMT1	5|4	0.62	4.5	0.09
SCCPDH	4|5	0.65	6	0.09
AARS2	6|8	0.64	4.67	0.09
NELFCD	9|4	0.63	5.2	0.09
PHB2	7|6	0.59	4.33	0.1
PRKAR2B	6|3	0.62	6	0.1
SALL2	3|3	0.58	6	0.1
SLC25A1	8|8	0.58	3.2	0.1
RAB35	4|4	0.58	5.33	0.1
GNL3	6|5	0.6	4.4	0.1
AKAP8L	3|3	0.58	6	0.1
AGK	4|4	0.58	5.33	0.1
NAT10	3|3	0.58	6	0.1
HMOX2	6|3	0.57	4.5	0.11
GATAD2A	5|3	0.57	5.33	0.11
TRMT1L	6|3	0.57	4.5	0.11
HADH	4|2	0.56	6	0.12
ZSWIM8	5|8	0.56	4.33	0.12
PMPCA	4|2	0.56	6	0.12
ALDH1B1	4|5	0.53	4.5	0.13
BCAP31	5|4	0.53	4.5	0.13
ACSL3	18|13	0.54	2.95	0.13
PRDX4	5|7	0.52	4	0.13
TMED10	6|6	0.56	4	0.13
TMX1	16|12	0.55	2.95	0.13
ABCB7	6|11	0.5	3.09	0.14
PARG	4|3	0.51	4.67	0.14
NOP14	4|4	0.5	4	0.14
SART1	4|6	0.5	4	0.14
DDOST	5|5	0.52	4	0.14
RNF113A	3|4	0.51	4.67	0.14
FAM169A	6|10	0.51	4	0.14
EIF5AL1	0|7	0.5	35	0.15
RGPD8	0|19	0.5	95	0.15

RAB5B	0|5	0.5	25	0.15
FLNC	9|0	0.5	45	0.15
STRBP	6|0	0.5	30	0.15
GPC4	0|4	0.5	20	0.16
ACSL1	4|0	0.5	20	0.16
RER1	4|0	0.5	20	0.16
FAF2	0|4	0.5	20	0.16
PCF11	4|0	0.5	20	0.16
IGSF3	0|3	0.49	15	0.17
OCIAD1	3|0	0.49	15	0.17
HRAS	0|3	0.49	15	0.17
TRA2B	3|0	0.49	15	0.17
TMEM161A	0|3	0.49	15	0.17
GNA11	3|0	0.49	15	0.17
MTX1	3|0	0.49	15	0.17
PTK7	0|3	0.49	15	0.17
FLOT2	3|0	0.49	15	0.17
ENPP1	0|3	0.49	15	0.17
TAF5	3|0	0.49	15	0.17
RRP12	3|0	0.49	15	0.17
BCL2L13	0|3	0.49	15	0.17
ARID4B	0|3	0.49	15	0.17
ARID1B	3|0	0.49	15	0.17
NSMCE4A	0|3	0.49	15	0.17
NAT14	0|3	0.49	15	0.17
SMC6	3|0	0.49	15	0.17
TSC22D4	0|3	0.49	15	0.17
CDK13	0|3	0.49	15	0.17
YME1L1	3|0	0.49	15	0.17
TRIM11	3|0	0.49	15	0.17
CDK5RAP3	3|0	0.49	15	0.17
GPAT4	3|0	0.49	15	0.17
FMNL3	3|0	0.49	15	0.17
HEXA	2|5	0.48	4.67	0.21
ORC4	5|3	0.49	4	0.21
SNRPA	14|11	0.48	3.33	0.21
PTPMT1	3|5	0.49	4	0.21
DLD	3|2	0.47	5	0.22
IMMT	7|3	0.48	4	0.22
PHF8	2|3	0.47	5	0.22
SCARB2	2|3	0.47	5	0.22
LSR	3|2	0.47	5	0.22
EIF4G3	6|5	0.48	3.67	0.22
XRCC1	3|2	0.47	5	0.22
TBL3	3|2	0.47	5	0.22

UPF2	2|3	0.47	5	0.22
TMEM33	2|3	0.47	5	0.22
CTBP2	3|2	0.47	5	0.22
TOR1A	2|0	0.45	10	0.23
GCDH	0|2	0.45	10	0.23
PON2	0|2	0.45	10	0.23
ERCC2	0|2	0.45	10	0.23
SLX4IP	0|2	0.45	10	0.23
COA3	0|2	0.45	10	0.23
EFL1	2|0	0.45	10	0.23
MIER1	0|2	0.45	10	0.23
RBFOX2	0|2	0.45	10	0.23
FBRS	2|0	0.45	10	0.23
NUDT19	0|2	0.45	10	0.23
LYN	0|2	0.45	10	0.23
KCTD15	2|0	0.45	10	0.23
CDC7	0|2	0.45	10	0.23
MFAP3	2|0	0.45	10	0.23
PPP1R21	0|2	0.45	10	0.23
ZNF507	2|0	0.45	10	0.23
SIRT1	0|2	0.45	10	0.23
FLYWCH2	2|0	0.45	10	0.23
C17orf49	2|0	0.45	10	0.23
MPP1	0|2	0.45	10	0.23
NRCAM	2|0	0.45	10	0.23
HSDL2	2|0	0.45	10	0.23
ERAP1	2|0	0.45	10	0.23
DTNA	2|0	0.45	10	0.23
GALC	0|2	0.45	10	0.23
NR3C1	0|2	0.45	10	0.23
ABHD10	2|0	0.45	10	0.23
CAMLG	2|0	0.45	10	0.23
SUMO1	2|0	0.45	10	0.23
NRP1	2|0	0.45	10	0.23
PKMYT1	2|0	0.45	10	0.23
DVL3	2|0	0.45	10	0.23
PCK2	0|2	0.45	10	0.23
PRPF4	0|2	0.45	10	0.23
ARHGEF6	2|0	0.45	10	0.23
ATP6V1G1	0|2	0.45	10	0.23
THOC1	2|0	0.45	10	0.23
ERCC4	2|0	0.45	10	0.23
RALA	0|2	0.45	10	0.23
GTF2E1	2|0	0.45	10	0.23
LNPEP	2|0	0.45	10	0.23

ERAL1	0|2	0.45	10	0.23
TSNAX	2|0	0.45	10	0.23
PRKAB1	2|0	0.45	10	0.23
DNAJC3	2|0	0.45	10	0.23
SSSCA1	2|0	0.45	10	0.23
DRAP1	2|0	0.45	10	0.23
P3H4	0|2	0.45	10	0.23
CPSF4	0|2	0.45	10	0.23
BRAP	2|0	0.45	10	0.23
SEC63	0|2	0.45	10	0.23
PGLS	0|2	0.45	10	0.23
SEPHS1	0|2	0.45	10	0.23
KIN	0|2	0.45	10	0.23
ACAD9	0|2	0.45	10	0.23
HELZ	0|2	0.45	10	0.23
MORC2	0|2	0.45	10	0.23
EXOSC7	2|0	0.45	10	0.23
COG4	0|2	0.45	10	0.23
DHRS7B	2|0	0.45	10	0.23
OSBPL3	2|0	0.45	10	0.23
DYNC2LI1	0|2	0.45	10	0.23
VPS36	2|0	0.45	10	0.23
COMMD2	2|0	0.45	10	0.23
SMN2	0|2	0.45	10	0.23
TMED9	0|2	0.45	10	0.23
SLC35F2	2|0	0.45	10	0.23
RNF31	2|0	0.45	10	0.23
SDAD1	2|0	0.45	10	0.23
RHOT1	2|0	0.45	10	0.23
ALG1	2|0	0.45	10	0.23
CACHD1	0|2	0.45	10	0.23
METTL14	2|0	0.45	10	0.23
VIPAS39	2|0	0.45	10	0.23
ERAP2	2|0	0.45	10	0.23
RMND5A	0|2	0.45	10	0.23
RBM42	2|0	0.45	10	0.23
SLC27A3	2|0	0.45	10	0.23
HAUS3	0|2	0.45	10	0.23
RNASEH2B	2|0	0.45	10	0.23
PLXNA2	0|2	0.45	10	0.23
ILKAP	2|0	0.45	10	0.23
HSDL1	0|2	0.45	10	0.23
AGAP3	2|0	0.45	10	0.23
PLXNA1	2|0	0.45	10	0.23
COQ5	0|2	0.45	10	0.23

GHDC	0|2	0.45	10	0.23
SSBP4	0|2	0.45	10	0.23
TMEM209	2|0	0.45	10	0.23
BRD4	6|0	0.47	6	0.23
PKHD1	0|2	0.45	10	0.23
SLC39A11	0|2	0.45	10	0.23
MRPL10	0|2	0.45	10	0.23
SNX5	2|0	0.45	10	0.23
CCDC110	0|2	0.45	10	0.23
LPCAT4	0|2	0.45	10	0.23
C3orf58	2|0	0.45	10	0.23
CYP4X1	0|2	0.45	10	0.23
SASS6	2|0	0.45	10	0.23
RPS19BP1	2|0	0.45	10	0.23
PHC2	2|0	0.45	10	0.23
VDAC3	5|4	0.45	3.6	0.32
NCOR1	4|5	0.45	3.6	0.32
ARID2	4|5	0.45	3.6	0.32
RGPD1	18|15	0.41	3.47	0.33
EXOC3	3|3	0.43	4	0.33
UBQLN1	3|3	0.43	4	0.33
SACM1L	5|4	0.42	3.6	0.33
C2CD5	2|4	0.42	4	0.33
RRP1B	5|6	0.41	3.14	0.33
SMC5	5|4	0.42	3.6	0.33
PPIP5K2	5|4	0.42	3.6	0.33
PCYOX1L	4|3	0.4	3.5	0.33
NOC4L	9|11	0.44	2.86	0.33
ZFP91	5|4	0.42	3.6	0.33
CDC42EP1	3|3	0.43	4	0.33
CDC16	2|2	0.36	4	0.34
GXYLT1	5|5	0.36	3.33	0.34
ATL2	2|2	0.36	4	0.34
GRAMD1A	2|2	0.36	4	0.34
ECI1	2|2	0.36	4	0.34
RAB2A	6|4	0.36	3.33	0.34
RAB6A	6|6	0.37	3	0.34
SLC7A1	2|2	0.36	4	0.34
SLC7A5	2|2	0.36	4	0.34
NDUFA7	2|2	0.36	4	0.34
TUBA1B	0|43	0.38	2.15	0.34
LSM6	2|2	0.36	4	0.34
TBC1D2B	2|2	0.36	4	0.34
ADNP	3|4	0.38	3.5	0.34
PI4K2A	4|0	0.38	4	0.34

SRSF10	2|2	0.36	4	0.34
WAPL	9|3	0.34	2.18	0.35
FAM114A2	3|2	0.34	3.33	0.35
OSBPL8	2|3	0.34	3.33	0.35
BCL7A	3|2	0.34	3.33	0.35
DDI2	2|3	0.34	3.33	0.35
DIDO1	12|8	0.35	2.5	0.35
CCNT2	0|5	0.35	3.33	0.35
ADD3	10|9	0.3	2.71	0.36
CHAMP1	12|15	0.31	2.57	0.36
CRMP1	6|3	0.31	3	0.36
KIFC1	4|4	0.31	3.2	0.36
STIM1	9|6	0.3	2.73	0.36
SUCLG1	4|4	0.31	3.2	0.36
PRCC	3|6	0.3	3	0.36
TBRG4	7|7	0.3	2.8	0.36
PML	7|3	0.33	2.5	0.36
GLB1	0|3	0.29	3	0.37
CCNK	3|0	0.29	3	0.37
PARN	3|0	0.29	3	0.37
RNF213	0|3	0.29	3	0.37
COL6A1	3|0	0.29	3	0.37
CDC23	3|0	0.29	3	0.37
ALDH6A1	3|0	0.29	3	0.37
AAAS	7|7	0.29	2.8	0.37
NBEA	4|0	0.29	2.67	0.37
MRPS11	0|3	0.29	3	0.37
ARPC5L	3|0	0.29	3	0.37
ZNF503	3|0	0.29	3	0.37
ASS1	3|0	0.29	3	0.37
NIPBL	0|3	0.29	3	0.37
CSNK1E	0|3	0.29	3	0.37
UGDH	9|6	0.26	2.73	0.38
CUL5	2|2	0.25	2.67	0.38
RRP1	3|3	0.25	3	0.38
ATP5MG	2|2	0.25	2.67	0.38
SEC61B	2|2	0.25	2.67	0.38
MORC3	6|4	0.25	2.86	0.38
UBXN7	6|5	0.25	2.75	0.38
NOP16	3|3	0.25	3	0.38
IWS1	6|5	0.25	2.75	0.38
SSBP3	2|2	0.25	2.67	0.38
UBB	5|4	0.28	3	0.38
3-Sep	5|3	0.25	2.67	0.38
CERS2	4|0	0.25	2	0.38

FAM192A	3|3	0.25	3	0.38
KDELC2	5|4	0.28	3	0.38
MTDH	2|2	0.25	2.67	0.38
CPOX	0|3	0.22	2	0.39
ZNF629	3|0	0.22	2	0.39
ZSCAN18	0|3	0.22	2	0.39
SDHB	3|0	0.22	2	0.39
PPIH	3|0	0.22	2	0.39
PACSIN2	8|6	0.22	2.8	0.39
HARS2	0|3	0.22	2	0.39
RAI14	4|0	0.24	2	0.39
GTSE1	4|3	0.23	2.8	0.39
COQ8A	7|3	0.23	2.22	0.39
INTS3	2|3	0.24	2.5	0.39
NOTCH2	5|3	0.22	2.67	0.39
CDC73	5|3	0.22	2.67	0.39
NOMO2	0|8	0.25	1.78	0.39
PLOD2	6|3	0.22	2.57	0.39
C3	3|3	0.21	2.4	0.4
TXN2	4|4	0.19	2.67	0.4
RTN1	4|6	0.2	2.5	0.4
COQ8B	4|4	0.19	2.67	0.4
VMP1	4|4	0.19	2.67	0.4
CTSB	2|5	0.19	2.33	0.4
POR	0|2	0.18	2	0.41
CELF1	4|2	0.18	2.4	0.41
ORC3	4|2	0.18	2.4	0.41
CNOT9	2|0	0.18	2	0.41
MSH4	2|0	0.18	2	0.41
PTPRG	0|2	0.18	2	0.41
SCP2	2|0	0.18	2	0.41
SMARCB1	0|2	0.18	2	0.41
CAMK1	2|0	0.18	2	0.41
TIMELESS	2|0	0.18	2	0.41
HIP1R	0|2	0.18	2	0.41
SELENOF	2|0	0.18	2	0.41
RAC3	2|0	0.18	2	0.41
VPS26C	0|2	0.18	2	0.41
MICU1	2|0	0.18	2	0.41
STXBP3	0|2	0.18	2	0.41
PALD1	2|0	0.18	2	0.41
SUPT7L	0|2	0.18	2	0.41
CNOT10	2|0	0.18	2	0.41
THUMPD3	2|0	0.18	2	0.41
UBXN1	4|2	0.18	2.4	0.41

RDH11	0|2	0.18	2	0.41
MPP6	0|2	0.18	2	0.41
DCAF16	0|2	0.18	2	0.41
OSGEP	2|0	0.18	2	0.41
HEATR1	2|0	0.18	2	0.41
RBM28	2|0	0.18	2	0.41
LARP1B	4|2	0.18	2.4	0.41
PUS7	2|0	0.18	2	0.41
RALGAPB	0|2	0.18	2	0.41
PHACTR4	5|4	0.18	2.57	0.41
NDUFS7	2|0	0.18	2	0.41
IRF2BPL	2|0	0.18	2	0.41
CBLL1	2|0	0.18	2	0.41
ZNF703	0|2	0.18	2	0.41
RAMAC	2|0	0.18	2	0.41
ATRX	0|5	0.18	1.67	0.41
PPA2	0|2	0.18	2	0.41
VIRMA	12|11	0.18	2.56	0.41
UQCC1	0|2	0.18	2	0.41
PRKAA1	2|0	0.18	2	0.41
CASK	6|7	0.16	2.6	0.44
LSS	2|3	0.17	2.5	0.44
SHOC2	3|2	0.17	2.5	0.44
DENR	2|3	0.17	2.5	0.44
ARHGEF2	0|3	0.16	1.5	0.44
SDF2	3|2	0.17	2.5	0.44
RAB21	2|3	0.17	2.5	0.44
CKMT1B	4|0	0.16	1.6	0.44
SPNS1	2|3	0.17	2.5	0.44
RNASEH2C	3|2	0.17	2.5	0.44
PRC1	3|2	0.17	2.5	0.44
RNF214	3|0	0.16	1.5	0.44
RTL8C	3|5	0.14	2.29	0.45
ZBTB21	5|5	0.14	2.5	0.45
MAPK3	2|0	0.12	1.33	0.45
SLC39A10	4|3	0.13	2.33	0.45
SMARCAD1	2|0	0.12	1.33	0.45
ALG5	0|2	0.12	1.33	0.45
VDAC2	6|5	0.13	2.44	0.45
GMDS	2|0	0.12	1.33	0.45
USP39	0|2	0.12	1.33	0.45
CALM2	2|2	0.15	2	0.45
CAPNS1	2|0	0.12	1.33	0.45
NFX1	0|2	0.12	1.33	0.45
S100A10	2|0	0.12	1.33	0.45

SLC16A1	3|6	0.15	2.25	0.45
ASH2L	4|3	0.13	2.33	0.45
LAGE3	2|0	0.12	1.33	0.45
CFDP1	2|2	0.15	2	0.45
PDIA5	6|8	0.14	2.55	0.45
FHOD1	4|3	0.14	2.33	0.45
RABGEF1	2|0	0.12	1.33	0.45
MED15	0|2	0.12	1.33	0.45
WDR13	2|0	0.12	1.33	0.45
NCLN	3|4	0.13	2.33	0.45
SNTB1	4|3	0.13	2.33	0.45
CCNB1	2|0	0.12	1.33	0.45
PHACTR2	0|3	0.12	1.5	0.46
SLC25A13	3|0	0.12	1.5	0.46
BCL2L2-PABPN1	3|0	0.12	1.5	0.46
PPP1R8	3|0	0.12	1.5	0.46
TKFC	0|3	0.12	1.5	0.46
CDK16	3|0	0.12	1.5	0.46
STX12	3|0	0.12	1.5	0.46
ORC5	3|0	0.12	1.5	0.46
PUS1	6|6	0.1	2.4	0.47
TXNDC5	2|4	0.11	2	0.47
GRB2	3|6	0.1	2	0.47
MKI67	3|7	0.11	2	0.47
ORC2	6|2	0.1	1.78	0.47
SCAF8	8|11	0.1	2.24	0.47
PDS5B	5|7	0.12	2.4	0.47
MTREX	8|6	0.1	2.33	0.47
ESF1	4|5	0.1	2.25	0.47
NAA25	4|0	0.09	1.33	0.47
RAB2B	4|0	0.09	1.33	0.47
TOR1AIP2	4|4	0.12	2.29	0.47
CYB5A	4|3	0.12	2.33	0.47
POLR1C	5|4	0.1	2.25	0.47
APOA1	2|2	0.08	2	0.48
HEXB	4|5	0.09	2.25	0.48
TP53	2|2	0.08	2	0.48
HP	2|2	0.08	2	0.48
PPHLN1	2|2	0.08	2	0.48
MAVS	2|3	0.09	2	0.48
FHL1	2|2	0.08	2	0.48
SNX4	2|2	0.08	2	0.48
WASF1	2|2	0.08	2	0.48
SHMT1	3|2	0.09	2	0.48
MOGS	16|16	0.09	2.46	0.48

RNASEH2A	3|2	0.09	2	0.48
SNTB2	2|2	0.08	2	0.48
LSM3	2|2	0.08	2	0.48
SMG1	2|2	0.08	2	0.48
FAM126A	2|3	0.09	2	0.48
SNX18	2|2	0.08	2	0.48
OPA1	19|21	0.09	2.42	0.48
UBE2E2	2|2	0.08	2	0.48
NDUFAF3	3|2	0.09	2	0.48
AURKB	3|5	0.07	2	0.49
GNAI2	3|4	0.07	2	0.49
SCO1	3|4	0.07	2	0.49
ABI2	7|6	0.08	2.36	0.49
PITPNB	3|4	0.07	2	0.49
CPSF1	3|5	0.08	2	0.49
NCAPG2	3|4	0.07	2	0.49
TRAPPC11	5|3	0.08	2	0.49
SFXN1	3|5	0.08	2	0.49
MRPS26	3|4	0.07	2	0.49
H2AFY	3|5	0.08	2	0.49
MAP7D2	4|3	0.07	2	0.49
IQGAP3	4|3	0.07	2	0.49
RACGAP1	3|3	0.06	2	0.5
EPHX1	5|2	0.07	1.75	0.5
DAXX	3|0	0.07	1.2	0.5
NFKB1	3|0	0.07	1.2	0.5
MLLT10	2|4	0.06	1.71	0.5
CDC5L	7|10	0.07	2.12	0.5
IRAK1	3|0	0.07	1.2	0.5
HLA-A	8|7	0.06	2.31	0.5
PIK3C3	0|3	0.07	1.2	0.5
TRIM25	5|6	0.06	2.2	0.5
DNAJA3	4|2	0.06	1.71	0.5
RAB8A	5|6	0.07	2.2	0.5
FAF1	5|2	0.07	1.75	0.5
VPS13A	0|3	0.07	1.2	0.5
HDGFL3	6|5	0.07	2.2	0.5
FASTKD5	3|0	0.07	1.2	0.5
CRBN	2|3	0.05	1.67	0.51
NFS1	4|2	0.05	1.71	0.51
GTF3C1	0|4	0.05	1.14	0.51
INPPL1	6|3	0.05	1.8	0.51
POLR3D	2|3	0.05	1.67	0.51
IDH3G	6|6	0.05	2.18	0.51
HMGN1	4|4	0.05	2	0.51

TSG101	3|2	0.05	1.67	0.51
QKI	2|3	0.05	1.67	0.51
MRM2	3|2	0.05	1.67	0.51
PDE4DIP	4|0	0.05	1.14	0.51
CSTF2T	7|6	0.05	2.17	0.51
PCYOX1	7|6	0.05	2.17	0.51
ZCCHC8	3|2	0.05	1.67	0.51
PRPF40A	6|4	0.05	2	0.51
TM9SF3	3|2	0.05	1.67	0.51
CCDC47	3|2	0.05	1.67	0.51
SPC25	3|2	0.05	1.67	0.51
LSM2	2|3	0.05	1.67	0.51
HEATR6	3|2	0.05	1.67	0.51
NCKIPSD	3|2	0.05	1.67	0.51
MPRIP	0|4	0.05	1.14	0.51
ARFIP1	2|2	0.04	1.6	0.52
NCBP2	2|0	0.04	1	0.52
COMMD5	2|2	0.04	1.6	0.52
SRP9	4|4	0.04	2	0.52
CCDC85C	0|2	0.04	1	0.52
GTF3C3	2|2	0.04	1.6	0.52
NIPSNAP2	0|2	0.04	1	0.52
MRPL58	0|2	0.04	1	0.52
ARL2	2|2	0.04	1.6	0.52
GNB1	5|4	0.04	2	0.52
GNS	0|2	0.04	1	0.52
DFFA	4|4	0.04	2	0.52
GOLGA2	0|2	0.04	1	0.52
LMAN1	2|2	0.04	1.6	0.52
SLC1A5	0|2	0.04	1	0.52
SCML1	0|2	0.04	1	0.52
COMMD9	2|0	0.04	1	0.52
USP22	2|2	0.04	1.6	0.52
SLC39A14	2|2	0.04	1.6	0.52
DBR1	4|4	0.04	2	0.52
CDKN2AIP	4|4	0.04	2	0.52
DENND4C	2|2	0.04	1.6	0.52
RBM22	2|2	0.04	1.6	0.52
DLG3	0|2	0.04	1	0.52
MAP1LC3B	2|0	0.04	1	0.52
DCUN1D5	2|2	0.04	1.6	0.52
FAM207A	0|2	0.04	1	0.52
ERC1	0|2	0.04	1	0.52
FAM45A	2|2	0.04	1.6	0.52
MUT	5|3	0.04	1.78	0.54

TTC7B	4|3	0.04	1.75	0.54
RAB12	3|2	0.04	1.67	0.54
EIF4E	3|2	0.04	1.67	0.54
NEDD1	3|2	0.04	1.67	0.54
RCOR3	4|3	0.04	1.75	0.54
CAMK2G	8|9	0.04	2.27	0.54
ARAF	0|3	0.04	1	0.54
MEAF6	2|3	0.04	1.67	0.54
SP1	3|0	0.04	1	0.54
TFRC	2|3	0.04	1.67	0.54
HLA-B	5|3	0.04	1.78	0.54
SCAMP2	3|3	0.04	1.71	0.54
ANKS1A	5|3	0.04	1.78	0.54
CCDC93	7|4	0.04	1.83	0.54
METTL3	3|3	0.04	1.71	0.54
MCCC1	14|10	0.04	2.29	0.54
DTNBP1	0|3	0.04	1	0.54
GLYR1	0|3	0.04	1	0.54
PPP2R5D	5|3	0.04	1.78	0.54
SLC3A2	7|8	0.03	2.14	0.55
RPS29	5|0	0.03	1.11	0.55
CYB5R3	10|10	0.03	2.22	0.55
MYO9B	4|3	0.03	1.75	0.55
IKBKB	7|3	0.03	1.67	0.55
RBM15	3|2	0.03	1.43	0.55
SRPRA	8|12	0.03	2	0.55
DCLK1	4|3	0.03	1.75	0.55
CHAF1B	3|0	0.03	1	0.55
POLE	7|3	0.03	1.67	0.55
TACC1	4|3	0.03	1.75	0.55
AFG3L2	8|6	0.03	2	0.55
PDCD10	3|0	0.03	1	0.55
UBXN4	4|3	0.03	1.75	0.55
CMTR1	8|4	0.03	1.71	0.55
PINX1	3|3	0.03	1.71	0.55
PRPF39	4|0	0.03	1	0.55
PANK4	5|2	0.03	1.56	0.55
PHF10	3|3	0.03	1.71	0.55
ESYT2	5|4	0.03	1.8	0.55
NDUFV3	8|5	0.03	1.86	0.55
NUP210	0|4	0.03	1	0.55
PAPOLA	5|0	0.03	1.11	0.55
QSOX2	6|5	0.03	2	0.55
PM20D2	2|0	0.02	0.8	0.56
TP53BP2	0|4	0.02	1	0.56

AHCYL2	2|0	0.02	0.8	0.56
CYP51A1	2|0	0.02	0.8	0.56
LEMD3	4|2	0.02	1.5	0.56
PEX19	2|0	0.02	0.8	0.56
PRKAG1	0|2	0.02	0.8	0.56
POLR2G	2|0	0.02	0.8	0.56
EIPR1	3|2	0.02	1.43	0.56
NIPSNAP1	6|6	0.02	2	0.56
NAE1	0|4	0.02	1	0.56
UBA3	2|3	0.02	1.43	0.56
DLG1	9|8	0.02	2.12	0.56
NDUFS2	9|8	0.02	2.12	0.56
SEC24B	4|0	0.02	1	0.56
LRRC41	4|2	0.02	1.5	0.56
ORC6	2|0	0.02	0.8	0.56
TOMM70	4|0	0.02	1	0.56
DDX41	4|2	0.02	1.5	0.56
HAUS6	2|0	0.02	0.8	0.56
INTS11	0|2	0.02	0.8	0.56
SMG9	2|2	0.02	1.33	0.56
XPO4	4|0	0.02	1	0.56
PHAX	4|2	0.02	1.5	0.56
UQCC2	2|0	0.02	0.8	0.56
AES	4|0	0.02	1	0.56
RPL7A	5|5	0.02	1.82	0.57
HNRNPUL2	4|4	0.02	1.78	0.57
OCLN	2|2	0.02	1.33	0.57
BUB1B	3|0	0.02	0.86	0.57
SSBP1	2|2	0.02	1.33	0.57
SLC25A3	12|10	0.02	2.1	0.57
PRPF4B	4|3	0.02	1.56	0.57
SNX9	2|2	0.02	1.33	0.57
PTGFRN	5|4	0.02	1.8	0.57
TRAPPC9	0|3	0.02	0.86	0.57
MPLKIP	0|3	0.02	0.86	0.57
BAX	5|4	0.02	1.8	0.57
TCEANC2	9|10	0.02	2.11	0.57
NDUFAF2	5|4	0.02	1.8	0.57
VPS28	2|2	0.02	1.33	0.57
CKAP2	5|5	0.01	1.82	0.58
DCXR	2|3	0.02	1.43	0.58
EIF4G2	4|4	0.01	1.6	0.58
ATP6V1C1	2|2	0.02	1.33	0.58
RBM4	6|5	0.01	1.83	0.58
SUPT4H1	2|2	0.02	1.33	0.58

RNMT	5|3	0.02	1.6	0.58
GTF3C5	3|2	0.02	1.43	0.58
CDC42EP4	2|2	0.02	1.33	0.58
EIF3K	3|2	0.02	1.43	0.58
NDUFAF4	7|7	0.02	2	0.58
GNPAT	6|5	0.01	1.83	0.58
SPEN	3|0	0.01	0.86	0.58
ZC3H4	4|4	0.01	1.6	0.58
ZFR	2|2	0.02	1.33	0.58
HACD3	6|8	0.01	1.87	0.58
BCAS3	2|2	0.02	1.33	0.58
MTA3	5|6	0.01	1.83	0.58
HIST1H2AE	0|6	0.01	1	0.58
SRPRB	5|5	0.01	1.82	0.58
MRPS33	0|3	0.01	0.86	0.58
BCCIP	2|2	0.02	1.33	0.58
STK24	3|2	0.01	1.25	0.59
TUBB2A	50|0	0.01	1.59	0.59
THADA	0|2	0.01	0.67	0.59
RPP38	4|3	0.01	1.56	0.59
TBC1D15	8|8	0.01	2	0.59
ETFB	0|4	0.01	0.89	0.59
AASS	11|9	0.01	2	0.59
H1FX	5|8	0.01	1.73	0.59
GMPPA	3|3	0.01	1.5	0.59
DOCK4	0|2	0.01	0.67	0.59
WBP11	11|18	0.01	1.87	0.59
OTUB1	3|4	0.01	1.56	0.59
COMMD4	3|3	0.01	1.5	0.59
RFC4	3|3	0.01	1.5	0.59
C4B	3|3	0.01	1.33	0.6
TLK2	4|2	0.01	1.33	0.6
CBX5	5|4	0.01	1.64	0.6
NSDHL	4|4	0.01	1.6	0.6
PGAM5	3|3	0.01	1.33	0.6
INTS7	0|2	0.01	0.67	0.6
HSPE1-MOB4	4|3	0.01	1.4	0.6
CHMP2B	2|0	0.01	0.67	0.6
SEC22B	5|4	0.01	1.64	0.6
ERP29	5|3	0.01	1.45	0.6
ADRM1	2|0	0.01	0.67	0.6
MRTFB	2|0	0.01	0.67	0.6
PBDC1	2|0	0.01	0.67	0.6
NPLOC4	4|4	0.01	1.6	0.6
MOV10	2|0	0.01	0.67	0.6

PSIP1	6|6	0.01	1.85	0.6
OSBPL11	2|0	0.01	0.67	0.6
YTHDC2	3|3	0.01	1.33	0.6
NOL9	4|4	0.01	1.6	0.6
MRPS21	0|2	0.01	0.67	0.6
MRPL45	2|0	0.01	0.67	0.6
TRMT61A	4|5	0.01	1.64	0.6
ARHGAP42	4|5	0.01	1.64	0.6
BRAT1	2|0	0.01	0.67	0.6
ZNF598	2|2	0.01	1.14	0.6
MZT2A	2|2	0.01	1.14	0.61
BTF3L4	3|0	0.01	0.75	0.61
MAD2L1	5|3	0.01	1.45	0.61
PYGB	4|2	0.01	1.2	0.61
SMARCD1	2|2	0.01	1.14	0.61
IRS2	3|2	0.01	1.25	0.61
ACTL6A	3|0	0.01	0.75	0.61
SCAMP1	5|3	0.01	1.45	0.61
NDUFA5	2|2	0.01	1.14	0.61
RBM6	3|4	0.01	1.4	0.61
PAIP1	0|3	0.01	0.75	0.61
TMOD3	7|4	0.01	1.57	0.61
ERO1A	3|2	0.01	1.25	0.61
RCOR1	5|3	0.01	1.45	0.61
RBMX2	0|3	0.01	0.75	0.61
PTRH2	6|5	0.01	1.69	0.61
THUMPD1	3|4	0.01	1.4	0.61
FOCAD	3|2	0.01	1.25	0.61
ARFGAP1	3|2	0.01	1.25	0.61
AP2S1	2|2	0.01	1.14	0.61
LRWD1	2|3	0.01	1.25	0.61
RFC5	2|2	0.01	1.14	0.61
PRDX5	2|2	0.01	1.14	0.61
HPX	3|3	0	1.33	0.62
SRSF5	3|3	0	1.33	0.62
SMARCC2	10|10	0.01	2	0.62
RPN2	6|7	0	1.73	0.62
ARHGAP5	2|0	0	0.57	0.62
PRR36	2|0	0	0.57	0.62
MTAP	2|0	0	0.57	0.62
PPP1CB	0|5	0	0.83	0.62
VASP	0|2	0	0.57	0.62
AP2M1	2|0	0	0.57	0.62
WDHD1	18|12	0	1.88	0.62
BRD3	4|6	0.01	1.54	0.62

PLD3	2|0	0	0.57	0.62
EMC1	0|2	0	0.57	0.62
DDX49	3|3	0	1.33	0.62
ESS2	3|3	0	1.33	0.62
C1orf35	3|3	0	1.33	0.62
VPS41	2|0	0	0.57	0.62
COG7	2|0	0	0.57	0.62
RFC2	3|3	0	1.33	0.62
ZNF326	3|4	0	1.4	0.62
FGFR1OP	0|2	0	0.57	0.62
RPL9	4|4	0	1.45	0.63
CTNND1	9|6	0	1.67	0.63
PEX5	3|0	0	0.67	0.63
DCAF6	0|2	0	0.57	0.63
NOP2	5|4	0	1.5	0.63
EIF4A2	11|9	0	1.9	0.63
ME2	6|3	0	1.29	0.63
ATXN2	4|2	0	1.2	0.63
URI1	2|0	0	0.57	0.63
EIF3M	5|6	0	1.57	0.63
SNAPIN	2|0	0	0.57	0.63
GEMIN4	3|2	0	1.11	0.63
RPS27L	4|4	0	1.33	0.63
CDK12	7|7	0	1.75	0.63
WDR11	5|7	0	1.6	0.63
DAZAP1	0|2	0	0.57	0.63
SDF2L1	5|7	0	1.6	0.63
MARCKSL1	4|3	0	1.27	0.63
SPATS2	2|0	0	0.57	0.63
CCDC134	7|7	0	1.75	0.63
FBXO30	2|0	0	0.57	0.63
PFN2	0|2	0	0.57	0.63
PRRC1	4|4	0	1.45	0.63
MAP4K4	5|3	0	1.33	0.63
NADK2	4|0	0	0.8	0.63
FECH	6|3	0	1.29	0.64
RPL11	4|3	0	1.27	0.64
RCC1	6|6	0	1.6	0.64
PLOD3	8|9	0	1.79	0.64
C12orf65	3|3	0	1.2	0.64
QPCTL	3|4	0	1.27	0.64
CHD8	4|0	0	0.73	0.64
SRI	3|3	0	1.2	0.64
SMARCC1	5|6	0	1.57	0.64
VAPA	6|5	0	1.57	0.64

FUBP3	3|0	0	0.6	0.64
NDUFS3	5|5	0	1.54	0.64
CIAO1	4|3	0	1.27	0.64
PIP4K2A	2|3	0	1.11	0.64
HINT1	3|0	0	0.67	0.64
AFF4	7|9	0	1.68	0.64
TRIM33	11|8	0	1.73	0.64
TACO1	2|3	0	1.11	0.64
PPIL3	6|5	0	1.57	0.64
PTPN13	4|6	0	1.43	0.64
FAM76B	3|2	0	1.11	0.64
EMD	4|5	0	1.38	0.65
HPRT1	5|4	0	1.38	0.65
TROVE2	5|0	0	0.77	0.65
RRBP1	9|9	0	1.8	0.65
CHCHD4	4|5	0	1.38	0.65
ACADM	7|5	0	1.5	0.65
TNIK	5|3	0	1.23	0.65
ARL1	4|4	0	1.33	0.65
SRSF7	5|5	0	1.43	0.65
CDC20	4|4	0	1.33	0.65
POLR2E	8|6	0	1.56	0.65
PRKACA	6|8	0	1.56	0.65
RFC1	6|8	0	1.56	0.65
RPN1	24|19	0	2	0.65
COX5A	3|0	0	0.6	0.65
GATD3A	3|5	0	1.23	0.65
SEL1L	7|5	0	1.5	0.65
ARPC1B	5|3	0	1.23	0.65
PLIN3	7|3	0	1.25	0.65
TIMM44	16|16	0	2.06	0.65
SLU7	7|10	0	1.62	0.65
TCERG1	24|22	0	2.14	0.65
PUM1	4|2	0	1	0.65
ANKLE2	4|4	0	1.33	0.65
JMJD6	4|4	0	1.33	0.65
SCFD1	4|7	0	1.38	0.65
MRPS12	4|4	0	1.33	0.65
VPS11	3|2	0	1	0.65
P3H1	5|3	0	1.23	0.65
ANAPC1	4|2	0	1	0.65
ACSL4	10|8	0	1.71	0.65
GNAS	5|3	0	1.23	0.65
SLFN11	9|8	0	1.7	0.66
ADD1	9|8	0	1.7	0.66

TCOF1	30|28	0	2.19	0.66
LYAR	10|11	0	1.83	0.66
ENY2	0|3	0	0.6	0.66
RAB9A	3|3	0	1.09	0.66
SERPINH1	6|6	0	1.5	0.66
RAP1B	5|5	0	1.43	0.66
FAU	7|6	0	1.53	0.66
RING1	3|3	0	1.09	0.66
MRPL12	3|3	0	1.09	0.66
SNRPG	3|3	0	1.09	0.66
SRM	3|2	0	1	0.66
TPT1	3|3	0	1.09	0.66
LLGL1	5|3	0	1.14	0.66
SDHA	6|6	0	1.5	0.66
ASNA1	6|9	0	1.5	0.66
RAB4A	0|3	0	0.6	0.66
HMGN2	6|7	0	1.53	0.66
ACTR3	9|7	0	1.6	0.66
DCAF7	8|7	0	1.58	0.66
SF3B4	3|2	0	1	0.66
NISCH	5|5	0	1.43	0.66
GPN1	3|0	0	0.6	0.66
GET4	3|2	0	1	0.66
TAF9B	3|2	0	1	0.66
RAB14	3|3	0	1.09	0.66
POLR3B	5|5	0	1.43	0.66
PBK	2|4	0	1	0.66
UGGT1	11|9	0	1.74	0.66
RPRD1B	3|2	0	1	0.66
TUBAL3	6|0	0	0.8	0.66
NTPCR	6|6	0	1.5	0.66
SNX1	3|0	0	0.6	0.66
PPFIA1	5|5	0	1.43	0.66
NCAM1	11|9	0	1.74	0.66
LIG1	5|0	0	0.71	0.67
BLMH	5|4	0	1.29	0.67
PCCB	16|15	0	2	0.67
BCS1L	4|2	0	1	0.67
ARHGEF7	6|4	0	1.25	0.67
PFKFB3	7|8	0	1.58	0.67
CDK5	0|4	0	0.67	0.67
CNOT2	4|3	0	1.17	0.67
CORO7-PAM16	4|5	0	1.29	0.67
TPD52L2	5|3	0	1.14	0.67
TRAPPC3	4|3	0	1.17	0.67

ARF1	4|5	0	1.29	0.67
OSBP	4|4	0	1.23	0.67
PPP1R10	6|8	0	1.47	0.67
SAFB	8|6	0	1.47	0.67
SRSF6	6|5	0	1.38	0.67
GNAI3	3|4	0	1.17	0.67
FKBP15	4|4	0	1.23	0.67
ZNF593	4|4	0	1.23	0.67
LAP3	10|6	0	1.45	0.67
NUP54	8|6	0	1.47	0.67
EIF4ENIF1	4|3	0	1.17	0.67
NMT1	3|0	0	0.55	0.67
CLSPN	2|4	0	1	0.67
C12orf43	3|4	0	1.17	0.67
CHID1	5|4	0	1.29	0.67
TRIR	7|5	0	1.41	0.67
CTNNBL1	5|2	0	1	0.67
CPVL	8|10	0	1.64	0.67
SARNP	7|8	0	1.58	0.67
CNP	5|6	0	1.38	0.67
TJP1	8|6	0	1.47	0.67
OXCT1	6|6	0	1.41	0.68
XRN1	3|4	0	1.08	0.68
SCAF4	5|4	0	1.2	0.68
PITRM1	2|4	0	0.92	0.68
GLS	3|2	0	0.91	0.68
ARPC5	4|2	0	0.92	0.68
PNN	0|4	0	0.62	0.68
STAM	8|8	0	1.6	0.68
CPNE1	7|7	0	1.47	0.68
USP11	11|6	0	1.42	0.68
SCO2	3|2	0	0.91	0.68
PPIF	5|4	0	1.2	0.68
HEXIM1	4|4	0	1.14	0.68
TALDO1	5|4	0	1.2	0.68
APPL1	4|3	0	1.08	0.68
C11orf58	3|4	0	1.08	0.68
POP1	12|10	0	1.69	0.68
SF3B6	3|4	0	1.08	0.68
COMMD10	3|2	0	0.91	0.68
HYPK	4|2	0	0.92	0.68
ZCCHC17	3|4	0	1.08	0.68
TRMT10C	11|11	0	1.76	0.68
ATF7IP	3|2	0	0.91	0.68
CMAS	9|10	0	1.65	0.68

UBR4	5|0	0	0.67	0.68
HIST1H2BB	7|8	0	1.5	0.68
EIF4H	4|4	0	1.14	0.68
COLGALT1	8|9	0	1.62	0.68
NUP85	10|8	0	1.57	0.68
SON	5|4	0	1.2	0.68
WDR83	3|2	0	0.91	0.68
RPL8	4|4	0	1.14	0.68
GADD45GIP1	5|5	0	1.33	0.68
DOCK11	8|7	0	1.5	0.68
MRPS9	3|0	0	0.55	0.68
STAG2	20|14	0	1.74	0.69
RPP30	6|5	0	1.22	0.69
DKC1	11|11	0	1.69	0.69
PYGL	2|4	0	0.86	0.69
LTA4H	4|2	0	0.86	0.69
NFKB2	4|6	0	1.18	0.69
NUP88	6|6	0	1.33	0.69
PPP4C	5|5	0	1.25	0.69
PTPN11	10|6	0	1.39	0.69
SMARCE1	8|5	0	1.3	0.69
SORD	4|0	0	0.57	0.69
SRP14	6|5	0	1.29	0.69
UBE2N	5|5	0	1.25	0.69
HMGA2	3|4	0	1	0.69
HOMER2	5|6	0	1.29	0.69
GNL1	5|3	0	1	0.69
GNPDA1	4|6	0	1.18	0.69
TARBP1	5|2	0	0.88	0.69
CWC27	5|7	0	1.26	0.69
POLR3C	4|2	0	0.86	0.69
RALBP1	4|0	0	0.57	0.69
POLR3A	6|5	0	1.22	0.69
XPOT	4|3	0	1	0.69
TARDBP	7|7	0	1.47	0.69
LSM1	4|3	0	1	0.69
MYBBP1A	9|8	0	1.55	0.69
GLOD4	3|5	0	1.07	0.69
KDM3B	14|7	0	1.4	0.69
CPSF2	4|3	0	1	0.69
WDR70	7|5	0	1.26	0.69
TMX3	6|8	0	1.4	0.69
CHTF18	9|7	0	1.45	0.69
NUP37	8|5	0	1.3	0.69
DHX40	6|10	0	1.39	0.69

VCPIP1	6|3	0	1.06	0.69
POLDIP3	6|4	0	1.18	0.69
RBM17	6|5	0	1.29	0.69
RPS24	3|4	0	1	0.69
KIF23	4|3	0	1	0.69
CHCHD1	6|6	0	1.33	0.69
ECI2	6|4	0	1.18	0.69
ALDH2	15|13	0	1.75	0.7
PQBP1	6|6	0	1.26	0.7
CSNK1A1	5|4	0	1.12	0.7
CSTF3	15|15	0	1.82	0.7
ELAVL1	7|6	0	1.3	0.7
ATP2B1	5|3	0	1	0.7
GLUL	4|4	0	1.07	0.7
H2AFZ	7|7	0	1.4	0.7
ITGB1	6|7	0	1.3	0.7
RANBP3	6|6	0	1.26	0.7
ALDH1A2	4|5	0	1.12	0.7
SNRNP40	2|5	0	0.88	0.7
ABCF2	3|4	0	0.93	0.7
EIF1B	5|4	0	1.12	0.7
TBCD	7|8	0	1.43	0.7
SEC23A	5|4	0	1.12	0.7
G3BP2	6|6	0	1.26	0.7
NUP62	7|7	0	1.4	0.7
MAT2B	7|6	0	1.3	0.7
MDC1	9|13	0	1.52	0.7
RABGAP1L	7|4	0	1.16	0.7
SARM1	4|7	0	1.16	0.7
TTLL12	9|7	0	1.39	0.7
IRF2BP1	6|6	0	1.26	0.7
DNAJB11	10|10	0	1.6	0.7
DARS2	10|6	0	1.33	0.7
TSR1	10|6	0	1.33	0.7
EXOC2	6|3	0	1	0.7
SCYL1	4|4	0	1.07	0.7
LRRC47	8|8	0	1.45	0.7
HIST1H3F	6|4	0	1.11	0.7
LENG1	7|3	0	1.05	0.7
TBL1XR1	10|11	0	1.62	0.7
WDR82	7|4	0	1.16	0.7
LEO1	9|5	0	1.22	0.7
BOD1L1	9|8	0	1.48	0.7
TCF20	8|11	0	1.46	0.7
KRT18	6|6	0	1.26	0.7

ELOB	5|3	0	1	0.7
BLOC1S3	3|5	0	1	0.7
ALDH3A2	10|13	0	1.53	0.71
ALDH9A1	7|3	0	1	0.71
PRIM2	10|6	0	1.28	0.71
DIAPH3	5|3	0	0.94	0.71
CLCC1	7|9	0	1.33	0.71
8-Sep	8|8	0	1.39	0.71
IKBKG	3|4	0	0.93	0.71
PPP6C	5|2	0	0.82	0.71
PIP5K1A	9|12	0	1.5	0.71
PEG10	4|3	0	0.93	0.71
ABI1	7|7	0	1.33	0.71
ANK3	9|9	0	1.5	0.71
ELOC	5|3	0	0.94	0.71
RAB5C	7|10	0	1.36	0.71
TRIM65	8|9	0	1.42	0.71
AP3S1	5|5	0	1.11	0.71
CARS	6|3	0	0.95	0.71
ITSN1	0|6	0	0.63	0.71
VPS26A	5|2	0	0.82	0.71
HMGB3	6|6	0	1.2	0.71
TSSC4	5|0	0	0.59	0.71
CLPP	7|6	0	1.24	0.71
U2AF1	7|8	0	1.36	0.71
OGFR	7|7	0	1.33	0.71
ALDH1L1	7|4	0	1.1	0.71
CORO1C	7|6	0	1.24	0.71
PELP1	11|11	0	1.57	0.71
PSME4	5|3	0	0.94	0.71
NUDCD3	5|3	0	0.94	0.71
NELFB	7|6	0	1.24	0.71
CPSF3	7|5	0	1.2	0.71
CDV3	6|6	0	1.2	0.71
IARS2	5|6	0	1.16	0.71
LIN7C	5|5	0	1.11	0.71
COPRS	4|3	0	0.93	0.71
USP28	6|7	0	1.24	0.71
TRAF2	3|4	0	0.93	0.71
FKBP10	6|6	0	1.2	0.71
RELA	7|6	0	1.24	0.71
COPS7B	5|4	0	1.06	0.71
SEC23B	3|4	0	0.93	0.71
NEK9	8|6	0	1.27	0.71
CDK11B	13|19	0	1.64	0.71

FAM133B	5|5	0	1.11	0.71
IDH3B	12|10	0	1.57	0.71
GPATCH8	8|7	0	1.25	0.72
WDR26	3|5	0	0.89	0.72
PCCA	20|23	0	1.91	0.72
VPS53	7|5	0	1.09	0.72
NDRG1	7|5	0	1.14	0.72
ANXA2	5|4	0	0.95	0.72
SEC13	5|4	0	1	0.72
ALDH7A1	10|10	0	1.48	0.72
GSTO1	6|6	0	1.2	0.72
C1QBP	20|16	0	1.67	0.72
EIF3L	12|17	0	1.53	0.72
CUL3	14|13	0	1.64	0.72
CSTF1	7|8	0	1.3	0.72
KRT19	17|20	0	1.76	0.72
PSMB6	4|5	0	0.95	0.72
SBF1	5|4	0	0.95	0.72
DHX16	7|7	0	1.22	0.72
EIF3J	4|5	0	1	0.72
PRKAR2A	6|5	0	1.1	0.72
SFSWAP	11|10	0	1.45	0.72
IDH3A	8|7	0	1.3	0.72
RAD21	12|12	0	1.6	0.72
PYCR1	12|10	0	1.52	0.72
SAP30BP	5|7	0	1.14	0.72
EIF2B2	5|3	0	0.89	0.72
CADM1	4|5	0	0.95	0.72
DIS3	13|7	0	1.29	0.72
GPKOW	6|6	0	1.14	0.72
NOP58	7|8	0	1.25	0.72
SBDS	2|5	0	0.78	0.72
RAB10	6|5	0	1.1	0.72
MAP1S	5|5	0	1.05	0.72
ZC3H15	6|9	0	1.2	0.72
CEP41	5|5	0	1.05	0.72
DNAJC10	5|4	0	1	0.72
NUP107	15|9	0	1.37	0.72
GATAD2B	6|6	0	1.2	0.72
PSAT1	7|5	0	1.14	0.72
ACBD3	7|6	0	1.18	0.72
SNIP1	5|7	0	1.09	0.72
SEH1L	7|7	0	1.27	0.72
NFATC2IP	5|5	0	1.05	0.72
PNPT1	11|6	0	1.21	0.72

PPP4R2	10|9	0	1.41	0.72
RAB11FIP1	5|6	0	1.05	0.73
KDM1A	14|9	0	1.31	0.73
RPS25	6|5	0	1.05	0.73
HIST2H2AA4	10|8	0	1.29	0.73
DYNC2H1	7|6	0	1.13	0.73
STRN3	9|7	0	1.23	0.73
TRMT1	10|10	0	1.38	0.73
AIMP1	4|6	0	0.91	0.73
RPS20	6|5	0	1.05	0.73
ACIN1	8|11	0	1.27	0.73
DSG2	7|9	0	1.23	0.73
VDAC1	14|12	0	1.53	0.73
NAA10	9|7	0	1.19	0.73
EIF3H	8|8	0	1.23	0.73
TRIP13	5|6	0	1	0.73
HOMER1	5|6	0	1	0.73
PPIG	10|11	0	1.4	0.73
NELFA	9|4	0	1	0.73
MRPS31	7|6	0	1.08	0.73
RPL10	4|6	0	0.95	0.73
ANP32A	6|6	0	1.04	0.73
NOP56	7|6	0	1.13	0.73
ARPC1A	9|8	0	1.26	0.73
GLO1	7|6	0	1.13	0.73
COPS8	9|8	0	1.26	0.73
SRP72	12|13	0	1.52	0.73
HNRNPH3	7|6	0	1.08	0.73
KPNA6	10|11	0	1.4	0.73
PRPF6	8|10	0	1.29	0.73
NUDT5	6|6	0	1.04	0.73
WASHC4	7|5	0	1	0.73
WAC	8|8	0	1.23	0.73
VPS13C	9|2	0	0.85	0.73
CYCS	7|7	0	1.17	0.73
MEPCE	16|14	0	1.62	0.73
VPS18	7|9	0	1.23	0.73
GRPEL1	10|7	0	1.21	0.73
LAS1L	7|5	0	1.04	0.73
HNRNPAB	7|6	0	1.08	0.73
ABRAXAS2	7|7	0	1.17	0.73
PNISR	6|6	0	1.04	0.73
HK1	8|7	0	1.2	0.73
CDK2	7|6	0	1.08	0.73
TMEM263	6|4	0	0.95	0.73

ARPC2	9|4	0	0.96	0.73
SF1	9|9	0	1.29	0.73
ENSA	5|7	0	1.04	0.73
RPL5	9|6	0	1.03	0.74
RPL22	7|7	0	1.12	0.74
RPS5	7|6	0	1.04	0.74
TIMM50	10|11	0	1.35	0.74
TWISTNB	7|10	0	1.17	0.74
PSPC1	14|12	0	1.44	0.74
EIF2B3	11|13	0	1.41	0.74
ISYNA1	7|6	0	1.04	0.74
RIF1	12|10	0	1.33	0.74
MAP7	7|7	0	1.12	0.74
CD2BP2	5|6	0	0.96	0.74
ACADVL	14|13	0	1.5	0.74
CSNK2B	7|7	0	1.12	0.74
APEX1	8|8	0	1.19	0.74
BSG	7|7	0	1.08	0.74
CTNNA1	9|10	0	1.23	0.74
EPS15	13|9	0	1.29	0.74
RBMX	5|7	0	0.92	0.74
KPNA1	9|10	0	1.27	0.74
NPM1	11|10	0	1.27	0.74
PPP2CA	8|6	0	1.04	0.74
TXN	8|7	0	1.15	0.74
EIF3F	8|8	0	1.19	0.74
BAG3	5|7	0	0.96	0.74
SNRPD2	6|7	0	1.04	0.74
BUB3	8|9	0	1.17	0.74
DDX23	7|6	0	1	0.74
MTOR	8|7	0	1.11	0.74
HIST1H1C	7|6	0	1.04	0.74
CCDC6	9|8	0	1.13	0.74
SRSF4	7|6	0	1	0.74
ALYREF	11|14	0	1.39	0.74
RBM12	12|10	0	1.29	0.74
IK	13|12	0	1.39	0.74
PSME1	9|6	0	1.07	0.74
ANP32B	10|8	0	1.24	0.74
PGRMC1	6|8	0	1.08	0.74
TCEA1	7|6	0	1	0.74
CCDC22	9|5	0	0.97	0.74
TTC37	14|15	0	1.49	0.74
MESD	6|7	0	1	0.74
UBR5	4|6	0	0.87	0.74

HSD17B12	10|10	0	1.33	0.74
NUP98	16|13	0	1.49	0.74
SMU1	11|12	0	1.35	0.74
EML4	7|8	0	1.15	0.74
HIST1H2BD	9|10	0	1.27	0.74
EPS15L1	10|9	0	1.23	0.74
RBM25	8|10	0	1.2	0.74
RBM26	6|7	0	1	0.74
MCCC2	11|11	0	1.33	0.74
PHF6	6|5	0	0.96	0.74
AP1M1	12|12	0	1.41	0.74
RTN4	10|9	0	1.23	0.74
FH	8|6	0	0.97	0.75
CTNNB1	11|10	0	1.2	0.75
PDS5A	17|16	0	1.43	0.75
PSMA4	10|9	0	1.15	0.75
SHTN1	8|6	0	0.9	0.75
FIP1L1	12|11	0	1.24	0.75
TNPO2	12|10	0	1.16	0.75
P4HA1	12|11	0	1.28	0.75
API5	7|8	0	1.07	0.75
SLC25A5	15|14	0	1.38	0.75
RAB3GAP1	12|8	0	1.08	0.75
RPL17	8|8	0	1.07	0.75
C7orf55-LUC7L2	33|31	0	1.91	0.75
PAF1	10|11	0	1.17	0.75
ECH1	10|11	0	1.17	0.75
EIF2B1	8|7	0	1	0.75
EIF2S3	10|10	0	1.18	0.75
ATP6V1E1	5|8	0	0.84	0.75
ESD	8|7	0	1.07	0.75
FKBP3	13|11	0	1.23	0.75
POLD1	16|13	0	1.38	0.75
PSMB4	11|10	0	1.17	0.75
SET	12|10	0	1.16	0.75
TMPO	29|24	0	1.68	0.75
TRIP6	8|7	0	1.07	0.75
CUL2	9|10	0	1.12	0.75
EIF3D	7|8	0	1.07	0.75
BUD31	7|5	0	0.89	0.75
FEN1	13|10	0	1.21	0.75
GTF2F2	12|10	0	1.22	0.75
ILF2	16|16	0	1.42	0.75
RAB7A	7|8	0	1.03	0.75
EIF1AY	18|17	0	1.52	0.75

SYMPK	27|24	0	1.73	0.75
TLE3	11|10	0	1.24	0.75
SRRM1	11|12	0	1.31	0.75
BASP1	8|8	0	1.03	0.75
SNRNP27	6|8	0	0.97	0.75
RABGAP1	8|7	0	0.97	0.75
LSM4	8|7	0	1.07	0.75
UBL4A	8|6	0	0.97	0.75
ZNF638	13|15	0	1.4	0.75
PRPF19	17|15	0	1.42	0.75
MDN1	7|14	0	1.11	0.75
NUP93	16|13	0	1.41	0.75
CEP170	9|11	0	1.21	0.75
MON2	9|10	0	1.19	0.75
NUP205	18|18	0	1.5	0.75
RTF1	12|11	0	1.24	0.75
RPRD2	22|23	0	1.7	0.75
RPAP1	8|6	0	0.9	0.75
CIAO2B	8|6	0	1	0.75
CWC25	10|9	0	1.12	0.75
SARS2	9|8	0	1.03	0.75
GPALPP1	12|10	0	1.26	0.75
MATR3	18|20	0	1.55	0.75
WRNIP1	14|11	0	1.28	0.75
CAMK2D	12|12	0	1.26	0.75
IRF2BP2	11|8	0	1.12	0.75
ACAT1	12|11	0	1.15	0.76
MSH2	17|15	0	1.31	0.76
RPL23A	10|9	0	1	0.76
CYFIP2	12|9	0	1.08	0.76
SREK1	11|15	0	1.21	0.76
TP53BP1	14|15	0	1.23	0.76
EPB41	15|14	0	1.29	0.76
GNL3L	10|11	0	1.05	0.76
RPS10	11|11	0	1.13	0.76
AFDN	14|12	0	1.24	0.76
CCNT1	19|20	0	1.42	0.76
CSRP2	9|9	0	1.03	0.76
FBL	16|15	0	1.32	0.76
SLC25A6	15|14	0	1.32	0.76
PSMA6	14|15	0	1.23	0.76
PSMB1	14|14	0	1.22	0.76
RCN1	9|11	0	1	0.76
DEK	13|14	0	1.23	0.76
EDF1	11|9	0	1.05	0.76

AMPD2	11|8	0	1.03	0.76
CS	20|17	0	1.42	0.76
COIL	15|17	0	1.31	0.76
FAM50A	10|9	0	1.06	0.76
LONP1	32|37	0	1.73	0.76
ROCK2	15|8	0	1.02	0.76
SEC24C	8|9	0	0.97	0.76
HDAC1	16|11	0	1.12	0.76
MRE11	16|14	0	1.3	0.76
RAD50	23|21	0	1.54	0.76
PRDX2	10|10	0	1.05	0.76
TFG	13|15	0	1.24	0.76
NUMA1	9|12	0	1.11	0.76
PCBP1	11|11	0	1.07	0.76
IQGAP2	37|28	0	1.62	0.76
9-Sep	18|18	0	1.38	0.76
SRSF1	10|10	0	1.05	0.76
SKIV2L	12|7	0	0.97	0.76
HNRNPUL1	18|17	0	1.46	0.76
SNW1	13|11	0	1.2	0.76
XRN2	19|20	0	1.47	0.76
LETM1	13|15	0	1.19	0.76
RAB3GAP2	17|12	0	1.18	0.76
SRP68	10|10	0	1.11	0.76
6-Sep	11|9	0	1.03	0.76
NUP160	18|19	0	1.45	0.76
LARP7	18|14	0	1.23	0.76
TXNDC12	21|25	0	1.56	0.76
TMA7	14|13	0	1.23	0.76
DNAAF5	10|8	0	1	0.76
PPP6R3	12|7	0	0.95	0.76
LRRC59	14|10	0	1.12	0.76
DPYSL5	10|8	0	1.03	0.76
JUP	11|15	0	1.13	0.76
RPS18	9|10	0	1.09	0.76
NT5DC2	14|16	0	1.28	0.76
SLIRP	9|10	0	1	0.76
HNRNPC	12|11	0	1.1	0.76
FANCD2	18|17	0	1.35	0.76
MYL12B	11|10	0	1.05	0.76
AIFM1	14|15	0	1.29	0.76
CWF19L2	9|8	0	0.97	0.76
LMNA	19|19	0	1.46	0.76
SCRIB	24|18	0	1.45	0.76
GPS1	10|9	0	0.97	0.76

HADHA	17|16	0	1.25	0.77
HADHB	12|13	0	1.11	0.77
POLR2B	26|26	0	1.46	0.77
RPSA	11|8	0	0.93	0.77
CAST	15|14	0	1.12	0.77
FANCI	20|18	0	1.25	0.77
SRRT	13|11	0	1	0.77
RBBP4	10|13	0	1	0.77
RNF40	21|21	0	1.4	0.77
CSNK2A2	17|14	0	1.19	0.77
CTPS1	12|8	0	0.93	0.77
GTF2F1	16|12	0	1.08	0.77
HMGB2	19|21	0	1.33	0.77
TNPO1	12|16	0	1.12	0.77
PRKCSH	23|20	0	1.41	0.77
PSMB2	12|14	0	1.08	0.77
PSMB3	13|11	0	1.09	0.77
PSMB5	15|13	0	1.08	0.77
PSMD13	14|9	0	0.96	0.77
NELFE	12|10	0	1	0.77
SNRPA1	12|12	0	1.04	0.77
COPS3	13|11	0	1.09	0.77
EIF2S1	13|12	0	1.11	0.77
FARSA	15|11	0	1.08	0.77
MED1	18|18	0	1.31	0.77
NUP153	27|26	0	1.51	0.77
GLUD1	25|24	0	1.44	0.77
HCFC1	24|23	0	1.38	0.77
UBA2	15|12	0	1.06	0.77
HNRNPR	13|9	0	0.92	0.77
MDH2	14|13	0	1.08	0.77
ARID1A	16|15	0	1.22	0.77
TACC3	14|10	0	1.02	0.77
CHERP	18|18	0	1.22	0.77
PHGDH	14|13	0	1.1	0.77
UGP2	12|8	0	0.85	0.77
SF3A3	14|13	0	1.12	0.77
COPS6	14|12	0	1.06	0.77
COPS5	12|9	0	0.95	0.77
SF3B2	11|10	0	1	0.77
SNRPD1	8|11	0	0.9	0.77
NUDT21	18|16	0	1.17	0.77
CPSF6	12|11	0	1	0.77
COPE	17|13	0	1.15	0.77
RTCB	20|17	0	1.21	0.77

EDC4	14|13	0	1.08	0.77
DCAF1	14|11	0	0.98	0.77
PDAP1	13|14	0	1.15	0.77
PDXDC1	15|11	0	1.02	0.77
CLASP2	12|12	0	1.02	0.77
COPS4	21|16	0	1.17	0.77
PRPF38B	16|13	0	1.16	0.77
11-Sep	18|16	0	1.21	0.77
WDR33	17|18	0	1.21	0.77
RBM27	22|23	0	1.45	0.77
RNF20	24|18	0	1.35	0.77
XPO5	22|10	0	1.07	0.77
PPA1	12|10	0	0.98	0.77
HIST1H4J	13|12	0	1	0.77
PRPF38A	12|11	0	1	0.77
RBM33	19|26	0	1.27	0.77
DDX39B	16|16	0	1.28	0.77
PSMA3	15|14	0	1.21	0.77
ATP2A2	16|14	0	1.22	0.77
FAM98B	12|9	0	0.98	0.77
HNRNPA3	18|14	0	1.12	0.77
NME1	16|15	0	1.17	0.77
NCKAP1	12|11	0	1	0.77
MSH6	25|20	0	1.23	0.78
OAT	16|18	0	1.03	0.78
RPL23	17|16	0	1.1	0.78
RPS7	14|14	0	1.04	0.78
NME1-NME2	19|18	0	1.07	0.78
PAICS	20|20	0	1.14	0.78
U2SURP	36|34	0	1.46	0.78
MGA	16|17	0	1	0.78
THOC2	19|15	0	1.05	0.78
HNRNPF	21|20	0	1.17	0.78
DDX17	22|21	0	1.3	0.78
MAP4	17|20	0	1.03	0.78
COPS2	17|13	0	1	0.78
SRSF11	19|16	0	1.11	0.78
NSUN2	22|18	0	1.08	0.78
DPYSL3	27|18	0	1.17	0.78
RBBP7	19|19	0	1.15	0.78
EIF3C	13|15	0	0.97	0.78
CALU	21|16	0	0.97	0.78
PRDX1	23|21	0	1.14	0.78
CHD4	35|42	0	1.57	0.78
CPD	22|18	0	1.04	0.78

DARS	12|16	0	0.93	0.78
AHNAK	56|46	0	1.7	0.78
7-Sep	19|19	0	1.15	0.78
PSMA2	12|15	0	0.98	0.78
RANGAP1	14|16	0	1.02	0.78
RPA1	22|20	0	1.29	0.78
SNRNP70	16|16	0	1.14	0.78
SRP54	14|13	0	1.02	0.78
SSRP1	15|12	0	0.98	0.78
SUPT6H	37|39	0	1.5	0.78
ELOA	17|16	0	1.12	0.78
TPP2	35|27	0	1.28	0.78
CUL4B	23|20	0	1.16	0.78
YARS	19|18	0	1.16	0.78
KHSRP	32|27	0	1.28	0.78
EIF3B	20|17	0	1.04	0.78
FUBP1	13|13	0	1.02	0.78
DDX6	13|11	0	0.94	0.78
2-Sep	17|16	0	1.12	0.78
MTA2	19|17	0	1.06	0.78
PRDX6	14|15	0	1.07	0.78
FARSB	15|15	0	1.09	0.78
PSME3	20|19	0	1.1	0.78
DNAJA2	14|11	0	0.91	0.78
HNRNPM	16|18	0	1.11	0.78
TUBB4A	41|41	0	1.67	0.78
CAPZA1	15|14	0	1	0.78
PIN4	17|16	0	1.06	0.78
VAT1	13|11	0	0.94	0.78
SPIN1	18|17	0	1.08	0.78
EWSR1	33|34	0	1.38	0.78
PRDX3	22|19	0	1.26	0.78
HTATSF1	16|13	0	1.02	0.78
SLK	18|15	0	1.05	0.78
XPO7	23|21	0	1.13	0.78
PRRC2C	18|15	0	0.93	0.78
TBC1D10B	19|18	0	1.07	0.78
ARL6IP4	19|19	0	1.19	0.78
POLA1	25|19	0	1.22	0.78
ILF3	32|30	0	1.46	0.78
CCAR1	18|20	0	1.07	0.78
SNRPN	18|15	0	0.96	0.78
NSRP1	17|15	0	0.96	0.78
LMNB2	18|17	0	1.19	0.78
CMBL	19|13	0	0.89	0.78

TAF15	27|22	0	1.2	0.78
AP1B1	18|12	0	0.88	0.78
PSMA1	20|19	0	1.15	0.78
NUP155	25|22	0	1.31	0.78
RBM39	20|18	0	1.09	0.78
PRMT1	21|20	0	1.09	0.78
MIA3	16|12	0	0.92	0.78
LUC7L	17|19	0	1.14	0.78
KLC4	17|16	0	1.14	0.78
KRT9	44|54	0	1.19	0.79
KRT2	38|38	0	1.09	0.79
ATP1A1	23|18	0	0.96	0.79
P4HB	25|23	0	1.02	0.79
POLR2A	24|23	0	1.15	0.79
PPIB	49|57	0	1.29	0.79
RPL38	33|27	0	1.14	0.79
ECPAS	31|29	0	1.07	0.79
CAMSAP3	30|29	0	1.09	0.79
ACTA1	28|31	0	1.01	0.79
SUPT5H	34|33	0	1.19	0.79
EZR	26|27	0	1.02	0.79
ACTN1	38|38	0	1.23	0.79
HYOU1	32|32	0	1.11	0.79
RCC2	32|39	0	1.07	0.79
NONO	33|30	0	1.22	0.79
RBM10	44|49	0	1.16	0.79
DHX9	37|38	0	1.35	0.79
DHX15	29|25	0	1.05	0.79
EPB41L2	31|38	0	1.23	0.79
XRCC6	35|34	0	1.03	0.79
ARCN1	23|25	0	1.07	0.79
ATP5F1B	29|27	0	1.09	0.79
CANX	40|41	0	1.25	0.79
DDB1	36|38	0	1.03	0.79
GSPT1	21|20	0	1.05	0.79
HMGB1	37|35	0	1.09	0.79
HSPA4	40|31	0	0.96	0.79
PFKP	25|21	0	0.94	0.79
PSMA7	20|16	0	0.91	0.79
ALDH18A1	38|32	0	1.13	0.79
SSB	26|20	0	1.06	0.79
TAF4	28|27	0	1.07	0.79
TPR	47|56	0	1.32	0.79
VIM	31|34	0	1.15	0.79
XPO1	29|25	0	1	0.79

USP7	38|34	0	1.3	0.79
USO1	33|28	0	1.26	0.79
EIF3A	40|42	0	1.31	0.79
EIF3I	17|23	0	0.89	0.79
HSPA9	49|45	0	1.2	0.79
CALR	20|23	0	1.04	0.79
HNRNPU	29|23	0	0.96	0.79
MTA1	23|25	0	1.12	0.79
PDIA4	27|28	0	1.04	0.79
DDX1	23|25	0	1.07	0.79
SFPQ	27|24	0	1.09	0.79
THRAP3	44|44	0	1.41	0.79
PDIA3	21|20	0	1	0.79
SHMT2	24|27	0	1.1	0.79
SMC3	37|40	0	1.12	0.79
LMNB1	35|34	0	1.18	0.79
TRIM28	53|50	0	1.29	0.79
SF3A1	31|30	0	1.12	0.79
MCM6	31|31	0	1.02	0.79
MTHFD1	33|28	0	0.97	0.79
TUBB4B	51|51	0	1.23	0.79
RANBP2	64|65	0	1.6	0.79
SMC1A	39|40	0	1.16	0.79
RUVBL2	33|28	0	1.04	0.79
SUB1	40|35	0	1.08	0.79
U2AF2	31|28	0	1.13	0.79
DDX42	33|29	0	1.11	0.79
COPG2	21|22	0	1.04	0.79
EPB41L3	29|32	0	1.03	0.79
SF3B3	37|37	0	1.03	0.79
IARS	28|27	0	1.06	0.79
PUF60	33|33	0	1.06	0.79
SART3	27|23	0	1.04	0.79
BCLAF1	62|65	0	1.4	0.79
ESYT1	26|23	0	1.13	0.79
LUC7L2	38|36	0	1.06	0.79
CNOT1	22|18	0	0.98	0.79
TRAP1	32|34	0	1.12	0.79
LUC7L3	21|20	0	1	0.79
NUP133	27|19	0	0.92	0.79
CAND1	29|25	0	1.02	0.79
LARS	27|24	0	1.15	0.79
CCAR2	16|22	0	0.9	0.79
HNRNPA2B1	32|27	0	0.98	0.79
TUBB6	30|27	0	1.16	0.79

TUBA1C	41|0	0	0.66	0.79
PRRC2A	31|26	0	1.19	0.79
RAVER1	27|28	0	1.15	0.79
PPIL4	32|30	0	1.1	0.79
CSNK2A1	28|30	0	1.01	0.79
PPM1B	25|31	0	1.07	0.79
TUBB2B	50|48	0	1.27	0.79
KLC1	19|20	0	0.99	0.79
G3BP1	30|28	0	0.99	0.79
HDLBP	21|16	0	0.96	0.79
ADSL	6|7	0	0.74	0.8
GPI	3|3	0	0.32	0.8
PNP	8|7	0	0.77	0.8
PGK1	17|13	0	0.86	0.8
PLOD1	9|12	0	0.82	0.8
PTS	12|9	0	0.6	0.8
RB1	2|0	0	0.31	0.8
HSD17B4	13|12	0	0.71	0.8
APRT	2|2	0	1	0.8
AHCY	15|14	0	0.83	0.8
BLVRA	2|0	0	0.5	0.8
IGF2R	3|3	0	0.44	0.8
IMPDH2	12|8	0	0.65	0.8
PDHB	7|5	0	0.77	0.8
PRIM1	10|7	0	0.69	0.8
RPL4	4|0	0	0.32	0.8
RPL6	4|2	0	0.71	0.8
RPL12	3|3	0	0.92	0.8
RPL19	0|2	0	0.36	0.8
RPL24	2|2	0	1	0.8
RPL26	3|4	0	0.5	0.8
RPL27	2|0	0	0.25	0.8
RPL27A	2|2	0	0.89	0.8
RPLP2	7|5	0	0.75	0.8
RPS3	17|17	0	0.92	0.8
RPS3A	7|6	0	0.55	0.8
RPS4X	15|15	0	0.94	0.8
RPS6	0|2	0	0.29	0.8
SMC4	39|39	0	0.88	0.8
RPS8	4|4	0	0.62	0.8
RPS9	9|8	0	0.81	0.8
DNM1	8|0	0	0.48	0.8
WASHC2A	8|10	0	0.64	0.8
RPS11	6|6	0	1	0.8
ARHGAP17	5|3	0	0.8	0.8

RPS13	5|5	0	0.69	0.8
ENAH	11|10	0	0.79	0.8
CUL4A	9|11	0	0.89	0.8
CKAP5	24|27	0	0.63	0.8
RPS15	4|4	0	0.76	0.8
CADPS2	7|3	0	0.62	0.8
RPS15A	5|5	0	0.77	0.8
RPS16	9|10	0	0.83	0.8
RPS19	8|8	0	0.74	0.8
SLTM	14|16	0	0.92	0.8
RPS21	2|2	0	0.44	0.8
BAG5	2|0	0	0.29	0.8
RPS23	5|5	0	0.91	0.8
HSP90AA1	117|97	0	0.91	0.8
TPM1	4|3	0	0.58	0.8
SERBP1	53|52	0	1.14	0.8
RPS26	5|3	0	0.76	0.8
ABCF1	10|13	0	0.82	0.8
RPS27	6|6	0	0.89	0.8
RPS28	2|2	0	0.89	0.8
PLAA	2|3	0	0.62	0.8
INF2	6|4	0	0.65	0.8
HNRNPH2	10|9	0	0.9	0.8
USP9X	51|55	0	0.98	0.8
YARS2	4|0	0	0.47	0.8
PPP4R1	2|2	0	0.44	0.8
MAZ	5|5	0	1	0.8
SEC31A	21|21	0	0.88	0.8
KTN1	4|4	0	0.89	0.8
MYO1C	0|2	0	0.22	0.8
PRKDC	83|94	0	1.25	0.8
ACLY	59|53	0	0.83	0.8
ACO2	3|5	0	0.7	0.8
HMGCS1	2|0	0	0.21	0.8
RPL31	3|2	0	0.67	0.8
IFT74	2|2	0	0.8	0.8
OTUD4	3|4	0	0.67	0.8
GIGYF2	5|7	0	0.55	0.8
CAP1	23|21	0	0.83	0.8
BRD2	9|7	0	0.84	0.8
NACA	0|3	0	0.33	0.8
DBNL	4|4	0	0.89	0.8
PARK7	6|7	0	0.81	0.8
AP1G1	5|6	0	0.71	0.8
CSK	3|0	0	0.33	0.8

POLD2	4|3	0	0.82	0.8
ALDOA	21|17	0	0.71	0.8
ETFA	3|4	0	0.61	0.8
EEF1D	14|15	0	0.83	0.8
EPN1	2|2	0	0.53	0.8
KARS	5|4	0	0.45	0.8
CSDE1	14|9	0	0.68	0.8
SPAG9	36|40	0	0.9	0.8
DNMT1	5|5	0	0.49	0.8
LRRFIP2	2|2	0	0.67	0.8
NT5C2	2|0	0	0.29	0.8
IPO11	2|0	0	0.33	0.8
DCTN1	14|12	0	0.63	0.8
TKT	2|4	0	0.32	0.8
TAF12	0|2	0	0.5	0.8
DCTN4	0|3	0	0.35	0.8
PABPC4	9|8	0	0.67	0.8
YWHAZ	15|13	0	0.82	0.8
FDPS	2|0	0	0.5	0.8
GART	38|30	0	0.71	0.8
APP	3|3	0	0.8	0.8
PLS3	3|3	0	0.3	0.8
CPSF7	7|6	0	0.93	0.8
CISD3	3|3	0	0.92	0.8
MCTS1	0|2	0	0.36	0.8
CLASP1	2|2	0	1	0.8
MORF4L2	6|6	0	1	0.8
EIF5A	8|0	0	0.36	0.8
PPP1R12A	5|3	0	0.44	0.8
NDE1	2|0	0	0.44	0.8
COPB1	37|41	0	1	0.8
EXOC7	6|9	0	0.83	0.8
SETDB1	2|0	0	0.44	0.8
TTC28	5|3	0	0.48	0.8
STMN1	6|5	0	0.92	0.8
FKBP5	17|14	0	0.9	0.8
PAFAH1B3	2|0	0	0.44	0.8
SNCA	6|5	0	0.79	0.8
TPI1	0|2	0	0.31	0.8
COPS7A	5|5	0	0.74	0.8
LDHA	10|8	0	0.67	0.8
CCT7	36|32	0	0.69	0.8
PFKM	11|11	0	0.83	0.8
ATXN10	5|3	0	0.59	0.8
LPP	3|2	0	0.83	0.8

CUL7	4|3	0	0.74	0.8
TJP2	4|2	0	0.27	0.8
FUS	41|42	0	0.96	0.8
PDHA1	7|7	0	0.9	0.8
LDHB	12|12	0	0.74	0.8
PPP2R2A	11|8	0	0.83	0.8
CBS	4|0	0	0.5	0.8
WDR44	3|2	0	0.62	0.8
WNK1	7|7	0	0.97	0.8
ARHGDIA	0|2	0	0.33	0.8
PFDN6	3|0	0	0.24	0.8
DNM2	16|12	0	0.62	0.8
DDX3X	22|23	0	0.7	0.8
CCT6B	6|0	0	0.33	0.8
CSRP1	2|0	0	0.5	0.8
SRSF2	6|5	0	0.88	0.8
CLINT1	3|5	0	0.7	0.8
DPYSL2	21|19	0	0.88	0.8
ARHGEF12	2|2	0	0.89	0.8
ARPC4	2|2	0	0.53	0.8
RPS17L	4|4	0	0.67	0.8
MDH1	4|3	0	0.82	0.8
USP19	0|3	0	0.38	0.8
UCHL5	3|2	0	0.53	0.8
LRBA	25|18	0	0.83	0.8
GBF1	5|6	0	0.65	0.8
GLRX3	2|3	0	0.45	0.8
PPP5C	0|2	0	0.29	0.8
XIAP	9|4	0	0.7	0.8
BZW1	2|0	0	0.33	0.8
BTF3	7|6	0	0.63	0.8
MTHFD1L	18|20	0	0.61	0.8
ELP2	8|5	0	0.67	0.8
AKT2	7|6	0	0.53	0.8
PPP1CC	4|3	0	0.64	0.8
CLIP1	6|6	0	0.89	0.8
USP15	18|19	0	0.63	0.8
FLII	8|6	0	0.54	0.8
UBE2L3	2|4	0	0.67	0.8
HNRNPH1	17|21	0	0.84	0.8
HARS	8|6	0	0.74	0.8
CLPB	2|3	0	0.83	0.8
C20orf27	3|2	0	0.77	0.8
RDX	15|16	0	0.93	0.8
DCTN2	14|14	0	0.86	0.8

CDK9	4|5	0	0.82	0.8
COPZ1	3|3	0	0.8	0.8
PSMD6	16|18	0	0.94	0.8
RCN2	9|9	0	0.84	0.8
DOCK7	19|21	0	0.93	0.8
QARS	7|6	0	0.52	0.8
CHUK	4|5	0	0.75	0.8
CIRBP	4|5	0	0.82	0.8
TBCB	4|4	0	0.64	0.8
AP2B1	10|10	0	0.67	0.8
CLIC1	2|2	0	0.62	0.8
CLNS1A	19|17	0	0.56	0.8
CSE1L	34|32	0	0.85	0.8
CSTF2	7|8	0	0.88	0.8
DYNC1H1	52|58	0	0.75	0.8
DYNC1I2	5|4	0	0.67	0.8
DOCK1	0|3	0	0.43	0.8
EEF1A1	31|31	0	0.98	0.8
EEF1G	20|18	0	0.77	0.8
EIF4A1	26|26	0	0.87	0.8
EIF4B	27|21	0	0.75	0.8
ENO1	34|32	0	0.81	0.8
FLNA	92|99	0	0.83	0.8
FLNB	70|63	0	0.87	0.8
GDI2	7|9	0	0.58	0.8
GTF2B	5|3	0	0.59	0.8
GTF2I	85|82	0	1.02	0.8
HDAC2	14|13	0	0.93	0.8
HNRNPL	11|12	0	0.61	0.8
DNAJA1	15|17	0	0.88	0.8
IGBP1	2|0	0	0.29	0.8
EIF3E	7|7	0	0.82	0.8
AARS	14|11	0	0.79	0.8
PARP1	30|28	0	0.88	0.8
AK2	3|2	0	0.45	0.8
AKR1B1	0|2	0	0.44	0.8
AMD1	10|10	0	0.93	0.8
ARF4	3|0	0	0.3	0.8
ATP1B3	6|6	0	1	0.8
ATP6V1A	18|17	0	0.82	0.8
ATP6V1B2	14|12	0	0.75	0.8
ATP5PO	7|8	0	0.94	0.8
CAPN2	2|0	0	0.29	0.8
CCT6A	35|33	0	0.84	0.8
CDK1	12|11	0	0.9	0.8

CKB	29|25	0	0.82	0.8
CLTA	3|3	0	0.8	0.8
CNN3	5|5	0	0.56	0.8
DLAT	3|3	0	0.86	0.8
DUT	5|3	0	0.67	0.8
EEF1A2	20|0	0	0.49	0.8
EEF2	29|31	0	0.61	0.8
EIF5	5|6	0	0.81	0.8
FKBP4	28|23	0	0.73	0.8
GAPDH	46|41	0	0.94	0.8
GARS	13|13	0	0.87	0.8
GFPT1	6|6	0	0.59	0.8
HTT	4|3	0	0.88	0.8
HNRNPA1	24|21	0	0.83	0.8
HNRNPD	5|5	0	0.8	0.8
HSPD1	115|89	0	0.9	0.8
HSPE1	5|5	0	0.8	0.8
KPNB1	38|32	0	0.88	0.8
KPNA2	11|11	0	0.75	0.8
KPNA3	6|5	0	0.81	0.8
IPO5	12|10	0	0.51	0.8
LAMC1	3|3	0	0.57	0.8
LBR	5|3	0	0.62	0.8
LGALS3	2|4	0	0.6	0.8
ABLIM1	6|4	0	0.69	0.8
LRPAP1	7|7	0	0.62	0.8
MARCKS	11|9	0	0.83	0.8
MCM3	44|43	0	0.86	0.8
ME1	2|3	0	0.5	0.8
MSN	17|16	0	0.92	0.8
MYH9	107|104	0	0.81	0.8
NASP	55|56	0	1.27	0.8
NCBP1	3|3	0	0.57	0.8
PEBP1	3|3	0	0.71	0.8
PABPC1	12|15	0	0.76	0.8
PAK2	0|3	0	0.4	0.8
PAWR	0|2	0	0.21	0.8
PFDN5	2|0	0	0.21	0.8
PFKL	18|12	0	0.76	0.8
PGAM1	10|12	0	0.72	0.8
PGD	8|7	0	0.86	0.8
PGM1	3|0	0	0.43	0.8
PKM	37|31	0	0.86	0.8
POLA2	7|6	0	0.93	0.8
PPAT	8|7	0	0.86	0.8

PPP1CA	8|7	0	0.94	0.8
PPP1R7	2|0	0	0.36	0.8
PRKCI	0|2	0	0.4	0.8
MAPK6	4|0	0	0.26	0.8
MAP2K1	2|2	0	0.73	0.8
PRPS1	13|9	0	0.68	0.8
PRPS2	12|9	0	0.57	0.8
PRPSAP1	8|5	0	0.37	0.8
PRPSAP2	12|14	0	0.45	0.8
PSMA5	9|9	0	0.84	0.8
PSMB7	6|8	0	0.85	0.8
PSMC1	21|17	0	0.9	0.8
PSMC2	9|10	0	0.54	0.8
PSMC3	13|11	0	0.71	0.8
PSMC5	15|19	0	0.71	0.8
PSMC6	8|6	0	0.72	0.8
PSMD1	15|16	0	0.63	0.8
PSMD2	26|23	0	0.61	0.8
PSMD3	17|17	0	0.77	0.8
PSMD4	7|5	0	0.73	0.8
PSMD7	6|6	0	0.44	0.8
PSMD8	4|3	0	0.47	0.8
PSMD10	2|3	0	0.53	0.8
PSMD11	22|17	0	0.84	0.8
PSMD12	7|8	0	0.55	0.8
PSME2	4|2	0	0.75	0.8
RAD23B	3|2	0	0.67	0.8
RANBP1	4|4	0	0.64	0.8
RARS	11|8	0	0.75	0.8
UPF1	14|12	0	0.88	0.8
ABCE1	2|0	0	0.31	0.8
RPA2	3|2	0	0.83	0.8
RPA3	2|2	0	0.62	0.8
RPL15	3|0	0	0.5	0.8
RPS2	8|8	0	0.7	0.8
SRSF3	16|15	0	0.95	0.8
SGTA	3|3	0	0.75	0.8
FSCN1	2|0	0	0.36	0.8
SNRPE	2|2	0	0.89	0.8
SNX2	5|3	0	0.7	0.8
SPTAN1	24|23	0	0.76	0.8
SPTBN1	14|15	0	0.62	0.8
TRIM21	19|15	0	0.69	0.8
STAT3	4|4	0	0.84	0.8
STRN	3|0	0	0.25	0.8

TFAM	3|3	0	0.67	0.8
GCFC2	2|2	0	1	0.8
TOP1	21|22	0	0.93	0.8
TPM4	8|9	0	0.79	0.8
HSP90B1	108|109	0	1.38	0.8
TTC1	3|3	0	0.6	0.8
DNAJC7	9|6	0	0.57	0.8
TTK	0|3	0	0.35	0.8
TUFM	24|25	0	0.96	0.8
UBA1	36|34	0	0.95	0.8
UQCRC2	2|0	0	0.24	0.8
VBP1	3|0	0	0.27	0.8
VCL	6|8	0	0.64	0.8
YWHAH	8|6	0	0.76	0.8
ZYX	6|6	0	0.77	0.8
USP5	11|7	0	0.77	0.8
TAGLN2	8|5	0	0.58	0.8
EEA1	7|6	0	0.62	0.8
CUL1	7|4	0	0.73	0.8
IRS4	43|37	0	1.03	0.8
RAE1	2|0	0	0.31	0.8
ELP1	26|24	0	0.91	0.8
HAT1	4|4	0	1	0.8
YBX3	11|12	0	0.85	0.8
AGPS	3|6	0	0.67	0.8
AP3B1	28|23	0	0.85	0.8
MAPKAPK5	3|2	0	0.67	0.8
RUVBL1	22|20	0	0.92	0.8
EIF3G	14|12	0	0.68	0.8
IQGAP1	51|41	0	1.08	0.8
GMPS	5|3	0	0.57	0.8
SYNJ1	4|7	0	0.55	0.8
EIF2B5	5|5	0	0.65	0.8
EIF2S2	6|5	0	0.71	0.8
MBD3	4|3	0	0.88	0.8
ST13	9|8	0	0.87	0.8
AP3D1	23|18	0	0.8	0.8
UBE2M	3|3	0	0.86	0.8
BTAF1	5|3	0	0.39	0.8
AIP	3|3	0	0.86	0.8
ATIC	15|14	0	0.77	0.8
ATP5F1A	17|15	0	0.94	0.8
ECHS1	8|7	0	0.83	0.8
FASN	123|115	0	0.77	0.8
DRG1	6|6	0	0.89	0.8

SNRPD3	4|5	0	0.75	0.8
UCHL1	6|6	0	1	0.8
WARS	25|23	0	0.91	0.8
RAB11B	8|8	0	0.89	0.8
ATP6V1F	2|2	0	0.8	0.8
EFTUD2	29|27	0	0.83	0.8
PMPCB	3|2	0	0.71	0.8
BAG2	4|4	0	0.94	0.8
KIF1A	5|3	0	0.53	0.8
CAD	49|49	0	0.73	0.8
COPA	48|49	0	0.96	0.8
DBN1	2|0	0	0.31	0.8
DDX5	15|13	0	0.9	0.8
DSP	14|23	0	0.58	0.8
EPRS	27|26	0	0.87	0.8
FDFT1	4|2	0	0.55	0.8
FRG1	12|11	0	0.87	0.8
HSD17B10	12|14	0	0.87	0.8
HDGF	6|7	0	0.84	0.8
KIF5B	43|37	0	0.81	0.8
KIF5C	12|14	0	0.7	0.8
KIF11	12|9	0	0.82	0.8
MCM2	44|46	0	1.04	0.8
NARS	11|6	0	0.74	0.8
YBX1	12|12	0	0.68	0.8
TTC4	2|3	0	0.56	0.8
BAG6	27|24	0	0.88	0.8
RABEP1	6|4	0	0.77	0.8
ARHGEF1	4|4	0	0.84	0.8
PDCD5	6|6	0	0.73	0.8
HGS	4|5	0	0.75	0.8
SCAF11	7|7	0	0.85	0.8
ZW10	6|6	0	0.77	0.8
DDX21	10|13	0	0.84	0.8
ETF1	8|9	0	0.83	0.8
LRRFIP1	8|8	0	1	0.8
NOLC1	11|9	0	0.73	0.8
COPB2	28|25	0	0.86	0.8
SNAP29	3|5	0	0.53	0.8
TXNL1	0|2	0	0.17	0.8
EIF4E2	6|5	0	0.88	0.8
CLTC	41|40	0	0.94	0.8
FXR2	3|2	0	0.83	0.8
WTAP	4|4	0	1	0.8
ACTN4	83|74	0	1.06	0.8

CAPZB	13|11	0	0.84	0.8
IDE	4|2	0	0.5	0.8
MARS	10|11	0	0.76	0.8
MYO6	7|4	0	0.55	0.8
NME4	3|3	0	0.8	0.8
NUCB2	6|5	0	0.71	0.8
PFN1	10|11	0	0.88	0.8
TPD52	2|3	0	0.71	0.8
NUP214	17|16	0	0.88	0.8
FXR1	7|7	0	0.93	0.8
AKAP12	35|34	0	0.9	0.8
GOLGA5	3|3	0	0.67	0.8
USP14	2|0	0	0.16	0.8
ATP5F1C	2|2	0	0.73	0.8
CRKL	3|2	0	0.83	0.8
DIAPH1	22|21	0	0.82	0.8
CTTN	15|15	0	0.97	0.8
GAK	2|0	0	0.33	0.8
HSPA1B	62|62	0	0.68	0.8
HSPA5	56|54	0	0.99	0.8
NCL	19|17	0	0.95	0.8
PCMT1	3|4	0	0.54	0.8
ROCK1	32|24	0	0.7	0.8
MLF2	2|0	0	0.27	0.8
ZRANB2	17|17	0	0.91	0.8
RANBP9	2|0	0	0.24	0.8
SAE1	12|10	0	0.9	0.8
CFL1	4|4	0	0.73	0.8
DMXL1	4|0	0	0.29	0.8
LGALS3BP	0|2	0	0.4	0.8
RPS14	4|5	0	0.53	0.8
SCAMP3	3|3	0	0.75	0.8
ARPC3	2|0	0	0.29	0.8
ACTR2	5|2	0	0.5	0.8
ACTR1A	8|7	0	0.67	0.8
PDIA6	13|14	0	0.82	0.8
FARP1	8|7	0	0.88	0.8
TXNDC9	2|4	0	0.5	0.8
PSMD14	3|2	0	0.48	0.8
STUB1	12|13	0	0.93	0.8
SMNDC1	2|2	0	0.67	0.8
BCKDK	5|6	0	0.69	0.8
PAK4	9|6	0	0.7	0.8
ACAT2	2|0	0	0.25	0.8
GOLGA3	5|7	0	0.8	0.8

CAPRIN1	5|3	0	0.73	0.8
MAP1B	59|51	0	0.85	0.8
MAT2A	10|10	0	0.85	0.8
MCM7	45|41	0	0.96	0.8
MFAP1	32|32	0	1	0.8
MYH10	113|99	0	0.85	0.8
CCT3	52|51	0	0.77	0.8
TUBA1A	43|43	0	1.06	0.8
MANF	5|5	0	0.95	0.8
RACK1	4|4	0	0.62	0.8
NDC80	0|2	0	0.33	0.8
PRMT5	76|79	0	0.82	0.8
ACAA2	6|7	0	0.84	0.8
TAB1	15|15	0	0.59	0.8
KRT1	53|54	0	1.09	0.8
CAPZA2	8|9	0	0.81	0.8
DNAJB1	4|2	0	0.31	0.8
NUCB1	4|3	0	0.52	0.8
PA2G4	6|7	0	0.72	0.8
PIN1	2|0	0	0.44	0.8
POLR2H	2|2	0	1	0.8
PKN2	3|0	0	0.32	0.8
TLN1	35|30	0	0.7	0.8
VARS	28|28	0	0.79	0.8
AIMP2	3|3	0	0.71	0.8
NPEPPS	3|3	0	0.4	0.8
RAN	10|11	0	0.95	0.8
RBM14	12|14	0	0.91	0.8
COG5	4|2	0	0.75	0.8
ATP5PD	2|0	0	0.29	0.8
CAP2	4|4	0	0.73	0.8
SYNCRIP	9|13	0	0.76	0.8
IPO8	7|8	0	0.65	0.8
IPO7	10|9	0	0.83	0.8
ARFGEF1	0|2	0	0.5	0.8
CCT4	37|33	0	0.65	0.8
CCT2	47|46	0	0.71	0.8
SMC2	25|26	0	0.67	0.8
PRPF8	58|55	0	0.8	0.8
PDLIM5	7|7	0	0.56	0.8
IVNS1ABP	7|7	0	0.41	0.8
PSMC4	16|14	0	0.62	0.8
SARS	6|5	0	0.65	0.8
IGF2BP1	7|5	0	0.67	0.8
IGF2BP3	0|2	0	0.31	0.8

KHDRBS1	12|11	0	0.96	0.8
CCT8	39|36	0	0.79	0.8
POLD3	4|4	0	1	0.8
HSPA8	73|67	0	0.83	0.8
NUDC	18|15	0	0.81	0.8
HSPH1	20|16	0	0.67	0.8
CLPX	10|7	0	0.68	0.8
SUGT1	5|4	0	0.5	0.8
MCM5	51|40	0	1.04	0.8
RBM3	7|6	0	0.87	0.8
YWHAE	35|32	0	0.88	0.8
HNRNPA0	13|12	0	0.93	0.8
TOMM34	8|6	0	0.8	0.8
STIP1	49|41	0	0.79	0.8
YWHAQ	12|9	0	0.68	0.8
GCN1	49|49	0	0.84	0.8
WASF2	3|3	0	0.92	0.8
CDC37	4|5	0	0.67	0.8
TRIO	0|3	0	0.5	0.8
UTRN	6|6	0	0.71	0.8
VCP	78|77	0	0.99	0.8
NUP50	11|11	0	0.85	0.8
STRAP	12|11	0	0.96	0.8
SEC23IP	16|12	0	0.71	0.8
SUPT16H	4|3	0	0.48	0.8
DDX20	2|0	0	0.44	0.8
RPL35	3|2	0	0.77	0.8
DCTN3	2|0	0	0.29	0.8
PNMA2	2|3	0	0.71	0.8
STK38	48|50	0	0.85	0.8
CBX3	3|3	0	0.86	0.8
HSP90AB1	115|97	0	0.92	0.8
DNM1L	16|15	0	0.94	0.8
COMMD3	3|3	0	0.86	0.8
CCT5	39|38	0	0.68	0.8
MACF1	20|19	0	0.97	0.8
CD2AP	27|25	0	0.87	0.8
CHORDC1	4|2	0	0.44	0.8
FKBP8	3|3	0	1	0.8
OGA	11|9	0	0.87	0.8
HSPBP1	6|7	0	0.93	0.8
ACAP2	21|19	0	0.85	0.8
AP2A2	3|3	0	0.57	0.8
KIF4A	6|6	0	0.96	0.8
MAPRE1	8|6	0	0.88	0.8

MYCBP	9|10	0	0.93	0.8
PFAS	13|8	0	0.71	0.8
PFDN2	6|5	0	0.73	0.8
QPCT	20|23	0	0.91	0.8
RSU1	3|0	0	0.19	0.8
SF3B1	58|52	0	1.13	0.8
TIMM13	2|2	0	0.73	0.8
TNPO3	5|4	0	0.62	0.8
YWHAG	11|10	0	0.75	0.8
TBK1	4|2	0	0.75	0.8
VPS51	2|2	0	0.42	0.8
PYCR2	9|10	0	0.86	0.8
OLA1	3|2	0	0.34	0.8
PDCD6IP	8|8	0	0.84	0.8
NRBP1	4|5	0	0.9	0.8
STRN4	2|3	0	0.71	0.8
UBQLN2	5|3	0	0.76	0.8
DHX38	21|16	0	0.86	0.8
SNRNP200	47|41	0	0.83	0.8
MRPS18B	2|0	0	0.27	0.8
C19orf53	5|5	0	0.74	0.8
NOB1	2|0	0	0.29	0.8
FAM32A	5|6	0	0.88	0.8
CRIPT	2|2	0	0.89	0.8
BABAM1	2|2	0	0.62	0.8
PPP2R1A	17|17	0	0.78	0.8
LIMD1	3|3	0	0.86	0.8
HSPA4L	11|9	0	0.82	0.8
DNAJC8	4|5	0	0.9	0.8
NCDN	0|2	0	0.25	0.8
NOMO1	7|0	0	0.5	0.8
SND1	25|26	0	0.8	0.8
CACYBP	8|7	0	0.54	0.8
MAGED2	8|8	0	0.8	0.8
PIK3R4	3|2	0	0.59	0.8
CYFIP1	11|10	0	0.95	0.8
ARHGEF10	2|3	0	0.48	0.8
KNTC1	6|5	0	0.5	0.8
ZFYVE16	5|4	0	0.72	0.8
PCLAF	2|0	0	0.36	0.8
EIF4A3	12|12	0	0.87	0.8
DDX46	102|109	0	1.01	0.8
TBC1D4	6|3	0	0.62	0.8
WASHC5	5|6	0	0.92	0.8
UBAP2L	29|30	0	0.86	0.8

NCAPD2	24|22	0	0.66	0.8
SEC16A	10|7	0	0.51	0.8
PPP6R1	5|3	0	0.64	0.8
TRAPPC8	7|5	0	0.67	0.8
STK38L	30|30	0	0.87	0.8
ZC3H13	0|2	0	0.44	0.8
MRPS27	3|4	0	0.44	0.8
TNRC6B	8|8	0	0.42	0.8
TAB2	3|3	0	0.48	0.8
CLUH	15|13	0	0.74	0.8
BICD2	3|3	0	0.5	0.8
WASHC2C	0|10	0	0.37	0.8
DMXL2	3|0	0	0.4	0.8
DNAJC13	2|0	0	0.11	0.8
WDR7	0|4	0	0.42	0.8
HAUS5	6|3	0	0.72	0.8
USP24	14|11	0	0.85	0.8
LARP1	8|6	0	0.76	0.8
NCAPH	13|16	0	0.87	0.8
NUP188	2|2	0	0.62	0.8
HECTD1	8|6	0	0.23	0.8
GEMIN5	29|28	0	0.97	0.8
PTPN23	3|3	0	0.86	0.8
FAM98A	9|8	0	0.83	0.8
AAR2	3|3	0	0.55	0.8
DST	3|2	0	0.83	0.8
LTN1	5|5	0	0.62	0.8
LSM14A	25|24	0	0.86	0.8
POLDIP2	4|5	0	0.69	0.8
PRPF31	16|13	0	0.76	0.8
GAPVD1	18|15	0	0.92	0.8
DCAF8	10|11	0	0.95	0.8
LMO7	2|0	0	0.36	0.8
HOOK1	9|5	0	0.78	0.8
EIF5B	23|23	0	0.81	0.8
TRMT6	4|5	0	0.9	0.8
MRPS7	0|2	0	0.33	0.8
ATP6V1D	0|2	0	0.44	0.8
MRPS2	0|2	0	0.44	0.8
RTRAF	7|7	0	0.78	0.8
MRPS23	0|2	0	0.21	0.8
COPG1	15|16	0	0.69	0.8
NSFL1C	8|8	0	0.89	0.8
TRAPPC4	3|3	0	0.8	0.8
SDF4	3|4	0	0.78	0.8

JPT1	2|0	0	0.25	0.8
LSM8	2|2	0	0.8	0.8
VPS29	5|3	0	0.73	0.8
ANAPC5	0|3	0	0.46	0.8
SRRM2	45|45	0	1.05	0.8
ANKFY1	29|29	0	0.94	0.8
RTF2	0|2	0	0.4	0.8
CHMP5	2|0	0	0.4	0.8
RSRC1	8|9	0	0.94	0.8
KLHDC4	0|2	0	0.25	0.8
KCTD9	2|0	0	0.29	0.8
CARMIL1	3|2	0	0.83	0.8
VPS50	3|3	0	0.55	0.8
ERCC6L	3|4	0	0.42	0.8
CC2D1A	3|4	0	0.67	0.8
ALKBH5	0|3	0	0.46	0.8
LRRC40	4|4	0	0.94	0.8
GID8	2|2	0	0.67	0.8
PTCD3	3|3	0	0.52	0.8
ZWILCH	2|2	0	0.57	0.8
SCYL2	13|12	0	0.74	0.8
GPATCH1	10|9	0	0.67	0.8
IPO9	6|5	0	0.65	0.8
ELP3	12|8	0	0.77	0.8
ELAC2	4|4	0	0.67	0.8
MRPS10	2|0	0	0.44	0.8
SLC4A1AP	5|4	0	0.67	0.8
VPS35	10|7	0	0.79	0.8
MOB1A	7|6	0	0.74	0.8
TXLNG	3|4	0	0.58	0.8
UBAP2	5|2	0	0.47	0.8
BAIAP2L1	2|3	0	0.53	0.8
RAC1	2|2	0	0.8	0.8
NANS	0|2	0	0.4	0.8
PIMREG	2|0	0	0.4	0.8
PARD3	4|3	0	0.58	0.8
DIABLO	14|15	0	0.95	0.8
GRIPAP1	9|8	0	0.92	0.8
SH3GLB2	2|4	0	0.43	0.8
MRPS22	4|0	0	0.47	0.8
RNPEP	2|0	0	0.36	0.8
KIF15	4|3	0	0.44	0.8
VPS35L	2|3	0	0.67	0.8
PCNP	4|4	0	0.84	0.8
AVEN	0|2	0	0.31	0.8

ANKHD1-EIF4EBP3	7|3	0	0.61	0.8
NUFIP2	7|6	0	0.7	0.8
TAOK1	3|6	0	0.67	0.8
GPHN	5|3	0	0.59	0.8
SLAIN2	7|10	0	0.71	0.8
RELCH	4|3	0	0.56	0.8
CWC22	11|8	0	0.83	0.8
PDLIM1	5|5	0	0.87	0.8
PPM1A	16|18	0	0.94	0.8
MYL6	9|6	0	0.73	0.8
EEF1B2	8|8	0	0.91	0.8
PPIA	9|9	0	0.78	0.8
XRCC5	30|26	0	0.85	0.8
EXOC4	8|5	0	0.65	0.8
MRPS35	2|3	0	0.42	0.8
HSPA2	21|0	0	0.44	0.8
SEC24A	3|4	0	0.78	0.8
EGLN1	2|0	0	0.33	0.8
UBE2O	17|15	0	0.76	0.8
NCAPG	11|13	0	0.8	0.8
MMS19	8|5	0	0.79	0.8
VPS16	3|5	0	0.62	0.8
NUCKS1	7|5	0	0.63	0.8
KLC2	18|19	0	0.95	0.8
VPS33A	2|4	0	0.5	0.8
RSRC2	12|12	0	0.92	0.8
MRPS34	5|3	0	0.55	0.8
DDA1	2|2	0	0.53	0.8
PDCL3	3|2	0	0.45	0.8
ASPSCR1	13|8	0	0.79	0.8
DCTPP1	2|3	0	0.71	0.8
WDR77	21|21	0	0.84	0.8
BRCC3	6|6	0	0.8	0.8
IPO4	5|4	0	0.45	0.8
RABL6	10|9	0	0.83	0.8
MYH14	8|9	0	0.47	0.8
RABEP2	2|3	0	0.83	0.8
MCMBP	4|3	0	0.78	0.8
EDC3	3|3	0	0.92	0.8
NAA50	2|0	0	0.4	0.8
PAAF1	3|2	0	0.71	0.8
WDR61	7|6	0	0.93	0.8
UBXN6	3|3	0	0.41	0.8
DICER1	2|4	0	0.71	0.8
TCP1	43|41	0	0.74	0.8

ANP32E	4|4	0	0.8	0.8
RAB1B	7|8	0	0.88	0.8
HNRNPK	30|28	0	0.94	0.8
SF3B5	2|2	0	0.5	0.8
EPPK1	57|43	0	0.69	0.8
HUWE1	74|70	0	0.89	0.8
RIOK1	18|16	0	0.73	0.8
REPS1	3|2	0	0.59	0.8
PCBP2	9|10	0	0.9	0.8
PTBP1	10|8	0	0.86	0.8
MRPL14	3|3	0	0.92	0.8
THOC3	0|2	0	0.4	0.8
HOOK3	0|2	0	0.27	0.8
MRPS6	2|2	0	0.53	0.8
PPP4R3A	13|11	0	0.92	0.8
HDGFL2	15|15	0	0.98	0.8
SYAP1	7|6	0	0.84	0.8
NUDCD1	2|2	0	0.89	0.8
POLR2C	5|5	0	1	0.8
RECQL	5|0	0	0.36	0.8
DAP3	2|2	0	0.67	0.8
CCDC97	3|3	0	0.92	0.8
RPLP0	7|8	0	0.81	0.8
NAA15	9|8	0	0.57	0.8
PI4KA	10|8	0	0.86	0.8
EXOSC6	4|3	0	0.5	0.8
DNAJB6	3|0	0	0.38	0.8
SYNRG	2|0	0	0.24	0.8
RNPS1	7|6	0	0.53	0.8
ELMO2	5|5	0	0.62	0.8
LRPPRC	87|76	0	0.93	0.8
ASNS	12|11	0	0.73	0.8
CCDC124	8|9	0	0.57	0.8
KCTD12	3|2	0	0.59	0.8
SPINDOC	15|13	0	0.92	0.8
MRRF	2|2	0	0.62	0.8
MAPK1	2|0	0	0.5	0.8
DPP9	2|0	0	0.19	0.8
NAP1L1	10|10	0	0.69	0.8
YWHAB	13|12	0	0.71	0.8
JPT2	9|7	0	0.86	0.8
C15orf40	2|2	0	0.89	0.8
ZC3H18	2|0	0	0.5	0.8
LSM14B	3|4	0	0.78	0.8
ZMAT2	5|5	0	0.95	0.8

SKA1	2|2	0	0.36	0.8
FRA10AC1	5|3	0	0.76	0.8
MAP3K7	11|9	0	0.49	0.8
ATXN2L	13|14	0	0.87	0.8
HMGA1	7|5	0	0.86	0.8
PPIL2	3|0	0	0.43	0.8
SNX6	2|3	0	0.67	0.8
TARS	15|12	0	0.73	0.8
TAB3	3|5	0	0.8	0.8
PROSER2	3|3	0	0.67	0.8
ALDH16A1	3|4	0	0.56	0.8
TPM3	10|8	0	0.6	0.8
SKP1	5|4	0	0.67	0.8
ALDH5A1	4|3	0	0.88	0.8
SELENOH	2|3	0	0.83	0.8
EIF2B4	9|6	0	0.62	0.8
TRAPPC5	2|2	0	0.44	0.8
CCDC50	3|3	0	0.86	0.8
TXLNA	11|9	0	0.87	0.8
CHMP4B	4|4	0	0.94	0.8
PPM1G	16|16	0	0.97	0.8
TUBB	66|64	0	1.29	0.8
EXOC1	4|3	0	0.7	0.8
EXOSC8	3|3	0	0.92	0.8
RFC3	0|2	0	0.5	0.8
OGT	5|4	0	0.36	0.8
ACOT7	6|5	0	0.81	0.8
PCNA	10|9	0	0.79	0.8
MCM4	49|48	0	1.03	0.8
PLCG1	0|3	0	0.32	0.8
WASHC1	3|3	0	0.92	0.8
EIF4G1	39|34	0	0.88	0.8
UBE2I	2|2	0	0.62	0.8
GANAB	63|61	0	1.25	0.8
ACACA	28|22	0	0.68	0.8
NUP43	4|5	0	0.78	0.8
FIBP	0|2	0	0.36	0.8
CARM1	3|4	0	0.82	0.8
BABAM2	0|3	0	0.5	0.8
PLEC	10|8	0	0.9	0.8
E2F7	5|8	0	0.57	0.8
LTBP1	4|5	0	0.4	0.8
AP3M1	9|8	0	0.68	0.8
ATP6V1H	3|3	0	0.8	0.8

Supplementary Table 2: BioID data for TBC1D9 after SAINT analysis

BioID data for TBC1D9-BirA-FLAG in Flp-In T-REx HEK293 cells. Preys with BFDR ≤ 1% are considered high-confidence and shown in Green here. Bait for BioID experiment was BirA-FLAG tagged TBC1D9 protein. Prey Gene is the Official Gene Symbol (from NCBI). Spectral counts for the prey (Spec, separated by "I" delimiter), Averaged probability across replicates (AvgP) Fold Change (counts in the purification divided by counts in the controls plus small factor to prevent division by 0) and Bayesian FDR (BFDR) are listed for each bait-prey relationship and are directly from the SAINTexpress output. 12 controls (cells expressing either BirA-FLAG alone, BirA-FLAG-NLS or BirA-FLAG-GFP) were compressed to 4 to increase the stringency of the SAINT analysis. The experiment was repeated twice.

PreyGene	Spec	AvgP	FoldChange	BFDR
TOR1A	3|5	0.99	40	0
HADHB	5|3	0.99	40	0
FYTTD1	3|4	0.99	35	0
NCKAP5L	4|5	1	45	0
CCDC88C	4|3	0.99	35	0
PLD1	4|5	1	45	0
ACOT13	4|4	1	40	0
ARL13B	10|11	1	105	0
CEP112	4|4	1	40	0
COMMD3-BMI1	4|3	0.99	35	0
CLCN7	18|19	1	10.57	0
GLE1	5|5	1	50	0
PTK7	5|6	1	55	0
SRSF3	3|5	0.99	40	0
HIRA	4|4	1	40	0
AGPS	7|3	0.99	50	0
GPAA1	5|4	1	45	0
GATD3A	4|3	0.99	35	0
DLG5	30|32	1	3.54	0
RUVBL2	30|43	1	5.21	0
U2AF1	8|8	1	80	0
RAB35	7|5	1	60	0
CEP152	24|22	1	18.4	0
SAMM50	6|3	0.99	45	0
AUTS2	3|5	0.99	40	0
RDH11	3|4	0.99	35	0
RAB14	3|4	0.99	35	0
INTS8	4|3	0.99	35	0
LAMTOR1	6|5	1	55	0
SLC30A6	6|7	1	65	0
N4BP2	8|8	1	80	0

UQCC1	3|4	0.99	35	0
SLC38A2	5|5	1	50	0
MRPS22	8|5	1	65	0
NFS1	9|6	1	75	0
SLC25A19	4|5	1	45	0
TRAPPC11	4|3	0.99	35	0
TNS3	8|7	1	75	0
MRPS11	4|5	1	45	0
SLC30A5	4|4	1	40	0
KDELC1	4|3	0.99	35	0
FDXR	4|4	1	40	0
HAUS3	3|4	0.99	35	0
RIOX1	3|4	0.99	35	0
NARS2	4|3	0.99	35	0
SNIP1	5|4	1	45	0
THNSL1	6|5	1	55	0
DGLUCY	5|4	1	45	0
RAB34	3|5	0.99	40	0
CEP192	28|30	1	4.83	0
LZTS2	7|5	1	60	0
GTPBP10	4|4	1	40	0
STK11IP	4|3	0.99	35	0
SRSF10	3|4	0.99	35	0
PIP4P1	4|3	0.99	35	0
SPATA18	5|5	1	50	0
TMED4	6|4	1	50	0
GLI3	3|3	0.97	30	0.01
KIF16B	3|3	0.97	30	0.01
APEX1	3|3	0.97	30	0.01
IDH2	7|7	0.94	7	0.01
NTHL1	3|3	0.97	30	0.01
TMEM11	3|3	0.97	30	0.01
SHMT2	15|13	0.94	3.73	0.01
MAD2L2	3|3	0.97	30	0.01
LATS2	3|3	0.97	30	0.01
TRAPPC8	7|8	0.96	15	0.01
TTLL12	6|2	0.93	40	0.01
UTP11	3|3	0.97	30	0.01
MCUB	3|3	0.97	30	0.01
AGK	7|8	0.96	15	0.01
GTPBP2	2|5	0.93	35	0.01
ABHD16A	3|3	0.97	30	0.01
NAA16	9|10	0.94	4.75	0.01
MAP3K21	5|2	0.93	35	0.01
HNRNPLL	13|13	0.97	4	0.01

NNT	21|18	0.95	4.11	0.01
NPC1	2|3	0.92	25	0.02
ALDH2	4|2	0.93	30	0.02
AGPAT3	2|3	0.92	25	0.02
MZT2A	2|3	0.92	25	0.02
LRRC8A	3|2	0.92	25	0.02
PHLDB2	3|2	0.92	25	0.02
SPATA2	2|3	0.92	25	0.02
SLC6A8	3|2	0.92	25	0.02
MYO19	2|3	0.92	25	0.02
PEX3	2|3	0.92	25	0.02
NIPSNAP1	3|2	0.92	25	0.02
NRP1	3|2	0.92	25	0.02
H1F0	2|3	0.92	25	0.02
LPCAT3	3|2	0.92	25	0.02
MICU1	3|2	0.92	25	0.02
MOGS	3|2	0.92	25	0.02
PIBF1	2|3	0.92	25	0.02
VAT1	3|2	0.92	25	0.02
CAMKK2	3|2	0.92	25	0.02
HAUS5	3|2	0.92	25	0.02
TMEM87A	3|2	0.92	25	0.02
FAHD2A	3|2	0.92	25	0.02
RSL24D1	2|3	0.92	25	0.02
EMC4	4|2	0.93	30	0.02
PARL	2|3	0.92	25	0.02
MARK1	2|4	0.93	30	0.02
CCSER2	3|2	0.92	25	0.02
APMAP	3|2	0.92	25	0.02
NDUFV2	2|3	0.92	25	0.02
LAMTOR3	4|2	0.93	30	0.02
OSGEPL1	2|4	0.93	30	0.02
PAPOLG	2|3	0.92	25	0.02
ESRP2	4|2	0.93	30	0.02
SGPP1	2|3	0.92	25	0.02
CNOT6L	2|3	0.92	25	0.02
APTX	3|2	0.92	25	0.02
MRPS9	3|2	0.92	25	0.02
FAM45A	2|3	0.92	25	0.02
DPY19L3	3|2	0.92	25	0.02
SLC29A1	2|2	0.86	20	0.04
PLEKHM3	2|2	0.86	20	0.04
GOLGA3	18|17	0.91	3.33	0.04
TRAPPC3	2|2	0.86	20	0.04
NDUFA2	2|2	0.86	20	0.04

TXN	2|2	0.86	20	0.04
TRIP11	33|42	0.9	4.17	0.04
LATS1	2|2	0.86	20	0.04
AP4M1	2|2	0.86	20	0.04
RAB8A	9|10	0.88	4.22	0.04
LMAN1	6|6	0.9	12	0.04
TMED2	2|2	0.86	20	0.04
ATP5F1E	2|2	0.86	20	0.04
PYCR1	12|14	0.91	3.71	0.04
PRDX3	2|2	0.86	20	0.04
HSD17B11	2|2	0.86	20	0.04
FAM120C	2|2	0.86	20	0.04
TMCC3	2|2	0.86	20	0.04
ARHGAP31	2|2	0.86	20	0.04
NDUFV3	6|6	0.9	12	0.04
SLC5A6	2|2	0.86	20	0.04
PLEKHG2	2|2	0.86	20	0.04
SECISBP2	2|2	0.86	20	0.04
TMX1	2|2	0.86	20	0.04
MRPL9	2|2	0.86	20	0.04
RAB2B	2|2	0.86	20	0.04
NFXL1	2|2	0.86	20	0.04
RAB12	7|7	0.8	9.33	0.06
AMOT	11|12	0.8	5.75	0.06
RAB13	7|7	0.8	9.33	0.06
PMPCB	5|5	0.81	10	0.06
NDUFV1	9|9	0.8	9	0.06
PRPF38B	6|6	0.83	6	0.06
UGGT1	8|4	0.82	12	0.06
GRPEL1	5|5	0.81	10	0.06
DDX51	6|5	0.85	11	0.06
PLEKHG1	8|7	0.79	6	0.07
ORC4	8|9	0.75	3.78	0.07
GAPDH	22|16	0.69	3.3	0.07
CBR1	4|6	0.78	10	0.07
SGPL1	8|7	0.79	6	0.07
RAB7A	5|4	0.73	9	0.07
SIPA1L3	16|12	0.76	3.5	0.07
SIPA1L1	11|11	0.76	3.38	0.07
MRPS2	9|7	0.69	4	0.07
ALDH1B1	12|9	0.67	3.5	0.08
RCC1	9|10	0.68	4.22	0.08
NDUFS1	17|12	0.66	3.41	0.08
ATP5PB	7|4	0.66	5.5	0.08
SHPK	4|4	0.66	8	0.08

DAP3	4|4	0.66	8	0.08
NSA2	5|6	0.66	7.33	0.09
MAD1L1	5|3	0.63	8	0.09
ERAL1	5|3	0.63	8	0.09
ERLIN1	6|5	0.66	7.33	0.09
MOXD1	5|3	0.63	8	0.09
THEM6	7|6	0.65	5.2	0.09
CEP97	5|3	0.63	8	0.09
NGDN	7|8	0.62	3.75	0.1
FLAD1	4|3	0.56	7	0.1
PTPN9	6|4	0.61	5	0.1
TRAPPC10	4|3	0.56	7	0.1
WDR46	3|4	0.56	7	0.1
SMC5	11|18	0.56	3.41	0.1
RPGRIP1L	4|3	0.56	7	0.1
NUDT19	4|5	0.51	4.5	0.11
MRPS28	4|5	0.51	4.5	0.11
ADPRHL2	7|6	0.51	4.33	0.11
CNTROB	4|5	0.51	4.5	0.11
BCKDHA	5|0	0.5	25	0.12
CSNK1G2	0|5	0.5	25	0.12
NDUFS6	5|0	0.5	25	0.12
ARL8B	0|5	0.5	25	0.12
RPS25	4|0	0.5	20	0.13
EIF5AL1	4|0	0.5	20	0.13
RAB3D	4|0	0.5	20	0.13
ARHGEF6	0|4	0.5	20	0.13
IDH3A	4|0	0.5	20	0.13
ITPRIP	0|4	0.5	20	0.13
GPT2	0|4	0.5	20	0.13
FMNL3	0|4	0.5	20	0.13
SP3	0|3	0.49	15	0.15
SLC12A4	3|0	0.49	15	0.15
CORO7-PAM16	0|3	0.49	15	0.15
CAMK2B	6|6	0.49	4.8	0.15
NSUN4	3|0	0.49	15	0.15
CDK5RAP2	0|3	0.49	15	0.15
FAU	3|0	0.49	15	0.15
NDUFB10	3|0	0.49	15	0.15
EIF1AY	3|0	0.49	15	0.15
NR2F1	0|3	0.49	15	0.15
GNAI3	3|0	0.49	15	0.15
TMED1	3|0	0.49	15	0.15
ATP5ME	0|3	0.49	15	0.15
RBM15B	3|0	0.49	15	0.15

GMPPA	3|0	0.49	15	0.15
MRPL15	3|0	0.49	15	0.15
ADNP2	3|0	0.49	15	0.15
ADCY6	0|3	0.49	15	0.15
AIG1	3|0	0.49	15	0.15
CTDSPL2	3|0	0.49	15	0.15
RAB39A	0|3	0.49	15	0.15
ARHGEF40	0|3	0.49	15	0.15
MRPS25	3|0	0.49	15	0.15
RNF219	0|3	0.49	15	0.15
PPCS	3|0	0.49	15	0.15
CANT1	3|0	0.49	15	0.15
ARL8A	3|0	0.49	15	0.15
MSI2	3|0	0.49	15	0.15
PDHA1	5|6	0.49	4.4	0.19
RAB9A	5|6	0.49	4.4	0.19
TFRC	16|19	0.49	2.69	0.2
PTP4A1	3|3	0.46	6	0.2
IDH3G	4|4	0.46	5.33	0.2
NRM	4|4	0.46	5.33	0.2
MTF2	3|5	0.46	5.33	0.2
DROSHA	3|3	0.46	6	0.2
VPS13D	3|3	0.46	6	0.2
CCDC93	3|3	0.46	6	0.2
VKORC1L1	3|3	0.46	6	0.2
PGK1	8|5	0.44	3.25	0.21
TGFBI	0|2	0.43	10	0.21
RHBDD2	0|2	0.43	10	0.21
PRKD2	2|0	0.43	10	0.21
TMEM223	0|2	0.43	10	0.21
UQCC3	2|0	0.43	10	0.21
MEX3A	2|0	0.43	10	0.21
SYK	2|0	0.43	10	0.21
PIP5K1A	0|2	0.43	10	0.21
SEC13	2|0	0.43	10	0.21
FAM71E2	0|2	0.43	10	0.21
NOS1AP	0|2	0.43	10	0.21
CEP120	0|2	0.43	10	0.21
TIMM17B	0|2	0.43	10	0.21
TUT7	2|4	0.46	6	0.21
ATP7B	2|0	0.43	10	0.21
LETMD1	0|2	0.43	10	0.21
GMDS	2|0	0.43	10	0.21
TMEM161A	2|0	0.43	10	0.21
DOCK1	2|6	0.44	4	0.21

COX7A2	0|2	0.43	10	0.21
MTIF2	0|2	0.43	10	0.21
PRPSAP1	2|0	0.43	10	0.21
FSCN1	2|0	0.43	10	0.21
TIAL1	2|0	0.43	10	0.21
SEC62	2|0	0.43	10	0.21
UGCG	0|2	0.43	10	0.21
PKMYT1	0|2	0.43	10	0.21
RUNX3	2|0	0.43	10	0.21
XPR1	0|2	0.43	10	0.21
STX8	0|2	0.43	10	0.21
POU3F2	2|0	0.43	10	0.21
LAGE3	0|2	0.43	10	0.21
EXOC5	0|2	0.43	10	0.21
YWHAQ	0|2	0.43	10	0.21
DUSP12	0|2	0.43	10	0.21
ICMT	0|2	0.43	10	0.21
SGK3	2|0	0.43	10	0.21
SNX8	2|0	0.43	10	0.21
LAMTOR2	2|0	0.43	10	0.21
WDR37	2|0	0.43	10	0.21
TIMM21	2|0	0.43	10	0.21
TOR1B	2|0	0.43	10	0.21
MRPL19	2|0	0.43	10	0.21
DPY19L1	0|2	0.43	10	0.21
YIPF3	2|0	0.43	10	0.21
TMEM186	0|2	0.43	10	0.21
FCF1	2|0	0.43	10	0.21
MPP6	2|0	0.43	10	0.21
MRPL27	2|0	0.43	10	0.21
WDR13	0|2	0.43	10	0.21
MRPS10	0|2	0.43	10	0.21
EMC7	0|2	0.43	10	0.21
DUS3L	2|0	0.43	10	0.21
CNNM4	2|0	0.43	10	0.21
KIF15	2|0	0.43	10	0.21
SLC12A5	0|2	0.43	10	0.21
DENND1A	2|0	0.43	10	0.21
MRPL17	2|0	0.43	10	0.21
MRPS14	2|0	0.43	10	0.21
RAB38	0|2	0.43	10	0.21
ATPAF1	0|2	0.43	10	0.21
ACTR8	2|0	0.43	10	0.21
SLC26A6	2|0	0.43	10	0.21
MRPL44	0|2	0.43	10	0.21

ALG12	0|2	0.43	10	0.21
MRPL24	2|0	0.43	10	0.21
COLGALT1	11|10	0.45	3	0.21
HPS6	2|0	0.43	10	0.21
TTC21B	2|0	0.43	10	0.21
NAA40	2|0	0.43	10	0.21
CSPP1	0|2	0.43	10	0.21
SCD5	0|2	0.43	10	0.21
OPA3	2|0	0.43	10	0.21
EEPD1	2|0	0.43	10	0.21
SLC38A1	2|0	0.43	10	0.21
MRPS26	2|0	0.43	10	0.21
ISCA1	2|0	0.43	10	0.21
LRRC8C	2|0	0.43	10	0.21
LLPH	2|0	0.43	10	0.21
C7orf50	0|2	0.43	10	0.21
ARHGAP18	0|2	0.43	10	0.21
SLC12A6	0|2	0.43	10	0.21
USP54	0|2	0.43	10	0.21
DCP2	2|0	0.43	10	0.21
STON1-GTF2A1L	0|2	0.43	10	0.21
MPP7	2|0	0.43	10	0.21
NDUFA11	2|0	0.43	10	0.21
LEMD2	2|0	0.43	10	0.21
CERS1	0|2	0.43	10	0.21
NHS	0|2	0.43	10	0.21
CHMP2A	2|0	0.43	10	0.21
UBE3D	2|0	0.43	10	0.21
MBNL1	0|2	0.43	10	0.21
CDC7	3|4	0.39	4.67	0.31
PREB	3|4	0.39	4.67	0.31
WDR7	4|3	0.39	4.67	0.31
CGN	3|4	0.39	4.67	0.31
MOV10	8|9	0.43	3.09	0.31
USP30	5|0	0.4	5	0.31
SPICE1	9|8	0.43	3.09	0.31
SASS6	4|3	0.39	4.67	0.31
RPSA	4|4	0.38	4	0.32
COG6	3|2	0.36	5	0.32
ARMC10	3|2	0.36	5	0.32
SURF1	3|2	0.36	5	0.32
NBR1	3|2	0.36	5	0.32
SLC25A17	4|4	0.38	4	0.32
GNA13	9|9	0.38	3	0.32
PGRMC1	2|3	0.36	5	0.32

SDF2	3|2	0.36	5	0.32
COMMD3	3|2	0.36	5	0.32
NDUFA12	3|2	0.36	5	0.32
ARMT1	6|4	0.38	4	0.32
TAB3	2|3	0.36	5	0.32
RC3H1	2|5	0.39	4.67	0.32
RRBP1	6|10	0.34	2.67	0.33
PIK3R2	3|3	0.32	4	0.33
PLK1	18|15	0.32	2.64	0.33
MED16	3|3	0.32	4	0.33
TBK1	11|19	0.35	2.31	0.33
PPP1R13B	8|5	0.34	2.89	0.33
PIGT	10|10	0.35	3.33	0.33
NIN	3|3	0.32	4	0.33
FAM83H	3|3	0.32	4	0.33
MIA3	5|5	0.33	3.33	0.33
DOCK4	2|6	0.3	3.2	0.34
DDHD2	15|13	0.27	2.67	0.34
SLC6A15	6|5	0.31	3.14	0.34
CEP85	10|11	0.29	2.8	0.34
COQ8B	3|4	0.28	3.5	0.34
FMNL2	5|0	0.31	2.5	0.34
PTPRF	6|0	0.29	3	0.34
TMEM214	7|6	0.25	2.89	0.35
PTBP3	11|13	0.25	2.67	0.35
SNX3	2|2	0.25	4	0.35
MPDU1	2|2	0.25	4	0.35
ATP5MPL	2|2	0.25	4	0.35
RPL35	7|6	0.25	2.89	0.35
EFR3B	2|2	0.25	4	0.35
EFR3A	2|2	0.25	4	0.35
NKIRAS2	2|2	0.25	4	0.35
EPC1	2|2	0.25	4	0.35
RTTN	2|2	0.25	4	0.35
TAP2	5|3	0.24	3.2	0.36
IQSEC1	2|3	0.24	3.33	0.36
ATL2	5|3	0.24	3.2	0.36
TRA2B	3|2	0.24	3.33	0.36
CDC20	2|3	0.24	3.33	0.36
HMGA2	3|2	0.24	3.33	0.36
PNKP	3|2	0.24	3.33	0.36
HOOK1	3|2	0.24	3.33	0.36
LRRC47	2|3	0.24	3.33	0.36
RELA	3|2	0.24	3.33	0.36
SLC25A21	3|5	0.24	3.2	0.36

NUP35	3|2	0.24	3.33	0.36
YIF1B	3|0	0.23	3	0.37
IKBKB	3|0	0.23	3	0.37
XPO6	0|4	0.23	2.67	0.37
PIN4	3|0	0.23	3	0.37
MAU2	0|3	0.23	3	0.37
VPS36	3|0	0.23	3	0.37
ZCCHC17	0|3	0.23	3	0.37
UHRF1BP1	6|7	0.23	2.89	0.37
RMND1	3|0	0.23	3	0.37
SAMD4B	0|3	0.23	3	0.37
ITCH	8|7	0.23	3	0.37
PARS2	0|4	0.23	2.67	0.37
RBM45	0|3	0.23	3	0.37
PLEKHA7	4|2	0.23	3	0.37
EPB41L1	7|5	0.24	3	0.37
WNK3	6|5	0.23	3.14	0.38
TMED5	3|3	0.23	3	0.38
HIP1	6|2	0.22	2.29	0.39
HARS2	5|5	0.18	2.86	0.39
ERGIC2	5|4	0.19	3	0.39
DARS2	21|21	0.2	2.8	0.39
SDR39U1	4|2	0.22	3	0.39
RALGAPB	9|5	0.19	2.33	0.39
PTBP2	8|0	0.18	1.6	0.39
RRAGD	0|4	0.19	2	0.39
SPCS3	2|2	0.17	2.67	0.39
RRAGC	5|0	0.2	2	0.39
WDCP	12|14	0.22	2.6	0.39
POTEE	0|15	0.17	1.76	0.4
SENP1	5|5	0.16	2.86	0.4
DBT	3|0	0.16	2	0.4
GSK3B	7|7	0.17	2.8	0.4
ACTC1	31|29	0.16	3.33	0.4
RPS27L	6|7	0.16	2.89	0.4
OTUB1	3|6	0.16	2.57	0.4
C9orf64	2|3	0.16	2.5	0.4
COQ6	2|3	0.16	2.5	0.4
PHC2	3|2	0.16	2.5	0.4
FAM83B	13|10	0.14	2.42	0.41
VDAC3	13|14	0.14	2.57	0.41
OSBP	5|3	0.15	2.67	0.41
RAB11B	4|3	0.16	2.8	0.41
PDIA3	8|0	0.14	1.45	0.41
PNPLA6	3|4	0.16	2.8	0.41

SACS	3|3	0.14	2.4	0.41
COG4	5|2	0.14	2.33	0.41
DONSON	4|4	0.13	2.67	0.41
NXN	4|4	0.13	2.67	0.41
SOGA1	4|4	0.13	2.67	0.41
GPAT4	4|4	0.13	2.67	0.41
PSEN2	0|2	0.13	2	0.42
GTF2H2C_2	0|2	0.13	2	0.42
CELF1	0|2	0.13	2	0.42
GCLC	0|2	0.13	2	0.42
TBC1D23	0|2	0.13	2	0.42
C20orf27	2|0	0.13	2	0.42
EYA4	2|0	0.13	2	0.42
CEPT1	0|2	0.13	2	0.42
SIRT2	0|2	0.13	2	0.42
PISD	0|2	0.13	2	0.42
IFT172	0|2	0.13	2	0.42
TBC1D8B	2|0	0.13	2	0.42
USE1	0|2	0.13	2	0.42
TMX3	0|2	0.13	2	0.42
DTNB	2|0	0.13	2	0.42
NDUFS7	0|2	0.13	2	0.42
NAA50	2|0	0.13	2	0.42
YIPF5	2|0	0.13	2	0.42
ZCCHC3	2|0	0.13	2	0.42
ZCRB1	0|2	0.13	2	0.42
PIGU	2|0	0.13	2	0.42
TMEM41A	2|0	0.13	2	0.42
PGM2L1	0|2	0.13	2	0.42
GPR180	0|2	0.13	2	0.42
ITPR1	4|5	0.11	2.57	0.44
ARVCF	4|5	0.11	2.57	0.44
RALY	16|16	0.11	2.56	0.44
GSK3A	11|12	0.12	2.56	0.44
MRPL47	4|4	0.13	2.67	0.44
ATP13A1	6|6	0.13	2.67	0.44
ABCA3	2|3	0.11	2.5	0.45
MBOAT7	2|2	0.1	2	0.45
ENO3	3|0	0.11	1.5	0.45
MTX2	2|3	0.11	2.5	0.45
PMVK	3|0	0.11	1.5	0.45
SMG5	3|5	0.1	2.29	0.45
CHCHD3	2|2	0.1	2	0.45
ISG20L2	2|3	0.11	2.5	0.45
JAGN1	3|2	0.11	2.5	0.45

LMNB2	8|8	0.1	2.67	0.45
RAB39B	0|3	0.11	1.5	0.45
ATM	2|3	0.09	2	0.46
PHKA2	2|0	0.08	1.33	0.46
PPT1	0|2	0.08	1.33	0.46
IQCB1	4|3	0.08	2.33	0.46
CDK6	4|3	0.09	2.33	0.46
RPS17L	4|3	0.08	2.33	0.46
DHCR7	3|3	0.09	2.4	0.46
GNB1	3|3	0.09	2.4	0.46
ARHGAP29	2|0	0.08	1.33	0.46
TMEM97	2|0	0.08	1.33	0.46
DDX28	2|0	0.08	1.33	0.46
TAMM41	0|2	0.08	1.33	0.46
NCKIPSD	0|2	0.08	1.33	0.46
EIF2AK4	3|0	0.08	1.5	0.47
LYAR	2|5	0.08	2	0.47
SLC33A1	5|2	0.08	2	0.47
SRSF9	8|6	0.08	2.33	0.47
BNIP2	3|0	0.08	1.5	0.47
MYCBP2	8|4	0.08	2	0.47
PDXP	6|8	0.08	2.33	0.47
SIPA1L2	8|9	0.07	2.43	0.47
SHROOM3	0|3	0.08	1.5	0.47
ALG2	3|0	0.08	1.5	0.47
APRT	7|6	0.05	2.36	0.48
AHCY	3|0	0.07	1.2	0.48
RPL29	2|2	0.05	2	0.48
SLC37A4	2|2	0.05	2	0.48
MARF1	5|4	0.06	2.25	0.48
PPP2CA	2|2	0.05	2	0.48
RBM4	4|4	0.07	2.29	0.48
NDUFA5	3|2	0.06	2	0.48
ADRM1	2|2	0.05	2	0.48
TONSL	4|0	0.07	1.33	0.48
KBTBD11	2|2	0.05	2	0.48
HECTD4	5|0	0.05	1.25	0.49
ANXA7	2|0	0.05	1	0.49
DFFB	0|2	0.05	1	0.49
PLXNB2	0|2	0.05	1	0.49
STK38L	2|0	0.05	1	0.49
MRPS27	5|2	0.05	1.75	0.49
VAC14	5|3	0.05	2	0.49
FASTKD5	4|7	0.05	2	0.49
CAMKK1	2|0	0.05	1	0.49

XXYLT1	0|2	0.05	1	0.49
TEX9	2|2	0.05	1.6	0.49
TAP1	0|3	0.05	1.2	0.5
PGP	5|6	0.04	2.2	0.5
GTF2H4	5|5	0.04	2.22	0.5
PKP4	10|11	0.04	2.33	0.5
YBX3	0|5	0.04	1.25	0.5
RAB1A	9|12	0.04	2.33	0.5
WRB	4|3	0.04	2	0.5
RERE	0|3	0.05	1.2	0.5
EPM2AIP1	3|4	0.04	2	0.5
NF2	3|3	0.05	2	0.5
TM9SF3	3|3	0.05	2	0.5
MRPS12	3|3	0.04	2	0.5
ERCC3	8|8	0.03	2.29	0.51
HOXD13	2|4	0.04	1.71	0.51
PRMT3	4|2	0.03	1.71	0.51
SLC25A12	6|6	0.03	2.18	0.51
SEC24C	11|7	0.03	2	0.51
TRAM1	4|4	0.03	2	0.51
BRD1	6|4	0.03	2	0.51
EMC1	18|15	0.03	2.28	0.51
MRPS35	6|4	0.04	2	0.51
DHX35	6|4	0.03	2	0.51
NUP210	18|19	0.03	2.39	0.51
GNAS	9|7	0.03	2.13	0.51
HMGA1	0|4	0.03	1.14	0.51
WEE1	2|0	0.03	1	0.52
CHEK1	2|0	0.03	1	0.52
ATN1	2|0	0.03	1	0.52
MRPL12	2|0	0.03	1	0.52
PRKCH	2|0	0.03	1	0.52
ACOT2	8|9	0.03	2.27	0.52
ZNF346	5|4	0.03	2	0.52
MRPL22	2|0	0.03	1	0.52
URB1	9|4	0.03	1.73	0.52
TTC27	2|0	0.03	1	0.52
COG1	13|16	0.03	2.23	0.52
GIGYF1	5|4	0.03	2	0.52
DDX31	0|2	0.03	1	0.52
NLRX1	2|0	0.03	1	0.52
RPAP2	2|2	0.03	1.6	0.52
GRWD1	0|2	0.03	1	0.52
TMEM126A	2|2	0.03	1.6	0.52
TP53RK	7|6	0.03	2.17	0.52

PFN2	2|0	0.03	1	0.52
MBOAT2	2|0	0.03	1	0.52
NUDCD2	2|2	0.03	1.6	0.52
MCAT	0|2	0.03	1	0.52
ASPH	4|4	0.03	2	0.53
WASHC5	4|4	0.03	2	0.53
DCAF13	4|4	0.03	2	0.53
RASAL2	0|3	0.02	1	0.53
AP4E1	3|3	0.02	1.71	0.54
PSMA5	3|2	0.02	1.67	0.54
SMARCD1	5|6	0.02	2	0.54
SUPT4H1	2|3	0.02	1.67	0.54
TPP2	2|0	0.02	0.8	0.54
RER1	5|5	0.02	2	0.54
NUPL2	3|5	0.02	1.78	0.54
STAT5B	2|3	0.02	1.67	0.54
RAB21	9|9	0.02	2.25	0.54
EMC3	7|7	0.02	2.15	0.54
LRRC59	5|5	0.02	2	0.54
VARS2	5|0	0.02	1.11	0.54
GFM1	6|5	0.02	2	0.54
PTDSS2	5|6	0.02	2	0.54
AES	3|3	0.02	1.71	0.54
PPIB	2|0	0.01	0.8	0.55
ESCO2	0|2	0.01	0.8	0.55
SHPRH	2|0	0.01	0.8	0.55
TSC2	3|2	0.02	1.43	0.55
OTUD4	6|4	0.02	1.82	0.55
LSR	0|2	0.01	0.8	0.55
GTF2H3	2|0	0.01	0.8	0.55
KIAA0355	8|8	0.02	2.13	0.55
GPD1L	2|0	0.01	0.8	0.55
DMXL2	2|0	0.01	0.8	0.55
TFB1M	4|2	0.01	1.5	0.55
JPH1	3|0	0.02	1	0.55
PARP12	3|4	0.02	1.75	0.55
NAA35	3|2	0.01	1.43	0.55
ZYG11B	2|0	0.01	0.8	0.55
FAHD1	3|4	0.02	1.75	0.55
SESN2	0|2	0.01	0.8	0.55
LRCH3	0|2	0.01	0.8	0.55
MYL12B	2|0	0.01	0.8	0.55
ELMO2	14|13	0.01	2.25	0.55
UBE4A	2|2	0.01	1.33	0.56
KIF20A	6|7	0.01	2	0.56

TBL2	7|6	0.01	2	0.56
SLC25A15	6|6	0.01	2	0.56
TBKBP1	4|0	0.01	1	0.56
FARP2	5|4	0.01	1.8	0.56
AKAP11	2|2	0.01	1.33	0.56
TFB2M	2|2	0.01	1.33	0.56
GPAT3	4|0	0.01	1	0.56
ERCC2	3|2	0.01	1.43	0.57
DHFR	5|4	0.01	1.8	0.57
AGO2	2|0	0.01	0.67	0.57
NDUFA6	4|4	0.01	1.78	0.57
PLCG2	3|2	0.01	1.43	0.57
SCAMP1	4|4	0.01	1.78	0.57
DNAJC3	7|5	0.01	1.85	0.57
TRA2A	7|5	0.01	1.85	0.57
ZSWIM8	9|9	0.01	2.12	0.57
GGA2	3|0	0.01	0.86	0.57
QRICH1	3|2	0.01	1.43	0.57
HS1BP3	3|2	0.01	1.43	0.57
NUP85	7|5	0.01	1.85	0.57
KLHL15	3|0	0.01	0.86	0.57
MAGT1	5|3	0.01	1.6	0.57
MEN1	8|7	0.01	2	0.58
NSDHL	4|3	0.01	1.56	0.58
NDUFS2	8|6	0.01	1.87	0.58
PRPSAP2	5|5	0.01	1.82	0.58
SUCLG2	6|4	0.01	1.67	0.58
EIF2B2	8|6	0.01	1.87	0.58
HSD17B7	2|2	0.01	1.33	0.58
TMED9	2|2	0.01	1.33	0.58
NDC1	6|4	0.01	1.67	0.58
MRM3	4|2	0.01	1.33	0.58
IMP3	0|3	0.01	0.86	0.58
DHX33	5|6	0.01	1.83	0.58
C17orf75	2|2	0.01	1.33	0.58
LCLAT1	5|2	0.01	1.4	0.58
NME7	5|2	0.01	1.4	0.58
CRTC3	0|2	0.01	0.67	0.59
UBE4B	0|2	0.01	0.67	0.59
MTO1	2|0	0.01	0.67	0.59
SUN2	2|0	0.01	0.67	0.59
ACADSB	13|9	0.01	1.91	0.59
MYO1E	5|4	0.01	1.64	0.59
SNU13	3|3	0.01	1.5	0.59
MED12	3|0	0.01	0.75	0.59

KRR1	9|4	0.01	1.53	0.59
PLD3	2|2	0.01	1.14	0.59
SZT2	2|2	0.01	1.14	0.59
PUM2	2|0	0.01	0.67	0.59
BIRC6	8|8	0.01	2	0.59
MRPS34	4|4	0.01	1.6	0.59
SPATA5L1	2|0	0.01	0.67	0.59
CARS2	5|4	0.01	1.64	0.59
FIBP	0|4	0.01	0.89	0.59
RPS28	3|2	0	1.25	0.6
MPC2	5|6	0	1.69	0.6
DCAF1	3|2	0	1.25	0.6
OXA1L	4|5	0.01	1.64	0.6
FKBP8	3|2	0	1.25	0.6
LDLRAP1	2|0	0	0.67	0.6
NBAS	5|7	0.01	1.71	0.6
UFM1	2|0	0	0.67	0.6
MRPL16	8|7	0	1.88	0.6
MOB1A	4|4	0	1.6	0.6
AGPAT4	0|2	0	0.67	0.6
SLC25A22	4|5	0.01	1.64	0.6
INTS5	5|6	0	1.69	0.6
MRPS15	4|4	0	1.6	0.6
UTP15	4|5	0.01	1.64	0.6
MRPS6	3|2	0	1.25	0.6
NAA15	9|5	0	1.65	0.6
DYNLL2	4|4	0	1.6	0.6
PRRC1	2|0	0	0.67	0.6
CYB5R3	10|11	0	2	0.61
HARS	0|3	0	0.67	0.61
DLST	6|8	0	1.75	0.61
SRM	3|3	0	1.33	0.61
AP1G2	4|3	0	1.4	0.61
HERC2	8|0	0	1	0.61
AKAP9	2|0	0	0.57	0.61
DENND4A	3|3	0	1.33	0.61
ERLIN2	8|5	0	1.62	0.61
CNOT3	10|10	0	2	0.61
USP34	0|2	0	0.57	0.61
ANKRD26	5|5	0	1.67	0.61
THUMPD3	4|3	0	1.4	0.61
LUC7L2	6|3	0	1.38	0.61
CEP41	3|3	0	1.33	0.61
ANXA11	4|3	0	1.4	0.61
LUC7L	4|3	0	1.4	0.61

RBM12B	8|2	0	1.25	0.61
PLOD1	14|10	0	1.85	0.62
BTBD11	0|2	0	0.57	0.62
CEP44	4|4	0	1.45	0.62
ATL1	0|2	0	0.57	0.62
LRCH1	6|6	0	1.71	0.62
TRRAP	19|18	0	2.18	0.62
FHL1	2|0	0	0.57	0.62
ITPR2	7|0	0	0.93	0.62
GOLGA2	4|5	0	1.5	0.62
PRKD3	2|0	0	0.57	0.62
SPTBN2	4|0	0	0.8	0.62
TRIP4	2|0	0	0.57	0.62
INTS10	2|0	0	0.57	0.62
RHOT1	4|4	0	1.45	0.62
ALG1	4|5	0	1.5	0.62
NAT14	3|3	0	1.33	0.62
SNTB1	10|9	0	1.9	0.62
ERAP2	5|4	0	1.5	0.62
RBM42	2|0	0	0.57	0.62
C10orf88	0|2	0	0.57	0.62
TTI2	0|2	0	0.57	0.62
RNF214	2|2	0	1	0.62
KAT6A	3|2	0	1.11	0.63
RTCA	6|5	0	1.57	0.63
TMEM201	3|2	0	1.11	0.63
TSC1	5|10	0	1.5	0.63
SEC11A	2|2	0	1	0.63
MSI1	2|3	0	1.11	0.63
OFD1	17|17	0	2.12	0.63
ABCC1	5|2	0	1.17	0.63
NDUFA9	7|8	0	1.76	0.63
ARFGEF1	3|5	0	1.33	0.63
LSM4	2|3	0	1.11	0.63
CEP131	9|9	0	1.89	0.63
FAM98A	8|10	0	1.8	0.63
MED23	5|6	0	1.57	0.63
INTS12	3|2	0	1.11	0.63
THAP11	2|3	0	1.11	0.63
SUN1	8|10	0	1.8	0.63
YTHDF3	8|7	0	1.76	0.63
OAT	19|20	0	2.17	0.64
RPS29	3|3	0	1.2	0.64
COG2	6|3	0	1.29	0.64
FKBP5	3|0	0	0.67	0.64

RHOG	3|4	0	1.27	0.64
CUL2	3|0	0	0.67	0.64
EZH2	2|0	0	0.5	0.64
PIGK	9|7	0	1.68	0.64
DPF2	3|3	0	1.2	0.64
INTS6	5|4	0	1.38	0.64
RRP8	10|9	0	1.81	0.64
CHTOP	5|5	0	1.54	0.64
MTPAP	8|8	0	1.78	0.64
NGLY1	4|3	0	1.27	0.64
CCDC86	2|4	0	1.09	0.64
TARS2	6|3	0	1.29	0.64
CDCA3	3|4	0	1.27	0.64
MRPS21	0|2	0	0.5	0.64
ANKRD27	3|3	0	1.2	0.64
MAK16	7|5	0	1.5	0.64
CYP20A1	0|2	0	0.5	0.64
PEX6	2|0	0	0.5	0.65
CUL7	0|2	0	0.5	0.65
GCAT	4|4	0	1.33	0.65
STX5	2|0	0	0.5	0.65
RAB5B	3|2	0	1	0.65
RNF213	6|10	0	1.52	0.65
TAF4	10|12	0	1.83	0.65
FLOT2	12|10	0	1.83	0.65
POLRMT	15|17	0	2	0.65
CAPZA1	3|2	0	1	0.65
PAXIP1	5|2	0	1.08	0.65
NMD3	4|7	0	1.38	0.65
DERL2	0|2	0	0.5	0.65
VPS35	6|6	0	1.5	0.65
JCAD	5|3	0	1.23	0.65
QTRT2	3|2	0	1	0.65
NAA25	0|3	0	0.6	0.65
VMP1	5|4	0	1.38	0.65
DAGLB	2|0	0	0.5	0.65
METTL15	3|0	0	0.6	0.65
ERI1	3|2	0	1	0.65
FAM111B	2|2	0	0.89	0.65
RPL30	3|4	0	1.17	0.66
SRP9	4|3	0	1.17	0.66
NHSL1	3|6	0	1.2	0.66
ARPC4	3|3	0	1.09	0.66
KAT7	9|8	0	1.7	0.66
CHUK	4|6	0	1.33	0.66

MARCKS	2|2	0	0.89	0.66
FRG1	3|3	0	1.09	0.66
CDIPT	2|2	0	0.89	0.66
ARIH2	3|3	0	1.09	0.66
NUP58	2|2	0	0.89	0.66
MOCS3	2|2	0	0.89	0.66
MLEC	6|5	0	1.47	0.66
AK3	6|4	0	1.33	0.66
FOXRED1	3|3	0	1.09	0.66
NCAPG2	3|3	0	1.09	0.66
SMPD4	11|10	0	1.83	0.66
VIPAS39	3|4	0	1.17	0.66
PCGF1	2|0	0	0.44	0.66
HDHD5	11|12	0	1.84	0.66
UBE3A	4|3	0	1.17	0.66
TIMM29	4|3	0	1.17	0.66
LNPEP	4|6	0	1.33	0.66
PATJ	6|3	0	1.2	0.66
SFXN2	4|3	0	1.17	0.66
CDC16	7|4	0	1.29	0.67
TBC1D8	6|6	0	1.5	0.67
LYN	7|5	0	1.41	0.67
AP1G1	12|11	0	1.84	0.67
VAV2	4|4	0	1.23	0.67
SRSF7	0|2	0	0.44	0.67
C1QBP	0|2	0	0.44	0.67
CPD	7|5	0	1.41	0.67
CTNNAL1	2|0	0	0.44	0.67
CPNE1	2|0	0	0.44	0.67
ZFYVE9	2|0	0	0.44	0.67
ACOT8	5|4	0	1.29	0.67
DNAJA2	12|11	0	1.84	0.67
PRKAB1	4|2	0	1	0.67
NOMO1	7|5	0	1.41	0.67
PELP1	6|6	0	1.5	0.67
SYNE2	11|12	0	1.84	0.67
RBM7	2|0	0	0.44	0.67
COMMD2	0|2	0	0.44	0.67
TOE1	5|7	0	1.41	0.67
NDRG3	2|0	0	0.44	0.67
ITPA	0|2	0	0.44	0.67
CFAP36	3|5	0	1.14	0.67
GLT8D1	2|2	0	0.8	0.67
EXOC1	3|5	0	1.14	0.67
HADHA	3|2	0	0.91	0.68

BCAP31	11|10	0	1.75	0.68
VAMP7	5|5	0	1.33	0.68
EXOC7	2|2	0	0.8	0.68
GPSM1	6|5	0	1.38	0.68
VDAC2	13|12	0	1.85	0.68
EPB41L5	5|5	0	1.33	0.68
PTPN1	8|9	0	1.62	0.68
SLC27A2	12|13	0	1.85	0.68
EPS8	3|0	0	0.55	0.68
DCAF7	3|2	0	0.91	0.68
CRTAP	2|2	0	0.8	0.68
SRCAP	5|6	0	1.38	0.68
TMEM115	3|2	0	0.91	0.68
PIKFYVE	3|4	0	1.08	0.68
UHRF1BP1L	6|4	0	1.25	0.68
POLR1A	13|14	0	1.86	0.68
AAAS	10|6	0	1.45	0.68
APOO	3|4	0	1.08	0.68
TMEM43	3|2	0	0.91	0.68
WDR76	2|3	0	0.91	0.68
DDX11	3|2	0	0.91	0.68
ATP5MD	2|2	0	0.8	0.68
ACSF3	10|10	0	1.74	0.68
DGKH	2|3	0	0.91	0.68
SMARCAD1	24|23	0	2.09	0.69
ERAP1	4|4	0	1.14	0.69
PICALM	10|8	0	1.57	0.69
MAD2L1	4|4	0	1.14	0.69
SSR1	3|2	0	0.83	0.69
TK1	3|3	0	1	0.69
PDHX	4|3	0	1.08	0.69
DCLK1	4|5	0	1.2	0.69
VAMP3	6|6	0	1.41	0.69
ABCB6	2|0	0	0.4	0.69
STAM2	2|0	0	0.4	0.69
ABCC4	7|6	0	1.44	0.69
NEFL	8|7	0	1.5	0.69
RASSF8	5|7	0	1.33	0.69
DHRS7B	8|7	0	1.5	0.69
RPAP1	6|6	0	1.41	0.69
RAB10	5|7	0	1.33	0.69
NRDE2	4|3	0	1.08	0.69
RAB18	5|4	0	1.2	0.69
VPS50	6|6	0	1.41	0.69
DCAKD	9|9	0	1.64	0.69

L2HGDH	4|7	0	1.22	0.69
FBXL18	4|0	0	0.62	0.69
ABRAXAS2	5|4	0	1.2	0.69
PHF6	6|9	0	1.43	0.69
CNOT7	4|4	0	1.14	0.69
MOSPD2	6|7	0	1.44	0.69
ZADH2	4|2	0	0.92	0.69
NF1	0|3	0	0.5	0.7
CSNK1G3	3|0	0	0.5	0.7
CLTA	3|2	0	0.83	0.7
APC	15|12	0	1.74	0.7
INPP1	0|2	0	0.4	0.7
GSTK1	7|7	0	1.47	0.7
DLAT	10|10	0	1.67	0.7
MMP15	3|0	0	0.5	0.7
AP2S1	0|3	0	0.5	0.7
BPHL	3|0	0	0.5	0.7
GRB10	0|2	0	0.4	0.7
COG5	2|2	0	0.73	0.7
CLIC4	2|2	0	0.73	0.7
RRP7A	0|3	0	0.5	0.7
UBXN1	0|3	0	0.5	0.7
VRK3	0|2	0	0.4	0.7
ZWILCH	3|2	0	0.83	0.7
TRMU	2|0	0	0.4	0.7
SMG8	5|5	0	1.25	0.7
DAZAP1	2|2	0	0.73	0.7
SH3GLB2	2|2	0	0.73	0.7
KIAA1522	2|2	0	0.73	0.7
RINT1	5|3	0	1.07	0.7
CERS2	6|5	0	1.29	0.7
TTC13	3|2	0	0.83	0.7
SMC6	10|10	0	1.67	0.7
SLIRP	2|0	0	0.4	0.7
CCNB1	5|3	0	1.07	0.7
NSUN5	5|6	0	1.29	0.7
RASSF2	3|2	0	0.83	0.7
NEDD4	0|2	0	0.4	0.7
PHKB	3|4	0	1	0.71
COASY	8|7	0	1.43	0.71
CPSF4	4|4	0	1.07	0.71
PRKG1	0|2	0	0.36	0.71
EEF1E1	9|8	0	1.55	0.71
IFT81	4|3	0	1	0.71
MOB2	3|3	0	0.92	0.71

GLRX3	4|3	0	1	0.71
DRG2	5|7	0	1.26	0.71
PIK3C2A	6|9	0	1.36	0.71
PSMD7	3|4	0	1	0.71
GOLGB1	13|7	0	1.43	0.71
YBX1	8|8	0	1.52	0.71
PCNT	13|23	0	1.71	0.71
SPTLC1	3|3	0	0.86	0.71
ARFGEF2	0|4	0	0.57	0.71
ATP5MG	7|6	0	1.37	0.71
ESPL1	3|4	0	1	0.71
SACM1L	3|3	0	0.92	0.71
CDC42BPA	4|6	0	1.18	0.71
NDUFA13	6|6	0	1.33	0.71
DYNC1LI1	7|8	0	1.43	0.71
CDK5RAP1	0|4	0	0.57	0.71
INTS11	3|3	0	0.92	0.71
DMAP1	7|8	0	1.43	0.71
RBSN	3|3	0	0.92	0.71
MAIP1	2|0	0	0.36	0.71
TRAPPC9	4|3	0	1	0.71
FAM120B	2|0	0	0.36	0.71
SKP2	4|4	0	1.07	0.71
FOXP1	2|4	0	0.86	0.71
FAM129A	2|0	0	0.36	0.71
GLMN	4|4	0	1.07	0.71
RPUSD3	5|3	0	1	0.71
APOOL	4|0	0	0.57	0.71
PDHB	9|8	0	1.48	0.72
TYMS	9|9	0	1.5	0.72
SAR1A	6|5	0	1.22	0.72
CDK5	11|11	0	1.63	0.72
ATP5MF-PTCD1	6|5	0	1.22	0.72
USP19	3|2	0	0.77	0.72
CSNK1A1	5|8	0	1.24	0.72
PDK1	6|6	0	1.26	0.72
SUMO1	2|2	0	0.67	0.72
STX7	3|2	0	0.77	0.72
VAPA	3|6	0	1	0.72
PPFIA1	10|9	0	1.52	0.72
OXSR1	3|3	0	0.86	0.72
CBX2	6|7	0	1.3	0.72
PDLIM7	3|3	0	0.86	0.72
SCAMP3	2|2	0	0.67	0.72
SLC2A1	2|3	0	0.77	0.72

ENTR1	8|8	0	1.45	0.72
EHD1	3|0	0	0.46	0.72
LIMD1	18|19	0	1.9	0.72
THUMPD1	6|5	0	1.22	0.72
CDKAL1	2|3	0	0.77	0.72
OSGEP	3|2	0	0.77	0.72
VPS35L	3|0	0	0.46	0.72
POLR1E	2|2	0	0.67	0.72
PYCR3	3|4	0	0.93	0.72
BRCC3	3|0	0	0.46	0.72
DHX40	4|6	0	1.11	0.72
RFT1	2|2	0	0.67	0.72
NEK7	2|3	0	0.77	0.72
OTULIN	3|2	0	0.77	0.72
CDC42EP1	0|3	0	0.46	0.72
DHX57	3|4	0	0.93	0.72
OXCT1	3|0	0	0.43	0.73
DNAJC2	3|0	0	0.43	0.73
FBRSL1	2|0	0	0.33	0.73
CDC45	4|4	0	1	0.73
IFRD1	4|5	0	1.06	0.73
C19orf47	2|3	0	0.71	0.73
KIAA0391	8|6	0	1.27	0.73
NDUFS8	0|2	0	0.33	0.73
PRPS2	6|6	0	1.2	0.73
VDAC1	18|16	0	1.79	0.73
AURKA	2|3	0	0.71	0.73
EED	7|4	0	1.1	0.73
FARSB	15|11	0	1.58	0.73
UQCRFS1	2|3	0	0.71	0.73
DYNLRB1	3|5	0	0.94	0.73
R3HCC1L	0|2	0	0.33	0.73
UBXN4	3|2	0	0.71	0.73
PAN2	9|8	0	1.42	0.73
RALGAPA1	4|4	0	1	0.73
RPL36	4|4	0	1	0.73
PTRH2	10|9	0	1.52	0.73
DNAJB11	3|2	0	0.71	0.73
SRBD1	0|4	0	0.53	0.73
RARS2	4|5	0	1.06	0.73
NAV1	7|5	0	1.2	0.73
CAMSAP3	11|13	0	1.6	0.73
HEATR6	3|2	0	0.71	0.73
ZFHX4	0|2	0	0.33	0.73
LPCAT1	4|4	0	1	0.73

HSDL1	4|7	0	1.1	0.73
ZNF503	5|4	0	1.06	0.73
FANCD2	13|9	0	1.47	0.73
IMP4	5|3	0	0.94	0.73
PIK3R1	0|2	0	0.33	0.73
TRMT2A	5|6	0	1.16	0.73
KIF7	2|2	0	0.62	0.73
PLEC	18|16	0	1.79	0.73
RBPJ	2|0	0	0.33	0.73
BRCA2	3|0	0	0.43	0.74
RPL12	4|4	0	0.94	0.74
UNK	2|4	0	0.75	0.74
GXYLT1	4|4	0	0.94	0.74
FBRS	3|3	0	0.8	0.74
GRAMD1A	12|9	0	1.45	0.74
OCLN	2|2	0	0.62	0.74
FAM49B	4|3	0	0.88	0.74
ARF6	4|5	0	1	0.74
DUT	3|6	0	0.95	0.74
H2AFZ	8|9	0	1.42	0.74
RAB3B	4|0	0	0.5	0.74
YY1	10|7	0	1.31	0.74
DYNLL1	3|3	0	0.8	0.74
ARL3	3|2	0	0.71	0.74
BABAM2	2|2	0	0.62	0.74
SCO2	5|3	0	0.89	0.74
TUBGCP3	12|14	0	1.62	0.74
TALDO1	2|2	0	0.62	0.74
VPS45	6|3	0	0.95	0.74
BABAM1	3|4	0	0.88	0.74
DIMT1	13|12	0	1.61	0.74
SCAF8	0|5	0	0.56	0.74
CNOT10	4|2	0	0.75	0.74
AAR2	3|5	0	0.89	0.74
SENP3	11|12	0	1.59	0.74
SBDS	10|6	0	1.23	0.74
MAP1S	5|5	0	1.11	0.74
HSPA12A	9|7	0	1.33	0.74
SPNS1	3|4	0	0.88	0.74
WDR75	7|6	0	1.24	0.74
ATAD1	6|7	0	1.24	0.74
TMEM209	4|4	0	0.94	0.74
SNAP47	6|3	0	0.95	0.74
DNAJB6	3|4	0	0.88	0.74
DOCK11	3|5	0	0.89	0.74

TOR1AIP2	3|3	0	0.8	0.74
MAP2K7	7|6	0	1.24	0.74
CDK5RAP3	2|2	0	0.62	0.74
AUP1	5|5	0	1.11	0.74
CENPV	6|2	0	0.84	0.74
HSD17B4	9|6	0	1.2	0.75
PEX1	3|2	0	0.67	0.75
PRIM1	8|11	0	1.36	0.75
EXOC6	3|2	0	0.67	0.75
RPS26	6|5	0	1.1	0.75
PLAA	4|6	0	1	0.75
RBFOX2	13|14	0	1.64	0.75
EARS2	5|5	0	1.05	0.75
FASTKD2	3|2	0	0.67	0.75
GTF2H1	3|3	0	0.75	0.75
PPAN-P2RY11	14|13	0	1.64	0.75
CNOT9	2|3	0	0.67	0.75
RPS6KB1	2|3	0	0.67	0.75
MTX1	3|8	0	0.96	0.75
PRKCI	2|2	0	0.57	0.75
ELP1	9|8	0	1.36	0.75
EIF2B5	6|2	0	0.8	0.75
BRSK2	2|0	0	0.31	0.75
ERBB2	0|2	0	0.31	0.75
ILK	2|3	0	0.67	0.75
VPS26A	2|0	0	0.31	0.75
THOC1	2|0	0	0.31	0.75
BASP1	3|5	0	0.84	0.75
TAF5	2|2	0	0.57	0.75
TAB2	3|4	0	0.82	0.75
CYB5R4	0|2	0	0.31	0.75
SDAD1	2|3	0	0.67	0.75
TMEM33	5|7	0	1.09	0.75
DNAAF2	2|0	0	0.31	0.75
HOXA10	2|2	0	0.57	0.75
NUDT4	2|0	0	0.31	0.75
3-Sep	2|0	0	0.31	0.75
HIST1H2AE	12|9	0	1.4	0.75
COTL1	0|3	0	0.4	0.75
AGAP3	2|2	0	0.57	0.75
KATNAL1	2|2	0	0.57	0.75
COQ5	6|4	0	1	0.75
CMSS1	4|2	0	0.71	0.75
GFM2	7|6	0	1.18	0.75
TANC1	5|6	0	1.1	0.75

C19orf25	3|2	0	0.67	0.75
IQGAP3	4|0	0	0.47	0.75
COPS8	3|2	0	0.67	0.75
POLR1C	6|8	0	1.22	0.75
GCDH	4|7	0	1	0.76
UMPS	6|3	0	0.86	0.76
RPS5	6|5	0	1.05	0.76
RPS12	4|5	0	0.95	0.76
G6PD	13|8	0	1.31	0.76
CYLD	6|4	0	0.95	0.76
CTNNB1	6|5	0	1.05	0.76
YWHAZ	2|3	0	0.62	0.76
FLYWCH2	3|2	0	0.62	0.76
ARHGAP32	0|2	0	0.29	0.76
UCKL1	0|3	0	0.38	0.76
NEK1	4|4	0	0.89	0.76
RAB5C	7|7	0	1.22	0.76
ATP5PO	8|7	0	1.25	0.76
POLR3D	3|2	0	0.62	0.76
HMGB2	2|2	0	0.53	0.76
NUP88	7|14	0	1.24	0.76
PSMD13	3|0	0	0.38	0.76
TTK	12|11	0	1.48	0.76
SNAP23	4|6	0	0.95	0.76
PHC1	2|2	0	0.53	0.76
HNRNPC	46|41	0	2.07	0.76
HIST1H1C	21|22	0	1.83	0.76
PTPN14	0|2	0	0.29	0.76
MNX1	0|2	0	0.29	0.76
CLPP	10|11	0	1.45	0.76
CDC42BPB	7|7	0	1.22	0.76
SNTB2	11|11	0	1.47	0.76
TMED10	13|11	0	1.5	0.76
DDX19B	11|9	0	1.33	0.76
MKRN2	4|4	0	0.89	0.76
AKAP8L	16|13	0	1.57	0.76
TAF5L	2|2	0	0.53	0.76
UTP20	26|18	0	1.73	0.76
FAF2	2|0	0	0.29	0.76
LTN1	3|5	0	0.84	0.76
PTCD3	3|0	0	0.38	0.76
RBM22	8|6	0	1.17	0.76
PNO1	9|8	0	1.31	0.76
VPS16	2|2	0	0.53	0.76
C11orf49	10|11	0	1.45	0.76

NOTCH2	2|3	0	0.62	0.76
TANGO6	3|0	0	0.38	0.76
NOM1	5|3	0	0.84	0.76
CTSB	5|5	0	1	0.76
TMEM263	5|6	0	1.05	0.76
FYN	11|9	0	1.38	0.76
NUP155	21|19	0	1.78	0.76
EXOSC8	0|2	0	0.29	0.76
CLCC1	4|5	0	0.9	0.77
CREBBP	5|2	0	0.7	0.77
MARK3	6|5	0	1	0.77
TEX264	3|2	0	0.59	0.77
NECAP2	4|3	0	0.78	0.77
DTYMK	8|11	0	1.27	0.77
MPP1	6|5	0	1	0.77
CAPZB	4|4	0	0.84	0.77
ARAF	6|6	0	1.09	0.77
SLC12A2	5|9	0	1.08	0.77
NFKB2	2|3	0	0.59	0.77
PIK3C2B	2|4	0	0.67	0.77
SNRPG	0|2	0	0.27	0.77
NUMB	5|3	0	0.8	0.77
CDS2	3|2	0	0.59	0.77
HDAC3	3|3	0	0.71	0.77
MED14	6|9	0	1.15	0.77
NFKBIE	2|0	0	0.27	0.77
CDC23	19|18	0	1.72	0.77
SRSF4	5|6	0	1	0.77
PRKAA2	6|6	0	1.09	0.77
EIF3M	13|12	0	1.52	0.77
POLR3C	5|2	0	0.7	0.77
SNRPD1	7|5	0	1.04	0.77
RNF2	9|8	0	1.26	0.77
STXBP3	7|5	0	1.04	0.77
ACAD9	5|4	0	0.9	0.77
CXXC1	3|3	0	0.71	0.77
CEP350	13|22	0	1.49	0.77
NUP160	19|19	0	1.77	0.77
AGTPBP1	5|7	0	1.04	0.77
NCAPD3	5|3	0	0.8	0.77
UPF2	4|7	0	0.96	0.77
WDR6	3|4	0	0.74	0.77
DNAJC11	8|12	0	1.29	0.77
PCID2	3|4	0	0.78	0.77
SMG9	3|2	0	0.59	0.77

TIGAR	3|3	0	0.71	0.77
RPTOR	4|2	0	0.67	0.77
SRR	3|2	0	0.59	0.77
CHTF18	5|6	0	1	0.77
DNMT3A	2|0	0	0.27	0.77
VPS52	5|6	0	1	0.77
MPHOSPH9	3|2	0	0.59	0.77
HM13	5|5	0	0.95	0.77
RIOK1	5|5	0	0.95	0.77
INTS4	5|5	0	0.95	0.77
GIT2	5|6	0	1	0.77
MCU	3|5	0	0.8	0.77
FOXP4	2|3	0	0.59	0.77
YWHAB	0|2	0	0.27	0.77
CSNK1E	7|7	0	1.17	0.77
LSM12	0|2	0	0.27	0.77
ECI2	4|5	0	0.9	0.77
FECH	3|0	0	0.33	0.78
GGCX	2|4	0	0.63	0.78
IGF2R	4|4	0	0.8	0.78
RPL27	18|13	0	1.48	0.78
GSTCD	2|0	0	0.25	0.78
RUFY1	2|3	0	0.56	0.78
RTL8C	4|2	0	0.63	0.78
MED24	3|0	0	0.33	0.78
THADA	0|2	0	0.25	0.78
KIAA1217	3|4	0	0.74	0.78
DDX54	11|8	0	1.23	0.78
KANK2	12|12	0	1.45	0.78
POLR1B	2|2	0	0.5	0.78
ALG5	9|13	0	1.29	0.78
UBAC2	2|3	0	0.59	0.78
SATB2	2|2	0	0.5	0.78
NFIB	0|3	0	0.33	0.78
ANXA6	16|15	0	1.59	0.78
BZW1	4|4	0	0.8	0.78
CAMK2G	7|8	0	1.15	0.78
POLR3E	4|3	0	0.74	0.78
KIF18B	5|5	0	0.95	0.78
TRIP6	7|8	0	1.15	0.78
CUL5	8|10	0	1.24	0.78
ARHGAP1	6|4	0	0.87	0.78
RPL10A	9|11	0	1.29	0.78
U2AF2	0|3	0	0.35	0.78
NOB1	4|3	0	0.74	0.78

PIK3R4	5|7	0	1	0.78
PUM3	18|15	0	1.57	0.78
SMG1	3|0	0	0.35	0.78
MAST4	0|3	0	0.33	0.78
SUZ12	7|8	0	1.15	0.78
MORC3	4|3	0	0.74	0.78
GEMIN4	8|8	0	1.23	0.78
NIP7	10|12	0	1.38	0.78
YTHDF1	12|11	0	1.39	0.78
IARS2	4|2	0	0.63	0.78
POLR3B	8|5	0	1	0.78
TMEM165	4|5	0	0.86	0.78
TOMM22	9|11	0	1.29	0.78
EDC3	4|5	0	0.86	0.78
RAB1B	13|14	0	1.5	0.78
COG3	4|2	0	0.63	0.78
MRPL14	5|5	0	0.91	0.78
SNX29	2|3	0	0.56	0.78
AGPAT1	2|3	0	0.59	0.78
PI4KA	10|9	0	1.31	0.78
BCCIP	9|8	0	1.21	0.78
SAMD1	2|2	0	0.5	0.78
LYPLAL1	6|4	0	0.91	0.78
LSM14B	2|0	0	0.25	0.78
UBLCP1	7|11	0	1.2	0.78
MAP3K7	0|3	0	0.35	0.78
PSMA1	2|0	0	0.25	0.78
RFC3	8|9	0	1.21	0.78
PKP2	7|7	0	1.08	0.79
RPS27	8|10	0	1.2	0.79
MYO9B	2|0	0	0.24	0.79
PSMB5	2|0	0	0.24	0.79
TBC1D5	6|6	0	1	0.79
PDK3	5|3	0	0.73	0.79
CTPS2	5|7	0	0.92	0.79
ZMYM3	8|7	0	1.11	0.79
PEG10	6|5	0	0.92	0.79
SMG7	8|6	0	1.04	0.79
HSDL2	9|8	0	1.17	0.79
PI4KB	3|3	0	0.63	0.79
RNF40	11|10	0	1.31	0.79
DYNC1I2	3|3	0	0.63	0.79
HTT	11|8	0	1.19	0.79
ORC5	7|10	0	1.1	0.79
PGD	9|10	0	1.27	0.79

PTPN12	2|3	0	0.53	0.79
RAB6A	9|8	0	1.17	0.79
RAF1	5|6	0	0.92	0.79
UPF1	22|23	0	1.73	0.79
SLC7A5	7|6	0	1.04	0.79
NDUFS3	6|6	0	1	0.79
PCK2	14|15	0	1.49	0.79
CAPN1	6|5	0	0.92	0.79
PDIA6	10|9	0	1.23	0.79
POLR2H	6|4	0	0.83	0.79
TIMM44	12|12	0	1.41	0.79
TM9SF1	0|3	0	0.32	0.79
TRIO	6|7	0	1	0.79
APPL1	10|11	0	1.31	0.79
RABGAP1	3|4	0	0.67	0.79
NUBP2	4|0	0	0.4	0.79
ATAD2	3|3	0	0.6	0.79
ZC3H7A	6|6	0	0.96	0.79
PPP2R1A	4|2	0	0.57	0.79
CTR9	9|9	0	1.24	0.79
AQR	13|14	0	1.46	0.79
C2CD5	2|0	0	0.24	0.79
RABGAP1L	3|2	0	0.53	0.79
GLS	10|7	0	1.1	0.79
RFTN1	3|2	0	0.53	0.79
WASHC4	5|8	0	0.96	0.79
IRF2BP1	3|3	0	0.63	0.79
METTL13	4|3	0	0.67	0.79
PIAS1	3|3	0	0.6	0.79
MTFP1	4|5	0	0.78	0.79
STX18	4|3	0	0.7	0.79
CNOT11	6|6	0	0.96	0.79
CDKN2AIP	7|11	0	1.12	0.79
CMAS	2|0	0	0.24	0.79
DDX55	5|9	0	1	0.79
HIST1H3F	14|13	0	1.46	0.79
PPIA	4|2	0	0.6	0.79
KDM6A	6|6	0	1	0.79
SLC27A3	2|2	0	0.44	0.79
PEAK1	8|9	0	1.17	0.79
NUBPL	2|2	0	0.44	0.79
ARMC6	5|4	0	0.78	0.79
TBC1D2B	4|7	0	0.85	0.79
MRPL10	2|2	0	0.47	0.79
CHEK2	6|6	0	1	0.79

KIF1B	4|6	0	0.83	0.79
C19orf70	3|3	0	0.6	0.79
PMS2	5|5	0	0.8	0.8
BLVRA	8|8	0	1.07	0.8
TROVE2	6|3	0	0.72	0.8
HNRNPUL2	9|11	0	1.21	0.8
INTS1	5|2	0	0.58	0.8
ACTN1	0|9	0	0.58	0.8
SSFA2	4|4	0	0.7	0.8
BAIAP2	2|0	0	0.22	0.8
TTC28	50|56	0	2.02	0.8
UGDH	11|8	0	1.15	0.8
ARHGEF12	9|7	0	1.03	0.8
GTF3C2	4|0	0	0.36	0.8
HSPB1	3|2	0	0.48	0.8
OCRL	5|5	0	0.83	0.8
ITPR3	8|9	0	1.13	0.8
PRKCA	10|9	0	1.15	0.8
PSMD11	5|5	0	0.83	0.8
RING1	5|5	0	0.83	0.8
MAP2K4	5|3	0	0.7	0.8
SRP14	4|4	0	0.7	0.8
RIPK2	2|3	0	0.48	0.8
MCM3AP	6|8	0	1	0.8
UBE2M	4|2	0	0.55	0.8
RECQL5	3|2	0	0.5	0.8
FADS2	0|2	0	0.21	0.8
KIF1A	7|11	0	1.06	0.8
IDI1	3|2	0	0.5	0.8
NDUFA10	7|8	0	1.03	0.8
TOP3A	0|3	0	0.29	0.8
ARHGEF1	5|6	0	0.85	0.8
ZW10	11|11	0	1.29	0.8
SNRNP40	3|5	0	0.7	0.8
ERCC4	2|0	0	0.22	0.8
H1FX	11|13	0	1.33	0.8
PRKCD	0|5	0	0.42	0.8
STXBP2	6|5	0	0.88	0.8
EXOC3	8|6	0	0.97	0.8
NCDN	2|2	0	0.42	0.8
AAK1	6|6	0	0.89	0.8
ANKLE2	12|10	0	1.26	0.8
USP22	3|0	0	0.29	0.8
GORASP2	3|4	0	0.61	0.8
TKFC	3|4	0	0.64	0.8

NBEA	4|3	0	0.61	0.8
CDC40	3|2	0	0.48	0.8
SMN2	5|7	0	0.89	0.8
ECT2	3|4	0	0.64	0.8
LRRC1	5|7	0	0.89	0.8
EXOC2	7|7	0	1.04	0.8
ABCF3	7|7	0	1.04	0.8
UTP6	15|14	0	1.41	0.8
DNAJC10	12|10	0	1.26	0.8
DHX36	3|2	0	0.48	0.8
HIST1H4J	29|16	0	1.43	0.8
SEC24A	9|10	0	1.19	0.8
SMAP2	8|8	0	1.07	0.8
CLPTM1L	11|11	0	1.29	0.8
HNRNPDL	8|5	0	0.9	0.8
MRPS5	6|7	0	0.93	0.8
PURB	4|4	0	0.7	0.8
SYNRG	7|8	0	1.07	0.8
SMARCA2	14|17	0	1.41	0.8
SPATA5	0|4	0	0.36	0.8
CPNE8	5|8	0	0.93	0.8
PTK2	7|7	0	1	0.8
CAMK2D	11|11	0	1.26	0.8
PDZD8	7|5	0	0.89	0.8
EXOC8	4|4	0	0.7	0.8
RFC4	4|5	0	0.75	0.8
HEATR3	4|4	0	0.73	0.8
LIG1	2|4	0	0.52	0.81
KRT2	25|31	0	1.67	0.81
AK1	4|3	0	0.61	0.81
ARFIP1	3|3	0	0.52	0.81
UFD1	2|4	0	0.52	0.81
KIF2A	12|13	0	1.28	0.81
ALDOA	2|0	0	0.2	0.81
ETFA	6|7	0	0.9	0.81
VPS53	2|7	0	0.64	0.81
ZNF384	7|4	0	0.79	0.81
HMMR	4|2	0	0.5	0.81
SETDB1	3|4	0	0.61	0.81
RPS20	8|8	0	1	0.81
GYS1	5|4	0	0.69	0.81
PHF8	11|10	0	1.2	0.81
EIF2D	4|7	0	0.79	0.81
BIRC2	3|5	0	0.62	0.81
TOR1AIP1	7|7	0	0.93	0.81

AP3S1	4|6	0	0.71	0.81
ARL2	9|8	0	1.06	0.81
PKN1	5|4	0	0.69	0.81
PYGB	14|13	0	1.35	0.81
PIP4K2B	3|3	0	0.52	0.81
CUL1	14|10	0	1.17	0.81
RAE1	12|13	0	1.28	0.81
IRS2	7|9	0	1	0.81
SUCLG1	9|9	0	1.12	0.81
BUD31	4|3	0	0.61	0.81
HIP1R	6|3	0	0.64	0.81
ACSL3	21|21	0	1.58	0.81
PWP2	3|5	0	0.64	0.81
GOLGA5	34|34	0	1.81	0.81
AKT1	0|4	0	0.35	0.81
DMXL1	0|2	0	0.2	0.81
FLOT1	6|8	0	0.9	0.81
TNIP1	3|3	0	0.52	0.81
ITSN2	3|4	0	0.61	0.81
SKP1	0|3	0	0.27	0.81
OGFR	3|0	0	0.27	0.81
PYCR2	11|6	0	0.97	0.81
SLC25A24	12|13	0	1.32	0.81
STRN4	6|8	0	0.93	0.81
CCDC22	4|3	0	0.58	0.81
UTP25	10|7	0	1	0.81
KIF14	12|15	0	1.32	0.81
RBM34	11|10	0	1.2	0.81
SARM1	5|8	0	0.87	0.81
LARS2	8|10	0	1.09	0.81
NELFB	14|12	0	1.27	0.81
ANKFY1	3|5	0	0.64	0.81
NUP54	6|5	0	0.79	0.81
VPS13C	11|14	0	1.22	0.81
TRMT10C	12|14	0	1.3	0.81
CWF19L1	2|3	0	0.45	0.81
BRIX1	31|31	0	1.8	0.81
VPS33B	3|6	0	0.64	0.81
BAIAP2L1	3|4	0	0.56	0.81
DIABLO	8|7	0	1	0.81
ARFGEF3	11|11	0	1.19	0.81
SRPRB	6|8	0	0.9	0.81
EEFSEC	3|4	0	0.61	0.81
NT5DC2	13|12	0	1.28	0.81
PHC3	2|5	0	0.56	0.81

ILKAP	4|4	0	0.67	0.81
NTPCR	7|7	0	0.93	0.81
NIFK	8|5	0	0.87	0.81
COX15	3|3	0	0.55	0.81
DBN1	6|4	0	0.74	0.81
TECR	11|9	0	1.11	0.81
RHOT2	9|8	0	1.06	0.81
RBM11	4|5	0	0.72	0.81
STT3A	13|14	0	1.32	0.81
MICU2	2|0	0	0.21	0.81
TAB1	5|6	0	0.81	0.81
ACTL6A	4|8	0	0.8	0.81
TRNT1	10|9	0	1.12	0.81
ZNF326	15|16	0	1.44	0.81
RPS19	11|11	0	1.13	0.82
HIST2H2AA4	13|11	0	1.17	0.82
WDR62	8|7	0	0.88	0.82
PPP6C	3|4	0	0.54	0.82
PARK7	4|3	0	0.56	0.82
SRRT	16|13	0	1.29	0.82
LDHA	17|16	0	1.4	0.82
SCAF4	6|7	0	0.84	0.82
PALLD	5|7	0	0.8	0.82
ARL1	6|5	0	0.73	0.82
RPS10	15|13	0	1.3	0.82
MAVS	4|4	0	0.62	0.82
EIF3L	12|9	0	1.11	0.82
CLPB	4|6	0	0.69	0.82
CDC27	6|5	0	0.76	0.82
CSNK2B	5|5	0	0.69	0.82
GCLM	0|3	0	0.25	0.82
HLA-A	5|4	0	0.64	0.82
KPNA1	8|9	0	0.97	0.82
POLR2E	7|8	0	0.94	0.82
SMARCB1	3|3	0	0.48	0.82
DNAJC7	12|8	0	1.03	0.82
VRK1	18|22	0	1.43	0.82
DEK	7|7	0	0.88	0.82
SNX4	3|3	0	0.5	0.82
RNGTT	6|7	0	0.81	0.82
SUCLA2	15|15	0	1.33	0.82
NAE1	5|3	0	0.57	0.82
AP2M1	7|6	0	0.87	0.82
DRG1	12|11	0	1.15	0.82
RAB5A	4|5	0	0.67	0.82

CHD1L	6|4	0	0.67	0.82
ARHGEF2	17|14	0	1.29	0.82
VAPB	6|7	0	0.87	0.82
PFN1	5|4	0	0.67	0.82
SLC27A4	6|7	0	0.87	0.82
ZMYM4	6|10	0	0.86	0.82
DNAJA3	7|8	0	0.88	0.82
USP10	12|13	0	1.22	0.82
PCMT1	6|6	0	0.8	0.82
PHF2	4|4	0	0.62	0.82
BCKDK	3|2	0	0.4	0.82
SEC24B	11|10	0	1.11	0.82
TIMM23	5|5	0	0.71	0.82
MAP4K5	5|5	0	0.69	0.82
RALBP1	4|2	0	0.48	0.82
CLP1	3|2	0	0.43	0.82
IDH3B	14|14	0	1.27	0.82
SRP72	9|10	0	1.09	0.82
TMF1	2|2	0	0.36	0.82
UHRF1	10|5	0	0.83	0.82
PKN3	5|5	0	0.71	0.82
TUBGCP4	3|2	0	0.43	0.82
MDN1	50|44	0	1.94	0.82
ZNF592	4|2	0	0.48	0.82
CCP110	16|18	0	1.42	0.82
PPP6R1	6|4	0	0.67	0.82
MON2	13|13	0	1.27	0.82
VWA8	15|14	0	1.32	0.82
NUDCD3	4|2	0	0.44	0.82
NOL11	3|4	0	0.54	0.82
TBC1D10B	7|6	0	0.84	0.82
COPS4	3|5	0	0.57	0.82
TEX10	4|6	0	0.67	0.82
DDX27	25|21	0	1.56	0.82
USP47	0|2	0	0.19	0.82
HEATR1	26|33	0	1.64	0.82
RNF20	8|6	0	0.88	0.82
SCYL1	6|4	0	0.69	0.82
INTS2	3|2	0	0.42	0.82
PLEKHA1	7|4	0	0.69	0.82
ZNF711	7|3	0	0.65	0.82
WDR18	4|5	0	0.64	0.82
INA	13|11	0	1.17	0.82
PIGS	9|6	0	0.86	0.82
HK1	22|16	0	1.36	0.82

YME1L1	7|5	0	0.77	0.82
SNX6	8|7	0	0.94	0.82
DCP1B	9|8	0	1	0.82
ARID2	6|6	0	0.83	0.82
PATL1	9|6	0	0.88	0.82
EIF2B4	10|8	0	1.03	0.82
FAM98B	3|6	0	0.62	0.82
TMTC3	9|9	0	1.03	0.82
GANAB	4|4	0	0.62	0.82
SFXN4	4|4	0	0.62	0.82
GALK1	10|7	0	0.87	0.83
RPL11	14|17	0	1.19	0.83
RPL23	17|20	0	1.32	0.83
RPL19	13|14	0	1.17	0.83
RPL32	20|17	0	1.32	0.83
MTUS1	0|3	0	0.24	0.83
USP16	0|4	0	0.29	0.83
YARS2	18|19	0	1.37	0.83
DYNC2H1	10|9	0	0.93	0.83
SMARCD2	3|6	0	0.56	0.83
CKAP2	12|12	0	1.14	0.83
SUGT1	13|14	0	1.2	0.83
NEDD1	12|14	0	1.13	0.83
AGFG1	9|8	0	0.92	0.83
CLASP1	4|6	0	0.61	0.83
CCT7	23|19	0	1.38	0.83
EIF2B3	5|6	0	0.69	0.83
RBM15	24|24	0	1.52	0.83
KIF22	19|20	0	1.42	0.83
DDX56	11|12	0	1.07	0.83
PPME1	7|6	0	0.79	0.83
CTBP1	11|12	0	1.12	0.83
EIF2B1	10|8	0	0.92	0.83
PFKL	24|27	0	1.52	0.83
PIK3C3	5|3	0	0.5	0.83
PLS1	5|8	0	0.7	0.83
PSMA7	5|4	0	0.62	0.83
PSMD12	6|5	0	0.69	0.83
VCL	3|0	0	0.23	0.83
NAA10	11|11	0	1.07	0.83
TTF2	19|21	0	1.38	0.83
DPM1	11|12	0	1.07	0.83
SYNJ2	3|5	0	0.55	0.83
EIF2S2	22|27	0	1.46	0.83
BYSL	16|14	0	1.2	0.83

AURKB	5|7	0	0.73	0.83
SNRPD2	5|6	0	0.69	0.83
ASH2L	4|3	0	0.52	0.83
DDX23	19|16	0	1.35	0.83
SEC22B	10|10	0	0.98	0.83
DOPEY2	5|3	0	0.52	0.83
SLC1A5	7|6	0	0.76	0.83
FARP1	19|17	0	1.38	0.83
PURA	6|7	0	0.76	0.83
PRMT5	4|0	0	0.29	0.83
STK3	6|6	0	0.69	0.83
YKT6	4|5	0	0.56	0.83
KHDRBS1	9|10	0	0.97	0.83
KIF1C	0|4	0	0.3	0.83
DDX52	11|11	0	1.07	0.83
CDC37	9|9	0	0.97	0.83
CHORDC1	17|17	0	1.28	0.83
G3BP2	0|3	0	0.23	0.83
ANAPC4	6|9	0	0.77	0.83
AP2A1	8|5	0	0.76	0.83
UBE3C	6|8	0	0.8	0.83
XPO7	22|21	0	1.48	0.83
DNMBP	5|6	0	0.65	0.83
TUT4	4|4	0	0.53	0.83
SORBS1	3|4	0	0.5	0.83
AASDHPPT	6|7	0	0.76	0.83
SAMHD1	5|7	0	0.73	0.83
TELO2	5|7	0	0.71	0.83
ANAPC7	8|11	0	0.95	0.83
YTHDF2	11|10	0	1.05	0.83
ZC3H7B	3|5	0	0.5	0.83
PIH1D1	6|3	0	0.55	0.83
SCYL2	10|12	0	1.02	0.83
RPRD1A	11|10	0	1	0.83
PBRM1	7|7	0	0.82	0.83
CTTNBP2NL	6|5	0	0.71	0.83
CCDC47	11|12	0	1.1	0.83
KIDINS220	6|6	0	0.73	0.83
MMS19	11|9	0	0.95	0.83
ACBD3	6|2	0	0.5	0.83
CTBP2	14|12	0	1.13	0.83
UBXN6	3|3	0	0.44	0.83
RPF2	14|14	0	1.24	0.83
TCHP	4|6	0	0.62	0.83
AP1M1	14|13	0	1.15	0.83

DHX37	10|12	0	1.07	0.83
CDK16	5|6	0	0.71	0.83
CNP	5|5	0	0.65	0.83
EHD4	9|8	0	0.87	0.83
OSBPL9	6|4	0	0.62	0.83
ZNF598	5|4	0	0.56	0.83
PPP2R5D	11|11	0	1.07	0.83
ACOT7	17|19	0	1.31	0.83
PRKACB	12|10	0	1	0.83
SHC1	6|4	0	0.62	0.83
RPL7L1	7|8	0	0.86	0.83
CARM1	5|5	0	0.62	0.83
TPD52L2	3|4	0	0.52	0.83
ZNF644	2|3	0	0.4	0.83
PRKAA1	9|9	0	0.97	0.83
AP3M1	9|8	0	0.92	0.83
NEBL	3|4	0	0.52	0.83
RB1	3|5	0	0.48	0.84
WRN	4|7	0	0.59	0.84
RPL22	14|12	0	1.06	0.84
RPL35A	10|9	0	0.93	0.84
RPLP2	7|7	0	0.72	0.84
PUS1	14|14	0	1.06	0.84
ECHDC1	7|5	0	0.63	0.84
DNM1	9|6	0	0.71	0.84
ELP6	9|11	0	0.83	0.84
YTHDC1	9|7	0	0.8	0.84
HNRNPH2	15|14	0	1.07	0.84
ACOT9	12|15	0	1.02	0.84
DIAPH3	7|9	0	0.74	0.84
SULT1A1	13|11	0	0.94	0.84
SEC31A	7|3	0	0.56	0.84
KIAA1191	4|4	0	0.52	0.84
GIT1	9|10	0	0.83	0.84
IFT74	8|12	0	0.89	0.84
MAPT	3|4	0	0.44	0.84
CSK	11|12	0	0.94	0.84
MFN2	5|5	0	0.59	0.84
TOMM40	9|7	0	0.8	0.84
MYO1B	13|17	0	1.11	0.84
EIF2AK2	13|14	0	1.12	0.84
CAND2	10|8	0	0.78	0.84
ATXN10	7|7	0	0.74	0.84
CBS	8|5	0	0.67	0.84
CNOT2	5|7	0	0.63	0.84

SSR4	9|7	0	0.8	0.84
NUB1	5|5	0	0.59	0.84
TBRG4	14|14	0	1.06	0.84
CNOT1	53|58	0	1.82	0.84
ABCB7	11|10	0	0.88	0.84
PSMD6	27|27	0	1.46	0.84
CHD2	6|4	0	0.51	0.84
CTNND1	17|16	0	1.18	0.84
DNAJA1	15|14	0	1.12	0.84
DSG2	11|11	0	0.96	0.84
PRPS1	9|9	0	0.88	0.84
RPS6KA1	19|23	0	1.35	0.84
SPTBN1	17|12	0	1.02	0.84
STIM1	5|5	0	0.56	0.84
UBE2N	8|6	0	0.72	0.84
UQCRC2	9|8	0	0.79	0.84
EIF3F	11|10	0	0.89	0.84
TIMELESS	4|5	0	0.51	0.84
BCR	5|3	0	0.46	0.84
FARSA	18|18	0	1.24	0.84
EIF4E2	22|26	0	1.41	0.84
TRIM25	22|22	0	1.4	0.84
LMO7	13|16	0	1.04	0.84
LMNB1	35|34	0	1.68	0.84
RHEB	6|5	0	0.65	0.84
ALYREF	4|3	0	0.4	0.84
PSMD14	6|5	0	0.59	0.84
PRDX2	6|5	0	0.56	0.84
SLC25A1	9|10	0	0.86	0.84
RPL10	15|12	0	1.08	0.84
PA2G4	10|15	0	0.98	0.84
PKN2	28|27	0	1.49	0.84
SYNCRIP	12|8	0	0.83	0.84
POLD3	8|6	0	0.72	0.84
CKAP4	7|4	0	0.56	0.84
KIF2C	8|6	0	0.7	0.84
KATNA1	4|0	0	0.23	0.84
SLC25A10	12|11	0	0.96	0.84
KPNA6	9|12	0	0.86	0.84
GTPBP4	17|20	0	1.25	0.84
ORC3	7|7	0	0.72	0.84
STOML2	15|15	0	1.11	0.84
TNRC6A	9|12	0	0.84	0.84
ARFGAP3	6|4	0	0.56	0.84
FAM120A	8|12	0	0.82	0.84

PPM1F	7|11	0	0.82	0.84
PUM1	18|20	0	1.31	0.84
SETD1A	10|8	0	0.88	0.84
MORC2	21|14	0	1.09	0.84
ALMS1	61|63	0	1.98	0.84
MYO1D	10|10	0	0.89	0.84
SCFD1	12|10	0	0.92	0.84
MYEF2	12|13	0	1.04	0.84
LUC7L3	7|7	0	0.76	0.84
ZC3HC1	4|6	0	0.56	0.84
CARMIL1	4|5	0	0.53	0.84
H2AFY2	28|25	0	1.41	0.84
PLEKHA5	17|16	0	1.16	0.84
PARD3	9|11	0	0.91	0.84
NCLN	24|17	0	1.24	0.84
NUP107	12|14	0	1	0.84
MRTFA	0|8	0	0.4	0.84
OSBPL8	26|27	0	1.51	0.84
RPRD1B	12|14	0	1.04	0.84
CFL2	8|9	0	0.79	0.84
JUP	12|11	0	1	0.84
SUDS3	6|6	0	0.65	0.84
RPS18	9|8	0	0.85	0.84
NOL6	15|14	0	1.09	0.84
MCMBP	11|10	0	0.98	0.84
USP36	6|7	0	0.72	0.84
MAP2K2	17|19	0	1.29	0.84
HNRNPAB	16|16	0	1.23	0.84
CPVL	4|5	0	0.55	0.84
ZNF512	9|8	0	0.81	0.84
UTP4	5|8	0	0.67	0.84
ZNF622	6|4	0	0.59	0.84
APBB1	6|7	0	0.72	0.84
LMNA	27|26	0	1.51	0.84
STT3B	9|8	0	0.81	0.84
KLC4	5|5	0	0.61	0.84
DLD	16|16	0	1.1	0.85
FH	9|7	0	0.59	0.85
RPL18A	9|9	0	0.77	0.85
RPL21	14|15	0	0.94	0.85
RPL28	13|13	0	0.88	0.85
RPL38	8|10	0	0.69	0.85
RPS6	13|15	0	1.02	0.85
TIMM50	15|13	0	0.9	0.85
RPS9	19|22	0	1.12	0.85

RPS11	15|16	0	0.95	0.85
CACYBP	8|7	0	0.62	0.85
RPS13	16|16	0	1.08	0.85
SH3D19	4|7	0	0.46	0.85
INF2	7|8	0	0.65	0.85
MYO1C	25|24	0	1.26	0.85
NXF1	18|17	0	1.15	0.85
CEP170B	8|9	0	0.63	0.85
EEF1D	9|9	0	0.71	0.85
PABPC4	10|8	0	0.68	0.85
AIMP1	11|12	0	0.87	0.85
P4HA1	16|13	0	0.98	0.85
CCDC85C	11|11	0	0.83	0.85
KIAA1671	48|48	0	1.67	0.85
SRP54	7|5	0	0.49	0.85
SLC25A13	20|18	0	1.15	0.85
NKRF	27|24	0	1.26	0.85
LDHB	8|10	0	0.77	0.85
C15orf38-AP3S2	4|4	0	0.41	0.85
RPL17	14|13	0	0.89	0.85
PRKAG1	9|9	0	0.77	0.85
MTHFD1L	16|17	0	0.99	0.85
RPL13A	9|12	0	0.78	0.85
EIF2S3	16|18	0	1.11	0.85
ELAVL1	22|20	0	1.14	0.85
FBL	29|27	0	1.29	0.85
INPPL1	8|8	0	0.7	0.85
ARF4	12|14	0	0.9	0.85
ARF5	12|15	0	0.9	0.85
FKBP4	8|7	0	0.67	0.85
RBMX	12|10	0	0.88	0.85
PFKP	37|33	0	1.41	0.85
PRKACA	19|18	0	1.06	0.85
MAP2K1	14|18	0	0.97	0.85
MAP2K3	4|5	0	0.43	0.85
PSMC3	19|16	0	1.04	0.85
PSMC6	10|12	0	0.83	0.85
SLC16A1	17|16	0	1.02	0.85
KMT2D	7|5	0	0.57	0.85
USO1	11|9	0	0.77	0.85
EIF3G	12|8	0	0.75	0.85
FUBP3	31|27	0	1.32	0.85
BTAF1	10|12	0	0.79	0.85
LLGL1	7|9	0	0.67	0.85
TRIP13	15|16	0	0.94	0.85

TRIP12	9|6	0	0.56	0.85
GTPBP1	16|11	0	0.93	0.85
FOXK2	3|6	0	0.45	0.85
NARS	7|2	0	0.42	0.85
RPS6KA3	24|25	0	1.23	0.85
DDOST	11|10	0	0.79	0.85
GLUD1	12|12	0	0.87	0.85
GNL1	6|7	0	0.55	0.85
HDAC5	10|14	0	0.87	0.85
CFL1	11|11	0	0.81	0.85
ABCF2	20|20	0	1.1	0.85
TSFM	12|13	0	0.93	0.85
RAD50	65|59	0	1.81	0.85
PAK4	16|20	0	1	0.85
RACK1	22|18	0	1.19	0.85
PCBP1	22|22	0	1.22	0.85
STK4	6|8	0	0.56	0.85
AIMP2	9|10	0	0.76	0.85
TACC3	8|10	0	0.67	0.85
AFG3L2	22|20	0	1.24	0.85
ASCC3	17|19	0	1.04	0.85
POLR3A	10|10	0	0.77	0.85
DDX20	9|10	0	0.76	0.85
SEC63	20|18	0	1.09	0.85
XPOT	14|13	0	1	0.85
TARDBP	10|9	0	0.81	0.85
HNRNPH3	30|27	0	1.28	0.85
RAB3GAP1	9|6	0	0.65	0.85
KNTC1	7|9	0	0.71	0.85
HELZ	18|20	0	1.07	0.85
PDCD11	65|67	0	1.83	0.85
TNRC6B	34|38	0	1.52	0.85
USP24	7|8	0	0.64	0.85
UFL1	28|27	0	1.29	0.85
NUP188	19|21	0	1.08	0.85
IBTK	14|16	0	0.97	0.85
PRPF31	12|9	0	0.74	0.85
NOC2L	22|21	0	1.16	0.85
UBR5	7|6	0	0.58	0.85
PELO	10|8	0	0.75	0.85
UTP18	12|11	0	0.9	0.85
ANAPC5	11|15	0	0.93	0.85
PAXBP1	12|11	0	0.9	0.85
CRNKL1	14|17	0	1.03	0.85
RBM28	30|26	0	1.3	0.85

DCP1A	12|12	0	0.94	0.85
COQ8A	12|10	0	0.76	0.85
MTA3	14|13	0	0.86	0.85
TRMT5	7|6	0	0.57	0.85
RELCH	6|4	0	0.5	0.85
EXOC4	16|25	0	1.05	0.85
NOL9	10|10	0	0.71	0.85
NOL10	6|9	0	0.59	0.85
LAS1L	8|12	0	0.75	0.85
C1orf198	7|7	0	0.57	0.85
SEC23B	13|14	0	0.93	0.85
RPLP0	10|13	0	0.82	0.85
PTPN13	31|30	0	1.37	0.85
NIPBL	10|11	0	0.74	0.85
FAM91A1	8|8	0	0.65	0.85
AP1B1	6|7	0	0.58	0.85
N4BP1	4|8	0	0.46	0.85
RFC5	11|12	0	0.88	0.85
PCNA	11|10	0	0.84	0.85
MPRIP	5|0	0	0.22	0.85
NCKAP1	12|11	0	0.9	0.85
ATP6V1H	6|8	0	0.55	0.85
RPL9	23|22	0	0.94	0.86
POLR2A	19|22	0	1.02	0.86
POLR2B	24|23	0	1.16	0.86
RPL5	25|24	0	1.05	0.86
RPL7A	28|28	0	1.18	0.86
RPL23A	24|20	0	1.02	0.86
RPS4X	26|27	0	1.05	0.86
RPS8	14|15	0	0.81	0.86
IGF2BP2	22|23	0	1.03	0.86
CUL4A	11|13	0	0.67	0.86
RPS15A	16|17	0	0.9	0.86
RPS16	21|22	0	1.02	0.86
NME1-NME2	11|8	0	0.54	0.86
CYFIP2	9|12	0	0.63	0.86
KTN1	14|14	0	0.85	0.86
ABCF1	19|15	0	0.79	0.86
HNRNPF	18|16	0	0.84	0.86
IMMT	25|25	0	1.19	0.86
SLFN11	9|10	0	0.64	0.86
SUPT5H	23|22	0	1.05	0.86
KARS	11|14	0	0.7	0.86
HYOU1	31|35	0	1.15	0.86
RPS27A	15|16	0	0.76	0.86

RPN2	31|29	0	1.26	0.86
ANXA2	16|14	0	0.77	0.86
PFKM	16|16	0	0.85	0.86
PGAM5	18|19	0	1.03	0.86
WNK1	16|15	0	0.87	0.86
PRDX1	13|13	0	0.79	0.86
XIAP	10|10	0	0.69	0.86
NVL	9|8	0	0.62	0.86
MYH10	24|14	0	0.84	0.86
DOCK7	14|13	0	0.75	0.86
AP2B1	13|15	0	0.81	0.86
EIF4G2	26|26	0	1.02	0.86
EIF3E	21|20	0	1.04	0.86
IRAK1	14|14	0	0.86	0.86
ATP2B1	26|25	0	1.12	0.86
CANX	18|16	0	0.92	0.86
CSNK2A2	15|14	0	0.83	0.86
HNRNPD	15|17	0	0.88	0.86
KPNB1	9|14	0	0.57	0.86
TNPO1	12|11	0	0.74	0.86
PABPC1	13|10	0	0.66	0.86
PAK2	16|22	0	0.83	0.86
SLC25A3	35|35	0	1.24	0.86
PSMC1	20|19	0	0.84	0.86
PSMC5	22|23	0	0.99	0.86
ABCD3	15|18	0	0.85	0.86
ABCE1	10|10	0	0.65	0.86
RPL15	20|23	0	0.98	0.86
RPN1	21|20	0	1.05	0.86
ITSN1	18|21	0	0.89	0.86
SPTAN1	15|13	0	0.76	0.86
SLC25A11	22|26	0	0.96	0.86
EIF3D	17|19	0	0.82	0.86
TRIM24	8|5	0	0.47	0.86
SYNJ1	21|17	0	0.9	0.86
EIF2S1	19|20	0	0.87	0.86
DVL3	29|26	0	1.15	0.86
EPHA2	8|7	0	0.49	0.86
HSD17B10	9|10	0	0.67	0.86
STAU1	18|18	0	0.99	0.86
SNAP29	10|11	0	0.64	0.86
SYMPK	16|14	0	0.86	0.86
PRDX6	20|18	0	0.82	0.86
MTOR	14|7	0	0.67	0.86
SAE1	25|24	0	0.98	0.86

RPS14	14|15	0	0.87	0.86
CEBPZ	21|25	0	0.98	0.86
AASS	13|13	0	0.78	0.86
TUBA4A	84|78	0	1.95	0.86
NPEPPS	25|20	0	0.89	0.86
RBM14	30|30	0	1.1	0.86
CCT2	38|35	0	1.25	0.86
TBL3	18|18	0	0.96	0.86
IGF2BP3	27|25	0	1.11	0.86
IQGAP2	28|26	0	1.14	0.86
MCM5	16|16	0	0.79	0.86
HNRNPA0	23|20	0	0.99	0.86
SKIV2L	13|13	0	0.64	0.86
COPE	11|12	0	0.72	0.86
GTF3C5	14|16	0	0.9	0.86
ABCB10	11|11	0	0.73	0.86
MACF1	33|27	0	1.1	0.86
AHSA1	15|17	0	0.86	0.86
GTF3C4	13|14	0	0.75	0.86
AP2A2	11|11	0	0.7	0.86
RTCB	15|13	0	0.67	0.86
GNL3	28|27	0	1.16	0.86
CYFIP1	13|16	0	0.79	0.86
TTC37	8|9	0	0.58	0.86
NCAPD2	24|22	0	0.98	0.86
SEC16A	28|28	0	1.24	0.86
DIS3	38|34	0	1.38	0.86
LIMCH1	5|10	0	0.49	0.86
PDS5B	38|38	0	1.3	0.86
CMTR1	15|15	0	0.71	0.86
CLASP2	13|12	0	0.72	0.86
NUP205	37|38	0	1.35	0.86
RRP12	21|19	0	1.05	0.86
NCAPH	16|16	0	0.79	0.86
EP400	19|22	0	1.04	0.86
PTPN23	11|11	0	0.7	0.86
VIRMA	10|4	0	0.44	0.86
KIF1BP	14|16	0	0.73	0.86
NOP58	25|22	0	1.06	0.86
RPL26L1	22|16	0	0.86	0.86
HSD17B12	19|18	0	1.03	0.86
MRTO4	12|12	0	0.77	0.86
DDX47	21|19	0	1.03	0.86
WWOX	23|27	0	1.16	0.86
HACD3	12|16	0	0.82	0.86

ILF3	19|14	0	0.78	0.86
CC2D1A	7|5	0	0.47	0.86
LRRC40	17|17	0	0.83	0.86
DNAAF5	18|16	0	0.93	0.86
DENND4C	11|13	0	0.73	0.86
WDR11	12|10	0	0.62	0.86
ARFGAP1	12|14	0	0.69	0.86
PANK4	11|11	0	0.72	0.86
CCAR1	10|9	0	0.6	0.86
ELP4	13|10	0	0.65	0.86
EIF4ENIF1	12|12	0	0.69	0.86
DDX24	25|24	0	1.03	0.86
ESYT2	20|16	0	0.83	0.86
ARHGAP21	14|16	0	0.82	0.86
CWC22	7|7	0	0.5	0.86
SFXN1	21|21	0	0.99	0.86
KLC2	10|15	0	0.64	0.86
YTHDC2	26|30	0	1.13	0.86
ATAD3B	20|20	0	0.93	0.86
PCBP2	27|25	0	1.12	0.86
PTBP1	26|20	0	1.1	0.86
NEK9	10|11	0	0.56	0.86
RPL13	23|19	0	1.04	0.86
RPL8	16|15	0	0.75	0.86
CDK2	14|17	0	0.79	0.86
TRIM9	6|9	0	0.44	0.86
OPA1	23|18	0	1.05	0.86
CRYBG3	10|12	0	0.55	0.86
ERC1	31|35	0	1.27	0.86
HNRNPA3	19|18	0	0.9	0.86
MSH6	27|24	0	0.89	0.87
MSH2	30|25	0	0.82	0.87
ATP1A1	27|33	0	0.92	0.87
RPL3	34|38	0	0.88	0.87
RPL4	35|31	0	0.86	0.87
RPL7	38|37	0	1.09	0.87
RPL26	25|18	0	0.82	0.87
RPS3A	34|32	0	1.05	0.87
RPS7	32|32	0	0.79	0.87
SMC4	35|33	0	0.7	0.87
WASHC2A	26|24	0	0.62	0.87
HSP90AA1	55|53	0	1.03	0.87
TP53BP2	21|23	0	0.88	0.87
USP9X	35|41	0	0.76	0.87
XRN1	41|49	0	0.82	0.87

STAG2	22|16	0	0.62	0.87
ECPAS	34|33	0	1	0.87
THOC2	28|28	0	0.96	0.87
DDX17	45|41	0	0.94	0.87
PDS5A	33|29	0	0.94	0.87
FANCI	17|23	0	0.78	0.87
SMARCA4	25|30	0	0.96	0.87
COPB1	23|29	0	0.68	0.87
PHB2	28|28	0	0.89	0.87
SLC25A4	29|33	0	0.91	0.87
SLC25A5	40|42	0	0.88	0.87
ABR	12|10	0	0.55	0.87
PYGL	29|29	0	0.96	0.87
CHAMP1	24|21	0	0.8	0.87
TJP2	16|15	0	0.64	0.87
DNM2	27|23	0	0.93	0.87
NSUN2	29|28	0	0.79	0.87
EIF3C	28|24	0	0.77	0.87
LRBA	30|32	0	0.82	0.87
SERPINH1	27|31	0	0.95	0.87
HNRNPH1	40|33	0	1.04	0.87
NOP2	35|35	0	0.87	0.87
CHD1	17|17	0	0.63	0.87
DARS	31|30	0	1.01	0.87
DHX15	48|45	0	1.08	0.87
XRCC6	42|32	0	0.9	0.87
GTF3C1	23|20	0	0.66	0.87
HDAC2	23|17	0	0.67	0.87
HNRNPL	30|27	0	1.02	0.87
SLC25A6	37|37	0	0.86	0.87
ARCN1	26|25	0	0.87	0.87
ARF1	18|18	0	0.77	0.87
CDK1	33|31	0	0.83	0.87
EIF4A2	27|27	0	0.9	0.87
HSPD1	37|35	0	1.03	0.87
KRT19	39|32	0	1.12	0.87
LBR	30|30	0	0.91	0.87
MCM3	21|25	0	0.71	0.87
MSH3	15|10	0	0.54	0.87
PSMC2	22|22	0	0.88	0.87
PSMD2	28|30	0	0.85	0.87
PSMD3	35|29	0	0.81	0.87
ALDH18A1	41|47	0	0.82	0.87
RARS	34|34	0	0.85	0.87
SRPRA	22|22	0	0.9	0.87

SSRP1	23|21	0	0.8	0.87
SVIL	16|19	0	0.66	0.87
TOP1	30|30	0	1.05	0.87
HSP90B1	45|54	0	1.14	0.87
TUFM	48|57	0	1.04	0.87
XPO1	39|40	0	0.98	0.87
AP3B1	23|21	0	0.82	0.87
YARS	33|30	0	0.88	0.87
RUVBL1	52|56	0	1.39	0.87
EIF3B	27|31	0	0.81	0.87
EIF4G3	32|38	0	0.75	0.87
IQGAP1	43|34	0	0.87	0.87
GMPS	25|30	0	0.85	0.87
PRPF4B	20|16	0	0.75	0.87
AP3D1	19|24	0	0.81	0.87
DCTN1	29|29	0	0.71	0.87
HSPA9	51|50	0	0.97	0.87
CAD	67|64	0	1.16	0.87
COPA	32|33	0	1.05	0.87
DDX5	43|40	0	0.86	0.87
DSP	48|48	0	1.17	0.87
DVL2	53|48	0	1.3	0.87
KIF11	23|27	0	0.79	0.87
MCM2	35|38	0	1.11	0.87
LONP1	35|33	0	0.84	0.87
CLTC	69|59	0	1.29	0.87
ACTN4	29|27	0	0.88	0.87
DDX1	30|30	0	0.79	0.87
MARS	32|28	0	0.8	0.87
QARS	33|37	0	1	0.87
NUP214	56|65	0	1.01	0.87
CRKL	34|37	0	0.78	0.87
DIAPH1	22|24	0	0.69	0.87
SMC3	62|56	0	1.05	0.87
UBA2	30|27	0	0.95	0.87
MCM6	23|24	0	0.78	0.87
MTHFD1	50|44	0	0.85	0.87
HNRNPM	98|92	0	1.28	0.87
ARID1A	33|33	0	0.81	0.87
PCM1	37|38	0	0.76	0.87
RAD21	11|17	0	0.6	0.87
VARS	34|35	0	0.95	0.87
SMC1A	64|70	0	1.13	0.87
RAN	32|31	0	0.87	0.87
NOP56	39|41	0	1.04	0.87

CCT4	43|41	0	0.86	0.87
SMC2	39|38	0	0.96	0.87
PDLIM5	22|16	0	0.74	0.87
IGF2BP1	40|43	0	1.12	0.87
DDX18	41|44	0	1.16	0.87
WDR3	28|28	0	1	0.87
SUPT16H	56|52	0	1	0.87
CCT5	38|39	0	0.83	0.87
PRPF6	31|32	0	1.07	0.87
PRRC2B	16|20	0	0.72	0.87
LIG3	33|29	0	0.98	0.87
PES1	15|15	0	0.68	0.87
EDC4	20|22	0	0.75	0.87
SND1	49|48	0	1.24	0.87
NUP93	29|37	0	0.9	0.87
EIF4A3	27|28	0	0.96	0.87
BMS1	15|12	0	0.48	0.87
DHX30	35|30	0	0.99	0.87
RRP1B	17|14	0	0.62	0.87
WASHC2C	0|24	0	0.37	0.87
SMCHD1	52|46	0	1.04	0.87
CAMSAP1	25|25	0	0.81	0.87
DST	32|36	0	0.84	0.87
RAI14	21|23	0	0.54	0.87
GAPVD1	27|26	0	0.94	0.87
RSL1D1	34|37	0	0.99	0.87
HP1BP3	22|22	0	0.76	0.87
NUP98	29|28	0	0.93	0.87
HAUS6	29|30	0	0.72	0.87
FTSJ3	24|24	0	0.74	0.87
ATAD3A	32|33	0	0.86	0.87
NUP133	19|18	0	0.74	0.87
ZC3HAV1	25|23	0	0.89	0.87
ANKHD1-EIF4EBP3	35|34	0	1.02	0.87
UBR4	28|28	0	0.94	0.87
XRCC5	40|37	0	0.88	0.87
HSPA2	27|28	0	0.65	0.87
NOC3L	21|19	0	0.82	0.87
DDX50	21|26	0	0.74	0.87
MAP7D3	16|17	0	0.68	0.87
TRMT1L	13|15	0	0.64	0.87
HNRNPA1	30|31	0	0.79	0.87
HNRNPA2B1	50|45	0	1.03	0.87
EPPK1	32|41	0	0.76	0.87
HUWE1	64|51	0	0.96	0.87

ANKRD17	34|30	0	0.7	0.87
DDX39B	25|25	0	0.81	0.87
LRPPRC	31|29	0	1	0.87
AHNAK2	36|44	0	1.05	0.87
H2AFY	32|34	0	0.98	0.87
AIFM1	17|20	0	0.73	0.87
SNX1	14|15	0	0.56	0.87
ATP2A2	39|36	0	0.9	0.87
CSNK2A1	25|25	0	0.96	0.87
SCRIB	24|28	0	0.61	0.87
MCM4	39|44	0	0.92	0.87
KRT18	23|21	0	0.77	0.87
ACAT1	12|11	0	0.45	0.88
ADSL	2|2	0	0.31	0.88
EMD	11|11	0	0.75	0.88
MTR	2|0	0	0.08	0.88
PON2	2|2	0	0.38	0.88
ALDH3A2	0|3	0	0.19	0.88
PCCB	123|135	0	1.05	0.88
TP53	8|10	0	0.39	0.88
PRIM2	0|3	0	0.14	0.88
RPL6	30|29	0	0.67	0.88
RPL24	4|4	0	0.73	0.88
RPL27A	4|4	0	0.59	0.88
RPL34	3|3	0	0.75	0.88
RPS3	40|42	0	0.76	0.88
ATP5F1C	8|10	0	0.45	0.88
EXOSC10	26|18	0	0.44	0.88
JPT1	3|3	0	0.48	0.88
GPATCH8	2|2	0	0.14	0.88
CHD3	17|21	0	0.33	0.88
KDM2B	2|0	0	0.08	0.88
SDCBP	0|2	0	0.11	0.88
TFIP11	14|15	0	0.43	0.88
CKAP5	79|82	0	0.83	0.88
RPS15	2|3	0	0.4	0.88
KDM1A	12|9	0	0.51	0.88
7-Sep	14|15	0	0.49	0.88
SLC3A2	6|7	0	0.46	0.88
SLTM	4|3	0	0.5	0.88
RPS23	5|5	0	0.67	0.88
SH3PXD2B	2|0	0	0.12	0.88
SERBP1	19|18	0	0.42	0.88
ZNF451	10|9	0	0.57	0.88
STK24	0|3	0	0.15	0.88

EFL1	5|3	0	0.4	0.88
PSPC1	17|16	0	0.62	0.88
TFAP2A	3|0	0	0.21	0.88
CAST	3|0	0	0.06	0.88
STK26	6|4	0	0.43	0.88
TOP2A	95|98	0	0.7	0.88
TOP2B	59|57	0	0.86	0.88
TUBB2A	101|94	0	0.67	0.88
TUBG1	14|16	0	0.62	0.88
QSER1	2|3	0	0.4	0.88
SREK1	3|3	0	0.23	0.88
NCOR2	3|5	0	0.3	0.88
TBCE	6|3	0	0.42	0.88
PAICS	13|16	0	0.53	0.88
CASZ1	0|3	0	0.19	0.88
U2SURP	14|17	0	0.24	0.88
MGA	3|4	0	0.1	0.88
PXN	4|4	0	0.43	0.88
RABEP1	4|3	0	0.3	0.88
ACACB	60|71	0	0.98	0.88
ACLY	14|9	0	0.45	0.88
ZBTB21	3|4	0	0.25	0.88
RPL31	8|9	0	0.71	0.88
8-Sep	5|4	0	0.42	0.88
CCNK	7|7	0	0.64	0.88
POM121C	2|2	0	0.4	0.88
ZNF146	3|3	0	0.6	0.88
PHF21A	2|0	0	0.25	0.88
HNRNPR	14|12	0	0.45	0.88
PSMA4	2|0	0	0.15	0.88
GIGYF2	47|55	0	0.76	0.88
RPP30	3|5	0	0.39	0.88
CAP1	4|5	0	0.35	0.88
ZBTB10	3|4	0	0.27	0.88
MECP2	2|0	0	0.04	0.88
EZR	11|11	0	0.4	0.88
TLK2	4|0	0	0.22	0.88
FAM208A	8|11	0	0.37	0.88
NACA	3|3	0	0.39	0.88
LIMA1	29|31	0	0.64	0.88
ADD1	10|8	0	0.45	0.88
ADD3	6|6	0	0.28	0.88
DBNL	4|6	0	0.29	0.88
BCOR	19|19	0	0.35	0.88
RACGAP1	0|2	0	0.06	0.88

SHTN1	6|8	0	0.25	0.88
POLD2	2|3	0	0.53	0.88
UBAP2L	25|29	0	0.44	0.88
ANK2	5|5	0	0.31	0.88
PCCA	252|248	0	1.48	0.88
PMS1	2|2	0	0.24	0.88
PSIP1	10|7	0	0.29	0.88
SMARCC2	4|5	0	0.28	0.88
CSDE1	21|21	0	0.44	0.88
EIF4E	0|3	0	0.3	0.88
DNMT1	0|3	0	0.05	0.88
CIZ1	8|8	0	0.32	0.88
PEX5	3|2	0	0.29	0.88
RBM20	2|3	0	0.16	0.88
MAP4	37|37	0	0.39	0.88
PARN	2|2	0	0.3	0.88
IPO11	5|5	0	0.53	0.88
FIP1L1	7|7	0	0.29	0.88
FERMT2	3|5	0	0.29	0.88
TKT	0|2	0	0.2	0.88
NDRG1	2|0	0	0.07	0.88
TCOF1	9|10	0	0.12	0.88
RBBP4	0|4	0	0.14	0.88
ECD	2|3	0	0.12	0.88
ABL2	3|3	0	0.43	0.88
GART	47|43	0	0.72	0.88
PLS3	2|0	0	0.07	0.88
CPSF7	2|0	0	0.1	0.88
RCC2	4|4	0	0.29	0.88
TP53BP1	8|7	0	0.27	0.88
SPART	8|9	0	0.47	0.88
DKC1	8|8	0	0.47	0.88
P4HA2	3|0	0	0.24	0.88
API5	7|5	0	0.56	0.88
EIF5A	0|4	0	0.15	0.88
PPHLN1	2|0	0	0.14	0.88
NFRKB	2|0	0	0.11	0.88
PPP1R12A	9|6	0	0.41	0.88
HBS1L	8|8	0	0.39	0.88
SIN3A	10|8	0	0.15	0.88
NONO	131|116	0	1.09	0.88
STMN1	3|4	0	0.34	0.88
TBC1D15	16|15	0	0.63	0.88
EBNA1BP2	3|3	0	0.32	0.88
ZC3H14	5|3	0	0.19	0.88

TNIK	8|9	0	0.51	0.88
PPP2R5C	3|2	0	0.32	0.88
FAM135A	3|2	0	0.33	0.88
ACIN1	7|9	0	0.11	0.88
RRM2	2|2	0	0.22	0.88
EPB41	30|32	0	0.52	0.88
LEMD3	11|11	0	0.4	0.88
LPP	6|5	0	0.46	0.88
FUS	9|5	0	0.49	0.88
ISYNA1	2|3	0	0.28	0.88
RIF1	24|26	0	0.48	0.88
ZBTB33	30|30	0	0.57	0.88
ZMYM2	13|13	0	0.56	0.88
SRSF11	6|6	0	0.29	0.88
DDX3X	45|44	0	0.82	0.88
ADAR	22|25	0	0.44	0.88
CLINT1	26|24	0	0.62	0.88
KMT2A	5|8	0	0.18	0.88
DPYSL2	27|29	0	0.48	0.88
DPYSL3	19|21	0	0.42	0.88
GBF1	6|7	0	0.46	0.88
INTS7	5|4	0	0.43	0.88
PGM3	4|3	0	0.45	0.88
NIPSNAP2	2|2	0	0.33	0.88
NR3C1	2|0	0	0.19	0.88
ANK3	8|9	0	0.42	0.88
ELOC	0|3	0	0.24	0.88
GTF3C3	9|9	0	0.6	0.88
AFDN	63|67	0	0.57	0.88
BUB1B	19|20	0	0.5	0.88
RBM39	11|10	0	0.68	0.88
HLCS	12|12	0	0.42	0.88
PPP6R2	0|2	0	0.21	0.88
AKAP1	6|10	0	0.4	0.88
AKT2	3|3	0	0.48	0.88
CCNT1	11|9	0	0.37	0.88
PPP1CC	6|7	0	0.33	0.88
CLIP1	0|2	0	0.2	0.88
USP15	15|20	0	0.38	0.88
ERBIN	16|14	0	0.49	0.88
C9orf72	0|2	0	0.17	0.88
FLII	6|5	0	0.5	0.88
MSTO1	4|2	0	0.5	0.88
TUBGCP2	13|13	0	0.61	0.88
FAM193A	2|0	0	0.21	0.88

PAF1	7|6	0	0.33	0.88
CDC5L	18|17	0	0.36	0.88
MLH1	3|5	0	0.38	0.88
EPS15L1	26|31	0	0.57	0.88
SLC4A7	3|2	0	0.16	0.88
RDX	9|9	0	0.39	0.88
DCTN2	5|4	0	0.33	0.88
CDK9	8|4	0	0.34	0.88
PDCD6	10|10	0	0.68	0.88
RPL18	8|8	0	0.53	0.88
STRIP1	0|2	0	0.14	0.88
CHD4	78|82	0	0.51	0.88
TBCB	0|3	0	0.12	0.88
CLPTM1	2|2	0	0.42	0.88
CRMP1	6|5	0	0.31	0.88
CSE1L	32|33	0	0.53	0.88
CSTF2	5|0	0	0.15	0.88
DHX9	104|103	0	0.99	0.88
DYNC1H1	184|186	0	1.04	0.88
EEF1A1	45|44	0	0.35	0.88
EEF1G	15|15	0	0.7	0.88
EIF4A1	45|48	0	0.66	0.88
EIF4B	12|13	0	0.31	0.88
ENO1	84|77	0	0.85	0.88
EPB41L2	48|44	0	0.79	0.88
FOXC1	0|2	0	0.1	0.88
FLNA	367|381	0	0.53	0.88
FLNB	129|132	0	0.74	0.88
IGBP1	3|5	0	0.36	0.88
PARP1	102|95	0	0.87	0.88
AHNAK	457|460	0	0.95	0.88
ATP1B3	3|3	0	0.39	0.88
ATP5F1B	65|63	0	0.96	0.88
ATP6V1A	7|2	0	0.35	0.88
CCT6A	32|31	0	0.59	0.88
CENPC	3|3	0	0.16	0.88
CKB	5|2	0	0.38	0.88
CNN3	9|8	0	0.4	0.88
CTPS1	19|18	0	0.53	0.88
DHPS	0|2	0	0.13	0.88
EEF1A2	26|28	0	0.37	0.88
EEF2	30|31	0	0.51	0.88
EIF5	4|5	0	0.17	0.88
EPS15	11|12	0	0.44	0.88
GARS	2|3	0	0.38	0.88

GFPT1	6|6	0	0.56	0.88
GSPT1	0|2	0	0.08	0.88
KIFC1	9|9	0	0.52	0.88
KPNA2	9|8	0	0.39	0.88
IPO5	19|14	0	0.41	0.88
LCP1	0|3	0	0.12	0.88
ABLIM1	4|5	0	0.35	0.88
MKI67	70|69	0	0.23	0.88
MYBL2	4|3	0	0.33	0.88
MYH9	54|42	0	0.33	0.88
NASP	12|11	0	0.13	0.88
NBN	4|6	0	0.31	0.88
NPM1	18|18	0	0.15	0.88
PHB	22|20	0	0.58	0.88
PHKA1	3|4	0	0.44	0.88
PKM	40|39	0	0.53	0.88
PLRG1	8|7	0	0.56	0.88
PNN	8|11	0	0.33	0.88
POLD1	21|20	0	0.59	0.88
PPP1CA	8|8	0	0.48	0.88
PPP1CB	5|6	0	0.43	0.88
PPP1R10	6|9	0	0.11	0.88
PRKAR2B	3|0	0	0.2	0.88
PSMD4	4|5	0	0.67	0.88
PSMD8	2|3	0	0.29	0.88
PSMD10	2|2	0	0.47	0.88
TWF1	2|2	0	0.3	0.88
PTPN11	5|6	0	0.2	0.88
RANBP1	2|2	0	0.32	0.88
RANGAP1	21|16	0	0.46	0.88
RBBP7	5|5	0	0.22	0.88
RFC1	17|16	0	0.6	0.88
RFC2	5|5	0	0.47	0.88
RFX1	2|0	0	0.05	0.88
RPA1	8|8	0	0.35	0.88
RPA2	2|0	0	0.09	0.88
RPS2	16|16	0	0.64	0.88
SAFB	7|3	0	0.22	0.88
SBF1	4|4	0	0.37	0.88
ATXN2	2|5	0	0.24	0.88
SET	4|4	0	0.34	0.88
SH3GL1	2|3	0	0.21	0.88
SMARCA1	16|19	0	0.41	0.88
HLTF	4|3	0	0.41	0.88
SMARCC1	2|0	0	0.14	0.88

SMARCE1	2|0	0	0.07	0.88
SNRNP70	7|11	0	0.41	0.88
SNRPA1	8|8	0	0.21	0.88
SNRPE	3|2	0	0.36	0.88
SNX2	12|12	0	0.68	0.88
SRPK1	4|3	0	0.34	0.88
SSB	2|0	0	0.05	0.88
STAT3	9|9	0	0.39	0.88
STRN	2|0	0	0.17	0.88
SUPT6H	5|2	0	0.14	0.88
TMPO	29|23	0	0.36	0.88
TPR	79|74	0	0.46	0.88
TTC1	2|2	0	0.4	0.88
UBA1	4|3	0	0.24	0.88
VIM	123|113	0	1.04	0.88
ZYX	5|5	0	0.29	0.88
USP7	10|13	0	0.46	0.88
STAM	0|2	0	0.11	0.88
TAGLN2	12|12	0	0.41	0.88
DHX16	9|7	0	0.4	0.88
CUL4B	9|9	0	0.37	0.88
SMARCA5	48|48	0	0.64	0.88
IRS4	60|65	0	0.91	0.88
PPFIBP1	2|2	0	0.26	0.88
RANBP3	10|12	0	0.37	0.88
PARG	2|3	0	0.37	0.88
KHSRP	28|28	0	0.7	0.88
NOP14	10|6	0	0.42	0.88
EIF3A	64|63	0	0.84	0.88
EIF3I	13|13	0	0.51	0.88
MTMR1	0|3	0	0.12	0.88
ALDH1A2	4|7	0	0.21	0.88
FUBP1	5|8	0	0.34	0.88
MBD3	0|4	0	0.16	0.88
SPAG9	23|25	0	0.53	0.88
RPL14	6|5	0	0.71	0.88
ATP5F1A	73|79	0	0.87	0.88
FASN	134|141	0	0.27	0.88
FEN1	10|10	0	0.56	0.88
GTF2F2	3|3	0	0.13	0.88
PRKAR2A	5|5	0	0.38	0.88
SNRPD3	3|3	0	0.71	0.88
WARS	15|17	0	0.4	0.88
EFTUD2	27|20	0	0.61	0.88
BAG3	2|2	0	0.13	0.88

BAG2	5|5	0	0.51	0.88
DDX6	14|16	0	0.75	0.88
DDX10	15|12	0	0.35	0.88
2-Sep	11|12	0	0.4	0.88
DVL1	10|11	0	0.5	0.88
EPRS	74|76	0	0.75	0.88
BPTF	11|15	0	0.43	0.88
HNRNPU	81|72	0	0.92	0.88
ILF2	14|11	0	0.38	0.88
KIF5B	29|30	0	0.58	0.88
KIF5C	0|6	0	0.16	0.88
PEX14	2|4	0	0.38	0.88
SFSWAP	0|2	0	0.05	0.88
SNRPA	2|3	0	0.29	0.88
TAF1	2|2	0	0.06	0.88
COIL	9|5	0	0.44	0.88
USP11	0|2	0	0.11	0.88
MTA1	31|29	0	0.52	0.88
PRPF4	13|15	0	0.59	0.88
PRPF3	13|12	0	0.31	0.88
FAM50A	0|3	0	0.11	0.88
PDCD5	4|4	0	0.76	0.88
BUB3	3|5	0	0.4	0.88
DDX21	90|85	0	0.87	0.88
ETF1	11|10	0	0.54	0.88
MTA2	23|20	0	0.66	0.88
NOLC1	9|6	0	0.27	0.88
RPS6KA5	4|0	0	0.24	0.88
COPB2	11|10	0	0.62	0.88
MED1	3|3	0	0.08	0.88
FXR2	8|5	0	0.46	0.88
VPS4B	2|2	0	0.53	0.88
MARK2	3|3	0	0.6	0.88
HDAC1	18|16	0	0.59	0.88
MYO6	20|19	0	0.65	0.88
SFPQ	57|48	0	0.89	0.88
TLE3	5|6	0	0.33	0.88
FXR1	5|5	0	0.36	0.88
AKAP12	11|17	0	0.43	0.88
THRAP3	10|9	0	0.17	0.88
NUP153	35|36	0	0.5	0.88
SART1	2|2	0	0.04	0.88
USP14	5|5	0	0.34	0.88
CBL	5|6	0	0.46	0.88
CTTN	46|46	0	0.67	0.88

GAK	4|2	0	0.44	0.88
HCFC1	32|31	0	0.53	0.88
HSPA1B	104|109	0	0.48	0.88
HSPA5	57|57	0	0.84	0.88
NCL	18|21	0	0.19	0.88
PDCL	2|3	0	0.4	0.88
SALL2	0|3	0	0.23	0.88
CHAF1B	4|2	0	0.36	0.88
MRE11	39|42	0	0.43	0.88
TAF7	7|8	0	0.54	0.88
TFCP2	2|2	0	0.44	0.88
RBM10	3|5	0	0.08	0.88
ACTR2	3|3	0	0.43	0.88
ACTR1A	9|7	0	0.44	0.88
NAMPT	22|23	0	0.75	0.88
TRIM28	12|12	0	0.25	0.88
RCL1	4|4	0	0.47	0.88
PSME3	5|6	0	0.23	0.88
DDX39A	21|22	0	0.75	0.88
PLIN3	0|2	0	0.06	0.88
SRRM1	2|3	0	0.19	0.88
AKAP8	2|0	0	0.15	0.88
SAP18	4|4	0	0.19	0.88
BCAS2	3|3	0	0.23	0.88
SF3A1	13|11	0	0.28	0.88
KATNB1	3|0	0	0.29	0.88
MAP1B	51|56	0	0.39	0.88
MAT2A	2|0	0	0.08	0.88
MCM7	47|47	0	0.81	0.88
TBCD	3|5	0	0.28	0.88
CCT3	42|41	0	0.79	0.88
TSNAX	2|3	0	0.29	0.88
TUBA1A	82|85	0	0.52	0.88
IK	4|4	0	0.1	0.88
TUBB3	72|65	0	0.71	0.88
TUBB4A	95|90	0	0.67	0.88
TUBB4B	110|101	0	0.68	0.88
SCML2	19|17	0	0.31	0.88
HAX1	7|7	0	0.62	0.88
DYNC1LI2	3|3	0	0.44	0.88
DNAJB1	7|6	0	0.31	0.88
NCK1	5|5	0	0.42	0.88
NSF	8|12	0	0.53	0.88
NUMA1	29|33	0	0.3	0.88
ORC2	8|6	0	0.16	0.88

POLE	6|6	0	0.48	0.88
RANBP2	159|160	0	1.13	0.88
TACC1	13|12	0	0.47	0.88
TLN1	44|42	0	0.4	0.88
XRCC1	6|8	0	0.43	0.88
NCOR1	5|3	0	0.12	0.88
PGRMC2	3|3	0	0.32	0.88
PPIH	6|6	0	0.52	0.88
SEC23A	4|5	0	0.42	0.88
LRRC41	8|8	0	0.63	0.88
CHERP	10|6	0	0.21	0.88
IPO8	2|3	0	0.32	0.88
IPO7	6|6	0	0.41	0.88
RNASEH2A	3|3	0	0.5	0.88
ARL6IP5	0|2	0	0.24	0.88
SLU7	2|2	0	0.11	0.88
DPYSL4	9|11	0	0.28	0.88
PRPF8	61|60	0	0.77	0.88
SPAG5	0|2	0	0.1	0.88
ARID3B	4|5	0	0.12	0.88
TAF6L	2|3	0	0.37	0.88
POLH	3|3	0	0.25	0.88
PSMC4	15|16	0	0.59	0.88
SARS	0|2	0	0.18	0.88
CCT8	105|109	0	0.99	0.88
HSPA8	72|80	0	0.64	0.88
NUDC	11|14	0	0.42	0.88
PHGDH	14|14	0	0.49	0.88
9-Sep	16|21	0	0.51	0.88
HSPH1	3|3	0	0.11	0.88
UTP14A	7|7	0	0.33	0.88
CLPX	2|5	0	0.42	0.88
TCERG1	19|14	0	0.27	0.88
EHMT2	0|2	0	0.08	0.88
SURF6	0|5	0	0.14	0.88
YWHAE	4|3	0	0.44	0.88
BRAP	9|8	0	0.35	0.88
SF3A3	6|4	0	0.33	0.88
STIP1	3|2	0	0.24	0.88
GCN1	83|88	0	0.7	0.88
SF3B2	21|21	0	0.3	0.88
ILVBL	8|6	0	0.42	0.88
PRKDC	217|219	0	1.11	0.88
RBBP6	4|3	0	0.07	0.88
SUMO2	2|0	0	0.25	0.88

TACC2	2|2	0	0.31	0.88
NUDT21	9|9	0	0.24	0.88
CPSF6	2|0	0	0.05	0.88
WDR45	0|2	0	0.15	0.88
UTRN	69|68	0	0.95	0.88
VCP	2|4	0	0.08	0.88
SF3A2	7|6	0	0.39	0.88
STRAP	8|8	0	0.35	0.88
SEC23IP	4|6	0	0.33	0.88
ATXN2L	34|33	0	0.47	0.88
LYPLA2	4|5	0	0.49	0.88
STK38	6|9	0	0.41	0.88
CBX3	4|5	0	0.31	0.88
TWF2	3|3	0	0.4	0.88
BRCA1	4|3	0	0.26	0.88
STAT1	16|14	0	0.62	0.88
HSP90AB1	71|69	0	0.78	0.88
DDX42	29|25	0	0.54	0.88
DNM1L	22|25	0	0.71	0.88
TPX2	7|10	0	0.12	0.88
CD2AP	44|45	0	0.46	0.88
COPG2	34|35	0	0.58	0.88
AATF	4|0	0	0.21	0.88
OGA	5|5	0	0.26	0.88
SNW1	5|5	0	0.15	0.88
XRN2	26|28	0	0.68	0.88
KEAP1	11|11	0	0.51	0.88
EPB41L3	50|49	0	0.61	0.88
KDM2A	2|2	0	0.24	0.88
KIF4A	9|8	0	0.23	0.88
MAPRE1	0|2	0	0.07	0.88
NUP62	3|6	0	0.58	0.88
PFAS	3|0	0	0.22	0.88
ZMYND8	4|4	0	0.12	0.88
RAB3GAP2	10|12	0	0.57	0.88
IFIT5	6|8	0	0.26	0.88
SF3B3	40|45	0	0.67	0.88
SF3B1	86|87	0	0.76	0.88
TNPO3	6|6	0	0.57	0.88
UCK2	2|0	0	0.09	0.88
ZNF281	4|5	0	0.25	0.88
FHOD1	4|0	0	0.24	0.88
CFAP20	3|2	0	0.3	0.88
SAP30BP	4|4	0	0.17	0.88
VPS51	3|3	0	0.41	0.88

GNL2	23|23	0	0.67	0.88
CPSF1	13|12	0	0.36	0.88
SEC61A1	8|7	0	0.65	0.88
PDCD6IP	31|31	0	0.69	0.88
IARS	60|56	0	0.97	0.88
BAZ1A	14|13	0	0.49	0.88
BAZ2A	5|3	0	0.08	0.88
DHX38	6|9	0	0.42	0.88
SNRNP200	65|68	0	0.97	0.88
CHMP2B	3|3	0	0.57	0.88
MRTFB	96|98	0	0.95	0.88
NUDT5	4|4	0	0.37	0.88
SETD2	4|0	0	0.17	0.88
SRP68	19|20	0	0.6	0.88
C11orf58	0|2	0	0.1	0.88
MAPRE2	7|7	0	0.33	0.88
PUF60	7|9	0	0.22	0.88
CORO1C	13|15	0	0.36	0.88
ZNF318	14|19	0	0.22	0.88
STAU2	9|7	0	0.44	0.88
AFF4	4|5	0	0.35	0.88
PALD1	7|8	0	0.45	0.88
PDLIM3	2|0	0	0.16	0.88
ZNF638	12|12	0	0.26	0.88
UBE2S	3|2	0	0.42	0.88
PRPF19	15|16	0	0.46	0.88
MYBBP1A	72|69	0	0.91	0.88
DNTTIP2	12|13	0	0.63	0.88
MAGED2	14|13	0	0.56	0.88
GSE1	9|10	0	0.34	0.88
MDC1	22|21	0	0.17	0.88
ZNF516	3|2	0	0.32	0.88
SAFB2	11|7	0	0.36	0.88
TTI1	2|0	0	0.2	0.88
PHF14	5|4	0	0.45	0.88
SART3	10|9	0	0.13	0.88
ZNF536	7|4	0	0.26	0.88
SLK	24|26	0	0.54	0.88
KMT2B	3|2	0	0.16	0.88
ZFYVE16	4|7	0	0.27	0.88
BCLAF1	10|10	0	0.16	0.88
DLGAP5	10|10	0	0.36	0.88
CEP170	57|59	0	0.61	0.88
ZC3H11A	11|7	0	0.3	0.88
TOX4	7|8	0	0.19	0.88

DDX46	34|40	0	0.31	0.88
TBC1D4	2|3	0	0.38	0.88
SUGP2	21|19	0	0.39	0.88
PDAP1	4|4	0	0.29	0.88
SPEN	27|31	0	0.36	0.88
PDXDC1	9|10	0	0.67	0.88
POP1	4|2	0	0.18	0.88
WAPL	11|9	0	0.33	0.88
WDR43	4|0	0	0.21	0.88
RTF1	5|4	0	0.21	0.88
PASK	2|4	0	0.21	0.88
PHF3	7|11	0	0.27	0.88
LARP4B	2|0	0	0.14	0.88
RRS1	2|0	0	0.06	0.88
PRRC2C	52|48	0	0.61	0.88
VPS13A	2|2	0	0.42	0.88
ANKRD28	3|3	0	0.23	0.88
BOP1	5|4	0	0.2	0.88
RPRD2	9|12	0	0.23	0.88
PPIP5K2	3|3	0	0.27	0.88
CSTF2T	9|8	0	0.56	0.88
MICAL3	0|2	0	0.1	0.88
ANKS1A	15|14	0	0.43	0.88
EHBP1	5|5	0	0.31	0.88
FKBP15	3|3	0	0.46	0.88
SIN3B	17|11	0	0.19	0.88
DNAJC13	3|2	0	0.37	0.88
ESYT1	25|25	0	0.59	0.88
LARP1	38|37	0	0.48	0.88
ADNP	22|18	0	0.56	0.88
MTREX	11|13	0	0.58	0.88
AHCTF1	15|13	0	0.21	0.88
DPCD	2|2	0	0.57	0.88
LARP7	4|4	0	0.5	0.88
GEMIN5	36|38	0	0.68	0.88
RAB11FIP5	3|4	0	0.3	0.88
UBXN7	2|0	0	0.09	0.88
LSM14A	2|0	0	0.11	0.88
GPKOW	2|2	0	0.09	0.88
NOP53	3|3	0	0.41	0.88
EIF5B	7|8	0	0.28	0.88
TRIM33	3|4	0	0.3	0.88
TAF9B	2|2	0	0.3	0.88
WIPI2	4|5	0	0.34	0.88
RTRAF	8|10	0	0.54	0.88

EXOSC3	3|0	0	0.18	0.88
SF3B6	5|6	0	0.69	0.88
PPIL1	0|2	0	0.16	0.88
CIAO2B	0|2	0	0.21	0.88
HDDC2	3|3	0	0.41	0.88
ZFR	23|20	0	0.49	0.88
COPG1	13|17	0	0.61	0.88
KIF20B	3|0	0	0.24	0.88
RBM19	0|2	0	0.2	0.88
CPSF3	9|9	0	0.43	0.88
DDX41	5|3	0	0.19	0.88
SNX9	4|3	0	0.26	0.88
TRAP1	62|59	0	0.89	0.88
CMPK1	4|4	0	0.52	0.88
WBP11	2|0	0	0.04	0.88
SRRM2	20|24	0	0.46	0.88
RAPGEF6	9|8	0	0.52	0.88
CHMP5	0|3	0	0.19	0.88
GTSE1	0|2	0	0.12	0.88
CDK12	8|7	0	0.35	0.88
RSF1	5|5	0	0.26	0.88
KDM3B	10|10	0	0.18	0.88
ESF1	2|0	0	0.03	0.88
CRK	31|28	0	0.67	0.88
POLA1	11|13	0	0.42	0.88
CPSF2	4|4	0	0.23	0.88
ARID1B	5|6	0	0.33	0.88
CDV3	6|6	0	0.53	0.88
WDR5	2|2	0	0.33	0.88
GATAD2A	12|9	0	0.42	0.88
ZNF280C	4|4	0	0.36	0.88
ERCC6L	6|6	0	0.53	0.88
BCAS3	2|2	0	0.24	0.88
FAM208B	2|0	0	0.11	0.88
SPDL1	0|2	0	0.06	0.88
PRPF40A	10|10	0	0.48	0.88
NPLOC4	6|4	0	0.41	0.88
YEATS2	5|11	0	0.32	0.88
GPATCH1	19|16	0	0.42	0.88
WDR70	0|3	0	0.05	0.88
ATG2B	44|45	0	0.6	0.88
HELLS	8|9	0	0.37	0.88
YJU2	3|4	0	0.4	0.88
LARP1B	5|5	0	0.43	0.88
IPO9	6|6	0	0.36	0.88

ELAC2	2|3	0	0.31	0.88
TSR1	2|0	0	0.07	0.88
CEP55	3|2	0	0.23	0.88
SBNO1	3|3	0	0.23	0.88
ENAH	2|4	0	0.16	0.88
11-Sep	10|10	0	0.53	0.88
INTS9	2|2	0	0.24	0.88
WDR41	5|4	0	0.6	0.88
PPP6R3	3|2	0	0.19	0.88
DDX19A	10|0	0	0.31	0.88
WDR33	7|12	0	0.31	0.88
LSG1	7|6	0	0.5	0.88
STRBP	3|0	0	0.21	0.88
CAND1	50|47	0	0.69	0.88
UBAP2	10|11	0	0.56	0.88
TEX2	3|3	0	0.48	0.88
ZC3H15	4|0	0	0.16	0.88
MCM10	2|3	0	0.45	0.88
UNC45A	9|9	0	0.47	0.88
MATR3	40|40	0	0.74	0.88
RBM27	0|4	0	0.06	0.88
DHX29	10|13	0	0.4	0.88
PUS7	4|2	0	0.17	0.88
EML4	3|2	0	0.26	0.88
DDX49	4|5	0	0.43	0.88
UBFD1	0|2	0	0.08	0.88
YLPM1	16|16	0	0.2	0.88
MEPCE	4|3	0	0.25	0.88
LARS	69|75	0	0.84	0.88
DPYSL5	18|16	0	0.45	0.88
WRNIP1	8|11	0	0.43	0.88
RAD18	2|0	0	0.13	0.88
MCCC1	150|159	0	1.42	0.88
EMSY	4|3	0	0.21	0.88
XAB2	17|15	0	0.47	0.88
CIAPIN1	2|5	0	0.21	0.88
PCNP	4|4	0	0.3	0.88
UTP3	5|4	0	0.43	0.88
REXO4	7|4	0	0.24	0.88
CORO1B	20|22	0	0.31	0.88
RTN4	7|6	0	0.36	0.88
CBX8	2|2	0	0.42	0.88
GATAD2B	11|10	0	0.44	0.88
ZNF512B	5|0	0	0.12	0.88
KIAA1211	5|3	0	0.17	0.88

XPO5	17|11	0	0.41	0.88
NUFIP2	16|14	0	0.45	0.88
GPHN	2|0	0	0.1	0.88
ZNF687	5|5	0	0.38	0.88
TSHZ3	2|0	0	0.07	0.88
CIP2A	9|10	0	0.45	0.88
CHD8	11|15	0	0.31	0.88
PDLIM1	12|12	0	0.63	0.88
NR2F2	2|0	0	0.2	0.88
MYL6	3|2	0	0.23	0.88
HIST1H2BB	111|100	0	1.09	0.88
HIST1H2BD	115|103	0	1.12	0.88
EEF1B2	0|2	0	0.19	0.88
CCAR2	20|22	0	0.55	0.88
PRUNE1	4|2	0	0.27	0.88
RBM25	13|6	0	0.31	0.88
WIZ	6|6	0	0.26	0.88
RIC8A	4|5	0	0.69	0.88
SMAP1	3|2	0	0.36	0.88
EGLN1	0|2	0	0.18	0.88
UBE2O	9|10	0	0.35	0.88
RBM26	2|3	0	0.06	0.88
MCCC2	59|56	0	0.59	0.88
EIF4H	3|0	0	0.21	0.88
PC	267|271	0	0.96	0.88
NCAPG	8|8	0	0.43	0.88
NSD1	2|3	0	0.1	0.88
ANAPC1	15|17	0	0.62	0.88
ESS2	2|0	0	0.1	0.88
OSBPL11	7|5	0	0.44	0.88
SNRPN	7|7	0	0.74	0.88
VPS33A	2|2	0	0.4	0.88
KRI1	3|3	0	0.23	0.88
UPF3B	2|0	0	0.15	0.88
INTS3	2|0	0	0.22	0.88
PHACTR4	3|3	0	0.16	0.88
TRIR	2|2	0	0.11	0.88
NOC4L	5|7	0	0.44	0.88
C1orf35	0|2	0	0.14	0.88
EFHD2	8|8	0	0.33	0.88
CDC73	3|3	0	0.16	0.88
CORO7	3|0	0	0.2	0.88
SAP130	2|4	0	0.29	0.88
RPAP3	17|17	0	0.58	0.88
IPO4	9|10	0	0.36	0.88

NAT10	25|33	0	0.42	0.88
RABL6	7|6	0	0.47	0.88
CLMN	4|5	0	0.28	0.88
EHMT1	6|0	0	0.18	0.88
PIP4K2C	0|2	0	0.08	0.88
ATAD5	2|0	0	0.12	0.88
VCPIP1	40|41	0	0.72	0.88
TRABD	2|2	0	0.42	0.88
WDR82	6|5	0	0.22	0.88
DICER1	6|4	0	0.26	0.88
RAI1	0|7	0	0.16	0.88
TCP1	49|49	0	0.77	0.88
NRBF2	3|4	0	0.4	0.88
TDRD3	2|0	0	0.06	0.88
TRIM56	2|0	0	0.17	0.88
HNRNPK	26|25	0	0.37	0.88
CDK13	5|7	0	0.53	0.88
SF3B5	3|3	0	0.5	0.88
REPS1	3|2	0	0.16	0.88
EIF2A	4|5	0	0.29	0.88
BRIP1	4|5	0	0.21	0.88
USP48	2|0	0	0.12	0.88
MRI1	3|4	0	0.37	0.88
POLDIP3	5|4	0	0.3	0.88
WDR83	3|3	0	0.32	0.88
PYM1	2|2	0	0.24	0.88
BRMS1L	0|2	0	0.17	0.88
BAZ1B	25|25	0	0.32	0.88
TUBB6	56|54	0	0.64	0.88
GLYR1	4|4	0	0.16	0.88
PPP1R9B	2|2	0	0.24	0.88
PAPOLA	12|14	0	0.36	0.88
TUBA1C	104|94	0	0.49	0.88
JMJD1C	29|29	0	0.43	0.88
SYAP1	6|5	0	0.31	0.88
MASTL	2|2	0	0.13	0.88
LTV1	6|5	0	0.46	0.88
RBM17	4|4	0	0.15	0.88
GTF2I	24|25	0	0.45	0.88
RPS24	6|8	0	0.62	0.88
DIDO1	13|18	0	0.32	0.88
SARNP	3|3	0	0.43	0.88
CALD1	13|16	0	0.34	0.88
SURF4	3|5	0	0.52	0.88
LMF2	0|4	0	0.23	0.88

MICALL1	2|0	0	0.05	0.88
TNKS1BP1	7|6	0	0.25	0.88
CDK11B	7|6	0	0.32	0.88
LUZP1	14|14	0	0.5	0.88
NACC1	2|2	0	0.17	0.88
LARP4	5|4	0	0.45	0.88
GPRIN1	5|4	0	0.21	0.88
LENG8	4|10	0	0.37	0.88
RBM33	4|4	0	0.15	0.88
ASS1	19|18	0	0.61	0.88
FAM207A	2|0	0	0.07	0.88
BRD4	7|5	0	0.1	0.88
RNPS1	6|8	0	0.39	0.88
PRRC2A	35|34	0	0.56	0.88
ASNS	13|10	0	0.55	0.88
RAVER1	8|7	0	0.39	0.88
ATRX	4|6	0	0.09	0.88
KIF23	11|10	0	0.42	0.88
LEO1	3|3	0	0.24	0.88
SON	14|15	0	0.4	0.88
NAP1L1	7|6	0	0.27	0.88
TAF15	8|0	0	0.3	0.88
WDR36	8|7	0	0.23	0.88
TAF6	2|0	0	0.11	0.88
JPT2	6|5	0	0.42	0.88
ZC3H18	5|4	0	0.27	0.88
HNRNPUL1	6|6	0	0.39	0.88
ZFC3H1	2|2	0	0.42	0.88
SKA3	3|6	0	0.42	0.88
MAP4K4	8|8	0	0.67	0.88
POGZ	16|17	0	0.58	0.88
PALM2-AKAP2	7|6	0	0.41	0.88
PPIL2	4|5	0	0.5	0.88
CHMP7	4|4	0	0.43	0.88
TARS	6|5	0	0.31	0.88
C12orf45	2|2	0	0.42	0.88
ZNF362	2|0	0	0.09	0.88
DENND6A	2|0	0	0.27	0.88
TES	6|4	0	0.34	0.88
LRWD1	7|8	0	0.26	0.88
TIPRL	13|15	0	0.37	0.88
COG7	6|5	0	0.34	0.88
NUP50	5|4	0	0.22	0.88
KNL1	0|3	0	0.08	0.88
SUGP1	5|4	0	0.12	0.88

DIP2B	2|2	0	0.31	0.88
CCDC50	0|2	0	0.14	0.88
TJP1	40|44	0	0.76	0.88
CHMP4B	2|3	0	0.36	0.88
PPM1G	3|3	0	0.24	0.88
TUBB2B	101|94	0	0.75	0.88
TUBB	123|116	0	0.62	0.88
MTDH	8|8	0	0.25	0.88
TCF20	15|13	0	0.29	0.88
CUX1	6|5	0	0.11	0.88
SRPK2	0|2	0	0.12	0.88
RFLNB	2|2	0	0.47	0.88
EIF4G1	64|67	0	0.64	0.88
KLC1	12|13	0	0.46	0.88
ARHGEF11	0|2	0	0.16	0.88
PRMT1	2|0	0	0.13	0.88
G3BP1	12|12	0	0.44	0.88
ACACA	163|169	0	0.66	0.88
STAMBP	3|0	0	0.26	0.88
SF1	11|8	0	0.32	0.88
GIPC1	4|4	0	0.59	0.88
HDLBP	49|50	0	0.8	0.88
ELP5	2|2	0	0.33	0.88
GPS1	2|0	0	0.25	0.88
RAPH1	5|4	0	0.36	0.88

Supplementary Table 3: Crapome analysis of AP-MS and BioID data of TBC1D9

AP-MS		BioID	
**Gene Symbol**	**Num of Expt. (found/total)**	**Gene Symbol**	**Num of Expt. (found/total)**
PVR	0 / 411	NCKAP5L	0 / 411
FAM162A	0 / 411	COMMD3-BMI1	0 / 411
CCDC51	0 / 411	GPAA1	0 / 411
COQ9	0 / 411	AUTS2	0 / 411
PGS1	0 / 411	INTS8	0 / 411
ADPGK	0 / 411	SLC30A6	0 / 411
VWA8	1 / 411	N4BP2	0 / 411
LONP2	1 / 411	UQCC1	0 / 411
ELMOD2	1 / 411	TRAPPC11	0 / 411
MBLAC2	1 / 411	SLC30A5	0 / 411
EP300	2 / 411	KDELC1	0 / 411
GBA	2 / 411	THNSL1	0 / 411
OS9	2 / 411	STK11IP	0 / 411
KATNB1	2 / 411	GLI3	0 / 411
TBL1X	3 / 411	MAD2L2	0 / 411
RDH14	3 / 411	LATS2	0 / 411
SLC25A17	3 / 411	ABHD16A	0 / 411
PTGES2	3 / 411	TOR1A	1 / 411
PCYT1A	4 / 411	SLC25A19	1 / 411
TMEM43	4 / 411	TMED4	1 / 411
TPP1	4 / 411	TMEM11	1 / 411
CELF2	4 / 411	TRAPPC8	1 / 411
PREB	4 / 411	ARL13B	2 / 411
GGT7	4 / 411	CEP112	2 / 411
ANGEL2	5 / 411	CLCN7	2 / 411
PPOX	6 / 411	RAB34	2 / 411
DNAJC19	7 / 411	GTPBP2	2 / 411
CBX4	8 / 411	PLD1	3 / 411
RBM12B	8 / 411	PTK7	3 / 411
TRRAP	9 / 411	LAMTOR1	4 / 411
TLE1	9 / 411	KIF16B	4 / 411
ILVBL	9 / 411	HIRA	5 / 411
ARL8A	9 / 411	NARS2	5 / 411
RHOT2	9 / 411	LZTS2	5 / 411
CLPTM1L	10 / 411	GTPBP10	5 / 411
GSTK1	10 / 411	GLE1	6 / 411
SGPL1	10 / 411	CEP152	6 / 411
UFL1	11 / 411	NTHL1	6 / 411
COMT	12 / 411	MRPS11	7 / 411
APMAP	13 / 411	FDXR	7 / 411
CCDC59	13 / 411	SPATA18	7 / 411

ANK2	14 / 411	SAMM50	8 / 411
PLK1	14 / 411	RDH11	9 / 411
TRIM24	15 / 411	HAUS3	9 / 411
ACOT9	16 / 411	NAA16	9 / 411
ZMYM2	17 / 411	CCDC88C	10 / 411
LSG1	18 / 411	NFS1	10 / 411
NUSAP1	19 / 411	CEP192	10 / 411
SQOR		ACOT13	11 / 411
RIOX1		AGPS	11 / 411
RETREG3		TNS3	11 / 411
PYCR3		NNT	12 / 411
GPRIN1	24 / 411	FYTTD1	13 / 411
HMGXB4	25 / 411	SLC38A2	15 / 411
NOC2L	27 / 411	SNIP1	16 / 411
KNOP1	34 / 411	AGK	16 / 411
PGRMC2	35 / 411	DLG5	18 / 411
TAF6	36 / 411	IDH2	19 / 411
SUGP2	37 / 411	GATD3A	
PLRG1	38 / 411	RIOX1	
RAB13	38 / 411	DGLUCY	
RBBP6	39 / 411	PIP4P1	
RAB5A	42 / 411	UTP11	
SMCHD1	45 / 411	MCUB	
YLPM1	45 / 411	MAP3K21	
SMARCA4	59 / 411	TTLL12	21 / 411
EXOSC10	70 / 411	HNRNPLL	21 / 411
PHB	85 / 411	MRPS22	32 / 411
		RAB14	40 / 411
		HADHB	47 / 411
		RAB35	48 / 411
		APEX1	54 / 411
		SRSF10	71 / 411
		SHMT2	101 / 411
		U2AF1	127 / 411
		RUVBL2	141 / 411
		SRSF3	197 / 411

Supplementary Table 4: Metascape result of AP-MS and BioID in TBC1D9

AP-MS and BioID

**Metabolism of lipids**	**pvalue**	**Organelle localization**	**pvalue**
Metabolism of lipids	-5.6	Organelle localization	-3.8
Glycerophospholipid metabolic process	-3	Microtubule-based process	-3.5
Glycerolipid metabolic process	-2.4	Loss of Nlp from mitotic centrosomes	-3.4
phospholipid metabolic process	-2.3	Loss of proteins required for interphase microtubule organization from the centrosome	-3.4
		AURKA Activation by TPX2	-3.4
		Recruitment of mitotic centrosome proteins and complexes	-3.2
		Centrosome maturation	-3.2
		Regulation of PLK1 Activity at G2/M Transition	-3.1
		Recruitment of NuMA to mitotic centrosomes	-2.9
		Ciliary basal body-plasma membrane docking	-2.9
		Anchoring of the basal body to the plasma membrane	-2.9
		Microtubule cytoskeleton organization	-2.8
		Cilium Assembly	-2.6
		Centrosome cycle	-2.5
		Spindle assembly	-2.5
		Microtubule organizing center organization	-2.4
		Plasma membrane bounded cell projection assembly	-2.3
		Cell projection assembly	-2.2

**AP-MS**		**BioID**	
**Response to estrogen**	**pvalue**	**Inner mitochondrial membrane organization**	**pvalue**
Response to estrogen	-4.7	Inner mitochondrial membrane organization	-3.6
**PID BETA CATENIN NUC PATHWAY**		Mitochondrial biogenesis	-2.6
PID BETA CATENIN NUC PATHWAY	-4.5	Mitochondrial membrane organization	-2.2
Beta-catenin-TCF complex assembly	-4.4		
NOTCH1 Intracellular Domain Regulates Transcription	-3.8	**Regulation of smoothened signaling pathway**	
Regulation of protein stability	-3.4	Respiratory tube development	-2.9
Signaling by NOTCH1	-3.3	Epithelial tube morphogenesis	-2.7
Formation of the beta-catenin:TCF transactivating complex	-2.9	Respiratory system development	-2.7
Diseases of signal transduction	-2.8	Morphogenesis of an epithelium	-2.5
Protein stabilization	-2.2	Neural tube development	-2
**Response to endoplasmic reticulum stress**		Tissue morphogenesis	-2
Response to endoplasmic reticulum stress	-3.4	**Organelle biogenesis and maintenance**	

Endoplasmic reticulum unfolded protein response	-2.6	Organelle biogenesis and maintenance	-4.8
Cellular response to unfolded protein	-2.4	Inner mitochondrial membrane organization	-3.6
Cellular response to topologically incorrect protein	-2.3	Mitochondrial biogenesis	-2.9
Response to unfolded protein	-2.2	Mitochondrial membrane organization	-2.4
Response to topologically incorrect protein	-2		

Supplementary Table 5: AP-MS data for MFGE8 after SAINT analysis

AP-MS MFGE8-3xFLAG in Flp-In T-REx HEK293 cells. Preys with BFDR ≤ 1% are considered high-confidence and shown in Green here. Bait is 3xFLAG tagged MFGE8. Prey Gene is the Official Gene Symbol (from NCBI). Spectral counts for the prey (Spec, separated by "I" delimiter), Averaged probability across replicates (AvgP), Fold Change (counts in the purification divided by counts in the controls plus small factor to prevent division by 0) and Bayesian FDR (BFDR) are listed for each bait-prey relationship and are directly from the SAINTexpress output. 4 uncompressed untagged controls were employed in the SAINT analysis.The experimet was repeated twice.

PreyGene	Spec	AvgP	FoldChange	BFDR
YBA	3|5	0.99	40	0
GALK1	5|5	1	50	0
GCDH	3|3	0.98	30	0
NAGLU	4|3	0.99	35	0
PON2	8|8	1	80	0
PPT1	4|7	1	55	0
UMPS	3|4	0.99	35	0
PPIC	6|6	1	60	0
MED24	5|3	0.99	40	0
EARS2	6|5	1	55	0
ARSA	4|3	0.99	35	0
ASIC1	5|3	0.99	40	0
NUDT19	5|10	1	75	0
ECE1	15|10	1	125	0
PPOX	3|4	0.99	35	0
ERLEC1	10|7	1	85	0
CLGN	37|38	1	16.67	0
COCH	4|4	1	40	0
COMT	6|8	1	70	0
MGRN1	3|4	0.99	35	0
TCP11L1	3|3	0.98	30	0
GBA	5|3	0.99	40	0
ANXA6	5|7	1	60	0
ERAP1	4|5	1	45	0
SSR4	6|5	1	55	0
ATP7B	3|3	0.98	30	0
OS9	13|14	1	135	0
CTBP1	3|4	0.99	35	0
CANX	129|124	1	3.89	0
MMP15	7|7	1	70	0
MSI1	3|3	0.98	30	0
NME3	5|6	1	55	0
ALDH4A1	4|3	0.99	35	0

SUCLA2	3|4	0.99	35	0
GGH	4|3	0.99	35	0
FLOT2	8|10	1	90	0
SEL1L	20|19	1	4.88	0
ACTC1	33|35	1	340	0
LNPEP	5|4	1	45	0
TUBA4A	82|81	1	815	0
TUBB3	50|48	1	490	0
HAX1	13|12	1	12.5	0
DNAJC3	23|21	1	220	0
CDIPT	5|4	1	45	0
SLC39A7	3|4	0.99	35	0
ERLIN2	5|4	1	45	0
CNPY2	4|5	1	45	0
FAF2	4|3	0.99	35	0
IPO13	10|9	1	95	0
TTI1	12|12	1	120	0
KIAA0355	3|4	0.99	35	0
ERP44	5|6	1	55	0
SMCHD1	3|4	0.99	35	0
NCSTN	6|5	1	55	0
POFUT1	3|3	0.98	30	0
MAN1B1	8|5	1	65	0
RAB6B	6|5	1	55	0
LRRC1	5|7	1	60	0
NGLY1	3|4	0.99	35	0
RHOT1	5|4	1	45	0
HLA-F	5|4	1	45	0
TMX3	18|19	0.99	3.7	0
NDUFB11	3|3	0.98	30	0
MYDGF	3|4	0.99	35	0
ALG1	8|8	1	80	0
UGGT1	43|40	1	7.22	0
UGGT2	20|22	1	210	0
UBQLN4	5|5	1	50	0
VARS2	14|14	1	28	0
APMAP	6|6	1	60	0
TMX4	3|3	0.98	30	0
SCPEP1	4|4	1	40	0
ITM2B	4|3	0.99	35	0
SIL1	8|5	1	65	0
C16orf58	3|3	0.98	30	0
KDELC1	11|10	1	105	0
SLC27A3	4|3	0.99	35	0
HPS6	5|5	1	50	0

L2HGDH	3|3	0.98	30	0
TRABD	3|4	0.99	35	0
ADPGK	11|9	1	100	0
FAM234A	4|3	0.99	35	0
FAR1	7|3	0.99	50	0
KLHL22	7|6	1	65	0
PIGS	4|5	1	45	0
CHST14	6|3	0.99	45	0
RHOT2	5|3	0.99	40	0
SLC39A11	3|3	0.98	30	0
TYSND1	3|3	0.98	30	0
GGT7	5|5	1	50	0
TOR1A	3|2	0.95	25	0.01
GAA	2|3	0.95	25	0.01
GGCX	3|2	0.95	25	0.01
PKP2	3|2	0.95	25	0.01
FAM69A	2|3	0.95	25	0.01
IRGQ	2|4	0.95	30	0.01
C6orf120	4|2	0.95	30	0.01
ACOT1	2|5	0.95	35	0.01
PNKD	3|2	0.95	25	0.01
FNDC3A	3|2	0.95	25	0.01
RBFOX2	3|2	0.95	25	0.01
TEX264	2|3	0.95	25	0.01
ZNF507	3|2	0.95	25	0.01
ACOT13	2|3	0.95	25	0.01
CACNA2D2	3|2	0.95	25	0.01
CES3	3|2	0.95	25	0.01
HSDL2	2|4	0.95	30	0.01
HLA-C	21|19	0.97	8	0.01
ABHD10	2|3	0.95	25	0.01
GPC4	3|2	0.95	25	0.01
FBLN1	3|2	0.95	25	0.01
MPI	2|5	0.95	35	0.01
EXOSC10	4|2	0.95	30	0.01
RAF1	8|6	0.96	14	0.01
CXCR4	2|3	0.95	25	0.01
NRP1	2|3	0.95	25	0.01
TRIP10	2|3	0.95	25	0.01
ERAL1	2|3	0.95	25	0.01
DTYMK	2|3	0.95	25	0.01
MED4	3|2	0.95	25	0.01
P3H3	3|2	0.95	25	0.01
YTHDF2	3|2	0.95	25	0.01
TTC19	2|3	0.95	25	0.01

TMEM165	2|3	0.95	25	0.01
CBWD1	3|2	0.95	25	0.01
UBFD1	2|5	0.95	35	0.01
RARS2	4|2	0.95	30	0.01
HEATR6	13|13	0.97	8.67	0.01
DDX54	6|2	0.95	40	0.01
TTC13	6|2	0.95	40	0.01
CARS2	2|4	0.95	30	0.01
TTI2	4|2	0.95	30	0.01
EDEM3	3|2	0.95	25	0.01
GINS4	2|3	0.95	25	0.01
HDHD5	9|10	0.96	7.6	0.01
EHD4	2|4	0.95	30	0.01
SNX5	2|3	0.95	25	0.01
RPS19BP1	2|3	0.95	25	0.01
MBLAC2	2|3	0.95	25	0.01
DLD	5|7	0.92	12	0.02
LPL	2|2	0.91	20	0.02
RBPMS	2|2	0.91	20	0.02
NECTIN2	2|2	0.91	20	0.02
GCAT	2|2	0.91	20	0.02
ARL1	12|11	0.94	3.83	0.02
ATP6AP1	2|2	0.91	20	0.02
LSR	5|7	0.92	12	0.02
HSPB1	2|2	0.91	20	0.02
COL6A1	7|5	0.92	12	0.02
HLA-A	16|14	0.94	4.62	0.02
M6PR	2|2	0.91	20	0.02
PAFAH1B2	2|2	0.91	20	0.02
STC2	2|2	0.91	20	0.02
BECN1	2|2	0.91	20	0.02
PSPH	2|2	0.91	20	0.02
VAMP3	2|2	0.91	20	0.02
MED16	2|2	0.91	20	0.02
PPP2R5E	2|2	0.91	20	0.02
B4GAT1	7|5	0.92	12	0.02
RALY	2|2	0.91	20	0.02
SUMF2	2|2	0.91	20	0.02
APIP	2|2	0.91	20	0.02
FIS1	2|2	0.91	20	0.02
TELO2	15|13	0.92	5.09	0.02
NDRG2	2|2	0.91	20	0.02
VTA1	2|2	0.91	20	0.02
PRMT6	2|2	0.91	20	0.02
SH3BP1	2|2	0.91	20	0.02

INTS2	2|2	0.91	20	0.02
XRCC4	2|2	0.91	20	0.02
DDRGK1	2|2	0.91	20	0.02
NABP2	2|2	0.91	20	0.02
FASTKD1	2|2	0.91	20	0.02
RIOX1	2|2	0.91	20	0.02
TSC22D4	2|2	0.91	20	0.02
NIFK	2|2	0.91	20	0.02
ACSS1	2|2	0.91	20	0.02
TMEM209	2|2	0.91	20	0.02
BRI3BP	2|2	0.91	20	0.02
GTPBP3	2|2	0.91	20	0.02
KDELC2	9|10	0.91	6.33	0.02
EPHX4	2|2	0.91	20	0.02
RETREG3	2|2	0.91	20	0.02
RILPL1	2|2	0.91	20	0.02
PRCP	2|2	0.91	20	0.02
GDI1	18|19	0.9	4.11	0.04
IDH2	4|5	0.82	9	0.04
NDUFA9	5|4	0.82	9	0.04
ATP6AP2	9|8	0.85	5.67	0.04
SPTLC1	10|8	0.87	6	0.04
PALD1	6|5	0.9	11	0.04
PMPCA	6|5	0.9	11	0.04
GEMIN4	14|14	0.91	6.22	0.04
COQ8B	9|9	0.89	6	0.04
NUP210	14|10	0.86	6	0.04
HEXA	6|6	0.8	8	0.05
GSTP1	5|8	0.81	8.67	0.05
POR	3|6	0.76	9	0.05
HADH	3|8	0.78	11	0.05
PGM3	4|9	0.76	8.67	0.05
ENO2	4|10	0.77	9.33	0.05
EMC1	10|8	0.77	5.14	0.05
UBXN7	7|8	0.76	3.75	0.05
PGM2	3|6	0.76	9	0.05
TARS2	3|6	0.76	9	0.05
ASS1	3|6	0.76	9	0.05
CYB5A	6|7	0.8	4.33	0.05
AKR1A1	3|7	0.77	10	0.05
OXCT1	13|13	0.73	3.06	0.06
HEXB	8|7	0.72	3.75	0.06
UBQLN1	5|5	0.71	6.67	0.06
ESYT2	14|11	0.74	5	0.06
PCYOX1L	6|7	0.72	6.5	0.06

HK1	18|17	0.71	2.8	0.06
TOR1AIP2	9|9	0.73	5.14	0.06
CTSB	9|7	0.75	5.33	0.06
PRKAA1	3|5	0.73	8	0.06
ALDH1B1	4|10	0.69	7	0.07
SLC39A10	8|6	0.68	4.67	0.07
OCLN	7|7	0.7	4.67	0.07
RAD23A	9|9	0.68	4.5	0.07
ARF1	11|13	0.69	3.43	0.07
XPOT	11|11	0.71	3.14	0.07
FASTKD5	6|6	0.69	4.8	0.07
MAP2K2	6|9	0.68	5	0.07
ADD2	3|4	0.67	7	0.08
TOR1AIP1	3|4	0.67	7	0.08
MTMR1	4|3	0.67	7	0.08
OSGEP	3|4	0.67	7	0.08
HEATR1	4|3	0.67	7	0.08
TXNDC16	4|3	0.67	7	0.08
DNAJC1	3|4	0.67	7	0.08
TUBB2A	66|62	0.62	4.06	0.09
SLC1A3	5|4	0.62	4.5	0.09
DNAJA3	7|8	0.63	4.29	0.09
TMED2	4|5	0.62	4.5	0.09
RAB35	5|4	0.65	6	0.09
CLIC4	3|7	0.65	6.67	0.09
MMAB	4|5	0.65	6	0.09
CPT2	8|6	0.61	3.5	0.1
SLC7A5	3|3	0.58	6	0.1
VPS26C	3|3	0.58	6	0.1
TBL3	3|3	0.58	6	0.1
CHP1	3|3	0.58	6	0.1
CAVIN1	3|3	0.58	6	0.1
RSL1D1	2|5	0.61	7	0.1
WDR18	3|3	0.58	6	0.1
NDUFS7	3|3	0.58	6	0.1
MAIP1	3|3	0.58	6	0.1
NAT10	3|3	0.58	6	0.1
NIPBL	3|3	0.58	6	0.1
PPP1R8	3|6	0.57	4.5	0.11
PCYOX1	8|11	0.57	3.17	0.11
MASTL	6|3	0.57	4.5	0.11
GLB1	2|4	0.56	6	0.12
TXNDC5	5|8	0.56	4.33	0.12
MAP2K1	7|11	0.56	3.27	0.12
SART1	7|4	0.57	4.4	0.12

CKAP4	2|4	0.56	6	0.12
CTBP2	2|4	0.56	6	0.12
ATP2A2	42|31	0.57	2.98	0.12
TMEM65	4|2	0.56	6	0.12
SAR1A	4|5	0.53	4.5	0.13
SERPINH1	12|17	0.55	3.62	0.13
MKI67	8|9	0.53	3.4	0.13
HINT1	6|9	0.54	3.33	0.13
PRDX5	5|7	0.53	3.43	0.13
QSOX2	11|7	0.54	3.27	0.13
PLOD2	8|6	0.52	4	0.13
P4HA2	4|4	0.5	4	0.14
VAPB	4|4	0.5	4	0.14
ILVBL	4|3	0.51	4.67	0.14
SDF2L1	12|10	0.52	2.93	0.14
BRAT1	3|8	0.51	3.67	0.14
HAGH	0|5	0.5	25	0.15
POTEE	10|0	0.5	50	0.15
RAB1A	7|0	0.5	35	0.15
DDX39A	0|11	0.5	55	0.15
SEC61A1	5|0	0.5	25	0.15
IWS1	5|8	0.5	3.25	0.15
SAR1B	0|4	0.5	20	0.16
C17orf49	0|4	0.5	20	0.16
FLAD1	0|4	0.5	20	0.16
RAB13	0|4	0.5	20	0.16
PCK2	0|4	0.5	20	0.16
NUBP2	4|0	0.5	20	0.16
PRRC2B	0|4	0.5	20	0.16
DYNC2LI1	0|4	0.5	20	0.16
VPS36	0|4	0.5	20	0.16
DYM	4|0	0.5	20	0.16
RBM23	4|0	0.5	20	0.16
ZC3HAV1	0|4	0.5	20	0.16
FDXR	0|4	0.5	20	0.16
SMC6	4|0	0.5	20	0.16
CDK13	4|0	0.5	20	0.16
SURF4	4|0	0.5	20	0.16
NT5DC1	0|4	0.5	20	0.16
CP	0|3	0.49	15	0.17
DNAJB12	3|0	0.49	15	0.17
MTFR2	3|0	0.49	15	0.17
GNE	0|3	0.49	15	0.17
SDK2	3|0	0.49	15	0.17
DTNA	3|0	0.49	15	0.17

ENTPD6	3|0	0.49	15	0.17
RAB5B	0|3	0.49	15	0.17
ADAM15	3|0	0.49	15	0.17
EP300	0|3	0.49	15	0.17
PCDH7	3|0	0.49	15	0.17
PREP	0|3	0.49	15	0.17
RPS6KA1	0|3	0.49	15	0.17
UAP1	0|3	0.49	15	0.17
RAB5A	3|0	0.49	15	0.17
FADS2	3|0	0.49	15	0.17
DVL3	0|3	0.49	15	0.17
PABPN1	0|3	0.49	15	0.17
RRP9	0|3	0.49	15	0.17
KATNB1	0|3	0.49	15	0.17
MAN1A1	0|3	0.49	15	0.17
UCHL3	0|3	0.49	15	0.17
CELF2	0|3	0.49	15	0.17
PGLS	0|3	0.49	15	0.17
GRHPR	0|3	0.49	15	0.17
PPM1F	0|3	0.49	15	0.17
SUGP2	0|3	0.49	15	0.17
UBE2J1	3|0	0.49	15	0.17
CWC15	0|3	0.49	15	0.17
BORCS6	0|3	0.49	15	0.17
OXR1	0|3	0.49	15	0.17
RAP2C	3|0	0.49	15	0.17
GPRC5C	3|0	0.49	15	0.17
DDX50	0|3	0.49	15	0.17
COQ5	3|0	0.49	15	0.17
SLC39A3	3|0	0.49	15	0.17
LPCAT4	0|3	0.49	15	0.17
P4HTM	3|0	0.49	15	0.17
PDCD4	3|5	0.49	4	0.21
ERO1A	5|8	0.48	3.25	0.21
SCCPDH	2|5	0.48	4.67	0.21
PM20D2	3|7	0.48	4	0.22
ADAM10	3|2	0.47	5	0.22
PARN	2|3	0.47	5	0.22
SMS	2|3	0.47	5	0.22
MICU1	3|2	0.47	5	0.22
ARPP19	3|2	0.47	5	0.22
AKAP8L	2|3	0.47	5	0.22
UBE2S	3|2	0.47	5	0.22
DPH5	2|3	0.47	5	0.22
ZC3HC1	3|2	0.47	5	0.22

EMD	9|10	0.46	2.92	0.23
GLA	2|0	0.45	10	0.23
PEPD	0|2	0.45	10	0.23
TPP1	2|0	0.45	10	0.23
EXT2	2|0	0.45	10	0.23
CACNA2D1	0|2	0.45	10	0.23
DHFR	0|2	0.45	10	0.23
RGS10	2|0	0.45	10	0.23
PKD1	2|0	0.45	10	0.23
GOPC	2|0	0.45	10	0.23
CDC42SE2	0|2	0.45	10	0.23
PGP	0|2	0.45	10	0.23
TOP2A	2|0	0.45	10	0.23
MIER1	0|2	0.45	10	0.23
FAM177A1	0|2	0.45	10	0.23
TOR2A	2|0	0.45	10	0.23
SZRD1	0|2	0.45	10	0.23
GINS3	0|2	0.45	10	0.23
PPP3CA	0|2	0.45	10	0.23
TMEM106B	2|0	0.45	10	0.23
FERMT2	0|2	0.45	10	0.23
AGFG1	0|2	0.45	10	0.23
APLP2	2|0	0.45	10	0.23
SIRT1	2|0	0.45	10	0.23
GSTK1	0|2	0.45	10	0.23
TMEM30A	2|0	0.45	10	0.23
CCDC168	0|2	0.45	10	0.23
PALLD	0|2	0.45	10	0.23
TPBG	2|0	0.45	10	0.23
MPP1	0|2	0.45	10	0.23
ABL2	0|2	0.45	10	0.23
RIF1	20|24	0.47	2.67	0.23
NRCAM	0|2	0.45	10	0.23
PI4KB	0|2	0.45	10	0.23
SUN2	2|0	0.45	10	0.23
NDUFS1	2|0	0.45	10	0.23
PLRG1	0|2	0.45	10	0.23
CD99L2	2|0	0.45	10	0.23
FHL3	2|0	0.45	10	0.23
TRA2B	0|2	0.45	10	0.23
DDX19B	0|2	0.45	10	0.23
MLH1	0|2	0.45	10	0.23
DPH2	0|2	0.45	10	0.23
FABP5	0|2	0.45	10	0.23
GLE1	0|2	0.45	10	0.23

CBFB	0|2	0.45	10	0.23
CTSC	2|0	0.45	10	0.23
COX5B	0|2	0.45	10	0.23
CPE	2|0	0.45	10	0.23
CTH	0|2	0.45	10	0.23
DLST	0|2	0.45	10	0.23
ACSL1	0|2	0.45	10	0.23
GSK3B	0|2	0.45	10	0.23
HLA-DQB1	2|0	0.45	10	0.23
LRP1	0|2	0.45	10	0.23
MAGOH	5|6	0.46	3.67	0.23
MTX1	0|2	0.45	10	0.23
NDUFS8	2|0	0.45	10	0.23
PHB	2|0	0.45	10	0.23
PTK7	2|0	0.45	10	0.23
PTPN9	0|2	0.45	10	0.23
UQCRC1	0|2	0.45	10	0.23
PDHX	0|2	0.45	10	0.23
PDXK	0|6	0.47	6	0.23
TRIM24	0|2	0.45	10	0.23
DPM1	2|0	0.45	10	0.23
TIMM8A	0|2	0.45	10	0.23
KDM5C	2|0	0.45	10	0.23
EFNB1	0|2	0.45	10	0.23
MOCS2	0|2	0.45	10	0.23
PFKFB4	2|0	0.45	10	0.23
RPS6KA3	0|6	0.47	6	0.23
STX4	0|2	0.45	10	0.23
OXA1L	0|2	0.45	10	0.23
PWP2	0|2	0.45	10	0.23
OXSR1	0|2	0.45	10	0.23
CRK	0|2	0.45	10	0.23
ARID3A	0|2	0.45	10	0.23
LOXL1	2|0	0.45	10	0.23
REEP5	0|2	0.45	10	0.23
UQCRFS1	0|2	0.45	10	0.23
BPNT1	0|2	0.45	10	0.23
DRAP1	2|0	0.45	10	0.23
LMAN2	2|0	0.45	10	0.23
TAF5	0|2	0.45	10	0.23
SLC39A6	2|0	0.45	10	0.23
NENF	0|2	0.45	10	0.23
CCDC59	2|0	0.45	10	0.23
MAPRE2	0|2	0.45	10	0.23
HEBP2	0|2	0.45	10	0.23

HIBCH	0|2	0.45	10	0.23
EHD3	0|2	0.45	10	0.23
AQR	2|0	0.45	10	0.23
EPM2AIP1	0|2	0.45	10	0.23
ZC3H11A	0|2	0.45	10	0.23
SV2A	2|0	0.45	10	0.23
RFTN1	2|0	0.45	10	0.23
NOC2L	0|2	0.45	10	0.23
METTL9	2|0	0.45	10	0.23
DHRS7	2|0	0.45	10	0.23
ISOC1	0|2	0.45	10	0.23
EVL	2|0	0.45	10	0.23
NSMCE4A	0|2	0.45	10	0.23
QRICH1	2|0	0.45	10	0.23
TEX10	0|2	0.45	10	0.23
WDR60	2|0	0.45	10	0.23
APPL2	0|2	0.45	10	0.23
C1orf112	2|0	0.45	10	0.23
NUSAP1	2|0	0.45	10	0.23
CHPF2	2|0	0.45	10	0.23
AGPAT4	2|0	0.45	10	0.23
PDXP	0|2	0.45	10	0.23
DDX24	0|2	0.45	10	0.23
DCUN1D1	0|2	0.45	10	0.23
TRMT5	0|2	0.45	10	0.23
METTL14	0|2	0.45	10	0.23
MPST	0|2	0.45	10	0.23
SPCS3	2|0	0.45	10	0.23
RIC8A	2|0	0.45	10	0.23
PC	0|2	0.45	10	0.23
C17orf75	2|0	0.45	10	0.23
RAB3IP	0|2	0.45	10	0.23
UPF3A	2|0	0.45	10	0.23
CHPF	2|0	0.45	10	0.23
GFM1	0|2	0.45	10	0.23
SUN1	0|2	0.45	10	0.23
PTER	0|2	0.45	10	0.23
CLPTM1L	2|0	0.45	10	0.23
APOL2	2|0	0.45	10	0.23
ITM2C	2|0	0.45	10	0.23
BBS2	2|0	0.45	10	0.23
FUCA2	2|0	0.45	10	0.23
AGPAT1	2|0	0.45	10	0.23
ATP5MD	2|0	0.45	10	0.23
ITPA	0|2	0.45	10	0.23

MGME1	0|2	0.45	10	0.23
GPRIN1	2|0	0.45	10	0.23
RDH13	0|2	0.45	10	0.23
NAXE	0|2	0.45	10	0.23
UBLCP1	0|2	0.45	10	0.23
NSMCE1	2|0	0.45	10	0.23
FAM69B	2|0	0.45	10	0.23
CKAP2L	2|0	0.45	10	0.23
ELMOD2	0|2	0.45	10	0.23
UBR3	0|2	0.45	10	0.23
FUT11	2|0	0.45	10	0.23
NNT	2|0	0.45	10	0.23
ZMYND8	2|0	0.45	10	0.23
GALNT18	2|0	0.45	10	0.23
SDF4	14|11	0.45	2.78	0.32
BCCIP	2|7	0.45	3	0.32
BZW2	3|3	0.43	4	0.33
DCXR	3|7	0.41	2.86	0.33
ADK	0|5	0.44	5	0.33
PPME1	2|4	0.42	4	0.33
ACADSB	3|4	0.4	3.5	0.33
AKR1B1	4|10	0.43	3.11	0.33
CAPNS1	3|3	0.43	4	0.33
PSMD9	3|3	0.43	4	0.33
GTPBP1	0|5	0.44	5	0.33
HLA-B	12|0	0.41	2.67	0.33
NAMPT	7|13	0.44	3.64	0.33
ARL6IP5	3|4	0.4	3.5	0.33
HARS2	3|3	0.43	4	0.33
HACL1	2|4	0.42	4	0.33
AASDHPPT	3|3	0.43	4	0.33
DNAJB11	17|17	0.42	2.72	0.33
WDR1	3|8	0.41	3.67	0.33
DCP1A	0|5	0.44	5	0.33
FAM114A2	3|3	0.43	4	0.33
NANS	4|9	0.42	2.6	0.33
PUS7	0|5	0.44	5	0.33
INTS3	3|4	0.4	3.5	0.33
DCAKD	3|3	0.43	4	0.33
HEATR3	2|4	0.42	4	0.33
G6PD	5|11	0.4	3.56	0.34
ACO2	11|17	0.39	2.43	0.34
GXYLT1	5|5	0.36	3.33	0.34
MAPK3	0|6	0.4	4	0.34
HMOX2	3|4	0.38	3.5	0.34

CAB39	4|0	0.38	4	0.34
CTPS2	2|2	0.36	4	0.34
SLC25A4	0|13	0.36	2.36	0.34
PHF8	2|2	0.36	4	0.34
DHPS	3|4	0.38	3.5	0.34
MIA2	2|2	0.36	4	0.34
DDX56	2|2	0.36	4	0.34
CRMP1	4|6	0.37	3.33	0.34
ARF6	2|2	0.36	4	0.34
IVD	0|6	0.4	4	0.34
CDS2	2|2	0.36	4	0.34
GMPS	13|20	0.37	2.36	0.34
SELENOF	2|2	0.36	4	0.34
SPTLC2	4|3	0.38	3.5	0.34
ALDH6A1	0|4	0.38	4	0.34
NEDD8	0|4	0.38	4	0.34
SLBP	2|2	0.36	4	0.34
PLPBP	0|4	0.38	4	0.34
TIMM8B	2|2	0.36	4	0.34
FAM3C	2|2	0.36	4	0.34
NUP188	10|8	0.36	2.77	0.34
SPNS1	4|3	0.38	3.5	0.34
CNP	10|12	0.38	2.75	0.34
TTC5	0|4	0.38	4	0.34
TES	6|6	0.4	3.43	0.34
RPL32	3|2	0.34	3.33	0.35
S100A10	2|3	0.34	3.33	0.35
SLC25A11	5|0	0.35	3.33	0.35
ARFGEF1	6|6	0.34	3	0.35
FAM169A	6|7	0.35	3.25	0.35
CNDP2	2|3	0.34	3.33	0.35
PLBD2	2|3	0.34	3.33	0.35
SULT1A1	5|8	0.31	2.6	0.36
PHB2	6|3	0.3	3	0.36
C4A	4|4	0.31	3.2	0.36
UBE2V1	6|3	0.31	3	0.36
IRAK1	4|4	0.31	3.2	0.36
SUCLG1	4|4	0.31	3.2	0.36
NDUFS2	12|9	0.34	2.62	0.36
PRDX4	5|4	0.34	3	0.36
DNPH1	2|4	0.31	3	0.36
AHCYL1	3|3	0.32	3	0.36
PLD3	6|5	0.32	3.14	0.36
CAND2	8|6	0.31	3.11	0.36
UBXN1	3|5	0.33	3.2	0.36

SMG9	3|6	0.3	3	0.36
METTL3	3|7	0.3	2.86	0.36
COQ8A	6|7	0.32	2.89	0.36
ZDBF2	3|3	0.32	3	0.36
PTGES2	4|4	0.31	3.2	0.36
KRT18	14|10	0.34	2.53	0.36
C18orf25	0|3	0.29	3	0.37
ORC4	2|4	0.29	3	0.37
NFS1	3|6	0.29	2.57	0.37
GNS	2|4	0.29	3	0.37
PRKAR2B	0|4	0.29	2.67	0.37
PKN1	0|3	0.29	3	0.37
EIF4EBP1	0|3	0.29	3	0.37
DPP3	0|4	0.29	2.67	0.37
ASF1A	0|3	0.29	3	0.37
TMA16	3|0	0.29	3	0.37
PTPA	0|4	0.29	2.67	0.37
ZNF703	0|3	0.29	3	0.37
HIBADH	0|3	0.29	3	0.37
PPA2	0|3	0.29	3	0.37
SLFN11	14|16	0.28	3	0.38
RAB2A	4|5	0.28	3	0.38
RAB6A	6|5	0.25	2.75	0.38
CUL5	2|2	0.25	2.67	0.38
NDUFA5	5|5	0.27	2.86	0.38
DOPEY2	5|7	0.29	3	0.38
PITPNB	4|6	0.25	2.86	0.38
STOML2	4|5	0.28	3	0.38
TKFC	0|4	0.25	2	0.38
ARFGAP1	5|6	0.25	2.75	0.38
GSK3A	4|5	0.28	3	0.38
RAB18	3|3	0.25	3	0.38
CCNT2	2|2	0.25	2.67	0.38
ARID2	0|5	0.26	2	0.38
HK2	4|3	0.23	2.8	0.39
STK24	4|7	0.25	2.75	0.39
TENT4B	3|0	0.22	2	0.39
IMMT	4|3	0.23	2.8	0.39
CYP51A1	3|4	0.23	2.8	0.39
GTF3C3	4|3	0.23	2.8	0.39
GMDS	3|0	0.22	2	0.39
GNAI1	3|0	0.22	2	0.39
SLC27A2	3|0	0.22	2	0.39
PPIH	0|3	0.22	2	0.39
UBAC1	4|3	0.23	2.8	0.39

UFC1	0|3	0.22	2	0.39
SBNO1	2|3	0.24	2.5	0.39
UBA6	3|2	0.24	2.5	0.39
YLPM1	0|3	0.22	2	0.39
CIP2A	2|3	0.24	2.5	0.39
VPS25	0|3	0.22	2	0.39
SCOC	0|3	0.22	2	0.39
TCEAL3	3|0	0.22	2	0.39
STAT1	2|5	0.23	2.33	0.39
CDC42EP1	3|0	0.22	2	0.39
GPD2	0|5	0.18	1.67	0.4
NEDD1	4|4	0.19	2.67	0.4
ARHGDIA	5|9	0.19	2.33	0.4
FSCN1	4|9	0.21	2.36	0.4
PMPCB	6|10	0.21	2.29	0.4
ERH	6|5	0.21	2.75	0.4
ASH2L	5|2	0.19	2.33	0.4
CRKL	4|9	0.19	2.17	0.4
SNX9	0|5	0.18	1.67	0.4
TBRG4	6|7	0.2	2.6	0.4
UROD	0|2	0.18	2	0.41
CARHSP1	0|2	0.18	2	0.41
TYMS	0|2	0.18	2	0.41
THOC6	0|2	0.18	2	0.41
FMR1	2|0	0.18	2	0.41
CDKN2A	2|4	0.18	2.4	0.41
SCARB2	2|0	0.18	2	0.41
PRKAG1	2|4	0.18	2.4	0.41
INTS14	2|0	0.18	2	0.41
PPP6R2	2|0	0.18	2	0.41
ARL2	2|4	0.18	2.4	0.41
PFDN1	2|4	0.18	2.4	0.41
PFDN4	2|0	0.18	2	0.41
SLC7A1	2|0	0.18	2	0.41
STX7	2|0	0.18	2	0.41
RNGTT	0|2	0.18	2	0.41
TIMELESS	0|2	0.18	2	0.41
SLC9A3R1	2|4	0.18	2.4	0.41
DDOST	2|4	0.18	2.4	0.41
LAMP1	2|0	0.18	2	0.41
BCAS2	0|2	0.18	2	0.41
F8A1	0|2	0.18	2	0.41
NTMT1	2|0	0.18	2	0.41
SUZ12	0|2	0.18	2	0.41
DPCD	0|2	0.18	2	0.41

GTSE1	4|2	0.18	2.4	0.41
MPP6	0|2	0.18	2	0.41
PNPO	0|2	0.18	2	0.41
PI4K2A	2|0	0.18	2	0.41
ELP4	0|2	0.18	2	0.41
ARFGEF3	2|0	0.18	2	0.41
AARS2	0|5	0.18	1.67	0.41
DTNB	0|2	0.18	2	0.41
SMAP2	0|2	0.18	2	0.41
SNX27	0|2	0.18	2	0.41
ARPC5L	2|0	0.18	2	0.41
PDCD2L	2|0	0.18	2	0.41
FKBP1A	0|2	0.18	2	0.41
BRD4	2|0	0.18	2	0.41
DCP1B	0|2	0.18	2	0.41
SPC24	0|2	0.18	2	0.41
GPATCH4	2|0	0.18	2	0.41
ERICH1	0|2	0.18	2	0.41
MCRIP1	0|2	0.18	2	0.41
ARFIP1	0|4	0.16	1.6	0.44
BCAP31	3|0	0.16	1.5	0.44
LSS	3|2	0.17	2.5	0.44
DRG2	0|4	0.16	1.6	0.44
RAD23B	10|8	0.16	2.4	0.44
SNU13	2|3	0.17	2.5	0.44
ALDOC	0|4	0.16	1.6	0.44
SLC1A5	3|2	0.17	2.5	0.44
STK4	0|3	0.16	1.5	0.44
SUMO2	2|3	0.17	2.5	0.44
CSTF2T	7|9	0.16	2.67	0.44
EHBP1	3|2	0.17	2.5	0.44
MOB4	3|2	0.17	2.5	0.44
PSIP1	6|9	0.16	2.31	0.44
SKA3	3|2	0.17	2.5	0.44
RPL3	3|5	0.14	2.29	0.45
SMARCAD1	2|0	0.12	1.33	0.45
FDPS	5|5	0.14	2.5	0.45
ALG5	0|2	0.12	1.33	0.45
ARAF	4|3	0.13	2.33	0.45
MTAP	3|5	0.14	2.29	0.45
CHMP1A	0|2	0.12	1.33	0.45
PIP4K2B	0|2	0.12	1.33	0.45
NIPSNAP1	7|8	0.15	2.5	0.45
SHMT1	3|3	0.14	2.4	0.45
ARFGEF2	2|0	0.12	1.33	0.45

SEC61B	0|2	0.12	1.33	0.45
MLEC	2|0	0.12	1.33	0.45
RRP1B	3|5	0.15	2.29	0.45
AGK	2|0	0.12	1.33	0.45
CCDC47	4|3	0.13	2.33	0.45
OSBPL8	2|0	0.12	1.33	0.45
GGCT	0|2	0.12	1.33	0.45
TMX1	12|12	0.14	2.53	0.45
CCNB1	0|2	0.12	1.33	0.45
RPL13	4|3	0.14	2.33	0.45
CASP7	0|2	0.12	1.33	0.45
ADH5	0|3	0.12	1.5	0.46
FHL1	0|3	0.12	1.5	0.46
RRP1	3|0	0.12	1.5	0.46
TSFM	0|3	0.12	1.5	0.46
SNTB2	0|3	0.12	1.5	0.46
COMMD9	3|0	0.12	1.5	0.46
ADNP	0|3	0.12	1.5	0.46
C3	3|0	0.11	1.2	0.47
MUT	3|6	0.1	2	0.47
RPL9	6|7	0.1	2.36	0.47
COASY	3|4	0.12	2.33	0.47
VDAC3	0|3	0.11	1.2	0.47
LTA4H	4|9	0.12	1.86	0.47
CDC42	2|4	0.1	2	0.47
SP1	0|4	0.09	1.33	0.47
UBA3	2|5	0.12	2	0.47
PPID	2|4	0.11	2	0.47
NCLN	4|0	0.09	1.33	0.47
TP53	2|2	0.08	2	0.48
RPL7	2|3	0.09	2	0.48
EPHX1	5|4	0.09	2.25	0.48
LPP	6|8	0.09	2.33	0.48
GLS	5|7	0.08	2.18	0.48
NIPSNAP2	2|2	0.08	2	0.48
NDUFA4	2|2	0.08	2	0.48
NOP14	2|2	0.08	2	0.48
ILK	2|2	0.08	2	0.48
PPP1R2	2|2	0.08	2	0.48
LRRC41	4|5	0.09	2.25	0.48
SDF2	2|2	0.08	2	0.48
ATP5IF1	2|2	0.08	2	0.48
NOC4L	9|7	0.09	2.29	0.48
PDGFA	2|2	0.08	2	0.48
LSM12	2|2	0.08	2	0.48

RFC5	4|4	0.09	2.29	0.48
NELFCD	2|3	0.09	2	0.48
BLVRB	0|2	0.07	1	0.49
TPI1	6|8	0.07	2.15	0.49
TUBGCP2	13|13	0.08	2.36	0.49
CFDP1	0|2	0.07	1	0.49
PLXNB2	5|3	0.08	2	0.49
ESF1	5|3	0.08	2	0.49
XPO4	3|5	0.07	2	0.49
NOTCH2	3|3	0.07	2	0.49
MAP7D2	3|4	0.07	2	0.49
LCP1	10|15	0.07	2.38	0.5
ME2	8|9	0.07	2.43	0.5
TFRC	3|3	0.06	2	0.5
ALDH1A2	5|10	0.06	1.88	0.5
SACM1L	3|0	0.07	1.2	0.5
GNL3	0|3	0.07	1.2	0.5
PPIP5K2	0|3	0.07	1.2	0.5
BCAS3	3|3	0.06	2	0.5
RNPEP	0|7	0.06	1.27	0.5
CKMT1B	0|3	0.07	1.2	0.5
PFN2	3|4	0.06	2	0.5
HIST1H2AA	4|6	0.06	2	0.5
RPLP1	2|3	0.05	1.67	0.51
CASK	4|6	0.05	2	0.51
SSBP1	3|2	0.05	1.67	0.51
MLLT11	5|4	0.05	2	0.51
GTF3C5	2|4	0.05	1.71	0.51
PJA2	4|0	0.05	1.14	0.51
SRGAP2	3|2	0.05	1.67	0.51
THUMPD1	4|6	0.05	2	0.51
VMP1	3|2	0.05	1.67	0.51
MTM1	2|2	0.04	1.6	0.52
RPL29	0|2	0.04	1	0.52
IBA57	2|2	0.04	1.6	0.52
NCBP2	2|0	0.04	1	0.52
CREBBP	0|2	0.04	1	0.52
PHACTR2	0|2	0.04	1	0.52
PPHLN1	2|0	0.04	1	0.52
AGO2	2|0	0.04	1	0.52
PEX19	2|2	0.04	1.6	0.52
GCLC	0|2	0.04	1	0.52
BID	2|0	0.04	1	0.52
FAM49B	0|2	0.04	1	0.52
RNH1	0|2	0.04	1	0.52

SNX4	0|2	0.04	1	0.52
BYSL	2|0	0.04	1	0.52
ACTL6A	4|4	0.04	2	0.52
ARIH1	0|2	0.04	1	0.52
RNASEH2A	2|2	0.04	1.6	0.52
DSTN	4|4	0.04	2	0.52
CPSF1	4|4	0.04	2	0.52
LSM3	2|0	0.04	1	0.52
UBXN4	4|4	0.04	2	0.52
RAB21	2|0	0.04	1	0.52
USP22	2|2	0.04	1.6	0.52
NOP16	0|2	0.04	1	0.52
SMG8	2|0	0.04	1	0.52
PANK4	5|4	0.04	2	0.52
DLG3	0|2	0.04	1	0.52
TRMT1L	0|2	0.04	1	0.52
RNASEH2C	0|2	0.04	1	0.52
FAM126A	2|2	0.04	1.6	0.52
CDK16	2|0	0.04	1	0.52
ZFP91	2|2	0.04	1.6	0.52
FAM207A	0|2	0.04	1	0.52
PTPRF	2|2	0.04	1.6	0.52
SAAL1	2|2	0.04	1.6	0.52
NHLRC2	4|4	0.04	2	0.52
PRC1	0|2	0.04	1	0.52
EIF4G2	5|5	0.04	2	0.54
ATP6V1C1	2|3	0.04	1.67	0.54
MAP1A	3|0	0.04	1	0.54
SUPT4H1	3|2	0.04	1.67	0.54
NCOR1	2|0	0.04	0.8	0.54
TMED10	3|0	0.04	1	0.54
OIP5	3|3	0.04	1.71	0.54
ZFR	2|3	0.04	1.67	0.54
TACO1	3|5	0.04	1.78	0.54
3-Sep	3|2	0.04	1.67	0.54
SPC25	3|0	0.04	1	0.54
ZNF622	0|3	0.04	1	0.54
DIP2B	0|3	0.04	1	0.54
LEMD3	4|3	0.03	1.75	0.55
PITRM1	7|7	0.03	2.15	0.55
AK2	12|13	0.03	2.27	0.55
CTSD	3|4	0.03	1.75	0.55
SH3GL1	3|3	0.03	1.71	0.55
IRS2	4|3	0.03	1.75	0.55
PIN1	2|5	0.03	1.56	0.55

PDIA5	6|2	0.03	1.45	0.55
FAF1	2|4	0.03	1.5	0.55
GMPPB	5|5	0.03	2	0.55
CMTR1	3|8	0.03	1.57	0.55
PINX1	3|3	0.03	1.71	0.55
ARL8B	5|4	0.03	1.8	0.55
PAPOLA	0|5	0.03	1.11	0.55
PML	3|4	0.03	1.75	0.55
CDK4	0|4	0.02	1	0.56
APRT	2|4	0.02	1.5	0.56
MARK3	2|0	0.02	0.8	0.56
BUB1B	3|2	0.02	1.43	0.56
NRDC	0|2	0.02	0.8	0.56
NAE1	0|4	0.02	1	0.56
ACP1	2|4	0.02	1.5	0.56
CMPK1	0|4	0.02	1	0.56
ARGLU1	2|0	0.02	0.8	0.56
NDC1	3|2	0.02	1.43	0.56
ASF1B	0|2	0.02	0.8	0.56
MARCKSL1	4|6	0.03	1.82	0.56
TMEM109	0|2	0.02	0.8	0.56
MRPS26	3|2	0.02	1.43	0.56
DCUN1D5	0|2	0.02	0.8	0.56
UQCC2	0|2	0.02	0.8	0.56
NDUFAF3	0|2	0.02	0.8	0.56
CLIC1	6|7	0.02	2	0.57
EIF4A2	11|13	0.02	2.29	0.57
GNAI2	3|0	0.02	0.86	0.57
PSME2	6|9	0.02	1.88	0.57
UBE2V2	4|4	0.02	1.78	0.57
AURKA	3|0	0.02	0.86	0.57
COX5A	5|4	0.02	1.8	0.57
RAB8A	5|4	0.02	1.8	0.57
NAP1L4	5|0	0.02	1	0.57
YEATS4	3|3	0.02	1.5	0.57
LSM8	4|5	0.02	1.8	0.57
OTUB1	4|4	0.02	1.78	0.57
UBB	2|2	0.02	1.33	0.57
UBE2E3	2|2	0.02	1.33	0.57
EIF4E	2|2	0.02	1.33	0.58
TRAFD1	3|0	0.01	0.86	0.58
MOB2	5|5	0.01	1.82	0.58
INTS7	2|2	0.02	1.33	0.58
CHMP2B	2|2	0.02	1.33	0.58
CLPB	5|6	0.01	1.83	0.58

PSMG1	0|3	0.01	0.86	0.58
GTF3C1	3|2	0.02	1.43	0.58
MAD2L1	5|5	0.01	1.82	0.58
RNMT	2|5	0.02	1.4	0.58
TBCA	8|7	0.01	2	0.58
ATP5F1C	5|5	0.01	1.82	0.58
ZC3H4	4|4	0.01	1.6	0.58
NPLOC4	2|5	0.02	1.4	0.58
RPRD1A	0|3	0.01	0.86	0.58
MOV10	2|2	0.02	1.33	0.58
CHID1	7|7	0.02	2	0.58
SLC3A2	6|7	0.01	1.86	0.59
YARS2	8|9	0.01	2	0.59
BCS1L	0|6	0.01	1	0.59
THADA	0|2	0.01	0.67	0.59
MICAL3	2|0	0.01	0.67	0.59
ETFA	11|13	0.01	2.09	0.59
VDAC2	0|4	0.01	0.89	0.59
GSTO1	8|11	0.01	1.9	0.59
PEBP1	7|9	0.01	1.88	0.59
RBM4	3|6	0.01	1.5	0.59
RPL15	4|6	0.01	1.67	0.59
SRPRA	9|11	0.01	2	0.59
WASL	3|3	0.01	1.5	0.59
BCR	4|2	0.01	1.33	0.59
DFFA	3|3	0.01	1.5	0.59
DDX20	4|3	0.01	1.56	0.59
FHOD1	2|0	0.01	0.67	0.59
NDUFAF4	7|6	0.01	1.86	0.59
ZSWIM8	2|0	0.01	0.67	0.59
SYNE2	5|3	0.01	1.45	0.59
ZCCHC8	0|2	0.01	0.67	0.59
TM9SF3	2|0	0.01	0.67	0.59
LSM2	0|2	0.01	0.67	0.59
CDC73	2|0	0.01	0.67	0.59
ATRX	2|0	0.01	0.67	0.59
RFC2	3|4	0.01	1.56	0.59
GIPC1	0|2	0.01	0.67	0.59
RAB12	2|0	0.01	0.67	0.6
ACOT9	0|2	0.01	0.67	0.6
RTL8C	2|2	0.01	1.14	0.6
HBS1L	5|4	0.01	1.64	0.6
QPCTL	5|4	0.01	1.64	0.6
RAB9A	3|5	0.01	1.45	0.6
MYL6B	4|5	0.01	1.64	0.6

MEAF6	2|0	0.01	0.67	0.6
IGBP1	4|7	0.01	1.57	0.6
SORD	4|7	0.01	1.57	0.6
SRM	4|4	0.01	1.6	0.6
STIM1	4|5	0.01	1.64	0.6
EIPR1	2|2	0.01	1.14	0.6
ERP29	3|5	0.01	1.45	0.6
PDCD10	0|2	0.01	0.67	0.6
CDC42EP4	2|0	0.01	0.67	0.6
MRTFB	0|2	0.01	0.67	0.6
DYNLRB1	0|2	0.01	0.67	0.6
ANKS1A	2|4	0.01	1.33	0.6
NMT1	5|4	0.01	1.64	0.6
SRPRB	5|4	0.01	1.64	0.6
BLOC1S2	2|2	0.01	1.14	0.6
MZT2A	2|2	0.01	1.14	0.61
CKAP2	3|5	0.01	1.45	0.61
ACADM	5|8	0.01	1.62	0.61
CDC5L	5|8	0.01	1.62	0.61
ARRB2	2|2	0.01	1.14	0.61
INPPL1	4|2	0.01	1.2	0.61
STAT3	5|10	0.01	1.58	0.61
FUBP3	0|4	0.01	0.8	0.61
NDUFS3	6|5	0.01	1.69	0.61
POLR2H	2|3	0.01	1.25	0.61
MTHFD2	3|7	0.01	1.43	0.61
TARDBP	7|10	0.01	1.79	0.61
EIF3K	2|2	0.01	1.14	0.61
NELFB	11|10	0.01	2	0.61
BUD23	2|2	0.01	1.14	0.61
CDKN2AIP	2|3	0.01	1.25	0.61
DAZAP1	2|2	0.01	1.14	0.61
AP2S1	2|2	0.01	1.14	0.61
UPF3B	5|6	0.01	1.69	0.61
SPATS2	2|2	0.01	1.14	0.61
PHAX	3|2	0.01	1.25	0.61
LRWD1	3|0	0.01	0.75	0.61
ALDH5A1	8|7	0.01	1.88	0.61
RFC3	3|2	0.01	1.25	0.61
PUS1	3|4	0	1.4	0.62
TLK2	3|3	0	1.33	0.62
SH3GLB1	3|3	0	1.33	0.62
RAE1	4|6	0	1.54	0.62
MAPKAPK5	6|7	0	1.73	0.62
TRIM25	2|4	0	1.2	0.62

ACAT2	5|8	0.01	1.62	0.62
SLC25A1	3|4	0	1.4	0.62
FBXO21	7|3	0	1.33	0.62
RPRD1B	0|4	0	0.8	0.62
ESS2	3|3	0	1.33	0.62
PHACTR4	0|2	0.01	0.57	0.62
HNRNPDL	3|3	0	1.33	0.62
LARP4	0|2	0	0.57	0.62
SDE2	0|2	0	0.57	0.62
ZNF598	2|0	0	0.57	0.62
ZNF326	4|3	0	1.4	0.62
FGFR1OP	2|0	0	0.57	0.62
RPL7A	3|4	0	1.27	0.63
FANCI	38|41	0	2.59	0.63
AMOT	2|3	0	1.11	0.63
DCAF11	2|4	0	1.2	0.63
ARF4	9|10	0	1.9	0.63
PRKCI	3|3	0	1.2	0.63
PRPF4B	3|0	0	0.67	0.63
GLO1	10|12	0	1.91	0.63
TALDO1	4|7	0	1.47	0.63
RSU1	17|14	0	2	0.63
SNAPIN	0|2	0	0.57	0.63
TIMM13	4|4	0	1.45	0.63
UCK2	5|3	0	1.33	0.63
SPEN	0|2	0	0.57	0.63
AAAS	4|2	0	1.2	0.63
ATP6V1D	3|0	0	0.67	0.63
RAB14	4|4	0	1.45	0.63
HACD3	7|5	0	1.6	0.63
CDV3	5|10	0	1.5	0.63
IARS2	9|9	0	1.89	0.63
CEP55	2|0	0	0.57	0.63
DNAJC10	9|7	0	1.78	0.63
YTHDC2	3|0	0	0.67	0.63
PRRC1	4|4	0	1.45	0.63
WIPF2	0|2	0	0.57	0.63
FECH	5|6	0	1.57	0.64
RPL6	4|8	0	1.41	0.64
RPS29	3|2	0	1.11	0.64
RPP38	0|3	0	0.67	0.64
HMGCS1	4|9	0	1.37	0.64
PARK7	11|17	0	1.75	0.64
NSDHL	3|3	0	1.2	0.64
MCTS1	0|4	0	0.73	0.64

NDE1	3|2	0	1.11	0.64
ENY2	3|3	0	1.2	0.64
CORO7-PAM16	3|6	0	1.29	0.64
PRKAR1A	2|3	0	1.11	0.64
ETFB	2|3	0	1.11	0.64
GNB1	2|3	0	1.11	0.64
PPP1R7	3|4	0	1.27	0.64
PYGB	3|0	0	0.6	0.64
MRPL12	4|3	0	1.27	0.64
ATXN2	3|3	0	1.2	0.64
TPT1	3|4	0	1.27	0.64
UGDH	0|4	0	0.73	0.64
SDHA	7|6	0	1.62	0.64
CIAO1	2|4	0	1.09	0.64
NME4	6|6	0	1.6	0.64
ORC2	2|3	0	1.11	0.64
PTGES3	2|3	0	1.11	0.64
MYBBP1A	10|11	0	1.91	0.64
ZC3H13	3|0	0	0.67	0.64
NUDCD1	0|3	0	0.67	0.64
MAP4K4	4|4	0	1.33	0.64
SNX1	3|3	0	1.2	0.64
TRMT61A	0|4	0	0.73	0.64
NADK2	3|3	0	1.2	0.64
PAFAH1B1	5|3	0	1.23	0.65
ALDH2	14|17	0	1.94	0.65
CHCHD4	3|5	0	1.23	0.65
PYGL	5|5	0	1.43	0.65
ALDH7A1	9|14	0	1.7	0.65
UBE2L3	7|8	0	1.67	0.65
PAK2	5|6	0	1.47	0.65
PFDN5	6|9	0	1.58	0.65
PNN	5|3	0	1.23	0.65
RCN1	22|21	0	2.15	0.65
HAT1	2|7	0	1.12	0.65
GATD3A	3|5	0	1.23	0.65
RBM6	3|2	0	1	0.65
TBCD	6|10	0	1.52	0.65
IGF2BP3	5|3	0	1.23	0.65
PACSIN2	3|2	0	1	0.65
MON2	17|13	0	1.88	0.65
NOP58	12|10	0	1.83	0.65
CDK12	7|4	0	1.38	0.65
UNC45A	5|4	0	1.38	0.65
TUBAL3	6|5	0	1.47	0.65

POLDIP3	7|7	0	1.65	0.65
DIDO1	7|5	0	1.5	0.65
UBE2I	3|5	0	1.23	0.65
BABAM2	4|4	0	1.33	0.65
RCC1	5|6	0	1.47	0.66
RPN2	6|4	0	1.33	0.66
MDH1	7|6	0	1.53	0.66
BZW1	4|3	0	1.17	0.66
ABCB7	3|3	0	1.09	0.66
ESD	10|14	0	1.71	0.66
GLUL	6|4	0	1.33	0.66
AIP	5|5	0	1.43	0.66
SCO2	3|3	0	1.09	0.66
LGALS3BP	2|3	0	1	0.66
IDH1	6|6	0	1.5	0.66
DYNC1LI2	3|3	0	1.09	0.66
DST	0|4	0	0.67	0.66
TRMT112	2|3	0	1	0.66
RAC1	2|3	0	1	0.66
PDLIM1	7|11	0	1.57	0.66
FKBP10	9|8	0	1.7	0.66
THOC3	2|3	0	1	0.66
BAX	2|3	0	1	0.66
NDUFAF2	3|0	0	0.6	0.66
VIRMA	8|5	0	1.44	0.66
LIG1	0|5	0	0.71	0.67
ALDH3A2	15|11	0	1.73	0.67
RPL12	4|4	0	1.23	0.67
ARHGAP17	5|9	0	1.4	0.67
NT5C2	3|5	0	1.14	0.67
ANXA2	3|8	0	1.16	0.67
PFKFB3	8|6	0	1.47	0.67
ISYNA1	9|12	0	1.68	0.67
CBS	5|6	0	1.38	0.67
NOP2	3|4	0	1.17	0.67
DLAT	2|5	0	1	0.67
PGM1	3|5	0	1.14	0.67
RPA2	2|4	0	1	0.67
RPA3	4|4	0	1.23	0.67
HMGA2	5|4	0	1.29	0.67
VAPA	3|5	0	1.14	0.67
DLG1	5|6	0	1.38	0.67
IDH3G	2|3	0	0.91	0.67
VPS26A	2|7	0	1.06	0.67
TPD52	4|5	0	1.29	0.67

CFL1	8|10	0	1.64	0.67
SEC23A	5|6	0	1.38	0.67
FKBP8	4|3	0	1.17	0.67
PSME4	7|3	0	1.18	0.67
KIF1BP	0|5	0	0.71	0.67
DARS2	11|11	0	1.83	0.67
CCDC93	0|4	0	0.67	0.67
XPNPEP1	2|4	0	1	0.67
DCTPP1	2|5	0	1	0.67
EDC3	4|4	0	1.23	0.67
RPL8	3|5	0	1.14	0.67
DNAJB6	6|5	0	1.38	0.67
MOSPD2	2|3	0	0.91	0.67
RB1	4|3	0	1.08	0.68
STK26	4|8	0	1.26	0.68
XRN1	3|4	0	1.08	0.68
ADD1	8|8	0	1.6	0.68
CISD3	4|0	0	0.62	0.68
SRSF7	4|4	0	1.14	0.68
ARPC4	5|4	0	1.2	0.68
CARS	5|8	0	1.37	0.68
COX4I1	4|3	0	1.08	0.68
RING1	2|3	0	0.91	0.68
TRIP12	2|3	0	0.91	0.68
ACSL3	9|6	0	1.43	0.68
ARPC1B	3|4	0	1.08	0.68
NOP56	9|10	0	1.65	0.68
AFG3L2	4|4	0	1.14	0.68
SF3A2	3|2	0	0.91	0.68
RPL35	4|3	0	1.08	0.68
BRD3	0|4	0	0.62	0.68
NUP62	7|8	0	1.5	0.68
BABAM1	2|4	0	0.92	0.68
WAPL	3|2	0	0.91	0.68
FKBP15	3|4	0	1.08	0.68
GLOD4	4|5	0	1.2	0.68
SCFD1	3|6	0	1.12	0.68
ATF7IP	2|3	0	0.91	0.68
COLGALT1	9|4	0	1.24	0.68
MRPL14	4|3	0	1.08	0.68
NTPCR	6|4	0	1.25	0.68
RRBP1	5|8	0	1.3	0.69
PLOD3	7|5	0	1.26	0.69
APP	5|3	0	1.07	0.69
TBC1D15	5|5	0	1.25	0.69

RAP1B	3|4	0	1	0.69
APEX1	10|12	0	1.63	0.69
RPN1	22|21	0	2	0.69
SAFB	6|7	0	1.37	0.69
LLGL1	4|3	0	1	0.69
TRIP13	9|8	0	1.55	0.69
SNRPA	4|4	0	1.07	0.69
USP11	9|10	0	1.58	0.69
MLF2	5|3	0	1.07	0.69
GNPDA1	5|6	0	1.29	0.69
H1FX	3|5	0	1.07	0.69
TIMM44	15|12	0	1.74	0.69
PNMA2	3|4	0	1	0.69
CBX3	3|4	0	1	0.69
VPS51	5|7	0	1.26	0.69
MAT2B	5|8	0	1.3	0.69
CADM1	7|6	0	1.37	0.69
NUDCD3	4|6	0	1.18	0.69
LAP3	6|9	0	1.36	0.69
NDUFV3	3|4	0	1	0.69
EIF4H	3|4	0	1	0.69
CTNNBL1	0|4	0	0.57	0.69
TUBA1C	97|94	0	3.08	0.69
SEC23B	3|5	0	1.07	0.69
OPA1	16|14	0	1.82	0.69
CCDC50	3|4	0	1	0.69
FH	11|13	0	1.66	0.7
RPL27	3|5	0	1	0.7
NACA	2|6	0	0.89	0.7
CYB5R3	5|6	0	1.22	0.7
CHAMP1	8|7	0	1.43	0.7
GLRX3	8|8	0	1.45	0.7
RPL18	7|7	0	1.4	0.7
AP3S1	6|3	0	1	0.7
ELAVL1	6|7	0	1.3	0.7
ATP2B1	5|3	0	1	0.7
PRKACA	4|6	0	1.11	0.7
PSMD10	0|7	0	0.74	0.7
SBF1	6|6	0	1.26	0.7
SGTA	4|5	0	1.12	0.7
UQCRC2	5|5	0	1.18	0.7
MBD3	4|5	0	1.12	0.7
SCAMP3	5|4	0	1.12	0.7
RACK1	7|11	0	1.38	0.7
NPEPPS	6|14	0	1.33	0.7

POLD3	5|3	0	1	0.7
OGFR	6|8	0	1.33	0.7
TTLL12	6|9	0	1.3	0.7
RAB10	6|7	0	1.3	0.7
NUP54	6|6	0	1.26	0.7
MAP1S	7|4	0	1.16	0.7
LIN7C	3|6	0	1	0.7
COPRS	4|4	0	1.07	0.7
CMAS	7|9	0	1.39	0.7
CEP41	6|6	0	1.26	0.7
CYCS	7|10	0	1.42	0.7
NUP107	16|16	0	1.83	0.7
PSAT1	7|8	0	1.43	0.7
ACSL4	8|7	0	1.43	0.7
LEO1	7|9	0	1.39	0.7
CARM1	5|5	0	1.18	0.7
ALDH9A1	6|6	0	1.2	0.71
DIAPH3	5|4	0	1.06	0.71
EPN1	3|4	0	0.93	0.71
NDRG1	6|7	0	1.24	0.71
SCAF4	4|3	0	0.93	0.71
RRM2	3|4	0	0.93	0.71
CIRBP	8|7	0	1.36	0.71
FAU	4|5	0	1.06	0.71
NUP88	5|5	0	1.11	0.71
SRP14	5|3	0	0.94	0.71
STAM	4|7	0	1.1	0.71
RANBP3	5|6	0	1.16	0.71
EIF3J	5|5	0	1.11	0.71
UCHL1	7|9	0	1.33	0.71
ASNA1	3|7	0	1	0.71
HOMER2	5|0	0	0.59	0.71
PPIF	2|4	0	0.8	0.71
AASS	4|7	0	1.1	0.71
CLPP	6|7	0	1.24	0.71
PKN2	2|6	0	0.84	0.71
RAD21	10|13	0	1.53	0.71
CHORDC1	6|11	0	1.26	0.71
HNRNPH3	7|9	0	1.33	0.71
RABGAP1L	3|6	0	0.95	0.71
LRRC40	2|5	0	0.82	0.71
CIAPIN1	3|4	0	0.93	0.71
USP28	7|7	0	1.33	0.71
TRAF2	3|4	0	0.93	0.71
HNRNPAB	8|9	0	1.42	0.71

CPVL	7|8	0	1.36	0.71
CDK2	7|9	0	1.33	0.71
GADD45GIP1	4|2	0	0.8	0.71
TMEM263	7|7	0	1.33	0.71
PLCG1	3|6	0	0.95	0.71
TIMM50	13|12	0	1.61	0.72
TBCE	3|7	0	0.95	0.72
CTNND1	5|2	0	0.78	0.72
DBNL	2|5	0	0.78	0.72
LYAR	7|7	0	1.22	0.72
CUL7	5|3	0	0.84	0.72
TBCB	5|9	0	1.12	0.72
CBR1	6|8	0	1.22	0.72
LBR	6|10	0	1.23	0.72
SLC25A3	7|4	0	1.05	0.72
POLR2E	3|5	0	0.89	0.72
PTPN11	4|8	0	1.04	0.72
RANBP1	7|9	0	1.28	0.72
SNX2	7|8	0	1.3	0.72
EIF3F	10|9	0	1.41	0.72
ARHGEF1	4|5	0	0.95	0.72
ACTR3	4|6	0	1	0.72
NUCB1	10|10	0	1.48	0.72
MOGS	9|10	0	1.46	0.72
G3BP2	3|5	0	0.84	0.72
MAPRE1	11|13	0	1.5	0.72
UBQLN2	5|7	0	1.14	0.72
VWA8	5|7	0	1.14	0.72
SARM1	4|5	0	0.95	0.72
IRF2BP1	5|4	0	0.95	0.72
CC2D1A	5|7	0	1.14	0.72
TSR1	5|8	0	1.08	0.72
MATR3	15|24	0	1.59	0.72
PPA1	15|22	0	1.64	0.72
SEC24A	2|5	0	0.78	0.72
ACBD3	3|7	0	0.91	0.72
MCMBP	0|5	0	0.56	0.72
WDR82	4|5	0	0.95	0.72
LSM14B	5|4	0	1	0.72
ARPC2	7|10	0	1.26	0.72
ACOT7	8|10	0	1.33	0.72
CHCHD1	3|5	0	0.89	0.72
ENSA	6|8	0	1.22	0.72
ACAT1	17|16	0	1.65	0.73
RPS23	6|5	0	1	0.73

8-Sep	6|6	0	1.04	0.73
ADSS	4|6	0	0.95	0.73
STMN1	7|6	0	1.08	0.73
CLINT1	5|7	0	1.04	0.73
MAP7	7|8	0	1.2	0.73
ANK3	5|7	0	1	0.73
PPP1CC	6|5	0	1	0.73
RCN2	20|19	0	1.81	0.73
DYNC1I2	8|9	0	1.26	0.73
DUT	6|7	0	1.08	0.73
HNRNPD	5|8	0	1.04	0.73
RBMX	8|8	0	1.23	0.73
MARCKS	16|23	0	1.62	0.73
PGD	6|14	0	1.14	0.73
TRIP6	8|10	0	1.29	0.73
VBP1	5|6	0	1	0.73
EIF3H	9|7	0	1.23	0.73
HDGF	8|12	0	1.29	0.73
PCMT1	8|9	0	1.31	0.73
NELFA	5|9	0	1.08	0.73
PSMD14	6|5	0	1.05	0.73
CAPRIN1	5|6	0	1	0.73
MANF	5|6	0	1.05	0.73
ANP32A	7|6	0	1.13	0.73
ARPC1A	5|9	0	1.04	0.73
TCEA1	8|9	0	1.31	0.73
PYCR1	8|10	0	1.24	0.73
CDC37	8|9	0	1.26	0.73
PFDN2	11|11	0	1.47	0.73
ZNF638	17|11	0	1.4	0.73
LTN1	12|10	0	1.38	0.73
VPS29	5|6	0	1	0.73
ZC3H15	8|6	0	1.12	0.73
DPYSL5	9|14	0	1.31	0.73
LRRC47	4|6	0	0.91	0.73
CHTF18	0|6	0	0.55	0.73
RBM26	9|8	0	1.31	0.73
PHF6	7|5	0	1.04	0.73
PNISR	4|7	0	0.96	0.73
FANCD2	24|24	0	1.85	0.73
NEK9	4|6	0	0.91	0.73
SKP1	9|9	0	1.33	0.73
LMNA	21|25	0	1.77	0.73
PPP4R2	6|9	0	1.11	0.73
NUP43	6|7	0	1.13	0.73

PRIM2	7|7	0	1.12	0.74
DNM1	12|11	0	1.39	0.74
PSPC1	13|14	0	1.5	0.74
PLS3	11|16	0	1.35	0.74
API5	8|8	0	1.14	0.74
ATXN10	5|8	0	0.96	0.74
PDHA1	6|11	0	1.1	0.74
RAB5C	5|7	0	0.96	0.74
ECH1	10|13	0	1.28	0.74
GDI2	20|26	0	1.67	0.74
BSG	7|7	0	1.08	0.74
CNN3	10|13	0	1.28	0.74
USP5	19|20	0	1.66	0.74
DHX16	4|6	0	0.87	0.74
CUL2	13|11	0	1.41	0.74
RAB7A	8|9	0	1.17	0.74
SEC24C	9|13	0	1.26	0.74
NUCB2	10|10	0	1.29	0.74
IDH3A	6|5	0	0.96	0.74
ALYREF	11|13	0	1.33	0.74
SRRM1	13|12	0	1.43	0.74
TUBB4A	64|61	0	2.55	0.74
ACAA2	10|11	0	1.35	0.74
PSME1	8|9	0	1.21	0.74
BASP1	9|11	0	1.29	0.74
ANP32B	9|9	0	1.24	0.74
SARS	9|13	0	1.29	0.74
PGRMC1	7|7	0	1.08	0.74
SNRNP27	9|5	0	0.97	0.74
SUPT16H	7|9	0	1.1	0.74
OLA1	6|10	0	1.1	0.74
MDC1	8|9	0	1.17	0.74
MESD	7|7	0	1.08	0.74
TRMT10C	7|6	0	1.04	0.74
SMU1	12|10	0	1.29	0.74
RELCH	7|6	0	1.04	0.74
GRPEL1	6|8	0	1	0.74
LAS1L	6|5	0	0.96	0.74
PTBP1	14|18	0	1.52	0.74
PNPT1	8|9	0	1.21	0.74
RTN4	4|10	0	0.9	0.74
TCF20	7|4	0	0.85	0.74
ADSL	9|12	0	1.2	0.75
PNP	10|14	0	1.23	0.75
RPL5	2|8	0	0.69	0.75

RPLP2	6|9	0	0.94	0.75
SNCA	8|5	0	0.93	0.75
CUL3	5|10	0	0.91	0.75
CSTF2	10|11	0	1.24	0.75
ATP5PO	9|9	0	1.12	0.75
CTNNA1	9|8	0	1.1	0.75
EIF5	5|7	0	0.89	0.75
KPNA3	6|7	0	0.96	0.75
PPAT	8|12	0	1.14	0.75
MAPK6	9|6	0	0.97	0.75
VDAC1	6|11	0	1	0.75
ZYX	6|9	0	0.97	0.75
BUD31	7|6	0	0.96	0.75
ECHS1	11|11	0	1.22	0.75
DRG1	7|5	0	0.89	0.75
SNAP29	8|9	0	1.13	0.75
HSPA5	130|119	0	2.24	0.75
RPS14	7|10	0	1	0.75
PA2G4	11|11	0	1.22	0.75
SUGT1	8|13	0	1.17	0.75
WDHD1	9|7	0	1	0.75
UTRN	10|7	0	1	0.75
HSPBP1	5|8	0	0.93	0.75
SRP68	8|11	0	1.06	0.75
MAGED2	14|12	0	1.3	0.75
MDN1	10|12	0	1.16	0.75
NUP205	18|20	0	1.58	0.75
HSD17B12	9|8	0	1.13	0.75
NSFL1C	9|11	0	1.11	0.75
WBP11	8|9	0	1.1	0.75
KDM3B	5|8	0	0.87	0.75
SARS2	7|11	0	1.09	0.75
IPO9	9|10	0	1.12	0.75
GPALPP1	11|8	0	1.09	0.75
PPIA	15|17	0	1.39	0.75
MCCC2	10|8	0	1.09	0.75
BRCC3	9|7	0	1.07	0.75
RAB1B	9|10	0	1.12	0.75
PCBP2	12|15	0	1.29	0.75
AP1M1	9|11	0	1.18	0.75
SYAP1	5|9	0	0.9	0.75
JPT2	11|12	0	1.24	0.75
IDH3B	5|8	0	0.93	0.75
IRF2BP2	9|10	0	1.12	0.75
E2F7	17|15	0	1.39	0.75

GPS1	14|14	0	1.44	0.75
GPI	10|10	0	1.08	0.76
RPL23A	11|8	0	1	0.76
CTNNB1	10|8	0	1.03	0.76
TKT	5|11	0	0.86	0.76
TNPO2	10|8	0	0.95	0.76
SLC25A5	13|13	0	1.24	0.76
PPP2R2A	11|15	0	1.13	0.76
CALU	35|35	0	1.84	0.76
AFDN	10|14	0	1.14	0.76
HARS	7|10	0	0.89	0.76
ACADVL	10|10	0	1.11	0.76
CSRP2	9|9	0	1.03	0.76
EIF3E	9|9	0	1.06	0.76
SLC25A6	13|14	0	1.23	0.76
EEF1A2	0|37	0	0.91	0.76
KRT19	13|14	0	1.29	0.76
PGAM1	20|25	0	1.48	0.76
PSMC6	9|11	0	1.03	0.76
TMPO	20|25	0	1.43	0.76
VCL	11|15	0	1.18	0.76
YWHAH	8|11	0	1.03	0.76
CS	18|19	0	1.42	0.76
GTF2F2	10|9	0	1.06	0.76
ETF1	6|12	0	0.88	0.76
PFN1	13|17	0	1.25	0.76
PDIA3	34|39	0	1.78	0.76
UBA2	14|19	0	1.29	0.76
MRE11	15|12	0	1.17	0.76
ACTR1A	14|13	0	1.2	0.76
FARP1	9|7	0	0.94	0.76
DNAJA2	20|17	0	1.35	0.76
TUBA1A	86|89	0	2.15	0.76
NUMA1	10|11	0	1.11	0.76
PCBP1	10|12	0	1.07	0.76
RAN	11|14	0	1.14	0.76
TCERG1	11|15	0	1.21	0.76
COPS5	12|14	0	1.18	0.76
LETM1	7|17	0	1.02	0.76
PYCR2	12|13	0	1.14	0.76
PDCD6IP	8|11	0	1	0.76
PRPF19	13|15	0	1.24	0.76
NUP93	13|12	0	1.22	0.76
NUP98	10|9	0	0.97	0.76
MOB1A	10|9	0	1.09	0.76

WDR33	23|13	0	1.24	0.76
MEPCE	10|11	0	1.14	0.76
IPO4	12|12	0	1.2	0.76
SLIRP	9|10	0	1	0.76
TUBB6	47|43	0	1.84	0.76
CDK11B	10|9	0	0.97	0.76
DDX39B	14|17	0	1.24	0.76
AIFM1	14|10	0	1.07	0.76
PLEC	12|0	0	0.6	0.76
HADHA	15|15	0	1.13	0.77
MSH2	15|15	0	1.22	0.77
OAT	23|20	0	1.3	0.77
PGK1	20|27	0	1.34	0.77
CUL4A	11|12	0	1.02	0.77
HNRNPH2	10|11	0	1	0.77
SREK1	11|10	0	0.98	0.77
PDS5A	12|14	0	1.13	0.77
TCOF1	16|18	0	1.28	0.77
TP53BP1	12|13	0	1.06	0.77
EIF5A	11|13	0	1.09	0.77
EPB41	8|12	0	0.89	0.77
CCNT1	19|19	0	1.38	0.77
C7orf55-LUC7L2	27|24	0	1.52	0.77
FBL	12|12	0	1.02	0.77
DNAJA1	28|30	0	1.59	0.77
AARS	20|22	0	1.33	0.77
CTPS1	12|11	0	1.07	0.77
PRKCSH	23|22	0	1.48	0.77
PSMD7	15|15	0	1.11	0.77
PSMD13	13|12	0	1.04	0.77
RPS2	12|11	0	1	0.77
TAGLN2	9|13	0	0.98	0.77
ATIC	21|29	0	1.33	0.77
ILF2	12|13	0	1.11	0.77
NARS	12|13	0	1.09	0.77
DDX21	14|18	0	1.16	0.77
NOLC1	18|18	0	1.31	0.77
PRDX6	11|16	0	1	0.77
MARS	16|17	0	1.2	0.77
CTTN	16|22	0	1.23	0.77
SAE1	10|15	0	1.02	0.77
RAD50	17|19	0	1.26	0.77
PDIA6	22|24	0	1.39	0.77
MAT2A	12|13	0	1.06	0.77
MDH2	14|16	0	1.2	0.77

HNRNPM	18|23	0	1.34	0.77
ARID1A	12|17	0	1.14	0.77
RBM14	19|16	0	1.23	0.77
TACC3	10|12	0	0.94	0.77
VAT1	12|15	0	1.06	0.77
KHDRBS1	13|14	0	1.12	0.77
PHGDH	14|14	0	1.14	0.77
UGP2	8|12	0	0.85	0.77
CPSF6	11|12	0	1	0.77
DNM1L	17|25	0	1.27	0.77
XRN2	13|17	0	1.13	0.77
PFAS	15|20	0	1.19	0.77
HSPA4L	11|16	0	1.1	0.77
CACYBP	13|18	0	1.11	0.77
PDXDC1	13|14	0	1.06	0.77
PRPF38B	13|10	0	0.92	0.77
VPS35	8|12	0	0.93	0.77
LRRC59	10|11	0	0.98	0.77
SLAIN2	11|13	0	1	0.77
HIST1H4J	13|10	0	0.92	0.77
LMNB2	19|17	0	1.22	0.77
NUP155	22|28	0	1.39	0.77
PCNA	12|14	0	1.08	0.77
SCRIB	21|18	0	1.34	0.77
NME1	14|15	0	1.09	0.77
HSD17B4	17|20	0	1.06	0.78
ATP1A1	29|20	0	1.15	0.78
P4HB	32|37	0	1.47	0.78
RPL23	16|14	0	1	0.78
SLTM	18|19	0	1.14	0.78
NME1-NME2	20|23	0	1.25	0.78
CAST	10|14	0	0.92	0.78
HNRNPF	18|21	0	1.11	0.78
DDX17	14|17	0	0.94	0.78
YWHAZ	16|21	0	1.09	0.78
FKBP5	18|17	0	1.01	0.78
LDHA	11|15	0	0.96	0.78
PFKM	13|14	0	1.02	0.78
LDHB	18|20	0	1.17	0.78
SRSF11	17|17	0	1.08	0.78
RBBP7	15|18	0	1	0.78
RDX	18|21	0	1.16	0.78
CPD	22|20	0	1.09	0.78
7-Sep	18|13	0	0.94	0.78
GTF2F1	12|14	0	1	0.78

MSN	18|26	0	1.22	0.78
PSMD12	14|12	0	0.95	0.78
RPA1	18|19	0	1.14	0.78
SSB	27|33	0	1.38	0.78
SSRP1	14|15	0	1.05	0.78
ELOA	14|17	0	1.05	0.78
YARS	18|19	0	1.16	0.78
ATP5F1A	21|22	0	1.26	0.78
CALR	30|31	0	1.47	0.78
DDX5	15|18	0	1.06	0.78
DDX6	13|11	0	0.94	0.78
2-Sep	14|15	0	0.98	0.78
HSD17B10	17|19	0	1.2	0.78
YBX1	15|18	0	0.93	0.78
MED1	15|13	0	1.02	0.78
LONP1	22|26	0	1.2	0.78
SYMPK	15|15	0	1.02	0.78
PDIA4	38|43	0	1.53	0.78
NUP153	18|19	0	1.06	0.78
GLUD1	18|18	0	1.06	0.78
HCFC1	16|19	0	1.03	0.78
NCL	22|22	0	1.16	0.78
SHMT2	26|32	0	1.25	0.78
NUDC	20|30	0	1.23	0.78
SPIN1	17|19	0	1.11	0.78
YWHAQ	13|17	0	0.97	0.78
TBC1D10B	20|15	0	1.01	0.78
LUC7L3	27|24	0	1.24	0.78
RBM27	19|20	0	1.26	0.78
DIABLO	14|16	0	0.98	0.78
XPO5	16|19	0	1.17	0.78
PPM1A	24|23	0	1.31	0.78
RBM33	18|17	0	0.99	0.78
ASNS	17|20	0	1.17	0.78
SPINDOC	17|15	0	1.05	0.78
NAP1L1	16|16	0	1.1	0.78
YWHAB	14|18	0	0.91	0.78
ATXN2L	17|20	0	1.19	0.78
TARS	12|21	0	0.89	0.78
TPM3	17|15	0	1.07	0.78
PPM1G	17|17	0	1.03	0.78
RBM39	19|17	0	1.03	0.78
HNRNPA3	13|15	0	0.98	0.78
SUPT5H	27|31	0	1.03	0.79
EZR	31|30	0	1.17	0.79

ALDOA	28|35	0	1.18	0.79
HYOU1	41|43	0	1.46	0.79
DNM2	27|26	0	1.18	0.79
NSUN2	14|19	0	0.89	0.79
PRDX1	19|20	0	1.01	0.79
HNRNPH1	19|26	0	0.99	0.79
DHX9	30|24	0	0.97	0.79
EEF1A1	44|48	0	1.45	0.79
EIF4B	44|42	0	1.34	0.79
ENO1	51|60	0	1.36	0.79
EPB41L2	31|37	0	1.21	0.79
AHNAK	24|31	0	0.92	0.79
ARCN1	22|24	0	1.02	0.79
ATP5F1B	25|29	0	1.05	0.79
CKB	39|43	0	1.24	0.79
FKBP4	35|44	0	1.13	0.79
GSPT1	20|22	0	1.08	0.79
HNRNPA1	27|32	0	1.09	0.79
HSPA4	39|42	0	1.09	0.79
IPO5	26|23	0	1.13	0.79
PFKP	30|30	0	1.22	0.79
PSMD1	23|25	0	0.98	0.79
SUPT6H	30|29	0	1.17	0.79
TPP2	19|26	0	0.93	0.79
TUFM	27|33	0	1.18	0.79
UBA1	38|44	0	1.12	0.79
VIM	35|35	0	1.24	0.79
XPO1	32|28	0	1.11	0.79
USP7	35|34	0	1.24	0.79
IRS4	51|44	0	1.22	0.79
KHSRP	21|26	0	1.02	0.79
USO1	26|29	0	1.13	0.79
EIF3G	18|20	0	1	0.79
MTA1	24|24	0	1.12	0.79
DDX1	20|26	0	1.02	0.79
SFPQ	28|27	0	1.17	0.79
THRAP3	43|41	0	1.34	0.79
DIAPH1	27|24	0	0.97	0.79
ZRANB2	19|19	0	1.01	0.79
LMNB1	42|42	0	1.44	0.79
MTHFD1	37|40	0	1.22	0.79
TUBB4B	69|65	0	1.61	0.79
HSPH1	30|31	0	1.14	0.79
YWHAE	38|45	0	1.09	0.79
SEC23IP	21|17	0	0.96	0.79

U2AF2	27|21	0	0.92	0.79
ACAP2	25|19	0	0.94	0.79
EWSR1	19|25	0	0.91	0.79
PPP2R1A	19|22	0	0.94	0.79
UBAP2L	33|36	0	1	0.79
ESYT1	24|26	0	1.15	0.79
TRAP1	32|35	0	1.14	0.79
NUP133	26|22	0	0.96	0.79
CAND1	29|31	0	1.13	0.79
XRCC5	28|33	0	0.93	0.79
HNRNPA2B1	31|34	0	1.07	0.79
HNRNPK	29|35	0	1.04	0.79
PPM1B	27|26	0	1.01	0.79
TUBB2B	66|62	0	1.66	0.79
TUBB	85|81	0	1.65	0.79
MSH6	14|18	0	0.88	0.8
HADHB	11|10	0	0.93	0.8
HPRT1	0|3	0	0.46	0.8
KRT9	19|19	0	0.46	0.8
PLOD1	11|10	0	0.82	0.8
PTS	13|10	0	0.66	0.8
KRT2	18|33	0	0.73	0.8
PCCB	6|6	0	0.77	0.8
HPX	2|0	0	0.44	0.8
AHCY	15|15	0	0.86	0.8
BLVRA	2|2	0	1	0.8
IGF2R	0|2	0	0.15	0.8
IMPDH2	9|8	0	0.55	0.8
PDHB	6|6	0	0.77	0.8
POLR2A	14|12	0	0.63	0.8
POLR2B	13|12	0	0.7	0.8
PPIB	32|29	0	0.74	0.8
PRIM1	8|7	0	0.61	0.8
RPL4	3|6	0	0.72	0.8
RPL11	2|2	0	0.73	0.8
RPL19	2|2	0	0.73	0.8
RPL22	3|4	0	0.56	0.8
RPL24	0|2	0	0.5	0.8
RPL26	7|5	0	0.86	0.8
RPL38	14|13	0	0.51	0.8
RPS3	14|13	0	0.73	0.8
RPS3A	6|6	0	0.51	0.8
RPS4X	12|12	0	0.75	0.8
RPS5	4|4	0	0.64	0.8
RPS6	2|2	0	0.57	0.8

RPS7	11|11	0	0.81	0.8
SMC4	30|29	0	0.66	0.8
RAB11FIP1	4|5	0	0.86	0.8
GPATCH8	3|5	0	0.67	0.8
TWISTNB	4|2	0	0.41	0.8
RPS8	5|5	0	0.77	0.8
RPS9	7|7	0	0.67	0.8
WASHC2A	6|6	0	0.43	0.8
RPS11	4|4	0	0.67	0.8
TCEAL4	2|2	0	0.62	0.8
RPS13	4|4	0	0.55	0.8
ENAH	9|11	0	0.75	0.8
CKAP5	22|24	0	0.57	0.8
CADPS2	5|7	0	0.75	0.8
KDM1A	8|7	0	0.86	0.8
RPS15A	5|3	0	0.62	0.8
TTC7B	2|0	0	0.5	0.8
RPS16	9|7	0	0.7	0.8
RPSA	9|8	0	0.83	0.8
DNAJC21	0|2	0	0.5	0.8
RPS19	6|7	0	0.6	0.8
RPS21	3|3	0	0.67	0.8
HSP90AA1	92|94	0	0.79	0.8
SERBP1	38|39	0	0.84	0.8
RPS25	4|4	0	0.76	0.8
RPS26	4|3	0	0.67	0.8
ABCF1	13|13	0	0.93	0.8
RPS27	5|6	0	0.81	0.8
RPS28	2|2	0	0.89	0.8
RRM1	5|8	0	0.68	0.8
TP53BP2	2|2	0	1	0.8
PLAA	2|4	0	0.75	0.8
INF2	5|6	0	0.71	0.8
CYFIP2	9|6	0	0.77	0.8
SRSF5	2|0	0	0.44	0.8
USP9X	42|46	0	0.81	0.8
HIST2H2AA4	3|4	0	0.5	0.8
TROVE2	2|3	0	0.77	0.8
PPP4R1	3|2	0	0.56	0.8
MAZ	3|3	0	0.6	0.8
STAG2	9|8	0	0.87	0.8
CLCC1	6|5	0	0.92	0.8
TUBG1	0|2	0	0.19	0.8
SEC31A	18|17	0	0.74	0.8
URGCP	2|0	0	0.5	0.8

KTN1	4|4	0	0.89	0.8
PAICS	15|14	0	0.83	0.8
HNRNPUL2	0|2	0	0.44	0.8
ECPAS	28|23	0	0.91	0.8
U2SURP	15|19	0	0.71	0.8
CAMSAP3	24|25	0	0.91	0.8
DYNC2H1	4|2	0	0.52	0.8
MGA	14|16	0	0.91	0.8
THOC2	15|16	0	0.95	0.8
PRKDC	54|55	0	0.77	0.8
STRN3	3|3	0	0.46	0.8
WDR62	3|2	0	0.77	0.8
ACLY	56|56	0	0.83	0.8
GRSF1	2|2	0	0.73	0.8
RPL31	2|3	0	0.67	0.8
IFT74	0|2	0	0.4	0.8
OTUD4	3|0	0	0.29	0.8
PSMA4	3|2	0	0.3	0.8
GIGYF2	9|8	0	0.77	0.8
RPP30	2|3	0	0.56	0.8
CAP1	23|22	0	0.85	0.8
BRD2	5|3	0	0.42	0.8
ARHGEF7	0|2	0	0.25	0.8
WDR26	2|0	0	0.22	0.8
ADD3	3|3	0	0.86	0.8
PPP6C	4|3	0	0.82	0.8
AP1G1	0|6	0	0.39	0.8
CSK	2|4	0	0.67	0.8
SHTN1	2|6	0	0.52	0.8
POLD2	4|3	0	0.82	0.8
GPBP1	3|0	0	0.32	0.8
CBX5	2|2	0	0.73	0.8
PCCA	8|8	0	0.71	0.8
VPS53	0|2	0	0.18	0.8
SRRT	11|12	0	0.96	0.8
ACTN1	19|19	0	0.61	0.8
EEF1D	14|14	0	0.8	0.8
MYO9B	2|0	0	0.5	0.8
KARS	2|5	0	0.35	0.8
SMARCC2	5|4	0	0.9	0.8
CSDE1	7|6	0	0.38	0.8
SPAG9	35|37	0	0.86	0.8
DNMT1	5|9	0	0.68	0.8
PEX5	2|0	0	0.44	0.8
MAP4	10|12	0	0.61	0.8

LRRFIP2	2|2	0	0.67	0.8
IPO11	2|0	0	0.33	0.8
FIP1L1	9|7	0	0.86	0.8
DCTN1	13|11	0	0.59	0.8
TAF12	2|2	0	1	0.8
RBBP4	9|0	0	0.39	0.8
PIP5K1A	7|5	0	0.86	0.8
DCTN4	2|2	0	0.47	0.8
PABPC4	0|8	0	0.31	0.8
ECD	0|2	0	0.27	0.8
GART	40|47	0	0.91	0.8
TRMT1	5|5	0	0.69	0.8
CPSF7	6|7	0	0.93	0.8
RCC2	29|25	0	0.81	0.8
RCOR3	2|2	0	1	0.8
SEC13	3|3	0	0.67	0.8
BTF3L4	0|2	0	0.5	0.8
CLASP1	2|0	0	0.5	0.8
SPART	4|4	0	0.8	0.8
AIMP1	3|3	0	0.55	0.8
MORF4L2	6|6	0	1	0.8
DKC1	3|3	0	0.46	0.8
P4HA1	9|9	0	1	0.8
PPP1R12A	4|3	0	0.39	0.8
COPS2	13|13	0	0.87	0.8
C12orf65	2|0	0	0.4	0.8
COPB1	34|32	0	0.85	0.8
PRMT3	2|3	0	0.77	0.8
EXOC7	7|5	0	0.67	0.8
SIN3A	4|4	0	1	0.8
NONO	23|24	0	0.91	0.8
TTC28	3|3	0	0.36	0.8
PAFAH1B3	0|2	0	0.44	0.8
RPS20	5|5	0	0.95	0.8
TNIK	2|2	0	0.62	0.8
COPS7A	5|6	0	0.81	0.8
CDK5	3|0	0	0.5	0.8
ACIN1	7|7	0	0.93	0.8
CCT7	39|35	0	0.76	0.8
EIF2B3	7|5	0	0.71	0.8
PQBP1	4|4	0	0.84	0.8
TJP2	8|8	0	0.73	0.8
PGAM5	0|2	0	0.44	0.8
FUS	40|37	0	0.89	0.8
RAB3GAP1	3|9	0	0.65	0.8

PEG10	3|3	0	0.8	0.8
ABI1	4|5	0	0.86	0.8
GNL3L	8|6	0	0.7	0.8
WDR44	3|3	0	0.75	0.8
WNK1	7|7	0	0.97	0.8
PFDN6	2|3	0	0.4	0.8
UCKL1	0|2	0	0.44	0.8
DDX3X	26|21	0	0.73	0.8
CCT6B	5|5	0	0.56	0.8
SRSF2	3|4	0	0.56	0.8
DPYSL2	19|21	0	0.88	0.8
DPYSL3	15|18	0	0.86	0.8
ARHGEF12	0|2	0	0.44	0.8
RPS17L	4|6	0	0.83	0.8
EIF3C	11|13	0	0.83	0.8
USP19	2|2	0	0.5	0.8
UCHL5	4|0	0	0.42	0.8
LRBA	13|12	0	0.49	0.8
CNOT2	2|3	0	0.83	0.8
RPL17	6|7	0	0.87	0.8
GBF1	6|5	0	0.65	0.8
RPS10	8|8	0	0.82	0.8
XIAP	5|7	0	0.65	0.8
RBM10	36|36	0	0.9	0.8
ELOC	4|3	0	0.82	0.8
TCAF1	2|2	0	0.67	0.8
RNF40	7|9	0	0.53	0.8
BTF3	7|7	0	0.68	0.8
C1QBP	7|10	0	0.79	0.8
MTHFD1L	20|20	0	0.64	0.8
ELP2	5|8	0	0.67	0.8
EIF3L	8|8	0	0.84	0.8
AKT2	8|7	0	0.61	0.8
CD2BP2	3|2	0	0.43	0.8
TPD52L2	3|3	0	0.86	0.8
CLIP1	3|0	0	0.22	0.8
USP15	29|27	0	0.96	0.8
TRIM65	5|5	0	0.83	0.8
FLII	6|5	0	0.42	0.8
372266180	2|2	0	1	0.8
PAF1	5|6	0	0.61	0.8
AURKB	2|2	0	1	0.8
SRI	0|2	0	0.4	0.8
C20orf27	2|3	0	0.77	0.8
DCTN2	10|11	0	0.65	0.8

CDK9	5|4	0	0.82	0.8
ARPC5	0|3	0	0.46	0.8
TRAPPC3	2|3	0	0.83	0.8
COPZ1	3|2	0	0.67	0.8
PSMD6	15|14	0	0.81	0.8
DOCK7	6|5	0	0.26	0.8
QARS	6|6	0	0.48	0.8
CHD4	24|22	0	0.94	0.8
CHUK	4|4	0	0.67	0.8
AP2B1	14|14	0	0.93	0.8
CLNS1A	20|24	0	0.68	0.8
CSE1L	39|41	0	1.03	0.8
CSNK2B	3|6	0	0.72	0.8
CSTF1	3|5	0	0.7	0.8
CSTF3	7|8	0	0.91	0.8
DARS	11|12	0	0.77	0.8
DHX15	22|23	0	0.87	0.8
DYNC1H1	103|112	0	1.46	0.8
EEF1G	23|22	0	0.91	0.8
EIF2B1	0|5	0	0.33	0.8
EIF2S3	8|8	0	0.94	0.8
EIF4A1	26|29	0	0.92	0.8
FLNA	99|95	0	0.85	0.8
FLNB	68|67	0	0.89	0.8
XRCC6	23|26	0	0.73	0.8
GTF2B	5|6	0	0.81	0.8
GTF2I	81|77	0	0.96	0.8
HDAC2	13|12	0	0.86	0.8
HNRNPL	18|15	0	0.87	0.8
PARP1	22|21	0	0.65	0.8
AMD1	9|7	0	0.74	0.8
ATP1B3	3|3	0	0.5	0.8
ATP6V1A	21|20	0	0.96	0.8
ATP6V1B2	16|13	0	0.84	0.8
ATP6V1E1	6|7	0	0.84	0.8
CAPN2	0|3	0	0.43	0.8
CCT6A	31|32	0	0.78	0.8
CDK1	12|12	0	0.94	0.8
CLTA	3|2	0	0.67	0.8
CSNK2A2	8|8	0	0.62	0.8
DDB1	28|25	0	0.74	0.8
DSG2	6|4	0	0.77	0.8
EEF2	56|57	0	1.15	0.8
EPS15	4|5	0	0.53	0.8
FKBP3	7|7	0	0.72	0.8

GAPDH	44|61	0	1.13	0.8
GARS	14|14	0	0.93	0.8
GFPT1	6|9	0	0.73	0.8
H2AFZ	3|3	0	0.6	0.8
HTT	0|2	0	0.25	0.8
HMGB1	27|33	0	0.91	0.8
HMGB2	14|15	0	0.97	0.8
HSPD1	100|113	0	0.94	0.8
HSPE1	6|6	0	0.96	0.8
ITGB1	5|5	0	1	0.8
KPNA1	5|5	0	0.67	0.8
KPNB1	33|35	0	0.85	0.8
KPNA2	12|12	0	0.81	0.8
TNPO1	9|8	0	0.68	0.8
LAMC1	4|3	0	0.67	0.8
LGALS3	3|3	0	0.6	0.8
ABLIM1	6|6	0	0.83	0.8
LRPAP1	9|7	0	0.71	0.8
MCM3	40|45	0	0.84	0.8
ME1	2|0	0	0.2	0.8
MYH9	107|101	0	0.8	0.8
NASP	43|46	0	1.02	0.8
NCBP1	5|4	0	0.86	0.8
NFKB2	3|3	0	0.71	0.8
NPM1	8|8	0	0.97	0.8
OSBP	2|3	0	0.77	0.8
PABPC1	14|14	0	0.79	0.8
PAWR	3|4	0	0.74	0.8
PFKL	15|15	0	0.76	0.8
PKM	37|43	0	1.01	0.8
POLA2	6|7	0	0.93	0.8
POLD1	9|8	0	0.81	0.8
PPP1CA	6|7	0	0.81	0.8
PPP1CB	0|3	0	0.5	0.8
PPP1R10	4|4	0	0.84	0.8
PPP2CA	6|6	0	0.89	0.8
PRPS1	11|11	0	0.68	0.8
PRPS2	13|12	0	0.68	0.8
PRPSAP1	15|16	0	0.87	0.8
PRPSAP2	25|20	0	0.78	0.8
PSMA2	8|5	0	0.47	0.8
PSMA5	6|6	0	0.56	0.8
PSMA6	0|3	0	0.13	0.8
PSMA7	6|9	0	0.38	0.8
PSMB1	4|2	0	0.26	0.8

PSMB2	7|7	0	0.58	0.8
PSMB3	2|0	0	0.09	0.8
PSMB4	4|0	0	0.22	0.8
PSMB5	7|2	0	0.35	0.8
PSMB7	2|0	0	0.12	0.8
PSMC1	18|17	0	0.83	0.8
PSMC2	15|13	0	0.79	0.8
PSMC3	16|16	0	0.94	0.8
PSMC5	20|20	0	0.83	0.8
PSMD2	28|28	0	0.7	0.8
PSMD3	22|22	0	1	0.8
PSMD4	6|6	0	0.73	0.8
PSMD8	7|5	0	0.8	0.8
PSMD11	21|22	0	0.92	0.8
TWF1	0|2	0	0.24	0.8
ALDH18A1	30|27	0	0.92	0.8
RANGAP1	5|10	0	0.51	0.8
RARS	7|12	0	0.75	0.8
NELFE	10|10	0	0.91	0.8
UPF1	12|11	0	0.78	0.8
RFC1	4|3	0	0.78	0.8
ABCE1	2|2	0	0.62	0.8
SET	6|8	0	0.74	0.8
SRSF3	14|14	0	0.86	0.8
ITSN1	0|3	0	0.32	0.8
SMARCE1	4|4	0	0.8	0.8
SNRNP70	11|13	0	0.86	0.8
SNRPA1	8|9	0	0.74	0.8
SPTAN1	10|11	0	0.34	0.8
SPTBN1	8|8	0	0.34	0.8
SRP54	12|12	0	0.91	0.8
TRIM21	19|24	0	0.87	0.8
TAF4	24|25	0	0.95	0.8
TFAM	3|4	0	0.78	0.8
GCFC2	2|0	0	0.5	0.8
TOP1	14|16	0	0.65	0.8
TPM4	8|9	0	0.79	0.8
TPR	32|36	0	0.87	0.8
HSP90B1	102|107	0	1.33	0.8
TTC1	3|5	0	0.8	0.8
DNAJC7	8|10	0	0.68	0.8
TTK	0|3	0	0.35	0.8
TXN	5|5	0	0.77	0.8
UBE2N	4|4	0	1	0.8
DEK	11|9	0	0.91	0.8

NAA10	3|6	0	0.67	0.8
MAD1L1	2|2	0	0.62	0.8
EEA1	2|3	0	0.24	0.8
CUL4B	16|18	0	0.92	0.8
CUL1	4|6	0	0.67	0.8
ELP1	21|18	0	0.71	0.8
YBX3	12|13	0	0.93	0.8
COPS3	8|9	0	0.77	0.8
AGPS	3|3	0	0.44	0.8
AP3B1	24|22	0	0.77	0.8
AKR7A2	0|2	0	0.4	0.8
RUVBL1	20|21	0	0.9	0.8
EIF3A	24|22	0	0.74	0.8
EIF3B	11|16	0	0.76	0.8
EIF3D	7|7	0	1	0.8
EIF3I	18|16	0	0.76	0.8
EDF1	9|7	0	0.84	0.8
IQGAP1	37|38	0	0.88	0.8
SYNJ1	3|2	0	0.25	0.8
FUBP1	11|11	0	0.86	0.8
EIF2B5	5|4	0	0.58	0.8
EIF2S2	5|3	0	0.52	0.8
CPNE1	0|3	0	0.32	0.8
ST13	7|8	0	0.77	0.8
AP3D1	14|21	0	0.68	0.8
UBE2M	3|2	0	0.71	0.8
BTAF1	4|3	0	0.34	0.8
AMPD2	5|5	0	0.54	0.8
EIF2S1	5|7	0	0.53	0.8
FASN	152|144	0	0.96	0.8
FEN1	9|9	0	0.95	0.8
HSPA9	44|44	0	1.12	0.8
PRKAR2A	3|5	0	0.8	0.8
SNRPD3	3|3	0	0.5	0.8
WARS	25|23	0	0.91	0.8
RAB11B	6|8	0	0.78	0.8
EFTUD2	27|29	0	0.83	0.8
HOMER1	5|5	0	0.91	0.8
BAG3	0|4	0	0.32	0.8
BAG2	4|2	0	0.71	0.8
KIF1A	4|3	0	0.47	0.8
CAD	38|38	0	0.56	0.8
COPA	33|34	0	0.66	0.8
DBN1	0|2	0	0.31	0.8
DSP	15|13	0	0.44	0.8

EPRS	23|27	0	0.82	0.8
FARSA	10|11	0	0.88	0.8
FDFT1	5|4	0	0.82	0.8
FRG1	8|7	0	0.57	0.8
HNRNPU	26|26	0	0.96	0.8
KIF5B	47|47	0	0.95	0.8
KIF5C	16|16	0	0.86	0.8
KIF11	10|12	0	0.86	0.8
MCM2	39|43	0	0.95	0.8
SFSWAP	0|3	0	0.21	0.8
SNRPD2	3|5	0	0.64	0.8
TTC4	2|2	0	0.44	0.8
BAG6	18|22	0	0.69	0.8
COIL	10|9	0	0.78	0.8
EIF1AY	11|10	0	0.91	0.8
FAM50A	7|6	0	0.72	0.8
RABEP1	3|5	0	0.62	0.8
PDCD5	7|8	0	0.91	0.8
HGS	3|2	0	0.42	0.8
SCAF11	7|7	0	0.85	0.8
ZW10	6|4	0	0.65	0.8
BUB3	6|5	0	0.76	0.8
LRRFIP1	4|6	0	0.62	0.8
MTA2	15|16	0	0.91	0.8
COPB2	19|17	0	0.59	0.8
TXNL1	0|2	0	0.17	0.8
PPIG	4|6	0	0.67	0.8
SNRNP40	3|4	0	0.88	0.8
DDX23	5|3	0	0.62	0.8
EIF4E2	3|2	0	0.4	0.8
CLTC	42|42	0	0.97	0.8
SCAMP1	0|2	0	0.36	0.8
CIR1	2|0	0	0.36	0.8
SEC22B	0|2	0	0.36	0.8
WTAP	3|3	0	0.75	0.8
ACTN4	48|53	0	0.68	0.8
CAPZB	11|12	0	0.81	0.8
MARK2	3|2	0	0.62	0.8
MTOR	5|2	0	0.52	0.8
HDAC1	10|9	0	0.79	0.8
IDE	5|5	0	0.83	0.8
MYO6	4|4	0	0.4	0.8
TLE3	5|5	0	0.59	0.8
NUP214	13|14	0	0.72	0.8
FXR1	3|4	0	0.47	0.8

AKAP12	32|33	0	0.85	0.8
GOLGA5	3|3	0	0.67	0.8
USP14	3|5	0	0.64	0.8
GAK	3|3	0	1	0.8
GNL1	2|4	0	0.75	0.8
HIST1H1C	6|5	0	0.88	0.8
HMGB3	4|4	0	0.8	0.8
HSPA1B	78|79	0	0.86	0.8
ROCK1	28|27	0	0.68	0.8
CCDC6	2|0	0	0.13	0.8
SMC3	26|30	0	0.82	0.8
DMXL1	0|2	0	0.14	0.8
SRSF4	6|4	0	0.77	0.8
FARSB	13|11	0	0.87	0.8
ARPC3	3|2	0	0.71	0.8
ACTR2	4|6	0	0.71	0.8
TRIM28	31|32	0	0.79	0.8
PSME3	12|15	0	0.76	0.8
PRDX2	9|8	0	0.89	0.8
PLIN3	3|3	0	0.75	0.8
HNRNPR	7|10	0	0.71	0.8
DCAF7	3|4	0	0.74	0.8
MRPS31	0|2	0	0.17	0.8
AKAP8	0|2	0	0.4	0.8
STUB1	13|10	0	0.85	0.8
CWC27	2|4	0	0.63	0.8
SMNDC1	3|3	0	1	0.8
EIF1B	3|2	0	0.62	0.8
SF3A1	23|25	0	0.88	0.8
BCKDK	5|6	0	0.69	0.8
PAK4	7|8	0	0.7	0.8
GOLGA3	4|3	0	0.47	0.8
MAP1B	53|55	0	0.83	0.8
MCM6	26|25	0	0.84	0.8
MCM7	47|47	0	1.05	0.8
MFAP1	30|30	0	0.94	0.8
MYH10	107|108	0	0.87	0.8
CCT3	46|50	0	0.71	0.8
RPL10	4|4	0	0.76	0.8
RBM12	6|8	0	0.82	0.8
TFG	4|4	0	0.36	0.8
IK	8|9	0	0.94	0.8
NDC80	0|2	0	0.33	0.8
PRMT5	54|55	0	0.58	0.8
TAB1	25|23	0	0.95	0.8

KRT1	37|37	0	0.76	0.8
CAPZA1	9|10	0	0.66	0.8
CAPZA2	8|6	0	0.67	0.8
DNAJB1	7|7	0	0.72	0.8
NSF	3|4	0	0.61	0.8
PIN4	11|10	0	0.68	0.8
POLE	3|0	0	0.5	0.8
RANBP2	38|37	0	0.93	0.8
TLN1	51|55	0	1.15	0.8
VARS	28|25	0	0.75	0.8
AIMP2	4|3	0	0.82	0.8
SMC1A	28|28	0	0.82	0.8
COG5	0|3	0	0.38	0.8
ATP5PD	2|3	0	0.71	0.8
CAP2	4|4	0	0.73	0.8
SYNCRIP	10|13	0	0.79	0.8
CHERP	5|10	0	0.51	0.8
IPO8	7|7	0	0.61	0.8
IPO7	8|10	0	0.78	0.8
SLU7	2|3	0	0.48	0.8
CCT4	35|35	0	0.65	0.8
CCT2	57|58	0	0.88	0.8
SMC2	22|26	0	0.63	0.8
PRPF8	42|43	0	0.6	0.8
PDLIM5	4|8	0	0.48	0.8
HEXIM1	2|3	0	0.71	0.8
IVNS1ABP	15|13	0	0.81	0.8
GNAI3	3|0	0	0.5	0.8
PSMC4	19|21	0	0.83	0.8
IGF2BP1	4|4	0	0.44	0.8
CCT8	41|40	0	0.86	0.8
HSPA8	70|73	0	0.85	0.8
IQGAP2	18|13	0	0.78	0.8
9-Sep	12|13	0	0.96	0.8
CLPX	7|7	0	0.56	0.8
RUVBL2	23|27	0	0.85	0.8
COPS8	5|5	0	0.74	0.8
SUB1	19|19	0	0.55	0.8
MCM5	39|39	0	0.89	0.8
RBM3	5|7	0	0.8	0.8
U2AF1	5|4	0	0.82	0.8
RALBP1	2|2	0	0.57	0.8
SF3A3	8|10	0	0.75	0.8
HNRNPA0	10|12	0	0.81	0.8
TOMM34	6|7	0	0.74	0.8

STIP1	45|46	0	0.8	0.8
COPS6	10|11	0	0.86	0.8
GCN1	74|67	0	1.21	0.8
SF3B2	4|9	0	0.62	0.8
SRSF1	6|6	0	0.63	0.8
SKIV2L	7|6	0	0.67	0.8
SNRPD1	7|7	0	0.67	0.8
SRP72	7|6	0	0.79	0.8
WASF2	0|2	0	0.31	0.8
NUDT21	10|10	0	0.69	0.8
HNRNPUL1	8|8	0	0.67	0.8
POLR3A	0|2	0	0.22	0.8
TRIO	0|2	0	0.33	0.8
VCP	86|90	0	1.13	0.8
NUP50	10|8	0	0.69	0.8
STRAP	12|12	0	1	0.8
NISCH	3|2	0	0.71	0.8
DCTN3	2|0	0	0.29	0.8
COPE	12|10	0	0.85	0.8
STK38	45|43	0	0.77	0.8
HSP90AB1	93|87	0	0.78	0.8
DDX42	22|28	0	0.89	0.8
COMMD3	3|2	0	0.71	0.8
CCT5	47|48	0	0.84	0.8
MACF1	12|8	0	0.5	0.8
APPL1	0|3	0	0.46	0.8
AHSA1	3|3	0	0.86	0.8
CD2AP	16|13	0	0.48	0.8
COPG2	18|17	0	0.84	0.8
ALDH1L1	4|3	0	0.7	0.8
RABGAP1	2|0	0	0.13	0.8
OGA	9|8	0	0.74	0.8
SNW1	7|9	0	0.8	0.8
AP2A2	4|5	0	0.86	0.8
EPB41L3	21|19	0	0.67	0.8
KPNA6	5|5	0	0.67	0.8
LSM4	5|5	0	0.71	0.8
MYCBP	10|8	0	0.88	0.8
QPCT	12|10	0	0.47	0.8
RAB3GAP2	5|4	0	0.37	0.8
SF3B3	27|25	0	0.72	0.8
SF3B1	38|42	0	0.82	0.8
PRPF6	3|5	0	0.57	0.8
TNPO3	6|7	0	0.9	0.8
YWHAG	14|13	0	0.96	0.8

TBK1	0|3	0	0.38	0.8
SAP30BP	2|2	0	0.38	0.8
NRBP1	3|2	0	0.5	0.8
STRN4	2|2	0	0.57	0.8
IARS	26|23	0	0.94	0.8
DHX38	15|16	0	0.72	0.8
CCDC22	4|5	0	0.62	0.8
SNRNP200	30|41	0	0.67	0.8
C19orf53	6|4	0	0.74	0.8
FAM32A	5|4	0	0.72	0.8
PRDX3	10|13	0	0.71	0.8
NUDT5	5|5	0	0.87	0.8
UBL4A	5|5	0	0.69	0.8
GNPAT	3|0	0	0.5	0.8
EIF2B2	3|3	0	0.67	0.8
LIMD1	0|3	0	0.43	0.8
C11orf58	2|3	0	0.77	0.8
DNAJC8	5|4	0	0.9	0.8
PUF60	26|28	0	0.86	0.8
NOMO1	6|6	0	0.86	0.8
RTCB	13|15	0	0.92	0.8
CORO1C	3|2	0	0.48	0.8
EDC4	11|12	0	0.92	0.8
PELP1	4|7	0	0.79	0.8
SND1	29|31	0	0.94	0.8
AFF4	4|2	0	0.63	0.8
LSM1	2|2	0	0.57	0.8
HTATSF1	7|8	0	0.53	0.8
TMOD3	2|0	0	0.29	0.8
PIK3R4	2|0	0	0.24	0.8
CYFIP1	5|0	0	0.23	0.8
ARHGEF10	3|2	0	0.48	0.8
TTC37	8|9	0	0.87	0.8
DCAF1	4|4	0	0.31	0.8
SART3	14|19	0	0.69	0.8
KNTC1	3|2	0	0.23	0.8
SLK	11|11	0	0.7	0.8
ZFYVE16	2|4	0	0.48	0.8
BCLAF1	50|51	0	1.12	0.8
EIF4A3	8|11	0	0.69	0.8
CEP170	8|4	0	0.73	0.8
DDX46	90|87	0	0.85	0.8
TBC1D4	5|4	0	0.62	0.8
WASHC5	2|0	0	0.17	0.8
NCAPD2	21|20	0	0.59	0.8

SEC16A	6|8	0	0.42	0.8
PDAP1	11|10	0	0.89	0.8
SCAF8	3|4	0	0.82	0.8
PPP6R1	3|5	0	0.64	0.8
TRAPPC8	3|0	0	0.17	0.8
DIS3	2|4	0	0.39	0.8
STK38L	28|26	0	0.78	0.8
XPO7	16|15	0	0.79	0.8
POP1	4|4	0	0.62	0.8
PDS5B	0|2	0	0.4	0.8
MRPS27	0|3	0	0.19	0.8
TNRC6B	12|12	0	0.62	0.8
TAB2	3|5	0	0.64	0.8
CLASP2	10|10	0	0.85	0.8
ANKLE2	2|0	0	0.33	0.8
6-Sep	0|9	0	0.46	0.8
RTF1	6|8	0	0.76	0.8
RCOR1	2|2	0	0.73	0.8
PRRC2C	9|12	0	0.59	0.8
RPRD2	10|6	0	0.6	0.8
CLUH	11|12	0	0.61	0.8
NUP160	10|8	0	0.71	0.8
BICD2	3|5	0	0.67	0.8
DMXL2	3|2	0	0.67	0.8
DNAJC13	0|3	0	0.17	0.8
WASHC4	3|0	0	0.25	0.8
WDR7	0|2	0	0.21	0.8
HAUS5	4|3	0	0.56	0.8
USP24	8|8	0	0.54	0.8
LARP1	4|6	0	0.54	0.8
NCAPH	6|5	0	0.33	0.8
MTREX	0|2	0	0.33	0.8
HECTD1	15|16	0	0.51	0.8
LARP7	11|11	0	0.85	0.8
GEMIN5	19|20	0	0.67	0.8
SAMHD1	0|3	0	0.35	0.8
FAM98A	4|4	0	0.39	0.8
AAR2	4|5	0	0.82	0.8
RPAP1	7|5	0	0.77	0.8
LSM14A	24|25	0	0.86	0.8
POLDIP2	0|3	0	0.23	0.8
PRPF31	14|15	0	0.76	0.8
GAPVD1	10|16	0	0.72	0.8
GPKOW	5|5	0	0.95	0.8
DCAF8	9|10	0	0.86	0.8

HOOK1	5|4	0	0.5	0.8
UBR5	3|0	0	0.26	0.8
EIF5B	27|23	0	0.88	0.8
TRIM33	5|4	0	0.82	0.8
TXNDC12	11|11	0	0.75	0.8
TMA7	6|7	0	0.59	0.8
TRMT6	3|4	0	0.7	0.8
MRPS7	3|3	0	1	0.8
LUC7L2	27|24	0	0.73	0.8
SBDS	0|2	0	0.22	0.8
RTRAF	8|9	0	0.94	0.8
SF3B6	2|2	0	0.62	0.8
CIAO2B	6|7	0	0.93	0.8
PTRH2	3|2	0	0.77	0.8
COPG1	20|19	0	0.87	0.8
COPS4	14|13	0	0.86	0.8
JPT1	0|3	0	0.38	0.8
CPSF3	5|3	0	0.8	0.8
DBR1	2|0	0	0.5	0.8
CNOT1	14|13	0	0.66	0.8
SRRM2	37|35	0	0.84	0.8
ANKFY1	24|26	0	0.81	0.8
HYPK	3|3	0	0.92	0.8
CHMP5	2|2	0	0.8	0.8
ZCCHC17	2|3	0	0.77	0.8
RSRC1	5|5	0	0.56	0.8
WAC	5|4	0	0.69	0.8
ARL6IP4	16|15	0	0.97	0.8
POLA1	13|15	0	0.78	0.8
CPSF2	2|3	0	0.71	0.8
KLHDC4	2|0	0	0.25	0.8
ILF3	17|21	0	0.89	0.8
VPS50	4|2	0	0.55	0.8
ERCC6L	7|5	0	0.73	0.8
VPS13C	2|5	0	0.54	0.8
CWC25	6|5	0	0.65	0.8
ALKBH5	2|2	0	0.62	0.8
FOCAD	2|0	0	0.5	0.8
DNAAF5	5|6	0	0.61	0.8
COMMD4	2|2	0	1	0.8
GID8	2|2	0	0.67	0.8
PRPF39	0|2	0	0.5	0.8
USP47	0|2	0	0.5	0.8
ZWILCH	3|2	0	0.71	0.8
SCYL2	15|15	0	0.88	0.8

GPATCH1	3|2	0	0.18	0.8
ATG2B	3|3	0	0.92	0.8
ELP3	8|8	0	0.62	0.8
ELAC2	6|6	0	1	0.8
SLC4A1AP	0|3	0	0.22	0.8
CCAR1	9|10	0	0.54	0.8
11-Sep	13|14	0	0.96	0.8
PPP6R3	9|10	0	0.95	0.8
RIOK2	2|0	0	0.29	0.8
TXLNG	3|3	0	0.5	0.8
UBAP2	2|2	0	0.27	0.8
PBK	3|0	0	0.5	0.8
BAIAP2L1	2|2	0	0.42	0.8
PIMREG	2|2	0	0.8	0.8
EML4	6|6	0	0.92	0.8
DDX49	0|2	0	0.44	0.8
RNF20	10|9	0	0.61	0.8
PARD3	3|3	0	0.5	0.8
EIF4ENIF1	2|0	0	0.33	0.8
LARS	18|20	0	0.85	0.8
WRNIP1	9|8	0	0.87	0.8
GRIPAP1	6|5	0	0.59	0.8
SH3GLB2	4|4	0	0.57	0.8
MCCC1	5|3	0	0.76	0.8
KIF15	6|2	0	0.5	0.8
PCNP	3|4	0	0.74	0.8
AVEN	2|0	0	0.31	0.8
CORO1B	0|2	0	0.4	0.8
ANKHD1-EIF4EBP3	6|4	0	0.61	0.8
GATAD2B	3|0	0	0.3	0.8
UBR4	2|3	0	0.67	0.8
NUFIP2	8|7	0	0.81	0.8
GPHN	6|3	0	0.67	0.8
VPS18	0|2	0	0.15	0.8
CWC22	10|8	0	0.78	0.8
HIST1H3F	2|2	0	0.44	0.8
MYL6	7|9	0	0.78	0.8
HIST1H2BB	4|0	0	0.4	0.8
HIST1H2BD	4|4	0	0.53	0.8
MRPS12	3|3	0	1	0.8
EEF1B2	8|8	0	0.91	0.8
CCAR2	14|14	0	0.67	0.8
EPS15L1	4|7	0	0.71	0.8
RBM25	6|5	0	0.73	0.8
VPS11	2|0	0	0.4	0.8

EXOC4	9|7	0	0.8	0.8
TRAPPC11	0|2	0	0.5	0.8
RELA	5|2	0	0.67	0.8
HSPA2	16|18	0	0.72	0.8
JUP	10|8	0	0.78	0.8
UBE2O	12|13	0	0.6	0.8
CLSPN	0|2	0	0.33	0.8
NCAPG	13|8	0	0.7	0.8
P3H1	3|3	0	0.92	0.8
MMS19	6|7	0	0.79	0.8
RPS18	7|5	0	0.69	0.8
VPS52	2|0	0	0.33	0.8
VPS16	5|2	0	0.54	0.8
COPS7B	2|3	0	0.59	0.8
NUCKS1	7|8	0	0.79	0.8
SNRPN	11|11	0	0.64	0.8
KLC2	13|12	0	0.64	0.8
C12orf43	2|0	0	0.33	0.8
NT5DC2	10|8	0	0.77	0.8
VPS33A	2|0	0	0.17	0.8
RSRC2	11|10	0	0.81	0.8
MRPS34	4|2	0	0.41	0.8
TRIR	0|2	0	0.24	0.8
DDA1	3|0	0	0.4	0.8
NUP37	3|4	0	0.7	0.8
PDCL3	0|2	0	0.18	0.8
ASPSCR1	9|11	0	0.75	0.8
WDR77	18|19	0	0.74	0.8
C1orf35	2|0	0	0.44	0.8
NKAP	2|0	0	0.2	0.8
RPAP3	2|2	0	0.29	0.8
DHX40	4|3	0	0.61	0.8
TBL1XR1	5|4	0	0.69	0.8
SNIP1	4|4	0	0.73	0.8
RABL6	11|9	0	0.87	0.8
MYH14	17|14	0	0.85	0.8
PIP4K2C	0|2	0	0.33	0.8
RABEP2	0|3	0	0.5	0.8
NUP85	5|5	0	0.87	0.8
ATAT1	2|0	0	0.33	0.8
VCPIP1	3|3	0	0.71	0.8
NAA50	0|2	0	0.4	0.8
PAAF1	3|3	0	0.86	0.8
WDR61	4|6	0	0.71	0.8
UBXN6	3|4	0	0.48	0.8

DICER1	0|2	0	0.24	0.8
TCP1	51|51	0	0.9	0.8
ANP32E	5|5	0	1	0.8
SEH1L	4|5	0	0.82	0.8
SF3B5	4|4	0	1	0.8
EPPK1	59|57	0	0.8	0.8
HNRNPC	10|10	0	0.95	0.8
HUWE1	64|62	0	0.78	0.8
RIOK1	11|10	0	0.45	0.8
REPS1	2|2	0	0.47	0.8
CCDC8	3|2	0	0.83	0.8
NSRP1	16|16	0	0.96	0.8
ABRAXAS2	5|6	0	0.92	0.8
SON	3|3	0	0.8	0.8
ANKRD17	5|4	0	0.62	0.8
HOOK3	0|2	0	0.27	0.8
PPIL3	3|2	0	0.71	0.8
PPP4R3A	9|10	0	0.73	0.8
HDGFL2	12|14	0	0.85	0.8
PSRC1	2|0	0	0.4	0.8
NFATC2IP	3|3	0	0.63	0.8
PRPF38A	9|10	0	0.83	0.8
RBM17	0|2	0	0.24	0.8
POLR2C	5|3	0	0.8	0.8
RECQL	5|5	0	0.71	0.8
RPS24	2|2	0	0.57	0.8
SARNP	4|4	0	0.84	0.8
CALD1	0|2	0	0.21	0.8
MICALL1	2|3	0	0.71	0.8
MYL12B	6|6	0	0.6	0.8
CCDC97	0|2	0	0.31	0.8
RPLP0	7|9	0	0.86	0.8
NAA15	11|15	0	0.87	0.8
PI4KA	9|9	0	0.86	0.8
EXOSC6	4|5	0	0.64	0.8
RNPS1	5|4	0	0.37	0.8
PRRC2A	22|18	0	0.83	0.8
ELMO2	4|2	0	0.38	0.8
LRPPRC	82|83	0	0.94	0.8
RAVER1	22|24	0	0.96	0.8
CCDC124	14|11	0	0.83	0.8
KCTD12	3|4	0	0.82	0.8
KIF23	3|0	0	0.43	0.8
MRRF	2|2	0	0.62	0.8
CMBL	12|11	0	0.64	0.8

PPIL4	23|23	0	0.81	0.8
DPP9	0|2	0	0.19	0.8
TAF15	15|16	0	0.76	0.8
FAM76B	2|0	0	0.44	0.8
ZMAT2	2|0	0	0.19	0.8
SKA1	5|3	0	0.73	0.8
MAP3K7	10|13	0	0.57	0.8
AP1B1	14|15	0	0.85	0.8
HMGA1	5|6	0	0.79	0.8
PPIL2	0|2	0	0.29	0.8
BOD1L1	5|4	0	0.78	0.8
PSMA1	4|7	0	0.32	0.8
SNX6	2|3	0	0.67	0.8
ARHGAP42	2|0	0	0.36	0.8
TAB3	2|3	0	0.5	0.8
FAM133B	0|2	0	0.22	0.8
TCEANC2	0|2	0	0.22	0.8
PROSER2	3|4	0	0.78	0.8
ALDH16A1	2|2	0	0.32	0.8
CAMK2D	8|6	0	0.74	0.8
EIF2B4	11|10	0	0.88	0.8
FAM98B	6|9	0	0.7	0.8
TRAPPC5	2|3	0	0.56	0.8
TDRP	0|2	0	0.27	0.8
TJP1	3|4	0	0.74	0.8
TXLNA	11|10	0	0.91	0.8
CHMP4B	4|4	0	0.94	0.8
PPFIA1	2|3	0	0.71	0.8
CSNK2A1	21|22	0	0.75	0.8
NCAM1	5|3	0	0.7	0.8
EIF6	0|2	0	0.33	0.8
OGT	6|6	0	0.48	0.8
MCM4	39|41	0	0.85	0.8
WASHC1	2|2	0	0.62	0.8
EIF4G1	31|37	0	0.82	0.8
KLC1	19|18	0	0.94	0.8
PRMT1	16|18	0	0.91	0.8
GANAB	67|69	0	1.37	0.8
G3BP1	21|23	0	0.75	0.8
MIA3	6|7	0	0.43	0.8
ACACA	27|28	0	0.75	0.8
LUC7L	13|11	0	0.76	0.8
KLC4	13|10	0	0.79	0.8
SF1	7|6	0	0.93	0.8
HDLBP	14|17	0	0.81	0.8

NCKAP1	8|7	0	0.65	0.8
ECI2	4|3	0	0.82	0.8
LTBP1	5|4	0	0.4	0.8
AP3M1	12|12	0	0.96	0.8
ELOB	4|2	0	0.75	0.8
BLOC1S3	3|3	0	0.75	0.8
ATP6V1H	3|3	0	0.8	0.8

Supplementary Table 6: BioID data for MFGE8 after SAINT analysis

BioID data for MFGE8-BirA-FLAG in Flp-In T-REx HEK293 cells. Preys with BFDR ≤ 1% are considered high-confidence and shown in Green here. Bait for BioID experiment was BirA-FLAG tagged MFGE8 protein. Prey Gene is the Official Gene Symbol (also from NCBI). Spectral counts for the prey (Spec, separated by "I" delimiter, Averaged probability across replicates (AvgP), Fold Change (counts in the purification divided by counts in the controls plus small factor to prevent division by 0) and Bayesian FDR (BFDR) are listed for each bait-prey relationship and are directly from the SAINTexpress output. 24 controls (cells expressing either BirA-FLAG alone, BirA-FLAG-NLS or BirA-FLAG-GFP) were compressed to 12 to increase the stringency of the SAINT analysis. The experiment was repeated twice.

PreyGene	Spec	AvgSpec	AvgP	FoldChange	BFDR
HSPB1	20|15	17.5	0.98	3.82	0
RAE1	49|45	47	1	3.94	0
RUNX3	5|4	4.5	1	45	0
RAB6B	4|6	5	1	50	0
MRPS11	8|9	8.5	0.99	25.5	0
ALDH1B1	14|13	13.5	0.98	4.26	0.01
ALDH1L2	3|4	3.5	0.98	35	0.01
DCXR	3|4	3.5	0.98	35	0.01
DAD1	3|3	3	0.96	30	0.01
R3HDM2	3|3	3	0.96	30	0.01
GTPBP2	3|3	3	0.96	30	0.01
TRIM13	3|3	3	0.96	30	0.01
GLI2	6|4	5	0.95	30	0.02
FLOT1	18|19	18.5	0.94	3.08	0.02
FAHD2A	2|5	3.5	0.9	35	0.03
SDF4	2|4	3	0.9	30	0.03
MYO19	5|7	6	0.91	24	0.03
ARFRP1	2|3	2.5	0.89	25	0.04
ABCB8	3|2	2.5	0.89	25	0.04
HOXC11	3|2	2.5	0.89	25	0.04
DENND1A	3|2	2.5	0.89	25	0.04
ACTC1	58|68	63	0.87	8.49	0.05
THNSL1	7|4	5.5	0.87	22	0.06
SIK3	5|5	5	0.87	20	0.06
STK11IP	4|3	3.5	0.86	21	0.06
ALDH2	3|3	3	0.81	18	0.07
SP9	2|2	2	0.81	20	0.07
AGPS	4|6	5	0.85	20	0.07
TIMM13	2|2	2	0.81	20	0.07
EMC4	2|2	2	0.81	20	0.07
GLUD2	5|5	5	0.8	15	0.09
CEP85	43|42	42.5	0.79	4.72	0.09

SASS6	14|15	14.5	0.8	8.7	0.09
BCKDHA	8|11	9.5	0.78	11.4	0.1
FOXP4	12|13	12.5	0.77	3.41	0.1
FOXP2	15|14	14.5	0.79	9.67	0.1
ATN1	23|21	22	0.76	4.89	0.11
HIPK2	4|2	3	0.75	18	0.11
TMED4	4|5	4.5	0.75	13.5	0.11
PTK7	2|3	2.5	0.7	15	0.12
CALR	3|2	2.5	0.7	15	0.12
IDH3A	3|2	2.5	0.7	15	0.12
DARS2	28|29	28.5	0.75	4.62	0.12
ALDH7A1	8|4	6	0.67	8	0.13
TMED10	23|19	21	0.65	3.04	0.14
MIB2	8|4	6	0.67	10.29	0.14
RBPMS	6|5	5.5	0.64	8.25	0.15
TMED1	4|2	3	0.61	12	0.15
TRIM65	5|8	6.5	0.61	7.8	0.16
DNAJB2	2|2	2	0.6	12	0.16
RAB2A	3|2	2.5	0.54	10	0.17
HIST1H1D	22|18	20	0.55	4.62	0.17
DNAJA3	26|33	29.5	0.54	3.03	0.18
SP1	6|8	7	0.54	6.46	0.18
NFS1	5|2	3.5	0.52	8.4	0.19
SLC25A19	4|8	6	0.53	5.54	0.19
HADHB	7|8	7.5	0.51	5.62	0.2
TNRC18	5|5	5	0.52	6.67	0.2
PKP4	14|11	12.5	0.5	3.06	0.21
RAB8A	6|7	6.5	0.49	5.57	0.22
MBNL1	4|0	2	0.5	20	0.22
ALDH1A3	0|3	1.5	0.48	15	0.23
CTU2	0|3	1.5	0.48	15	0.23
ADAM10	3|0	1.5	0.48	15	0.23
APEX1	3|0	1.5	0.48	15	0.23
ROS1	0|3	1.5	0.48	15	0.23
SPG7	3|3	3	0.48	7.2	0.23
GPAA1	3|0	1.5	0.48	15	0.23
LOXL1	0|3	1.5	0.48	15	0.23
SLC30A9	0|3	1.5	0.48	15	0.23
EXOC5	3|0	1.5	0.48	15	0.23
RAB38	0|3	1.5	0.48	15	0.23
PAPLN	0|3	1.5	0.48	15	0.23
STIL	2|3	2.5	0.48	7.5	0.27
SLC25A20	4|0	2	0.46	12	0.28
MAD1L1	0|5	2.5	0.48	15	0.28
PUM2	7|5	6	0.47	5.54	0.28

NDUFV2	4|0	2	0.46	12	0.28
ZNF566	4|0	2	0.46	12	0.28
PLOD2	0|4	2	0.46	12	0.28
GLI3	9|6	7.5	0.45	5	0.3
POTEE	0|19	9.5	0.45	6.71	0.3
FSCN1	2|2	2	0.44	8	0.3
TAP1	3|3	3	0.43	7.2	0.31
EIF3CL	0|12	6	0.41	5.54	0.31
ATP2C1	2|4	3	0.43	7.2	0.31
ZNF703	9|6	7.5	0.43	4.5	0.31
CDK18	0|3	1.5	0.4	9	0.32
RAB7A	0|3	1.5	0.4	9	0.32
MICU1	3|0	1.5	0.4	9	0.32
SHPK	0|5	2.5	0.41	5	0.32
SUN1	11|14	12.5	0.41	2.88	0.32
GPX1	0|2	1	0.4	10	0.33
DALRD3	0|2	1	0.4	10	0.33
SETD5	2|0	1	0.4	10	0.33
LRRC8D	0|2	1	0.4	10	0.33
FZD6	0|2	1	0.4	10	0.33
DGKA	0|2	1	0.4	10	0.33
AHR	0|2	1	0.4	10	0.33
RUNX1	0|2	1	0.4	10	0.33
CBFB	2|0	1	0.4	10	0.33
COL6A1	2|0	1	0.4	10	0.33
RBMS1	2|0	1	0.4	10	0.33
STXBP1	0|2	1	0.4	10	0.33
ALDH4A1	0|2	1	0.4	10	0.33
KRAS	0|2	1	0.4	10	0.33
RELN	0|2	1	0.4	10	0.33
RAC3	0|2	1	0.4	10	0.33
HLA-E	0|2	1	0.4	10	0.33
HOXA11	0|2	1	0.4	10	0.33
SCAMP2	0|2	1	0.4	10	0.33
PCGF3	2|0	1	0.4	10	0.33
AGPAT2	0|2	1	0.4	10	0.33
PADI2	0|2	1	0.4	10	0.33
RYBP	0|2	1	0.4	10	0.33
RRAS2	0|2	1	0.4	10	0.33
MRPL15	0|2	1	0.4	10	0.33
CBX6	0|2	1	0.4	10	0.33
FAM168A	0|2	1	0.4	10	0.33
DNAJC16	0|2	1	0.4	10	0.33
MEX3C	0|2	1	0.4	10	0.33
RNF111	2|0	1	0.4	10	0.33

RETSAT	0|2	1	0.4	10	0.33
FAM120C	0|2	1	0.4	10	0.33
WDR60	2|0	1	0.4	10	0.33
ARHGEF40	0|2	1	0.4	10	0.33
DECR2	2|0	1	0.4	10	0.33
CCDC180	0|2	1	0.4	10	0.33
DHRS4	0|2	1	0.4	10	0.33
PPP3CB	2|0	1	0.4	10	0.33
HOXD11	2|0	1	0.4	10	0.33
MRPS14	0|2	1	0.4	10	0.33
RIOX1	2|0	1	0.4	10	0.33
TANC2	0|2	1	0.4	10	0.33
SRCIN1	0|2	1	0.4	10	0.33
ZMIZ2	2|0	1	0.4	10	0.33
CEP295	0|2	1	0.4	10	0.33
MSI2	0|2	1	0.4	10	0.33
USP54	0|2	1	0.4	10	0.33
NFXL1	0|2	1	0.4	10	0.33
SOWAHA	0|2	1	0.4	10	0.33
ZNF227	2|0	1	0.4	10	0.33
MTERF4	0|2	1	0.4	10	0.33
RBPMS2	2|0	1	0.4	10	0.33
NSUN4	0|2	1	0.4	10	0.33
GPSM1	6|8	7	0.37	3.23	0.43
TCF12	6|8	7	0.38	4.42	0.43
NCOA1	3|7	5	0.39	5	0.43
ERAL1	3|5	4	0.38	5.33	0.43
KAT6A	7|6	6.5	0.34	4.11	0.44
PIP5K1A	5|0	2.5	0.34	5	0.44
SUN2	3|4	3.5	0.34	5.25	0.44
GMDS	7|5	6	0.34	3.6	0.44
TONSL	4|6	5	0.34	4.29	0.44
KCMF1	3|4	3.5	0.34	5.25	0.44
STT3A	23|16	19.5	0.34	2.44	0.44
RPS25	3|3	3	0.33	5.14	0.45
CPS1	0|6	3	0.33	4.5	0.45
IQSEC1	0|3	1.5	0.32	6	0.45
CPEB3	3|0	1.5	0.32	6	0.45
TUBA4A	0|91	45.5	0.33	2.21	0.45
RERE	21|24	22.5	0.31	3.51	0.45
VARS2	5|6	5.5	0.32	4.12	0.45
FOXP1	21|25	23	0.33	3.58	0.45
SUMF2	2|0	1	0.3	6	0.46
NDUFB4	2|0	1	0.3	6	0.46
CDK5RAP2	3|2	2.5	0.31	5	0.46

CAMLG	2|0	1	0.3	6	0.46
SEL1L	0|2	1	0.3	6	0.46
RHBDD3	0|2	1	0.3	6	0.46
EFR3B	2|0	1	0.3	6	0.46
RAD54L2	3|2	2.5	0.31	5	0.46
C15orf39	2|0	1	0.3	6	0.46
UGGT1	0|2	1	0.3	6	0.46
AARS2	0|2	1	0.3	6	0.46
FDXR	0|2	1	0.3	6	0.46
NLRX1	2|4	3	0.31	4.5	0.46
ACAD10	0|2	1	0.3	6	0.46
PIGU	0|2	1	0.3	6	0.46
GPT2	0|2	1	0.3	6	0.46
PGM2L1	2|0	1	0.3	6	0.46
SRSF2	2|3	2.5	0.28	5	0.48
IDH2	0|3	1.5	0.28	4.5	0.48
NUDT1	3|0	1.5	0.28	4.5	0.48
ZFHX4	7|8	7.5	0.3	3.75	0.48
CPEB4	9|7	8	0.29	3	0.48
RNF214	10|6	8	0.29	3.31	0.48
PPT1	4|0	2	0.27	4	0.49
FNDC3A	3|3	3	0.27	4.5	0.49
VDAC3	40|38	39	0.27	2.71	0.49
AMFR	4|4	4	0.27	4	0.49
GMPPA	3|3	3	0.26	4.5	0.49
CHCHD3	3|5	4	0.28	4	0.49
COQ8B	5|0	2.5	0.26	3	0.49
RAB33B	3|3	3	0.26	4.5	0.49
ARVCF	3|3	3	0.24	4	0.5
MED16	2|2	2	0.24	4.8	0.5
NRM	2|0	1	0.22	4	0.5
ZNF503	14|15	14.5	0.24	3.41	0.5
NUP35	0|2	1	0.22	4	0.5
PARS2	2|0	1	0.22	4	0.5
ACADSB	12|8	10	0.22	2.5	0.51
ATP5PB	3|3	3	0.22	4	0.51
DBT	3|0	1.5	0.21	3.6	0.51
SLIT2	2|4	3	0.22	3.27	0.51
TAB2	20|17	18.5	0.21	2.96	0.51
SLC38A2	3|2	2.5	0.21	3.33	0.51
MIA3	4|0	2	0.2	2.67	0.51
SP3	3|0	1.5	0.19	3	0.52
BIRC2	23|20	21.5	0.2	3.22	0.52
RCN2	2|2	2	0.2	3.43	0.52
PABPN1	0|2	1	0.2	3	0.52

JPH1	3|3	3	0.19	3.6	0.52
WDR34	2|4	3	0.19	3.27	0.52
PLEKHA7	0|2	1	0.2	3	0.52
TOX3	0|3	1.5	0.18	2	0.53
METAP2	3|0	1.5	0.17	2.57	0.53
SDF2	2|3	2.5	0.17	3	0.53
RBM15B	0|5	2.5	0.19	2.5	0.53
NOMO1	8|5	6.5	0.18	2.89	0.53
CYP2S1	2|3	2.5	0.19	3.75	0.53
RABL3	3|3	3	0.19	3.6	0.53
PAN3	2|4	3	0.18	3.27	0.53
ZNF385A	2|0	1	0.15	3	0.54
UQCRC1	2|0	1	0.16	2.4	0.54
PREB	3|0	1.5	0.15	2.57	0.54
FOXRED1	4|3	3.5	0.17	3.23	0.54
NDUFV3	2|0	1	0.15	3	0.54
C16orf58	0|2	1	0.15	3	0.54
MRPS15	0|4	2	0.17	2.4	0.54
ALG2	0|2	1	0.15	3	0.54
TAB3	2|0	1	0.15	3	0.54
AGO1	0|3	1.5	0.14	2	0.55
ATP13A1	0|3	1.5	0.15	2	0.55
ERAP2	4|3	3.5	0.14	3	0.55
NUP85	7|8	7.5	0.14	2.9	0.55
HNRNPLL	5|3	4	0.14	2.82	0.55
CNNM3	2|2	2	0.14	3	0.55
IKBKG	0|2	1	0.12	2.4	0.56
ITPR1	2|4	3	0.14	2.57	0.56
RAB29	0|2	1	0.12	2.4	0.56
BAG4	0|2	1	0.12	2.4	0.56
PDHX	4|3	3.5	0.12	2.8	0.56
COG4	0|3	1.5	0.13	2.25	0.56
TFB1M	3|3	3	0.13	3	0.56
HSD17B7	0|3	1.5	0.13	2.25	0.56
BMP2K	4|5	4.5	0.13	2.84	0.56
FYTTD1	2|3	2.5	0.12	2.73	0.57
RAB12	3|0	1.5	0.12	2	0.57
PRPSAP1	0|3	1.5	0.12	2	0.57
ZFHX3	3|7	5	0.12	2.31	0.57
MRPL3	2|0	1	0.11	2	0.57
TMED9	6|3	4.5	0.12	2.45	0.57
IARS2	12|18	15	0.12	2.54	0.57
NDUFS7	0|2	1	0.11	2	0.57
RPSA	3|3	3	0.1	2.77	0.58
FAU	2|0	1	0.11	2	0.58

MSI1	0|4	2	0.1	1.71	0.58
LMAN1	3|3	3	0.1	2.77	0.58
PLD3	4|4	4	0.11	2.82	0.58
DDX28	0|2	1	0.1	1.71	0.58
POM121	0|6	3	0.1	1.64	0.58
MTO1	0|3	1.5	0.09	1.8	0.59
FBRSL1	17|14	15.5	0.09	2.82	0.59
HLA-C	5|7	6	0.09	2.57	0.59
ABCC1	4|3	3.5	0.09	2.62	0.59
HLA-B	2|0	1	0.09	2	0.59
ZSWIM8	3|4	3.5	0.09	2.47	0.59
MRPS12	3|0	1.5	0.09	1.64	0.59
IQGAP3	4|2	3	0.08	2.12	0.59
RHOG	5|2	3.5	0.08	2	0.6
FBN2	2|3	2.5	0.08	2.14	0.6
NDUFA6	2|3	2.5	0.08	2.5	0.6
RAB13	4|7	5.5	0.08	2.36	0.6
RAB11B	2|0	1	0.08	1.71	0.6
SPTLC1	2|4	3	0.07	2	0.6
PYCR1	2|4	3	0.08	2.25	0.6
INTS10	0|3	1.5	0.08	1.64	0.6
WDR59	0|2	1	0.08	1.71	0.6
TAP2	0|2	1	0.07	1.5	0.61
HNRNPH2	45|48	46.5	0.07	3.05	0.61
GSK3B	5|3	4	0.06	2.29	0.61
GATD3A	3|4	3.5	0.07	2.33	0.61
CLASRP	0|3	1.5	0.06	1.5	0.61
TMED5	3|0	1.5	0.07	1.5	0.61
ERGIC2	2|0	1	0.07	1.5	0.61
FAM83H	3|0	1.5	0.06	1.5	0.61
HADHA	3|4	3.5	0.06	2.33	0.62
PEX6	2|0	1	0.06	1.5	0.62
UQCRC2	34|23	28.5	0.06	2.69	0.62
SLC25A12	9|14	11.5	0.05	2.38	0.62
DROSHA	10|9	9.5	0.05	2.65	0.62
OSBPL3	2|2	2	0.06	2	0.62
CBWD1	0|3	1.5	0.06	1.38	0.62
EYA4	2|3	2.5	0.06	2.14	0.62
RBL1	0|2	1	0.06	1.33	0.62
OTUD4	3|3	3	0.05	2.12	0.63
GCAT	6|5	5.5	0.05	2.36	0.63
IKBKB	0|2	1	0.05	1.33	0.63
DCAF7	24|23	23.5	0.05	2.94	0.63
KIAA0355	4|5	4.5	0.05	2.35	0.63
AUTS2	16|17	16.5	0.05	2.87	0.63

MRPS2	3|2	2.5	0.05	2.14	0.63
CNDP2	2|2	2	0.05	2.18	0.63
ITCH	6|5	5.5	0.04	2.36	0.63
CDC42EP1	2|2	2	0.04	1.85	0.63
GRB10	3|2	2.5	0.04	2	0.64
NXT1	2|2	2	0.04	1.71	0.64
MOCS3	2|3	2.5	0.04	2	0.64
UBXN1	0|3	1.5	0.04	1.29	0.64
INO80	2|3	2.5	0.04	2	0.64
QTRT2	2|2	2	0.04	2	0.64
TAMM41	2|2	2	0.04	2	0.64
MINK1	5|0	2.5	0.04	1.25	0.64
LCLAT1	0|2	1	0.04	1.2	0.64
TSC2	2|0	1	0.04	1.09	0.65
METTL15	0|3	1.5	0.04	1.2	0.65
SP2	2|2	2	0.03	2	0.65
GPS2	3|0	1.5	0.04	1.29	0.65
AKAP9	3|4	3.5	0.04	1.91	0.65
SPCS2	2|0	1	0.04	1.2	0.65
CARS2	0|2	1	0.04	1.09	0.65
VMP1	5|3	4	0.03	2	0.65
CECR2	3|3	3	0.04	2.12	0.65
PTPMT1	0|2	1	0.04	1.09	0.65
NGDN	3|0	1.5	0.03	1.2	0.66
IFRD1	4|4	4	0.03	2.09	0.66
HIP1	2|0	1	0.03	1.09	0.66
SKI	0|2	1	0.03	1.2	0.66
SUCLG2	0|2	1	0.03	1	0.66
POLRMT	7|12	9.5	0.03	2.04	0.66
FARP2	2|0	1	0.03	1	0.66
C9orf64	2|0	1	0.03	1	0.66
HDHD5	9|7	8	0.03	2.29	0.66
ITPA	2|2	2	0.03	1.85	0.66
GPR89A	2|2	2	0.02	1.6	0.67
ORC4	2|3	2.5	0.02	1.76	0.67
RAB1A	6|0	3	0.02	1.2	0.67
BAG6	0|2	1	0.03	1	0.67
TRAM1	3|0	1.5	0.03	1.06	0.67
NEDD4L	3|2	2.5	0.02	1.67	0.67
NEPRO	2|0	1	0.03	1.09	0.67
UTP11	2|0	1	0.02	0.92	0.67
TRMU	0|2	1	0.02	1	0.67
MOB1A	0|2	1	0.02	1	0.67
SRR	3|3	3	0.02	1.8	0.67
GLT8D1	2|0	1	0.02	0.92	0.67

RPS28	2|2	2	0.02	1.71	0.68
ITPRID2	12|10	11	0.02	2.24	0.68
RAB5B	0|2	1	0.02	1	0.68
PDIA4	0|2	1	0.02	1	0.68
TMED2	5|4	4.5	0.02	2	0.68
RPL35	4|4	4	0.02	2	0.68
ZZEF1	2|0	1	0.02	0.92	0.68
RFTN1	3|6	4.5	0.02	1.8	0.68
QRICH1	3|4	3.5	0.02	1.91	0.68
PDXP	3|4	3.5	0.02	1.91	0.68
MAGT1	3|4	3.5	0.02	1.91	0.68
LMNB2	10|5	7.5	0.02	1.88	0.68
HOXD13	2|3	2.5	0.01	1.58	0.69
YAF2	3|0	1.5	0.01	1	0.69
SSR1	2|0	1	0.02	0.86	0.69
OXA1L	2|4	3	0.02	1.64	0.69
ACOT8	3|4	3.5	0.02	1.83	0.69
RER1	0|2	1	0.01	0.86	0.69
RAB14	4|5	4.5	0.02	1.93	0.69
MRPL10	3|0	1.5	0.01	0.95	0.69
RASAL2	2|4	3	0.02	1.64	0.69
RPUSD3	4|4	4	0.02	1.92	0.69
FAM111B	2|3	2.5	0.02	1.58	0.69
GK	2|0	1	0.01	0.8	0.7
FAM49B	2|0	1	0.01	0.75	0.7
TAF4	19|21	20	0.01	2.55	0.7
RRP1	0|2	1	0.01	0.75	0.7
NDUFA5	0|2	1	0.01	0.92	0.7
PRKCD	10|10	10	0.01	2.18	0.7
FKBP8	2|0	1	0.01	0.86	0.7
EWSR1	7|3	5	0.01	1.62	0.7
MYCBP2	4|0	2	0.01	1	0.7
NRDE2	0|3	1.5	0.01	0.95	0.7
AGPAT5	2|0	1	0.01	0.86	0.7
SLC25A21	2|2	2	0.01	1.5	0.7
ZNF420	2|0	1	0.01	0.8	0.7
MALT1	2|4	3	0.01	1.5	0.7
ESCO2	0|3	1.5	0.01	0.9	0.71
PLEKHG1	0|2	1	0.01	0.75	0.71
RRBP1	12|13	12.5	0.01	2.17	0.71
FBRS	7|8	7.5	0.01	2.05	0.71
SLC37A4	2|0	1	0.01	0.8	0.71
CUL7	3|3	3	0.01	1.64	0.71
ITPR2	4|0	2	0.01	0.96	0.71
HDAC3	3|0	1.5	0.01	0.86	0.71

AKAP8	14|13	13.5	0.01	2.22	0.71
CDIPT	5|2	3.5	0.01	1.45	0.71
ARFGEF2	0|2	1	0.01	0.75	0.71
NUP42	2|0	1	0.01	0.8	0.71
PFAS	17|13	15	0.01	2.09	0.71
SAMD1	3|2	2.5	0.01	1.36	0.71
DHFR	4|5	4.5	0.01	1.69	0.72
LYN	5|4	4.5	0.01	1.69	0.72
PDHA1	6|7	6.5	0.01	1.9	0.72
CDS2	2|2	2	0	1.14	0.72
PHC1	0|5	2.5	0.01	1	0.72
PCK2	18|14	16	0.01	2.16	0.72
SHMT2	20|22	21	0.01	2.55	0.72
CHTOP	6|5	5.5	0.01	1.83	0.72
NOP16	2|2	2	0.01	1.2	0.72
GSK3A	3|4	3.5	0.01	1.56	0.72
NUBPL	5|4	4.5	0	1.69	0.72
ANKRD27	3|0	1.5	0.01	0.86	0.72
TIMM29	0|2	1	0.01	0.75	0.72
DOCK11	0|3	1.5	0.01	0.82	0.72
HMGA1	2|3	2.5	0.01	1.36	0.72
VAV2	2|0	1	0	0.63	0.73
CTPS2	5|7	6	0	1.67	0.73
SATB2	7|5	6	0	1.67	0.73
ERAP1	2|0	1	0	0.6	0.73
NDUFS1	5|3	4	0	1.41	0.73
CDC20	3|3	3	0	1.38	0.73
MCM3AP	24|16	20	0	2.05	0.73
DNAJB11	4|2	3	0	1.33	0.73
NUP54	14|11	12.5	0	2	0.73
OSGEP	5|3	4	0	1.5	0.73
HOXA10	2|0	1	0	0.63	0.73
RPL36A	2|0	1	0	0.63	0.73
NAA35	0|2	1	0	0.6	0.73
COQ5	3|2	2.5	0	1.15	0.73
SKP2	3|2	2.5	0	1.3	0.73
COX15	4|3	3.5	0	1.4	0.73
SMARCAL1	0|2	1	0	0.6	0.74
AHCY	0|2	1	0	0.57	0.74
CHUK	3|2	2.5	0	1.15	0.74
ITPR3	6|11	8.5	0	1.65	0.74
VDAC1	28|31	29.5	0	2.24	0.74
HMGA2	0|2	1	0	0.6	0.74
BRSK2	0|3	1.5	0	0.72	0.74
ABCC4	2|2	2	0	1	0.74

ARFGEF1	2|2	2	0	1.04	0.74
MAGED1	5|7	6	0	1.6	0.74
MED23	0|2	1	0	0.6	0.74
CDK5RAP1	0|2	1	0	0.6	0.74
NUDT4	0|2	1	0	0.6	0.74
ALG1	0|2	1	0	0.6	0.74
MAIP1	2|3	2.5	0	1.2	0.74
SLC25A22	4|3	3.5	0	1.35	0.74
MRPL14	6|9	7.5	0	1.67	0.74
GALK1	18|15	16.5	0	2	0.75
CEP44	0|2	1	0	0.55	0.75
POLD2	6|10	8	0	1.57	0.75
P4HA2	5|4	4.5	0	1.42	0.75
PTBP3	7|5	6	0	1.57	0.75
NDUFS2	2|3	2.5	0	1.11	0.75
CSRP2	0|2	1	0	0.55	0.75
GTF3C2	0|3	1.5	0	0.6	0.75
HLA-A	5|9	7	0	1.53	0.75
FAF2	2|4	3	0	1.12	0.75
NSA2	4|7	5.5	0	1.43	0.75
DHRS7B	8|5	6.5	0	1.53	0.75
AAAS	9|6	7.5	0	1.64	0.75
RAB10	5|0	2.5	0	0.81	0.75
YTHDF1	17|13	15	0	1.94	0.75
MRPL16	4|3	3.5	0	1.31	0.75
NDC1	5|5	5	0	1.58	0.75
TMEM209	8|7	7.5	0	1.76	0.75
NNT	3|4	3.5	0	1.2	0.75
ERCC3	2|0	1	0	0.48	0.76
RPL29	2|3	2.5	0	1.03	0.76
DNM1	7|9	8	0	1.66	0.76
RCC1	2|0	1	0	0.5	0.76
SAR1A	2|0	1	0	0.5	0.76
MTF2	0|2	1	0	0.48	0.76
GCLM	9|6	7.5	0	1.55	0.76
MARCKS	2|0	1	0	0.5	0.76
SNU13	2|2	2	0	0.92	0.76
UQCRFS1	2|0	1	0	0.52	0.76
ERLIN1	0|3	1.5	0	0.62	0.76
UBXN4	3|3	3	0	1.2	0.76
VWA8	14|13	13.5	0	2.05	0.76
PYCR3	0|2	1	0	0.5	0.76
DCAKD	2|0	1	0	0.48	0.76
ARMC6	3|4	3.5	0	1.2	0.76
GNAS	4|4	4	0	1.37	0.76

MAP3K7	9|9	9	0	1.83	0.76
LUC7L	3|3	3	0	1.2	0.76
RPL12	8|5	6.5	0	1.42	0.77
CLCC1	2|3	2.5	0	0.97	0.77
POM121C	6|0	3	0	0.82	0.77
NSDHL	3|0	1.5	0	0.58	0.77
PICALM	7|0	3.5	0	0.84	0.77
SENP1	4|4	4	0	1.3	0.77
PDK1	5|4	4.5	0	1.35	0.77
STXBP3	4|4	4	0	1.23	0.77
TUBGCP4	5|4	4.5	0	1.32	0.77
CEP131	5|4	4.5	0	1.32	0.77
RAB21	5|3	4	0	1.17	0.77
NMD3	0|3	1.5	0	0.58	0.77
LRRC1	3|0	1.5	0	0.56	0.77
EXOC2	4|8	6	0	1.33	0.77
RPTOR	3|3	3	0	1.12	0.77
CERS2	0|2	1	0	0.46	0.77
SMAP2	9|9	9	0	1.74	0.77
ATAD1	3|5	4	0	1.17	0.77
TOR1AIP2	6|4	5	0	1.33	0.77
CTSB	9|7	8	0	1.6	0.77
NSUN6	2|0	1	0	0.46	0.77
SFXN4	4|3	3.5	0	1.11	0.77
UNK	2|0	1	0	0.43	0.78
CPSF4	3|6	4.5	0	1.12	0.78
EARS2	0|3	1.5	0	0.53	0.78
SRP9	3|2	2.5	0	0.94	0.78
GRAMD1A	7|6	6.5	0	1.5	0.78
VDAC2	31|26	28.5	0	2.3	0.78
MLLT10	0|2	1	0	0.44	0.78
RBM14-RBM4	10|7	8.5	0	1.57	0.78
DDX19B	6|9	7.5	0	1.43	0.78
RPS6KB1	0|2	1	0	0.46	0.78
ECH1	7|7	7	0	1.56	0.78
NUP88	21|20	20.5	0	2.07	0.78
PIK3C2A	10|7	8.5	0	1.55	0.78
RAB3B	0|5	2.5	0	0.7	0.78
SRM	0|2	1	0	0.46	0.78
DYNLL1	6|7	6.5	0	1.5	0.78
UBA3	3|0	1.5	0	0.56	0.78
MARK2	7|5	6	0	1.38	0.78
SCAMP3	3|2	2.5	0	0.94	0.78
NEFL	5|8	6.5	0	1.39	0.78
ATP5MG	4|4	4	0	1.23	0.78

SLC25A15	2|3	2.5	0	0.88	0.78
INTS11	3|0	1.5	0	0.58	0.78
HSPA12A	5|7	6	0	1.38	0.78
MICOS13	0|2	1	0	0.43	0.78
HK2	5|4	4.5	0	1.12	0.79
MEN1	3|3	3	0	0.95	0.79
RBFOX2	18|16	17	0	1.91	0.79
ALG13	5|2	3.5	0	0.95	0.79
RTCA	0|2	1	0	0.41	0.79
GSTK1	2|2	2	0	0.73	0.79
NCOA3	3|3	3	0	0.97	0.79
DTNA	2|3	2.5	0	0.83	0.79
RNF213	2|2	2	0	0.73	0.79
C19orf47	3|2	2.5	0	0.81	0.79
RING1	9|10	9.5	0	1.63	0.79
YBX3	9|6	7.5	0	1.43	0.79
VPS4B	0|3	1.5	0	0.5	0.79
BCKDK	0|2	1	0	0.38	0.79
RALY	9|5	7	0	1.31	0.79
NME7	0|2	1	0	0.39	0.79
ECT2	0|5	2.5	0	0.65	0.79
EMC3	2|2	2	0	0.71	0.79
PRR12	3|3	3	0	0.95	0.79
DDX31	3|0	1.5	0	0.5	0.79
IMP4	2|4	3	0	0.9	0.79
PSMA1	3|0	1.5	0	0.5	0.79
CSNK1E	2|0	1	0	0.39	0.79
TAB1	11|13	12	0	1.69	0.79
SFXN2	2|0	1	0	0.4	0.79
ECI2	3|3	3	0	0.95	0.79
PDHB	14|13	13.5	0	1.74	0.8
RPL34	4|2	3	0	0.86	0.8
RO60	7|4	5.5	0	1.14	0.8
ALDOA	2|3	2.5	0	0.73	0.8
HSD17B4	9|8	8.5	0	1.46	0.8
ARF6	3|4	3.5	0	0.91	0.8
GARS	3|6	4.5	0	0.98	0.8
PRPS2	9|13	11	0	1.5	0.8
SUMO1	0|2	1	0	0.38	0.8
CBX4	0|2	1	0	0.36	0.8
RAB5A	4|5	4.5	0	1.12	0.8
NCOA2	5|8	6.5	0	1.26	0.8
CKAP4	17|18	17.5	0	1.89	0.8
YWHAQ	0|2	1	0	0.36	0.8
PAXIP1	3|2	2.5	0	0.77	0.8

NUP62	13|15	14	0	1.73	0.8
DYNLRB1	0|4	2	0	0.57	0.8
SYNE2	3|3	3	0	0.88	0.8
VPS13A	0|2	1	0	0.36	0.8
MYEF2	16|16	16	0	1.81	0.8
MAP1S	4|3	3.5	0	0.91	0.8
DHX33	4|2	3	0	0.8	0.8
L2HGDH	2|2	2	0	0.65	0.8
WDCP	8|7	7.5	0	1.43	0.8
RHOT2	12|11	11.5	0	1.66	0.8
DENND6A	0|2	1	0	0.36	0.8
PTK2	8|4	6	0	1.14	0.8
ZADH2	0|2	1	0	0.38	0.8
CARM1	6|6	6	0	1.24	0.8
PLOD1	5|7	6	0	1.16	0.81
RPS12	7|6	6.5	0	1.28	0.81
PLAA	3|3	3	0	0.84	0.81
ACOT9	20|11	15.5	0	1.5	0.81
MARK3	3|6	4.5	0	0.93	0.81
TSC1	3|2	2.5	0	0.7	0.81
PGM3	10|6	8	0	1.26	0.81
CPD	4|8	6	0	1.09	0.81
CTBP1	9|11	10	0	1.46	0.81
PSMD10	2|0	1	0	0.33	0.81
YY1	9|6	7.5	0	1.23	0.81
RIPK2	0|3	1.5	0	0.42	0.81
ARHGEF1	0|2	1	0	0.32	0.81
OXSR1	2|0	1	0	0.32	0.81
MLLT6	0|3	1.5	0	0.43	0.81
RACK1	47|36	41.5	0	2.25	0.81
BASP1	0|2	1	0	0.32	0.81
KIF2C	11|12	11.5	0	1.6	0.81
STXBP2	0|2	1	0	0.31	0.81
TKFC	3|3	3	0	0.82	0.81
NDUFA13	2|3	2.5	0	0.71	0.81
MTPAP	4|5	4.5	0	1	0.81
TMEM165	2|5	3.5	0	0.82	0.81
RARS2	0|2	1	0	0.32	0.81
RAB18	2|0	1	0	0.32	0.81
EEFSEC	3|0	1.5	0	0.42	0.81
INTS3	3|3	3	0	0.82	0.81
TBL1XR1	5|4	4.5	0	0.98	0.81
GRWD1	4|4	4	0	0.94	0.81
NOM1	5|2	3.5	0	0.82	0.81
SHMT1	2|3	2.5	0	0.7	0.81

TMEM263	5|4	4.5	0	1.02	0.81
CTNNB1	2|0	1	0	0.3	0.82
FASTKD2	0|2	1	0	0.31	0.82
PDK3	3|2	2.5	0	0.67	0.82
UBAC2	0|3	1.5	0	0.39	0.82
RPS20	13|13	13	0	1.62	0.82
FMR1	5|4	4.5	0	0.93	0.82
CAMK2G	3|0	1.5	0	0.38	0.82
GLS	8|9	8.5	0	1.29	0.82
MSTO1	3|6	4.5	0	0.9	0.82
MAD2L1	3|2	2.5	0	0.68	0.82
PRPSAP2	4|4	4	0	0.94	0.82
SUCLG1	3|6	4.5	0	0.9	0.82
NDUFA10	5|6	5.5	0	1.08	0.82
SLC27A4	0|5	2.5	0	0.55	0.82
MED12	0|3	1.5	0	0.41	0.82
CAPN1	2|4	3	0	0.71	0.82
CLPX	4|8	6	0	1.01	0.82
PYCR2	6|8	7	0	1.2	0.82
PUM1	29|23	26	0	1.88	0.82
SARM1	0|4	2	0	0.46	0.82
MRPS27	2|0	1	0	0.31	0.82
SAMHD1	8|4	6	0	1.01	0.82
RPAP1	3|4	3.5	0	0.82	0.82
NBEA	0|3	1.5	0	0.39	0.82
MTFP1	0|3	1.5	0	0.39	0.82
TM9SF3	2|2	2	0	0.59	0.82
SNTB1	4|4	4	0	0.91	0.82
MRPS34	0|2	1	0	0.29	0.82
HM13	5|4	4.5	0	0.98	0.82
GFM2	2|2	2	0	0.55	0.82
CNP	4|6	5	0	1.02	0.82
PURB	2|0	1	0	0.29	0.82
CDK11A	0|2	1	0	0.29	0.82
MAP7D2	3|3	3	0	0.77	0.82
STT3B	12|10	11	0	1.45	0.82
PLEC	11|10	10.5	0	1.48	0.82
RPL24	12|10	11	0	1.42	0.83
RPS15	5|3	4	0	0.8	0.83
RPS26	3|5	4	0	0.83	0.83
PARK7	3|3	3	0	0.69	0.83
MFN2	5|7	6	0	1.04	0.83
TBC1D5	4|2	3	0	0.68	0.83
AGO2	10|6	8	0	1.16	0.83
OAT	7|5	6	0	1.01	0.83

EIF2D	4|3	3.5	0	0.76	0.83
RAB5C	3|4	3.5	0	0.78	0.83
ABCB7	9|7	8	0	1.16	0.83
DLAT	3|0	1.5	0	0.35	0.83
PTPN1	3|4	3.5	0	0.76	0.83
HIP1R	3|0	1.5	0	0.36	0.83
NDUFS3	4|0	2	0	0.44	0.83
AKT1	0|2	1	0	0.29	0.83
TUBGCP3	5|7	6	0	1.01	0.83
KIF1C	5|0	2.5	0	0.49	0.83
RNF2	5|10	7.5	0	1.05	0.83
CNOT3	2|4	3	0	0.65	0.83
TUT4	8|8	8	0	1.26	0.83
LUC7L2	6|0	3	0	0.56	0.83
TELO2	4|6	5	0	0.9	0.83
TMEM33	4|3	3.5	0	0.74	0.83
PCID2	4|3	3.5	0	0.74	0.83
NAV1	4|2	3	0	0.65	0.83
SCYL1	3|0	1.5	0	0.36	0.83
ZNF608	4|3	3.5	0	0.78	0.83
TOE1	3|3	3	0	0.72	0.83
EDC3	4|0	2	0	0.44	0.83
UBXN6	0|2	1	0	0.27	0.83
MRPS5	3|0	1.5	0	0.35	0.83
PHF6	5|8	6.5	0	1.05	0.83
DNAJB6	4|3	3.5	0	0.76	0.83
MCU	2|3	2.5	0	0.61	0.83
YME1L1	3|6	4.5	0	0.84	0.83
MAP2K7	5|5	5	0	0.97	0.83
PATL1	6|2	4	0	0.72	0.83
PPFIA1	5|5	5	0	0.98	0.83
AUP1	0|3	1.5	0	0.38	0.83
GANAB	6|7	6.5	0	1.15	0.83
RPL27A	9|8	8.5	0	1.23	0.84
RPS23	8|3	5.5	0	0.78	0.84
YARS2	17|11	14	0	1.33	0.84
HNRNPUL2	11|4	7.5	0	0.9	0.84
INTS1	4|2	3	0	0.6	0.84
TOMM40	12|11	11.5	0	1.35	0.84
CDK5	8|7	7.5	0	1.15	0.84
PEG10	2|0	1	0	0.26	0.84
CNOT2	6|7	6.5	0	1	0.84
SSR4	8|11	9.5	0	1.23	0.84
NUB1	5|3	4	0	0.73	0.84
CSNK2B	2|5	3.5	0	0.6	0.84

ELAVL1	28|34	31	0	1.83	0.84
PRPS1	10|9	9.5	0	1.23	0.84
PSMD4	6|7	6.5	0	1.05	0.84
PSMD11	3|4	3.5	0	0.72	0.84
RAB6A	5|6	5.5	0	0.89	0.84
RAF1	3|4	3.5	0	0.72	0.84
HSD17B10	17|17	17	0	1.67	0.84
CLPP	3|3	3	0	0.6	0.84
SNTB2	5|5	5	0	0.91	0.84
MORC3	3|3	3	0	0.59	0.84
FAM98A	4|0	2	0	0.39	0.84
SCFD1	14|12	13	0	1.46	0.84
DYNC1LI1	5|2	3.5	0	0.67	0.84
BIRC6	5|4	4.5	0	0.79	0.84
ANKFY1	0|3	1.5	0	0.33	0.84
SMPD4	4|3	3.5	0	0.72	0.84
CBX8	2|3	2.5	0	0.57	0.84
CAMSAP3	3|0	1.5	0	0.32	0.84
DDX55	2|5	3.5	0	0.62	0.84
MOV10	4|0	2	0	0.41	0.84
PPIA	0|4	2	0	0.39	0.84
KDM6A	4|0	2	0	0.38	0.84
CTBP2	15|16	15.5	0	1.56	0.84
WDR18	5|5	5	0	0.91	0.84
NUP210	3|3	3	0	0.65	0.84
FAR1	4|3	3.5	0	0.66	0.84
MRI1	6|7	6.5	0	1.01	0.84
FANCD2	4|5	4.5	0	0.84	0.84
PIGS	2|4	3	0	0.55	0.84
PI4KA	2|4	3	0	0.59	0.84
ELMO2	3|3	3	0	0.63	0.84
EIF2B4	5|6	5.5	0	0.94	0.84
RFC3	5|5	5	0	0.92	0.84
DLD	22|15	18.5	0	1.44	0.85
BLVRA	4|4	4	0	0.65	0.85
RPL19	10|8	9	0	1.09	0.85
GPATCH8	22|20	21	0	1.56	0.85
RPS27	9|9	9	0	1.1	0.85
ETFA	2|3	2.5	0	0.47	0.85
ALG5	6|4	5	0	0.74	0.85
DTYMK	3|0	1.5	0	0.3	0.85
EIF2B3	4|3	3.5	0	0.63	0.85
UGDH	3|3	3	0	0.57	0.85
INTS7	7|8	7.5	0	1.01	0.85
SLC12A2	2|3	2.5	0	0.49	0.85

GAPDH	5|5	5	0	0.82	0.85
HNRNPH1	107|106	106.5	0	2.69	0.85
KIF18B	2|3	2.5	0	0.48	0.85
UBE2N	5|8	6.5	0	0.87	0.85
RPL14	8|6	7	0	0.95	0.85
RHEB	6|7	6.5	0	0.88	0.85
FARSB	3|5	4	0	0.62	0.85
ACTR2	2|5	3.5	0	0.54	0.85
AASS	20|21	20.5	0	1.62	0.85
PSMD14	8|6	7	0	0.93	0.85
DNAJA2	4|3	3.5	0	0.58	0.85
TIMM44	2|5	3.5	0	0.55	0.85
TAF5	4|3	3.5	0	0.58	0.85
ERLIN2	6|5	5.5	0	0.79	0.85
SLC25A24	4|5	4.5	0	0.72	0.85
UBE3C	5|0	2.5	0	0.41	0.85
HELZ	73|70	71.5	0	2.44	0.85
MYO1D	3|0	1.5	0	0.26	0.85
NUP160	12|7	9.5	0	1.04	0.85
LARS2	4|3	3.5	0	0.61	0.85
SENP3	8|10	9	0	1.06	0.85
HDDC2	4|2	3	0	0.55	0.85
YTHDF2	8|4	6	0	0.8	0.85
WDR5	4|4	4	0	0.69	0.85
DNAJC11	3|7	5	0	0.74	0.85
DNAJC10	6|3	4.5	0	0.66	0.85
CCDC47	10|8	9	0	1.11	0.85
SRPRB	2|5	3.5	0	0.55	0.85
RPS18	9|6	7.5	0	0.98	0.85
TCHP	0|5	2.5	0	0.42	0.85
TPGS1	3|0	1.5	0	0.28	0.85
SYNRG	4|5	4.5	0	0.68	0.85
OPA1	20|16	18	0	1.45	0.85
EHD4	4|8	6	0	0.81	0.85
YWHAB	4|3	3.5	0	0.63	0.85
SPATA5	3|3	3	0	0.55	0.85
TMTC3	4|3	3.5	0	0.58	0.85
TRNT1	3|4	3.5	0	0.63	0.85
ZNF326	21|20	20.5	0	1.63	0.85
SMG7	8|5	6.5	0	0.89	0.85
RPL21	14|17	15.5	0	1.18	0.86
RPL22	12|13	12.5	0	1.11	0.86
RPL28	19|9	14	0	1.07	0.86
PKP2	10|8	9	0	1.03	0.86
IGF2BP2	31|38	34.5	0	1.67	0.86

QSER1	3|5	4	0	0.56	0.86
NCOR2	16|16	16	0	1.25	0.86
KTN1	5|9	7	0	0.77	0.86
KIF2A	8|6	7	0	0.78	0.86
CSK	10|7	8.5	0	0.88	0.86
RPS27A	9|5	7	0	0.78	0.86
MTHFD1L	22|18	20	0	1.43	0.86
TRA2B	4|2	3	0	0.47	0.86
SRSF3	10|6	8	0	0.87	0.86
HLTF	2|5	3.5	0	0.49	0.86
TUFM	106|118	112	0	2.27	0.86
EIF3F	12|8	10	0	0.96	0.86
KIF1A	5|5	5	0	0.67	0.86
ACSL3	7|8	7.5	0	0.82	0.86
YBX1	15|11	13	0	1.17	0.86
ARHGEF2	10|11	10.5	0	1.1	0.86
FXR2	14|15	14.5	0	1.29	0.86
SEC22B	3|6	4.5	0	0.61	0.86
DDOST	14|16	15	0	1.31	0.86
ALYREF	5|5	5	0	0.67	0.86
SLC25A1	9|8	8.5	0	0.88	0.86
TBCD	4|6	5	0	0.68	0.86
RPL10	15|15	15	0	1.25	0.86
H1FX	8|6	7	0	0.88	0.86
PCNT	6|9	7.5	0	0.8	0.86
HAX1	11|11	11	0	1.19	0.86
CAPZA2	2|5	3.5	0	0.43	0.86
POLE	4|5	4.5	0	0.57	0.86
RBM14	67|61	64	0	2.1	0.86
KHDRBS1	6|4	5	0	0.65	0.86
MCM5	28|19	23.5	0	1.37	0.86
SRSF1	6|6	6	0	0.74	0.86
SRP72	2|4	3	0	0.47	0.86
ABCB10	12|9	10.5	0	0.98	0.86
PKN3	4|3	3.5	0	0.5	0.86
AKAP8L	7|9	8	0	0.89	0.86
FAM120A	13|11	12	0	1.2	0.86
PPM1F	8|9	8.5	0	0.96	0.86
ZNF609	12|10	11	0	1.01	0.86
NELFB	2|5	3.5	0	0.5	0.86
ZC3HC1	8|4	6	0	0.76	0.86
TRMT10C	9|11	10	0	1.1	0.86
DDX27	19|10	14.5	0	1.04	0.86
PANK4	7|5	6	0	0.67	0.86
NCLN	13|17	15	0	1.21	0.86

TOMM22	6|6	6	0	0.79	0.86
TRMT5	6|4	5	0	0.65	0.86
PPA1	7|3	5	0	0.55	0.86
RAB1B	8|7	7.5	0	0.87	0.86
INA	8|8	8	0	0.84	0.86
LARP4	3|7	5	0	0.61	0.86
CAMK2D	3|5	4	0	0.57	0.86
IDH3B	8|8	8	0	0.91	0.86
AP3M1	6|9	7.5	0	0.89	0.86
RPL11	11|12	11.5	0	0.88	0.87
RPL23	18|13	15.5	0	1.02	0.87
RPL18A	7|11	9	0	0.75	0.87
RPL32	11|7	9	0	0.69	0.87
RPLP2	14|10	12	0	0.98	0.87
RPS6	28|19	23.5	0	1.27	0.87
TIMM50	12|11	11.5	0	0.81	0.87
PUS1	3|6	4.5	0	0.51	0.87
RPS11	20|18	19	0	1.08	0.87
SULT1A1	7|7	7	0	0.71	0.87
MYO1C	11|13	12	0	0.94	0.87
NXF1	11|9	10	0	0.76	0.87
CKAP2	3|6	4.5	0	0.52	0.87
RPL31	9|7	8	0	0.77	0.87
IMMT	13|15	14	0	0.9	0.87
SLFN11	6|8	7	0	0.66	0.87
CIZ1	33|31	32	0	1.47	0.87
P4HA1	14|16	15	0	1.11	0.87
SLC25A13	23|25	24	0	1.27	0.87
RBM15	16|17	16.5	0	1.12	0.87
KIF22	9|10	9.5	0	0.81	0.87
TUBGCP2	16|14	15	0	0.94	0.87
DDX56	5|7	6	0	0.63	0.87
DNAJA1	7|5	6	0	0.58	0.87
CANX	13|5	9	0	0.72	0.87
RBMX	11|6	8.5	0	0.63	0.87
KRT19	41|45	43	0	1.62	0.87
PFKL	14|14	14	0	1.07	0.87
MAP2K1	10|11	10.5	0	0.88	0.87
SLC16A1	8|11	9.5	0	0.68	0.87
SNX2	18|13	15.5	0	1.03	0.87
VRK1	3|7	5	0	0.5	0.87
EIF2S2	8|6	7	0	0.57	0.87
TRIP13	11|7	9	0	0.67	0.87
RPS14	26|25	25.5	0	1.46	0.87
FARP1	6|7	6.5	0	0.61	0.87

MAT2A	0|5	2.5	0	0.29	0.87
MTHFD1	159|158	158.5	0	2.6	0.87
HNRNPM	158|175	166.5	0	2.44	0.87
PA2G4	11|0	5.5	0	0.48	0.87
PCBP1	19|20	19.5	0	1.1	0.87
AFG3L2	9|9	9	0	0.77	0.87
HNRNPA0	25|24	24.5	0	1.34	0.87
AHSA1	15|13	14	0	0.91	0.87
HNRNPH3	17|22	19.5	0	1.18	0.87
RPL13A	13|11	12	0	0.88	0.87
SEC61A1	7|8	7.5	0	0.77	0.87
TNRC6A	45|44	44.5	0	1.82	0.87
UTP20	6|6	6	0	0.55	0.87
EIF4A3	46|43	44.5	0	1.7	0.87
PDXDC1	6|8	7	0	0.61	0.87
TNRC6B	40|38	39	0	1.53	0.87
UFL1	14|13	13.5	0	0.93	0.87
KIF1BP	10|7	8.5	0	0.65	0.87
PELO	7|6	6.5	0	0.7	0.87
UTP18	9|6	7.5	0	0.64	0.87
NUP98	53|48	50.5	0	1.7	0.87
LRRC40	26|24	25	0	1.36	0.87
CCAR1	9|13	11	0	0.92	0.87
COQ8A	8|4	6	0	0.59	0.87
SFXN1	27|26	26.5	0	1.4	0.87
TBRG4	9|3	6	0	0.6	0.87
CPVL	6|5	5.5	0	0.63	0.87
ATAD3B	20|21	20.5	0	1.29	0.87
RPL13	16|15	15.5	0	0.95	0.87
HK1	8|6	7	0	0.62	0.87
LMNA	25|24	24.5	0	1.39	0.87
PCNA	16|15	15.5	0	1.09	0.87
RPL9	31|32	31.5	0	1.26	0.88
ATP1A1	22|28	25	0	1.03	0.88
RPL5	20|21	20.5	0	0.93	0.88
RPL7A	23|17	20	0	0.81	0.88
RPL18	22|22	22	0	1.13	0.88
RPL26	24|22	23	0	1.07	0.88
RPS3A	24|25	24.5	0	0.94	0.88
RPS4X	32|27	29.5	0	1.15	0.88
ATP5F1C	13|16	14.5	0	0.87	0.88
RPS8	21|19	20	0	0.87	0.88
RPS9	24|23	23.5	0	1.11	0.88
KDM1A	28|20	24	0	0.99	0.88
RPS15A	20|14	17	0	0.89	0.88

RPS16	22|17	19.5	0	1.04	0.88
TUBG1	20|17	18.5	0	0.79	0.88
HNRNPF	24|25	24.5	0	1.2	0.88
DDX54	12|12	12	0	0.75	0.88
HYOU1	24|20	22	0	1.06	0.88
GSE1	25|25	25	0	0.86	0.88
RPN2	19|17	18	0	0.97	0.88
PHB2	38|37	37.5	0	1.3	0.88
SLC25A4	26|25	25.5	0	0.87	0.88
PFKM	5|8	6.5	0	0.5	0.88
ATAD3A	27|29	28	0	0.95	0.88
PGAM5	15|11	13	0	0.66	0.88
DNM2	18|13	15.5	0	0.69	0.88
NSUN2	34|29	31.5	0	0.99	0.88
C1QBP	22|18	20	0	0.81	0.88
SERPINH1	15|19	17	0	0.72	0.88
DARS	23|25	24	0	0.94	0.88
FBL	16|21	18.5	0	0.93	0.88
ATP2B1	11|10	10.5	0	0.65	0.88
HSPD1	23|27	25	0	0.91	0.88
KPNA2	16|12	14	0	0.79	0.88
MCM3	14|19	16.5	0	0.67	0.88
PFKP	15|21	18	0	0.91	0.88
SLC25A3	31|26	28.5	0	1.05	0.88
RPL15	22|18	20	0	0.84	0.88
RPN1	19|15	17	0	0.97	0.88
RPS2	22|29	25.5	0	1.03	0.88
EIF2S1	10|13	11.5	0	0.71	0.88
HNRNPC	18|18	18	0	0.84	0.88
RPS6KA3	12|14	13	0	0.79	0.88
TXNL1	21|16	18.5	0	1	0.88
LONP1	20|20	20	0	0.74	0.88
NUP214	105|93	99	0	1.79	0.88
DIAPH1	25|24	24.5	0	0.95	0.88
LMNB1	30|26	28	0	1.13	0.88
NAMPT	36|28	32	0	1.15	0.88
DDX39A	15|22	18.5	0	0.74	0.88
MCM6	30|30	30	0	1.12	0.88
TUBA1B	124|135	129.5	0	2.25	0.88
TUBB3	228|194	211	0	2.44	0.88
NSF	13|13	13	0	0.82	0.88
NPEPPS	26|26	26	0	1.26	0.88
CCT7	63|71	67	0	1.44	0.88
IGF2BP1	25|33	29	0	0.91	0.88
IGF2BP3	10|16	13	0	0.66	0.88

NUDC	21|20	20.5	0	0.72	0.88
PHGDH	27|27	27	0	1.27	0.88
DDX18	24|23	23.5	0	0.79	0.88
PDCD6IP	44|37	40.5	0	1.15	0.88
SAFB2	16|8	12	0	0.56	0.88
NUP93	26|19	22.5	0	0.79	0.88
SEC16A	15|16	15.5	0	0.9	0.88
DHX30	17|10	13.5	0	0.57	0.88
NSFL1C	6|10	8	0	0.5	0.88
DDX47	11|8	9.5	0	0.64	0.88
ARID1B	13|7	10	0	0.59	0.88
UBR4	14|10	12	0	0.67	0.88
HIST1H2AE	13|13	13	0	0.6	0.88
EPPK1	54|53	53.5	0	1.57	0.88
PCBP2	23|24	23.5	0	1.04	0.88
PTBP1	17|12	14.5	0	0.84	0.88
RPL8	15|11	13	0	0.75	0.88
ASS1	16|25	20.5	0	0.99	0.88
LRPPRC	19|18	18.5	0	0.97	0.88
ATP2A2	36|46	41	0	1.14	0.88
KRT18	34|31	32.5	0	1.3	0.88
ACAT1	6|4	5	0	0.33	0.89
BLM	3|3	3	0	0.24	0.89
EMD	10|9	9.5	0	0.72	0.89
FH	2|2	2	0	0.28	0.89
MSH6	14|13	13.5	0	0.57	0.89
MSH2	14|18	16	0	0.59	0.89
MTR	0|3	1.5	0	0.16	0.89
RB1	0|2	1	0	0.19	0.89
ALDH3A2	3|3	3	0	0.38	0.89
PCCB	94|76	85	0	0.86	0.89
TP53	21|20	20.5	0	0.5	0.89
WRN	0|3	1.5	0	0.2	0.89
POLR2A	7|9	8	0	0.53	0.89
POLR2B	8|11	9.5	0	0.57	0.89
RPL3	37|34	35.5	0	0.89	0.89
RPL4	29|25	27	0	0.77	0.89
RPL6	16|16	16	0	0.4	0.89
RPL7	23|17	20	0	0.64	0.89
RPL23A	5|4	4.5	0	0.25	0.89
RPL27	6|7	6.5	0	0.55	0.89
RPL38	6|6	6	0	0.61	0.89
RPS3	51|46	48.5	0	0.96	0.89
RPS7	10|8	9	0	0.28	0.89
ECHDC1	0|2	1	0	0.16	0.89

SMC4	17|14	15.5	0	0.42	0.89
C11orf49	0|2	1	0	0.17	0.89
KDM2B	2|3	2.5	0	0.24	0.89
RPS13	8|7	7.5	0	0.65	0.89
TFIP11	0|2	1	0	0.03	0.89
CUL4A	6|0	3	0	0.22	0.89
CKAP5	6|6	6	0	0.08	0.89
SEPTIN7	6|0	3	0	0.11	0.89
SLC3A2	2|2	2	0	0.2	0.89
HSP90AA1	33|32	32.5	0	0.69	0.89
SERBP1	4|3	3.5	0	0.09	0.89
INF2	4|0	2	0	0.27	0.89
YTHDC1	3|5	4	0	0.47	0.89
FOXK1	3|3	3	0	0.51	0.89
CYFIP2	0|3	1.5	0	0.1	0.89
USP9X	16|14	15	0	0.44	0.89
HIST2H2AA4	17|15	16	0	0.31	0.89
G6PD	2|3	2.5	0	0.43	0.89
PSPC1	11|12	11.5	0	0.48	0.89
XRN1	5|7	6	0	0.12	0.89
STAG2	0|2	1	0	0.05	0.89
TOP2A	14|15	14.5	0	0.14	0.89
TOP2B	11|12	11.5	0	0.21	0.89
TUBB2A	274|239	256.5	0	1.88	0.89
ECPAS	4|8	6	0	0.26	0.89
U2SURP	8|5	6.5	0	0.11	0.89
MGA	2|0	1	0	0.03	0.89
THOC2	2|0	1	0	0.03	0.89
WDR62	0|2	1	0	0.15	0.89
ABCF1	5|4	4.5	0	0.24	0.89
ACACB	58|42	50	0	0.79	0.89
ACLY	0|4	2	0	0.08	0.89
SMARCD2	4|2	3	0	0.37	0.89
DDX17	47|39	43	0	1.03	0.89
PDS5A	16|16	16	0	0.62	0.89
HNRNPR	7|6	6.5	0	0.26	0.89
PSMA4	2|3	2.5	0	0.48	0.89
GIGYF2	15|12	13.5	0	0.23	0.89
TASOR	4|2	3	0	0.1	0.89
FANCI	6|6	6	0	0.3	0.89
ADD1	5|4	4.5	0	0.26	0.89
BCOR	41|33	37	0	0.74	0.89
PCCA	240|202	221	0	1.45	0.89
SMARCA4	4|5	4.5	0	0.16	0.89
SRRT	2|0	1	0	0.12	0.89

EEF1D	5|4	4.5	0	0.35	0.89
KARS	5|4	4.5	0	0.32	0.89
MYO1B	2|6	4	0	0.32	0.89
SMARCC2	0|4	2	0	0.11	0.89
SPAG9	3|0	1.5	0	0.04	0.89
EIF4E	0|2	1	0	0.2	0.89
SUGT1	3|2	2.5	0	0.25	0.89
NT5DC2	3|0	1.5	0	0.21	0.89
MAP4	9|6	7.5	0	0.1	0.89
FIP1L1	3|5	4	0	0.2	0.89
TKT	0|2	1	0	0.22	0.89
NEDD1	0|3	1.5	0	0.17	0.89
AGFG1	3|4	3.5	0	0.5	0.89
TCOF1	15|8	11.5	0	0.17	0.89
EIF2AK2	4|2	3	0	0.36	0.89
PABPC4	2|3	2.5	0	0.14	0.89
YWHAZ	3|3	3	0	0.57	0.89
GART	50|54	52	0	1.01	0.89
ANXA2	0|2	1	0	0.06	0.89
CPSF7	0|3	1.5	0	0.17	0.89
KANK2	3|0	1.5	0	0.19	0.89
RCC2	4|3	3.5	0	0.26	0.89
RCOR3	3|3	3	0	0.45	0.89
AIMP1	5|5	5	0	0.48	0.89
PPHLN1	2|2	2	0	0.26	0.89
COPB1	20|26	23	0	0.74	0.89
DNAJC7	2|3	2.5	0	0.36	0.89
SIN3A	2|0	1	0	0.02	0.89
NONO	147|128	137.5	0	1.36	0.89
TTC28	5|7	6	0	0.24	0.89
SCAF4	0|2	1	0	0.15	0.89
SAE1	5|5	5	0	0.26	0.89
SLC25A5	43|43	43	0	1.02	0.89
EBNA1BP2	2|0	1	0	0.11	0.89
TNIK	4|5	4.5	0	0.22	0.89
PYGL	4|11	7.5	0	0.34	0.89
CHAMP1	4|4	4	0	0.14	0.89
ACIN1	2|2	2	0	0.03	0.89
LDHA	4|2	3	0	0.36	0.89
EPB41	4|5	4.5	0	0.08	0.89
LEMD3	5|5	5	0	0.22	0.89
ATXN10	4|4	4	0	0.59	0.89
TJP2	2|0	1	0	0.04	0.89
CHD8	5|6	5.5	0	0.11	0.89
FUS	6|3	4.5	0	0.39	0.89

NKRF	9|7	8	0	0.46	0.89
LDHB	6|5	5.5	0	0.54	0.89
ARL1	2|2	2	0	0.3	0.89
RIF1	7|7	7	0	0.13	0.89
ZBTB33	8|8	8	0	0.15	0.89
PHF8	3|5	4	0	0.42	0.89
WNK1	7|8	7.5	0	0.55	0.89
ZMYM2	9|14	11.5	0	0.38	0.89
TNPO3	3|2	2.5	0	0.34	0.89
RBBP5	2|0	1	0	0.18	0.89
DDX3X	22|14	18	0	0.39	0.89
ADAR	14|18	16	0	0.36	0.89
SRSF7	0|3	1.5	0	0.21	0.89
CLINT1	13|16	14.5	0	0.42	0.89
HSDL2	2|0	1	0	0.17	0.89
KMT2A	0|2	1	0	0.03	0.89
DPYSL2	0|3	1.5	0	0.03	0.89
DPYSL3	6|3	4.5	0	0.11	0.89
PPAN-P2RY11	2|2	2	0	0.26	0.89
ARPIN-AP3S2	2|0	1	0	0.16	0.89
EIF3C	12|0	6	0	0.21	0.89
KAT7	2|2	2	0	0.42	0.89
RPL17	8|6	7	0	0.54	0.89
PRDX1	7|5	6	0	0.41	0.89
NIPSNAP2	0|2	1	0	0.23	0.89
RPS10	0|3	1.5	0	0.21	0.89
NR3C1	3|2	2.5	0	0.45	0.89
XIAP	8|7	7.5	0	0.52	0.89
ANK3	2|0	1	0	0.06	0.89
PRKAG1	2|2	2	0	0.24	0.89
RFC5	0|3	1.5	0	0.22	0.89
AFDN	5|4	4.5	0	0.04	0.89
RNF40	0|2	1	0	0.12	0.89
PRKACB	2|0	1	0	0.16	0.89
AKAP1	3|3	3	0	0.18	0.89
EIF3L	5|6	5.5	0	0.45	0.89
PPP1CC	4|0	2	0	0.1	0.89
ERBIN	3|3	3	0	0.1	0.89
MYH10	0|6	3	0	0.15	0.89
PAF1	5|3	4	0	0.21	0.89
AURKB	5|3	4	0	0.48	0.89
CDC5L	3|3	3	0	0.07	0.89
MLH1	3|3	3	0	0.22	0.89
NOP2	6|6	6	0	0.19	0.89
CDC27	2|2	2	0	0.2	0.89

CDK9	6|5	5.5	0	0.34	0.89
CNOT1	10|13	11.5	0	0.43	0.89
PDCD6	3|4	3.5	0	0.3	0.89
TOR1AIP1	3|4	3.5	0	0.53	0.89
PPME1	3|0	1.5	0	0.21	0.89
PSMD6	4|2	3	0	0.23	0.89
QARS	2|5	3.5	0	0.12	0.89
CHD1	0|2	1	0	0.04	0.89
CHD4	12|5	8.5	0	0.05	0.89
AP3S1	3|3	3	0	0.51	0.89
CSE1L	8|10	9	0	0.23	0.89
CTNND1	3|0	1.5	0	0.12	0.89
DHX9	34|32	33	0	0.41	0.89
DHX15	15|14	14.5	0	0.37	0.89
DKC1	2|0	1	0	0.07	0.89
DYNC1H1	63|59	61	0	0.43	0.89
EEF1A1	69|73	71	0	0.66	0.89
EEF1G	13|14	13.5	0	0.69	0.89
EIF2B1	2|3	2.5	0	0.39	0.89
EIF2S3	9|11	10	0	0.74	0.89
EIF4A1	76|69	72.5	0	1.17	0.89
EIF4G2	12|10	11	0	0.49	0.89
ENO1	165|98	131.5	0	1.32	0.89
EPB41L2	8|7	7.5	0	0.14	0.89
FLNA	136|126	131	0	0.21	0.89
FLNB	12|12	12	0	0.08	0.89
XRCC6	9|9	9	0	0.27	0.89
GTF3C1	7|6	6.5	0	0.21	0.89
HDAC2	8|9	8.5	0	0.28	0.89
HNRNPL	8|9	8.5	0	0.31	0.89
IGBP1	0|2	1	0	0.11	0.89
EIF3E	2|0	1	0	0.06	0.89
IRAK1	5|5	5	0	0.38	0.89
PARP1	21|21	21	0	0.24	0.89
AHNAK	31|55	43	0	0.11	0.89
SLC25A6	31|30	30.5	0	0.8	0.89
ARCN1	12|15	13.5	0	0.54	0.89
ARF1	12|10	11	0	0.52	0.89
ARF4	4|5	4.5	0	0.33	0.89
ARL2	4|4	4	0	0.6	0.89
ATP5F1B	80|77	78.5	0	1.29	0.89
ATP6V1B2	2|2	2	0	0.28	0.89
CCT6A	83|73	78	0	1.32	0.89
CDK1	30|26	28	0	0.84	0.89
CKB	0|4	2	0	0.29	0.89

CNN3	2|3	2.5	0	0.15	0.89
CSNK2A2	6|5	5.5	0	0.4	0.89
CTPS1	16|18	17	0	0.56	0.89
DSG2	0|3	1.5	0	0.09	0.89
EEF2	5|5	5	0	0.11	0.89
FKBP3	2|0	1	0	0.19	0.89
GFPT1	5|4	4.5	0	0.47	0.89
H2AFZ	11|9	10	0	0.36	0.89
HTT	3|4	3.5	0	0.53	0.89
HNRNPD	5|8	6.5	0	0.44	0.89
HSPA4	3|4	3.5	0	0.21	0.89
KIFC1	6|5	5.5	0	0.37	0.89
KPNA1	3|3	3	0	0.33	0.89
KPNB1	10|5	7.5	0	0.31	0.89
TNPO1	0|2	1	0	0.1	0.89
IPO5	6|7	6.5	0	0.23	0.89
LBR	13|16	14.5	0	0.45	0.89
MKI67	23|11	17	0	0.07	0.89
MSH3	2|0	1	0	0.06	0.89
MTX1	2|2	2	0	0.52	0.89
MYH9	16|16	16	0	0.12	0.89
NASP	2|2	2	0	0.03	0.89
NBN	5|5	5	0	0.3	0.89
NPM1	15|12	13.5	0	0.16	0.89
NVL	7|5	6	0	0.5	0.89
ORC5	2|2	2	0	0.32	0.89
PABPC1	5|5	5	0	0.19	0.89
PHB	15|12	13.5	0	0.52	0.89
PIK3C3	2|0	1	0	0.13	0.89
PKM	34|28	31	0	0.6	0.89
EXOSC10	6|5	5.5	0	0.14	0.89
POLD1	15|14	14.5	0	0.52	0.89
POLR2E	4|3	3.5	0	0.5	0.89
PPP1CA	3|2	2.5	0	0.15	0.89
PPP1CB	0|2	1	0	0.07	0.89
PKN1	0|2	1	0	0.22	0.89
MAP2K3	2|2	2	0	0.33	0.89
PSMC1	10|6	8	0	0.44	0.89
PSMC2	9|9	9	0	0.44	0.89
PSMC3	5|5	5	0	0.37	0.89
PSMC5	5|8	6.5	0	0.37	0.89
PSMC6	0|3	1.5	0	0.15	0.89
PSMD1	3|5	4	0	0.37	0.89
PSMD2	15|11	13	0	0.47	0.89
PSMD3	28|26	27	0	0.83	0.89

PSMD8	2|0	1	0	0.15	0.89
PSMD12	4|0	2	0	0.27	0.89
PTPN12	0|3	1.5	0	0.28	0.89
ABCD3	8|11	9.5	0	0.57	0.89
ALDH18A1	58|50	54	0	1.1	0.89
PYGB	3|2	2.5	0	0.34	0.89
RANGAP1	3|3	3	0	0.07	0.89
RARS	15|17	16	0	0.48	0.89
RBBP7	8|5	6.5	0	0.3	0.89
NELFE	0|2	1	0	0.05	0.89
UPF1	6|3	4.5	0	0.37	0.89
RFC1	0|10	5	0	0.23	0.89
RFX1	2|2	2	0	0.12	0.89
RPA2	2|0	1	0	0.12	0.89
RPS6KA1	8|8	8	0	0.62	0.89
SAFB	10|8	9	0	0.47	0.89
ATXN2	8|8	8	0	0.64	0.89
SET	6|7	6.5	0	0.62	0.89
SMARCB1	2|2	2	0	0.38	0.89
SMARCC1	3|0	1.5	0	0.18	0.89
SMARCD1	2|2	2	0	0.46	0.89
SMARCE1	2|3	2.5	0	0.19	0.89
SNRNP70	2|0	1	0	0.05	0.89
SNRPA1	2|5	3.5	0	0.11	0.89
SNX1	4|3	3.5	0	0.16	0.89
SRP14	2|2	2	0	0.46	0.89
SRPK1	3|0	1.5	0	0.15	0.89
SRPRA	7|11	9	0	0.5	0.89
SSB	0|3	1.5	0	0.09	0.89
SSRP1	5|4	4.5	0	0.18	0.89
STAT3	3|3	3	0	0.14	0.89
STIM1	4|4	4	0	0.51	0.89
TFRC	2|2	2	0	0.42	0.89
TMPO	24|23	23.5	0	0.39	0.89
TOP1	4|4	4	0	0.18	0.89
TPR	23|28	25.5	0	0.16	0.89
NR2C2	0|4	2	0	0.23	0.89
HSP90B1	19|22	20.5	0	0.66	0.89
TTK	4|0	2	0	0.2	0.89
UBA1	0|2	1	0	0.08	0.89
VIM	161|152	156.5	0	1.34	0.89
XPO1	14|14	14	0	0.51	0.89
DEK	2|2	2	0	0.34	0.89
KMT2D	3|3	3	0	0.28	0.89
SLC7A5	2|0	1	0	0.19	0.89

SLC25A11	11|10	10.5	0	0.52	0.89
TAGLN2	7|7	7	0	0.27	0.89
CUL4B	6|5	5.5	0	0.25	0.89
CUL1	3|4	3.5	0	0.4	0.89
TTF2	4|2	3	0	0.23	0.89
SMARCA5	24|21	22.5	0	0.32	0.89
IRS4	31|29	30	0	0.45	0.89
AP3B1	0|2	1	0	0.04	0.89
YARS	10|15	12.5	0	0.44	0.89
KHSRP	20|21	20.5	0	0.58	0.89
RUVBL1	12|9	10.5	0	0.16	0.89
USO1	7|4	5.5	0	0.46	0.89
IRS2	3|3	3	0	0.52	0.89
EIF3A	6|7	6.5	0	0.11	0.89
EIF3B	3|4	3.5	0	0.11	0.89
EIF3D	5|5	5	0	0.27	0.89
EIF3G	5|4	4.5	0	0.36	0.89
EIF3I	6|6	6	0	0.25	0.89
EED	0|2	1	0	0.21	0.89
SUCLA2	3|3	3	0	0.41	0.89
DPM1	6|6	6	0	0.66	0.89
IQGAP1	8|8	8	0	0.29	0.89
GMPS	0|3	1.5	0	0.06	0.89
ALDH1A2	3|0	1.5	0	0.07	0.89
NAE1	2|2	2	0	0.32	0.89
PRPF4B	4|0	2	0	0.11	0.89
FUBP3	8|8	8	0	0.43	0.89
AP3D1	5|4	4.5	0	0.2	0.89
ATP5F1A	148|148	148	0	1.72	0.89
BYSL	5|4	4.5	0	0.45	0.89
AP2M1	0|2	1	0	0.14	0.89
DCTN1	30|25	27.5	0	0.75	0.89
FASN	87|71	79	0	0.17	0.89
FEN1	3|3	3	0	0.22	0.89
HSPA9	56|56	56	0	0.97	0.89
LLGL1	2|2	2	0	0.24	0.89
WARS	14|9	11.5	0	0.37	0.89
TRIP12	3|4	3.5	0	0.28	0.89
EFTUD2	6|3	4.5	0	0.11	0.89
BAG3	3|2	2.5	0	0.2	0.89
BAG2	0|4	2	0	0.21	0.89
GTPBP1	0|5	2.5	0	0.21	0.89
CAD	33|28	30.5	0	0.62	0.89
COPA	4|5	4.5	0	0.19	0.89
DDX5	54|35	44.5	0	0.97	0.89

DDX6	6|4	5	0	0.29	0.89
DDX10	5|6	5.5	0	0.13	0.89
SEPTIN2	3|4	3.5	0	0.14	0.89
DSP	27|23	25	0	0.62	0.89
DVL2	13|17	15	0	0.45	0.89
DVL3	10|6	8	0	0.36	0.89
EPRS	50|59	54.5	0	0.65	0.89
FARSA	6|4	5	0	0.43	0.89
FLOT2	4|4	4	0	0.64	0.89
ILF2	8|7	7.5	0	0.26	0.89
KIF5B	3|2	2.5	0	0.05	0.89
KIF11	6|12	9	0	0.41	0.89
MCM2	7|8	7.5	0	0.31	0.89
SFSWAP	3|3	3	0	0.21	0.89
STAU1	8|8	8	0	0.48	0.89
COIL	0|6	3	0	0.22	0.89
MTA1	6|4	5	0	0.08	0.89
BUB3	2|3	2.5	0	0.26	0.89
DDX21	51|48	49.5	0	0.61	0.89
ETF1	7|8	7.5	0	0.51	0.89
NOLC1	6|5	5.5	0	0.28	0.89
RPS6KA5	3|2	2.5	0	0.39	0.89
MED1	2|0	1	0	0.03	0.89
SYMPK	2|0	1	0	0.07	0.89
EIF4E2	3|0	1.5	0	0.09	0.89
CLTC	4|3	3.5	0	0.1	0.89
H2AFY	8|3	5.5	0	0.19	0.89
PRDX6	8|8	8	0	0.48	0.89
CAPZB	5|4	4.5	0	0.36	0.89
DDX1	9|6	7.5	0	0.28	0.89
HDAC1	12|10	11	0	0.39	0.89
MARS	19|23	21	0	0.67	0.89
MYO6	3|3	3	0	0.12	0.89
PWP2	3|0	1.5	0	0.26	0.89
SFPQ	58|55	56.5	0	0.91	0.89
TLE1	2|0	1	0	0.2	0.89
TLE3	3|3	3	0	0.24	0.89
TRIM25	0|2	1	0	0.07	0.89
FXR1	3|3	3	0	0.25	0.89
ZMYM4	4|7	5.5	0	0.34	0.89
AKAP12	3|0	1.5	0	0.05	0.89
THRAP3	7|4	5.5	0	0.11	0.89
NUP153	38|34	36	0	0.6	0.89
SART1	0|3	1.5	0	0.04	0.89
USP14	3|0	1.5	0	0.11	0.89

USP10	0|2	1	0	0.11	0.89
CRKL	0|2	1	0	0.03	0.89
CTTN	15|8	11.5	0	0.18	0.89
GNL1	0|2	1	0	0.16	0.89
HCFC1	16|13	14.5	0	0.25	0.89
HSPA1B	103|102	102.5	0	0.52	0.89
HSPA5	33|37	35	0	0.52	0.89
NCL	13|10	11.5	0	0.14	0.89
SALL2	0|2	1	0	0.19	0.89
MLF2	2|0	1	0	0.18	0.89
CHAF1B	4|2	3	0	0.4	0.89
SMC3	30|33	31.5	0	0.65	0.89
HDAC5	4|3	3.5	0	0.34	0.89
UBA2	2|0	1	0	0.05	0.89
CFL1	2|2	2	0	0.18	0.89
MRE11	10|8	9	0	0.11	0.89
SLC1A5	4|3	3.5	0	0.49	0.89
ABCF2	3|5	4	0	0.23	0.89
RAD50	14|19	16.5	0	0.49	0.89
PDIA6	0|2	1	0	0.19	0.89
CEBPZ	4|4	4	0	0.21	0.89
TRIM28	6|6	6	0	0.14	0.89
RCL1	2|2	2	0	0.32	0.89
PURA	4|2	3	0	0.43	0.89
SAP18	6|0	3	0	0.18	0.89
SF3A1	5|2	3.5	0	0.09	0.89
PAK4	8|8	8	0	0.61	0.89
MAP1B	18|17	17.5	0	0.16	0.89
MCM7	48|45	46.5	0	0.94	0.89
CCT3	76|90	83	0	1.39	0.89
ARID1A	9|10	9.5	0	0.25	0.89
CDC42BPB	2|2	2	0	0.5	0.89
TUBB4A	228|201	214.5	0	1.65	0.89
TUBB4B	258|239	248.5	0	1.61	0.89
SCML2	5|3	4	0	0.08	0.89
CAPZA1	0|6	3	0	0.22	0.89
NUMA1	25|20	22.5	0	0.22	0.89
PCM1	13|11	12	0	0.26	0.89
PKN2	2|6	4	0	0.24	0.89
RAD21	3|3	3	0	0.14	0.89
RANBP2	63|53	58	0	0.39	0.89
SRSF6	4|3	3.5	0	0.51	0.89
STK3	0|2	1	0	0.15	0.89
TACC1	6|6	6	0	0.28	0.89
TLN1	6|4	5	0	0.06	0.89

VARS	18|9	13.5	0	0.49	0.89
XRCC1	2|0	1	0	0.08	0.89
AIMP2	4|4	4	0	0.38	0.89
SMC1A	42|45	43.5	0	0.85	0.89
NCOR1	15|20	17.5	0	0.6	0.89
PGRMC2	4|4	4	0	0.52	0.89
SEC24B	2|3	2.5	0	0.31	0.89
RAN	27|28	27.5	0	0.76	0.89
LRRC41	6|6	6	0	0.62	0.89
SYNCRIP	6|4	5	0	0.43	0.89
CHERP	7|2	4.5	0	0.13	0.89
IPO7	0|3	1.5	0	0.14	0.89
NOP56	7|12	9.5	0	0.29	0.89
ARL6IP5	2|2	2	0	0.6	0.89
CCT4	86|79	82.5	0	1.44	0.89
CCT2	67|70	68.5	0	0.97	0.89
SMC2	18|20	19	0	0.57	0.89
PRPF8	6|5	5.5	0	0.06	0.89
TBL3	9|7	8	0	0.57	0.89
PSMC4	6|4	5	0	0.26	0.89
YKT6	2|2	2	0	0.32	0.89
CCT8	80|79	79.5	0	0.73	0.89
POLD3	4|3	3.5	0	0.46	0.89
HSPA8	55|60	57.5	0	0.46	0.89
SEPTIN9	5|5	5	0	0.17	0.89
SRCAP	3|0	1.5	0	0.21	0.89
RUVBL2	8|8	8	0	0.13	0.89
TCERG1	13|7	10	0	0.21	0.89
WDR3	3|5	4	0	0.19	0.89
GCN1	24|28	26	0	0.25	0.89
SF3B2	7|5	6	0	0.1	0.89
ILVBL	4|6	5	0	0.44	0.89
PRKDC	158|139	148.5	0	0.87	0.89
SKIV2L	3|3	3	0	0.22	0.89
NUDT21	3|3	3	0	0.1	0.89
POLR3A	0|3	1.5	0	0.2	0.89
RPL10A	7|5	6	0	0.42	0.89
UTRN	6|6	6	0	0.11	0.89
VCP	3|4	3.5	0	0.15	0.89
STRAP	6|3	4.5	0	0.19	0.89
SEC23IP	0|3	1.5	0	0.11	0.89
SUPT16H	4|5	4.5	0	0.09	0.89
DDX20	2|3	2.5	0	0.23	0.89
SEC63	7|6	6.5	0	0.54	0.89
XPOT	4|5	4.5	0	0.39	0.89

ATXN2L	19|15	17	0	0.28	0.89
CBX3	2|2	2	0	0.15	0.89
STAT1	8|11	9.5	0	0.41	0.89
HSP90AB1	48|45	46.5	0	0.6	0.89
TARDBP	6|6	6	0	0.65	0.89
DNM1L	11|10	10.5	0	0.37	0.89
CCT5	68|67	67.5	0	1.14	0.89
GTF3C5	3|3	3	0	0.18	0.89
MACF1	6|6	6	0	0.27	0.89
CD2AP	2|2	2	0	0.02	0.89
CHORDC1	2|2	2	0	0.22	0.89
COPG2	11|14	12.5	0	0.17	0.89
SLC25A10	7|5	6	0	0.54	0.89
RABGAP1	2|0	1	0	0.2	0.89
GTF3C4	4|5	4.5	0	0.31	0.89
XRN2	9|12	10.5	0	0.32	0.89
ESPL1	2|0	1	0	0.25	0.89
G3BP2	0|2	1	0	0.18	0.89
EPB41L3	18|17	17.5	0	0.23	0.89
KIF4A	5|5	5	0	0.14	0.89
GTPBP4	10|8	9	0	0.44	0.89
PFDN2	2|0	1	0	0.14	0.89
RAB3GAP2	5|4	4.5	0	0.29	0.89
SF3B3	7|10	8.5	0	0.16	0.89
SF3B1	21|29	25	0	0.25	0.89
PRPF6	14|12	13	0	0.55	0.89
UCK2	0|2	1	0	0.16	0.89
ZNF281	4|6	5	0	0.29	0.89
TBK1	3|4	3.5	0	0.41	0.89
VPS51	0|2	1	0	0.19	0.89
UHRF1	2|0	1	0	0.16	0.89
GNL2	4|3	3.5	0	0.1	0.89
CPSF1	0|3	1.5	0	0.05	0.89
PRRC2B	15|15	15	0	0.65	0.89
STRN4	2|2	2	0	0.3	0.89
IARS	35|33	34	0	0.71	0.89
STOML2	3|0	1.5	0	0.14	0.89
BAZ1A	4|4	4	0	0.14	0.89
BAZ2A	0|4	2	0	0.05	0.89
LIG3	11|10	10.5	0	0.45	0.89
SNRNP200	5|4	4.5	0	0.06	0.89
MRTFB	21|18	19.5	0	0.24	0.89
NCOA6	0|2	1	0	0.08	0.89
SRP68	6|9	7.5	0	0.22	0.89
MAPRE2	4|5	4.5	0	0.26	0.89

PES1	7|4	5.5	0	0.28	0.89
RTCB	3|5	4	0	0.27	0.89
EDC4	3|4	3.5	0	0.16	0.89
GNL3	6|9	7.5	0	0.36	0.89
SND1	6|6	6	0	0.18	0.89
CACYBP	5|5	5	0	0.5	0.89
DIMT1	2|4	3	0	0.43	0.89
ZNF638	7|9	8	0	0.18	0.89
UBE2S	0|2	1	0	0.22	0.89
PRPF19	10|8	9	0	0.28	0.89
MYBBP1A	42|32	37	0	0.53	0.89
DNTTIP2	0|2	1	0	0.06	0.89
MAGED2	10|8	9	0	0.52	0.89
PIK3R4	3|0	1.5	0	0.29	0.89
CYFIP1	4|0	2	0	0.13	0.89
MDN1	3|5	4	0	0.21	0.89
ZNF592	0|2	1	0	0.15	0.89
TTC37	4|4	4	0	0.38	0.89
MDC1	0|3	1.5	0	0.01	0.89
AQR	4|3	3.5	0	0.43	0.89
SART3	4|3	3.5	0	0.06	0.89
KNTC1	0|2	1	0	0.11	0.89
SETD1A	0|4	2	0	0.16	0.89
SLK	4|4	4	0	0.08	0.89
BCLAF1	5|6	5.5	0	0.1	0.89
CEP170	5|3	4	0	0.05	0.89
ZC3H11A	2|2	2	0	0.07	0.89
TOX4	5|5	5	0	0.13	0.89
DDX46	12|10	11	0	0.1	0.89
UBAP2L	76|77	76.5	0	1.34	0.89
NCAPD2	2|3	2.5	0	0.18	0.89
SUGP2	18|14	16	0	0.32	0.89
PDAP1	0|2	1	0	0.09	0.89
MORC2	2|4	3	0	0.21	0.89
DIS3	5|5	5	0	0.27	0.89
PDCD11	13|16	14.5	0	0.45	0.89
SPEN	6|8	7	0	0.1	0.89
ZNF292	2|0	1	0	0.11	0.89
XPO7	2|0	1	0	0.1	0.89
PDS5B	14|8	11	0	0.48	0.89
WAPL	5|3	4	0	0.13	0.89
EMC1	4|4	4	0	0.6	0.89
UHRF1BP1L	0|2	1	0	0.23	0.89
RRP1B	6|0	3	0	0.16	0.89
TBC1D2B	2|0	1	0	0.19	0.89

SMG1	0|2	1	0	0.2	0.89
CLASP2	0|2	1	0	0.09	0.89
ANKLE2	3|3	3	0	0.51	0.89
ALMS1	5|0	2.5	0	0.09	0.89
NUP205	10|10	10	0	0.43	0.89
PHF3	17|16	16.5	0	0.51	0.89
RCOR1	4|4	4	0	0.4	0.89
PRRC2C	35|22	28.5	0	0.4	0.89
RRP12	6|6	6	0	0.39	0.89
FKBP15	0|3	1.5	0	0.26	0.89
ESYT1	3|5	4	0	0.12	0.89
SMCHD1	6|8	7	0	0.16	0.89
HAUS5	3|0	1.5	0	0.19	0.89
USP24	4|4	4	0	0.4	0.89
LARP1	8|5	6.5	0	0.09	0.89
RRP8	2|2	2	0	0.5	0.89
NUDCD3	0|2	1	0	0.21	0.89
ADNP	15|13	14	0	0.32	0.89
NCAPH	7|6	6.5	0	0.37	0.89
NUP188	0|4	2	0	0.15	0.89
MTREX	5|7	6	0	0.23	0.89
EP400	2|3	2.5	0	0.12	0.89
RPL36	2|0	1	0	0.23	0.89
AASDHPPT	0|2	1	0	0.16	0.89
POLR1A	0|2	1	0	0.13	0.89
AHCTF1	4|2	3	0	0.05	0.89
CAMSAP1	0|3	1.5	0	0.06	0.89
DPCD	0|2	1	0	0.26	0.89
LARP7	2|4	3	0	0.32	0.89
GEMIN5	6|5	5.5	0	0.09	0.89
PTPN23	0|2	1	0	0.09	0.89
PRPF31	5|6	5.5	0	0.45	0.89
GAPVD1	5|4	4.5	0	0.16	0.89
NOC2L	7|9	8	0	0.5	0.89
RSL1D1	7|5	6	0	0.2	0.89
GEMIN4	0|4	2	0	0.29	0.89
PCF11	7|3	5	0	0.32	0.89
UBR5	4|6	5	0	0.38	0.89
EIF5B	0|2	1	0	0.04	0.89
TRIM33	0|2	1	0	0.11	0.89
NOP58	11|9	10	0	0.54	0.89
TAF9B	3|3	3	0	0.4	0.89
RTRAF	2|2	2	0	0.15	0.89
ZFR	12|11	11.5	0	0.32	0.89
COPG1	11|11	11	0	0.56	0.89

HSD17B12	10|9	9.5	0	0.67	0.89
MRTO4	6|6	6	0	0.47	0.89
CPSF3	2|4	3	0	0.16	0.89
TRIP4	2|2	2	0	0.45	0.89
DDX41	3|3	3	0	0.18	0.89
HP1BP3	0|5	2.5	0	0.12	0.89
TRAP1	39|42	40.5	0	0.75	0.89
SRRM2	14|10	12	0	0.25	0.89
RAPGEF6	2|3	2.5	0	0.16	0.89
LIMA1	10|7	8.5	0	0.21	0.89
WWOX	6|6	6	0	0.35	0.89
HACD3	9|8	8.5	0	0.59	0.89
LUC7L3	3|3	3	0	0.41	0.89
CDK12	6|0	3	0	0.14	0.89
STK26	3|3	3	0	0.37	0.89
KDM3B	0|5	2.5	0	0.05	0.89
PAXBP1	2|0	1	0	0.06	0.89
CRK	0|3	1.5	0	0.04	0.89
POLA1	5|5	5	0	0.19	0.89
SMN2	3|3	3	0	0.49	0.89
CPSF2	2|0	1	0	0.06	0.89
ZCCHC8	4|2	3	0	0.14	0.89
ILF3	11|8	9.5	0	0.46	0.89
CDKN2AIP	3|4	3.5	0	0.55	0.89
CARMIL1	2|2	2	0	0.31	0.89
HAUS6	2|0	1	0	0.03	0.89
FTSJ3	12|12	12	0	0.38	0.89
TEX10	4|3	3.5	0	0.32	0.89
CDKAL1	2|2	2	0	0.57	0.89
TASOR2	0|3	1.5	0	0.12	0.89
DNAAF5	4|4	4	0	0.42	0.89
PRPF40A	0|4	2	0	0.11	0.89
SCYL2	2|3	2.5	0	0.33	0.89
WDR6	2|0	1	0	0.24	0.89
ATG2B	4|4	4	0	0.06	0.89
HEATR1	4|4	4	0	0.22	0.89
RBM28	7|4	5.5	0	0.28	0.89
LARP1B	2|0	1	0	0.09	0.89
IPO9	4|3	3.5	0	0.33	0.89
WDR11	3|0	1.5	0	0.08	0.89
ELAC2	2|2	2	0	0.29	0.89
RPRD1A	0|2	1	0	0.18	0.89
SBNO1	0|2	1	0	0.09	0.89
ARFGAP1	2|0	1	0	0.05	0.89
NUP133	4|0	2	0	0.08	0.89

BRIX1	5|0	2.5	0	0.16	0.89
ABCF3	2|0	1	0	0.16	0.89
WDR33	5|6	5.5	0	0.17	0.89
UTP6	4|0	2	0	0.27	0.89
CAND1	27|30	28.5	0	0.56	0.89
UBAP2	8|4	6	0	0.36	0.89
UNC45A	5|9	7	0	0.38	0.89
COG1	2|2	2	0	0.33	0.89
MATR3	21|20	20.5	0	0.48	0.89
DHX29	5|6	5.5	0	0.22	0.89
DMAP1	0|3	1.5	0	0.31	0.89
SMG9	0|2	1	0	0.25	0.89
YLPM1	45|36	40.5	0	0.55	0.89
MEPCE	3|4	3.5	0	0.27	0.89
PARD3	5|2	3.5	0	0.35	0.89
EIF4ENIF1	2|6	4	0	0.32	0.89
LARS	40|40	40	0	0.6	0.89
ZC3HAV1	8|5	6.5	0	0.25	0.89
DPYSL5	2|5	3.5	0	0.12	0.89
WRNIP1	0|3	1.5	0	0.07	0.89
PNO1	2|2	2	0	0.5	0.89
MCCC1	189|183	186	0	1.72	0.89
XAB2	0|2	1	0	0.03	0.89
PCNP	2|4	3	0	0.27	0.89
NUP107	2|3	2.5	0	0.19	0.89
DDX24	6|4	5	0	0.25	0.89
CORO1B	5|5	5	0	0.09	0.89
ANKHD1-EIF4EBP3	29|25	27	0	0.78	0.89
GATAD2B	4|5	4.5	0	0.16	0.89
ESYT2	3|8	5.5	0	0.31	0.89
MTA3	2|0	1	0	0.06	0.89
XPO5	5|5	5	0	0.21	0.89
NUFIP2	7|6	6.5	0	0.22	0.89
ZNF687	5|2	3.5	0	0.35	0.89
OSBPL8	6|4	5	0	0.34	0.89
CIP2A	3|0	1.5	0	0.09	0.89
HIST1H3F	10|7	8.5	0	0.54	0.89
MYL6	3|5	4	0	0.37	0.89
HIST1H2BB	24|21	22.5	0	0.23	0.89
HIST1H2BD	27|23	25	0	0.25	0.89
NMT1	2|0	1	0	0.12	0.89
XRCC5	5|3	4	0	0.12	0.89
CCAR2	27|24	25.5	0	0.77	0.89
RPRD1B	4|4	4	0	0.44	0.89
RBM25	9|4	6.5	0	0.23	0.89

EXOC4	5|4	4.5	0	0.18	0.89
DHX35	2|0	1	0	0.15	0.89
RIC8A	2|3	2.5	0	0.45	0.89
HIST1H4J	24|20	22	0	0.3	0.89
SEC24A	3|4	3.5	0	0.54	0.89
JUP	4|2	3	0	0.29	0.89
CHTF18	2|2	2	0	0.48	0.89
MCCC2	158|138	148	0	1.43	0.89
PC	261|228	244.5	0	1.01	0.89
NCAPG	2|4	3	0	0.23	0.89
MMS19	0|3	1.5	0	0.18	0.89
NOC3L	6|6	6	0	0.28	0.89
ANAPC1	3|3	3	0	0.12	0.89
ACBD3	2|2	2	0	0.29	0.89
SNRPN	6|6	6	0	0.62	0.89
NOL6	4|2	3	0	0.28	0.89
KRI1	3|3	3	0	0.27	0.89
DDX50	17|13	15	0	0.57	0.89
EFHD2	6|5	5.5	0	0.27	0.89
HAUS3	2|0	1	0	0.13	0.89
RPAP3	2|2	2	0	0.08	0.89
NOL9	3|6	4.5	0	0.39	0.89
IPO4	0|4	2	0	0.12	0.89
NAT10	16|17	16.5	0	0.32	0.89
ATAD5	0|2	1	0	0.08	0.89
NOL10	2|2	2	0	0.18	0.89
PHC3	0|2	1	0	0.15	0.89
VCPIP1	4|5	4.5	0	0.1	0.89
CHD9	2|0	1	0	0.18	0.89
WDR82	3|0	1.5	0	0.06	0.89
MAP2K2	7|6	6.5	0	0.57	0.89
RAI1	3|0	1.5	0	0.07	0.89
TCP1	112|108	110	0	1.51	0.89
ILKAP	0|2	1	0	0.23	0.89
CLPTM1L	0|3	1.5	0	0.24	0.89
TRMT1L	3|6	4.5	0	0.28	0.89
HNRNPA1	24|18	21	0	0.61	0.89
LAS1L	3|6	4.5	0	0.38	0.89
HNRNPA2B1	38|28	33	0	0.77	0.89
HNRNPK	21|11	16	0	0.28	0.89
HNRNPAB	4|4	4	0	0.35	0.89
HNRNPDL	0|2	1	0	0.16	0.89
HUWE1	13|15	14	0	0.34	0.89
HNRNPU	65|61	63	0	0.9	0.89
EIF2A	0|2	1	0	0.08	0.89

BRIP1	0|3	1.5	0	0.08	0.89
CEP192	0|2	1	0	0.14	0.89
ANKRD17	22|24	23	0	0.57	0.89
POLDIP3	3|2	2.5	0	0.22	0.89
NTPCR	3|2	2.5	0	0.44	0.89
NIFK	3|0	1.5	0	0.23	0.89
BAZ1B	7|9	8	0	0.11	0.89
ZNF512	6|6	6	0	0.52	0.89
TUBB6	131|121	126	0	1.68	0.89
DHX37	4|2	3	0	0.32	0.89
TUBA1C	114|119	116.5	0	0.78	0.89
JMJD1C	14|11	12.5	0	0.2	0.89
SEC23B	4|0	2	0	0.18	0.89
GTF2I	7|7	7	0	0.15	0.89
RPS24	7|9	8	0	0.74	0.89
NEK9	0|3	1.5	0	0.08	0.89
ZNF622	0|2	1	0	0.16	0.89
LUZP1	0|2	1	0	0.04	0.89
CDK2	11|9	10	0	0.65	0.89
LENG8	6|5	5.5	0	0.28	0.89
RPLP0	10|9	9.5	0	0.63	0.89
BCCIP	0|2	1	0	0.18	0.89
RNPS1	3|4	3.5	0	0.24	0.89
DDX39B	12|18	15	0	0.58	0.89
PRRC2A	7|9	8	0	0.16	0.89
NIPBL	6|4	5	0	0.36	0.89
ASNS	7|6	6.5	0	0.35	0.89
RAVER1	2|2	2	0	0.15	0.89
ATRX	0|3	1.5	0	0.03	0.89
TECR	5|5	5	0	0.52	0.89
KIF23	4|5	4.5	0	0.2	0.89
SON	4|5	4.5	0	0.14	0.89
VANGL1	2|0	1	0	0.23	0.89
SMARCA1	12|13	12.5	0	0.35	0.89
NAP1L1	3|3	3	0	0.16	0.89
TAF6	3|3	3	0	0.28	0.89
JPT2	0|2	1	0	0.09	0.89
ZC3H18	3|3	3	0	0.2	0.89
HNRNPUL1	4|3	3.5	0	0.27	0.89
UBLCP1	0|2	1	0	0.18	0.89
MAP4K4	8|5	6.5	0	0.5	0.89
AP1B1	0|3	1.5	0	0.23	0.89
POGZ	13|15	14	0	0.5	0.89
AIFM1	8|6	7	0	0.36	0.89
TARS	3|2	2.5	0	0.21	0.89

LSM12	0|3	1.5	0	0.23	0.89
TES	3|3	3	0	0.3	0.89
LRWD1	4|4	4	0	0.15	0.89
NUP155	6|5	5.5	0	0.61	0.89
SKP1	2|0	1	0	0.16	0.89
TJP1	3|6	4.5	0	0.09	0.89
CSNK2A1	13|15	14	0	0.62	0.89
TUBB2B	276|243	259.5	0	1.91	0.89
TUBB	316|291	303.5	0	1.65	0.89
MTDH	8|9	8.5	0	0.35	0.89
PPP2R5D	2|0	1	0	0.12	0.89
RFC2	3|3	3	0	0.38	0.89
OGT	2|4	3	0	0.31	0.89
ACOT7	0|2	1	0	0.1	0.89
MCM4	36|31	33.5	0	0.81	0.89
EIF4G1	8|8	8	0	0.08	0.89
TRMT2A	0|2	1	0	0.26	0.89
SHC1	2|2	2	0	0.3	0.89
RBM39	9|7	8	0	0.64	0.89
HNRNPA3	10|3	6.5	0	0.33	0.89
G3BP1	3|2	2.5	0	0.09	0.89
RPL7L1	2|3	2.5	0	0.33	0.89
ACACA	243|197	220	0	0.83	0.89
SF1	8|4	6	0	0.25	0.89
POLR1C	3|2	2.5	0	0.5	0.89
HDLBP	6|9	7.5	0	0.13	0.89
NCKAP1	2|0	1	0	0.08	0.89
PRKAA1	0|3	1.5	0	0.22	0.89
ATP6V1H	3|3	3	0	0.3	0.89

Supplementary Table 7: Crapome analysis of AP-MS and BioID data of MFGE8

AP-MS		BioID	
**Gene Symbol**	**Num of Expt. (found/total)**	**Gene Symbol**	**Num of Expt. (found/total)**
CYBA	0 / 411	RUNX3	0 / 411
NAGLU	0 / 411	R3HDM2	0 / 411
PPIC	0 / 411	TRIM13	0 / 411
ARSA	0 / 411	GTPBP2	2 / 411
ASIC1	0 / 411	ALDH1L2	3 / 411
ERLEC1	0 / 411	MRPS11	7 / 411
CLGN	0 / 411	DAD1	8 / 411
COCH	0 / 411	DCXR	15 / 411
MGRN1	0 / 411	ALDH1B1	20 / 411
ATP7B	0 / 411	RAB6B	34 / 411
MMP15	0 / 411	RAE1	36 / 411
IPO13	0 / 411	HSPB1	71 / 411
MAN1B1	0 / 411		
NGLY1	0 / 411		
UGGT2	0 / 411		
VARS2	0 / 411		
TMX4	0 / 411		
ITM2B	0 / 411		
SIL1	0 / 411		
KDELC1	0 / 411		
SLC27A3	0 / 411		
ADPGK	0 / 411		
SLC39A11	0 / 411		
FAM69A	0 / 411		
CES3	0 / 411		
TRIP10	0 / 411		
TTC19	0 / 411		
TTC13	0 / 411		
EDEM3	0 / 411		
GINS4	0 / 411		
DNAJC3	1 / 411		
TTI1	1 / 411		
KIAA0355	1 / 411		
POFUT1	1 / 411		
HLA-F	1 / 411		
C16ORF58	1 / 411		
HPS6	1 / 411		
CHST14	1 / 411		
TYSND1	1 / 411		
TOR1A	1 / 411		
TEX264	1 / 411		

CXCR4	1 / 411
NRP1	1 / 411
CBWD1	1 / 411
MBLAC2	1 / 411
ECE1	2 / 411
TCP11L1	2 / 411
GBA	2 / 411
ERAP1	2 / 411
OS9	2 / 411
LNPEP	2 / 411
NCSTN	2 / 411
SCPEP1	2 / 411
KLHL22	2 / 411
ZNF507	2 / 411
CACNA2D2	2 / 411
HEATR6	2 / 411
TTI2	2 / 411
MED24	3 / 411
EARS2	3 / 411
NUDT19	3 / 411
MSI1	3 / 411
ALDH4A1	3 / 411
SEL1L	3 / 411
TMX3	3 / 411
GAA	3 / 411
IRGQ	3 / 411
C6ORF120	3 / 411
FBLN1	3 / 411
MPI	3 / 411
EHD4	3 / 411
PON2	4 / 411
RHOT1	4 / 411
ALG1	4 / 411
UBQLN4	4 / 411
TRABD	4 / 411
PIGS	4 / 411
GGT7	4 / 411
FNDC3A	4 / 411
CARS2	4 / 411
PPT1	5 / 411
NME3	5 / 411
MED4	5 / 411
RARS2	5 / 411
PPOX	6 / 411
GGH	6 / 411
NDUFB11	6 / 411

L2HGDH	6 / 411
RAF1	6 / 411
DTYMK	6 / 411
UBFD1	6 / 411
SNX5	6 / 411
CNPY2	7 / 411
ABHD10	7 / 411
GCDH	9 / 411
RHOT2	9 / 411
SUCLA2	10 / 411
PNKD	10 / 411
ERAL1	10 / 411
UMPS	11 / 411
CTBP1	11 / 411
FLOT2	11 / 411
CDIPT	11 / 411
SLC39A7	11 / 411
ERP44	11 / 411
ACOT13	11 / 411
GALK1	12 / 411
COMT	12 / 411
APMAP	13 / 411
ANXA6	14 / 411
FAR1	14 / 411
RBFOX2	14 / 411
GPC4	14 / 411
TMEM165	14 / 411
UGGT1	15 / 411
HSDL2	15 / 411
ACOT1	17 / 411
LRRC1	18 / 411
TUBB3	
MYDGF	
FAM234A	
P3H3	
HDHD5	
FAF2	25 / 411
PKP2	25 / 411
RPS19BP1	25 / 411
HLA-C	26 / 411
HAX1	29 / 411
RAB6B	34 / 411
ERLIN2	45 / 411
SMCHD1	45 / 411
DDX54	49 / 411
YTHDF2	66 / 411
EXOSC10	70 / 411

SSR4	80 / 411
ACTC1	354 / 411
TUBA4A	377 / 411

Supplementary Table 8: Metascape result of AP-MS and BioID in MFGE8

AP-MS and BioID			
**Small molecule catabolic process**	**pvalue**		**pvalue**
Small molecule catabolic process	-4.4		

AP-MS	
**Protein deglycosylation**	**pvalue**
Protein deglycosylation	-11.5
N-glycan trimming in the ER and Calnexin/Calreticulin cycle	-10.8
Protein demannosylation	-10.8
Protein alpha-1,2-demannosylation	-10.8
ER Quality Control Compartment (ERQC)	-10.8
Calnexin/calreticulin cycle	-10.1
Protein processing in endoplasmic reticulum	-9.9
Endoplasmic reticulum mannose trimming	-9.5
ERAD pathway	-8.4
response to endoplasmic reticulum stress	-6.6
cellular protein catabolic process	-5.6
Asparagine N-linked glycosylation	-5.4
proteolysis involved in cellular protein catabolic process	-4.7
proteasomal protein catabolic process	-4.4
HRD1 complex	-4.4
ubiquitin-dependent ERAD pathway	-4.3
retrograde protein transport, ER to cytosol	-3.3
endoplasmic reticulum to cytosol transport	-3.3
proteasome-mediated ubiquitin-dependent protein catabolic process	-2.8
negative regulation of response to endoplasmic reticulum stress	-2.8
protein exit from endoplasmic reticulum	-2.7
Hh mutants that don't undergo autocatalytic processing are degraded by ERAD	-2.5
Hh mutants abrogate ligand secretion	-2.4
Defective CFTR causes cystic fibrosis	-2.4
Hedgehog ligand biogenesis	-2.3
ABC transporter disorders	-2.1
Signaling by Hedgehog	-2.1
regulation of response to endoplasmic reticulum stress	-2
**Mitochondrial tRNA aminoacylation**	
Mitochondrial tRNA aminoacylation	-5.4
tRNA Aminoacylation	-4.2

BioID	
**Carbohydrate derivative biosynthetic process**	
Carbohydrate derivative biosynthetic process	-9.4
Glycoprotein metabolic process	-9.2
Glycoprotein biosynthetic process	-6.5
Glycosylation	-5.8
Protein glycosylation	-5.1
Macromolecule glycosylation	-5.1
**Lysosome organization**	
Lysosome organization	-4.8
Lytic vacuole organization	-4.8
Lysosome	-3.3
Vacuole organization	-2.8
Cellular lipid catabolic process	-2.2
Lipid catabolic process	-2.1
**Cofactor metabolic process**	
Cofactor metabolic process	-4.8
Acyl-CoA metabolic process	-4.7
Thioester metabolic process	-4.7
Nucleotide metabolic process	-4.4
Nucleoside phosphate metabolic process	-4.1
Nucleoside bisphosphate metabolic process	-4.1
Ribonucleoside bisphosphate metabolic process	-4.1
Purine nucleoside bisphosphate metabolic process	-4.1
Sulfur compound metabolic process	-3.8
Ribonucleotide metabolic process	-3.8
Ribose phosphate metabolic process	-3.7
Coenzyme metabolic process	-3.6
Cofactor biosynthetic process	-3.6
Fatty acid metabolism	-3.5
Metabolism of lipids	-3.3
Purine ribonucleotide metabolic process	-3.3

tRNA aminoacylation for protein translation	-4.1	Purine nucleotide metabolic process	-3.1
tRNA aminoacylation	-3.9	Fatty-acyl-CoA metabolic process	-2.9
Amino acid activation	-3.9	Purine-containing compound metabolic process	-2.9
Aminoacyl-tRNA biosynthesis	-3.4	Fatty acid derivative biosynthetic process	-2.7
Cellular amino acid metabolic process	-2.5	Fatty acid derivative metabolic process	-2.7
**Protein quality control for misfolded or incompletely synthesized proteins**			
Protein quality control for misfolded or incompletely synthesized proteins	-4.9		
Protein folding	-4.6		
ER-associated misfolded protein catabolic process	-4.5		
Response to topologically incorrect protein	-4.2		
Cellular response to misfolded protein	-3.6		
Response to misfolded protein	-3.5		
Protein N-linked glycosylation	-3.1		
Nucleotide-sugar metabolic process	-3		
Cellular response to topologically incorrect protein	-2.9		

Supplementary Table 9: Primer sequence

Gene Symbol	Description	GenBank	Size (bp)	Primer sequence 5'→3' S/AS
**TBC1D9**	Homo sapiens TBC1 domain family member 9 (TBC1D9)	NM_015130	90	CAGCAGCCTCGTCTCCTCCAG/ GGGACGCTGTTGCCATTTAGG
**MFGE8**	Homo sapiens milk fat globule-EGF factor 8 protein (MFGE8), 2 transcripts	NM_005928	97	TGGGTAACAGGTGTGGTGACG/ ATTCGTGTCCATTAAGGCTGTAGG
**SLC16A6**	Homo sapiens solute carrier family 16 member 6 (SLC16A6), 2 transcipts	NM_001174166	88	TGGGCATTAGTCTGGGCATTGA/ GCTCCGATCCTTCCGAAAACT
**HPRT1**	Homo sapiens hypoxanthine phosphoribosyltransferase 1	NM_000194	157	AGTTCTGTGGCCATCTGCTTAGTAG/ AAACAACAATCCGCCCAAAGG
**GAPDH**	Homo sapiens glyceraldehyde-3-phosphate dehydrogenase	NM_002046	194	GGCTCTCCAGAACATCATCCCT/ ACGCCTGCTTCACCACCTTCTT
**ADNg**	Homo sapiens 3-beta-hydroxysteroid dehydrogenase/delta-5-delta-4-isomerase (3-beta-HSD) gene (intron)	M38180	260	GAAGGGCAGAGGTGGAACTAGAA/ AACAAAGACCAAAGACCAGTGAGA
